# Enzyme Repertoires and Genomic Insights into *Lycium barbarum* Pectin Polysaccharide Biosynthesis

**DOI:** 10.1093/gpbjnl/qzae079

**Published:** 2024-11-04

**Authors:** Haiyan Yue, Yiheng Tang, Aixuan Li, Lili Zhang, Yiwei Niu, Yiming Zhang, Hao Wang, Jianjun Luo, Yi Zhao, Shunmin He, Chang Chen, Runsheng Chen

**Affiliations:** State Key Laboratory of Epigenetic Regulation and Intervention, Institute of Biophysics, Chinese Academy of Sciences, Beijing 100101, China; CAS Key Laboratory of RNA Biology, Center for Big Data Research in Health, Institute of Biophysics, Chinese Academy of Sciences, Beijing 100101, China; State Key Laboratory of Epigenetic Regulation and Intervention, Institute of Biophysics, Chinese Academy of Sciences, Beijing 100101, China; College of Life Sciences, University of Chinese Academy of Sciences, Beijing 100049, China; German Cancer Research Center (DKFZ), 69120 Heidelberg, Germany; State Key Laboratory of Epigenetic Regulation and Intervention, Institute of Biophysics, Chinese Academy of Sciences, Beijing 100101, China; College of Life Sciences, University of Chinese Academy of Sciences, Beijing 100049, China; The Chinese University of Hong Kong, Hong Kong Special Administrative Region 999077, China; State Key Laboratory of Epigenetic Regulation and Intervention, Institute of Biophysics, Chinese Academy of Sciences, Beijing 100101, China; College of Life Sciences, University of Chinese Academy of Sciences, Beijing 100049, China; CAS Key Laboratory of RNA Biology, Center for Big Data Research in Health, Institute of Biophysics, Chinese Academy of Sciences, Beijing 100101, China; College of Life Sciences, University of Chinese Academy of Sciences, Beijing 100049, China; State Key Laboratory of Epigenetic Regulation and Intervention, Institute of Biophysics, Chinese Academy of Sciences, Beijing 100101, China; College of Life Sciences, University of Chinese Academy of Sciences, Beijing 100049, China; State Key Laboratory of Epigenetic Regulation and Intervention, Institute of Biophysics, Chinese Academy of Sciences, Beijing 100101, China; College of Life Sciences, University of Chinese Academy of Sciences, Beijing 100049, China; State Key Laboratory of Epigenetic Regulation and Intervention, Institute of Biophysics, Chinese Academy of Sciences, Beijing 100101, China; College of Life Sciences, University of Chinese Academy of Sciences, Beijing 100049, China; Beijing University of Chinese Medicine, Beijing 100029, China; Key Laboratory of Intelligent Information Processing, Advanced Computer Research Center, Institute of Computing Technology, Chinese Academy of Sciences, Beijing 100190, China; State Key Laboratory of Epigenetic Regulation and Intervention, Institute of Biophysics, Chinese Academy of Sciences, Beijing 100101, China; CAS Key Laboratory of RNA Biology, Center for Big Data Research in Health, Institute of Biophysics, Chinese Academy of Sciences, Beijing 100101, China; College of Life Sciences, University of Chinese Academy of Sciences, Beijing 100049, China; CAS Center for Excellence in Biomacromolecules, Institute of Biophysics, Chinese Academy of Sciences, Beijing 100101, China; State Key Laboratory of Epigenetic Regulation and Intervention, Institute of Biophysics, Chinese Academy of Sciences, Beijing 100101, China; College of Life Sciences, University of Chinese Academy of Sciences, Beijing 100049, China

**Keywords:** *Lycium barbarum*, *Lycium barbarum* pectin polysaccharide, Phylogenetic expansion, lncRNA, Rhamnogalacturonan I rhamnosyltransferase

## Abstract

*Lycium barbarum*, a member of the Solanaceae family, is an important eudicot with applications in both food and medicine. *L. barbarum* pectin polysaccharides (LBPPs) are key bioactive compounds of *L. barbarum*, notable for being among the few polysaccharides with both biocompatibility and biomedical activity. Although studies have analyzed the functional properties of LBPPs, the mechanisms underlying their biosynthesis and transport by key enzymes remain poorly understood. In this study, we assembled a 2.18-Gb reference genome of *L. barbarum*, reconstructed the first complete biosynthesis pathway of LBPPs, and elucidated the sugar transport system. We also characterized the important genes responsible for backbone extension, sidechain synthesis, and modification of LBPPs. Furthermore, we characterized the long non-coding RNAs (lncRNAs) associated with polysaccharide metabolism. We identified a specific rhamnogalacturonan I (RG-I) rhamnosyltransferase, RRT3020, which enhances RG-I biosynthesis within LBPPs. These newly identified enzymes and pivotal genes endow *L. barbarum* with unique pectin biosynthesis capabilities, distinguishing it from other Solanaceae species. Our findings thus provide a foundation for evolutionary studies and molecular breeding to expand the diverse applications of *L. barbarum*.

## Introduction

*Lycium barbarum*, also known as goji or wolfberry, is a perennial shrub native to temperate and subtropical regions. Some *Lycium* species are cultivated as economic crops across Asia and Europe due to their health benefits, including blood replenishment and health enhancement [1]. Among these, *L. barbarum* is particularly valued worldwide for its unique group of extractable pectins, which are known for their immunomodulatory and antioxidant properties.

Decades of pharmacological research have enabled the isolation and identification of a type of *L. barbarum*-specific metabolites, termed *L. barbarum* pectin polysaccharides (LBPPs), which are now used in health foods and oral drugs [2,3]. LBPPs exhibit a broad spectrum of biological activities, including antiaging, antioxidant, anticancer, and hepatoprotective effects [2,4–6]. Recent studies suggest that LBPPs may also decrease cortisol levels and alleviate posttraumatic stress disorder (PTSD) symptoms in patients with COVID-19 and COVID-19 survivors [7–9]. Furthermore, biological assays have indicated that LBPPs can modulate immunity and inhibit cancer cell growth [10]. Thus, *L. barbarum* has a high nutraceutical value because of its specific polysaccharides.

A lack of genomic resources and in-depth analyses has hindered the study of LBPP biosynthesis and transport. To address this gap, we assembled a high-quality chromosome-scale genome of 2.18 Gb. Recently, a 1.6-Gb genome of the cultivar *L. barbarum* has been reported [11], which is smaller than the flow cytometry estimate of 2.13 Gb [12]. Therefore, we performed whole-genome analysis and chromosome-wise alignments with the 1.6-Gb assembly. Our results indicated significant differences, including multiple large chromosome segments that are novel to this assembly, especially in regions related to polysaccharide metabolism. 

Utilizing the current genome, phylogenetic and comparative analyses revealed that key enzymes involved in LBPP biosynthesis, such as LyRRTs (GT106), LyGAUTs (GT8), LyPAEs (CE13), LyGALTs (GT31), and LySWEETs, have undergone remarkable alterations. These enzymes are mainly responsible for extending and modifying the main LBPP backbone, synthesizing sidechains, and mediating sugar transportation in *L. barbarum*. In particular, polysaccharide metabolism-related long non-coding RNAs (lncRNAs) are more conserved and exhibit greater tissue specificity. Importantly, we identified specific genes involved in LBPP synthesis. For example, a rhamnogalacturonan I (RG-I) rhamnosyltransferase encoded by *RRT3020* was observed to significantly increase the expression of the pectin RG-I.

The genomic landscape offers a novel glimpse into the biosynthesis and transport of pectins, suggesting the potential for a variety of unique pectins in *L. barbarum*. These resources and findings are expected to provide a molecular basis for LBPP metabolic engineering and further research.

## Results

### Genome evolution and comparative analysis

Phenotypes are usually regulated by genomic attributes. To elucidate the evolution of LBPP biosynthesis and transport, we constructed a chromosome-level genome assembly of *L. barbarum*. The genome attributes of *L. barbarum* were estimated through survey analysis, which revealed high heterozygosity (0.96) and repeat content (70.21%) (Figures S1 and S2; Table S1). Therefore, we adopted seven sequencing techniques combined with a self-built chromosome conformation capture approach and multiple assembly methods to obtain a high-quality genome of *L. barbarum* (Figure S3; Tables S2 and S3). Eventually, we obtained a chromosome-scale 2.18-Gb genome (Figure S4; Table S3), which was consistent with the 2.13-Gb estimate detected using flow cytometry (4.25 Gb for 2C DNA content) [12]. The genome completeness was estimated to be 98.7% (Table S4).

We then performed a whole-genome alignment on a per-chromosome basis to compare our assembly with the genome reported by Cao and colleagues [11]. We noted significant differences in chromosome configuration and large sections of chromosomes unaligned. Notably, our assembly contains larger chromosomes, some of which (*e.g.*, chromosome 4) incorporate segments that were split into multiple chromosomes in the previous assembly (Figure S5). Multiple large chromosomal segments novel to this assembly (LyBar), especially those involved in polysaccharide metabolism, were identified.

By combining transcriptome-based annotation, homology-based prediction, and *de novo* prediction, we identified 31,911 high-confidence protein-coding genes, 96.81% of which were annotated (Table S5). The *L. barbarum* assembly is rich in repetitive elements, accounting for 67.75% (1,479,425,826 bp) of the genome. These include retrotransposons (52.16%), DNA transposons (2.91%), low-complexity regions (0.17%), tandem repeats (2.67%), and unclassified elements (9.84%), with long terminal repeat (LTR) retrotransposons being the most abundant (Table S6).

**Figure 1:**
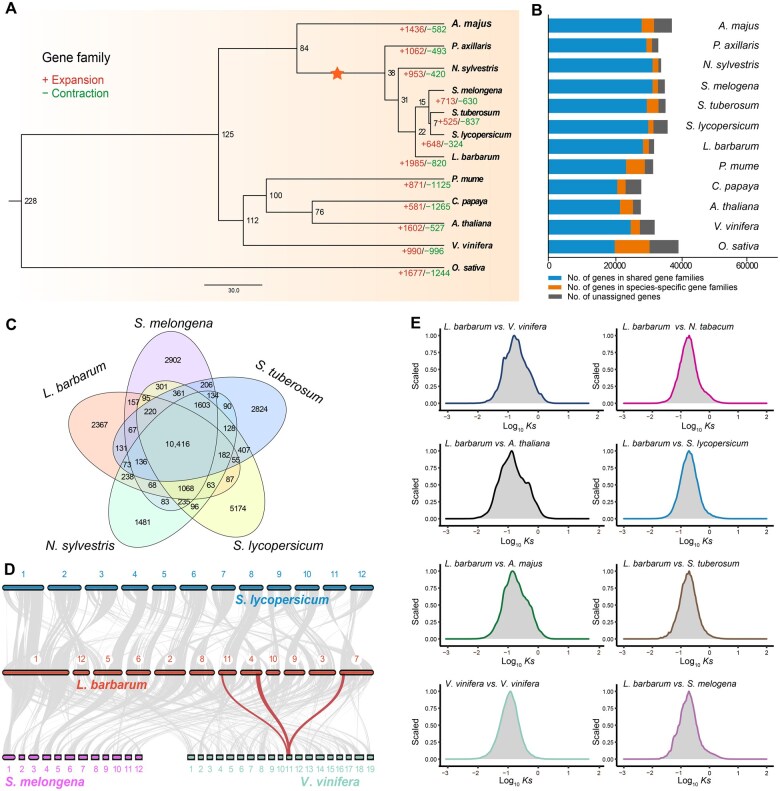
Evolutionary and comparative genomic analyses **A**. Phylogenetic tree depicting the evolutionary relationships and divergence time of 12 species. The numbers in red and green on the branches indicate the numbers of expanded (+) and contracted (−) gene families, respectively, in each species during evolution. The numbers on the branch nodes indicate divergence time. The orange star represents the WGT event that occurred in Solanaceae species. **B**. Distribution of genes in species-specific gene families (orange) and shared gene families (blue) as well as unassigned genes (gray). **C**. Venn diagram illustrating unique and shared gene families among *S. melongena*, *L. barbarum*, *N. sylvestris*, *S. lycopersicum*, and *S. tuberosum*. **D**. Intergenomic syntenic analysis between *L. barbarum* and *S. lycopersicum*, *S. melongena*, and *V. vinifera*. Syntenic pairs of genomic blocks are linked by gray lines. Red lines mark a representative syntenic block with one copy in *V. vinifera* and three copies in *L. barbarum*. **E**. Density distribution of synonymous substitutions per synonymous site (*Ks*) for paralogous genes based on the screened collinear regions among *L. barbarum*, other Solanaceae species (*N. tabacum*, *S. lycopersicum*, *S. tuberosum*, and *S. melongena*), and non-Solanaceae model plants (*V. vinifera*, *A. majus*, and *A. thaliana*). Only blocks with > 10 genes were retained. The synonymous substitution rate per gene (*Ks*) between each pair of species is shown in the distribution curves. *A. majus*, *Antirrhinum majus*; *P. axillaris*, *Petunia axillaris*; *N. sylvestris*, *Nicotiana sylvestris*; *S. melongena*, *Solanum melongena*; *S. tuberosum*, *Solanum tuberosum*; *S. lycopersicum*, *Solanum lycopersicum*; *L. barbarum*, *Lycium barbarum*; *P. mume*, *Prunus mume*; *C. papaya*, *Carica papaya*; *A. thaliana*, *Arabidopsis thaliana*; *V. vinifera*, *Vitis vinifera*; *O. sativa*, *Oryza sativa*; *N. tabacum*, *Nicotiana tabacum*; WGT, whole-genome triplication.

To elucidate the evolutionary status of *L. barbarum*, a phylogenetic tree was constructed using *L. barbarum*, *Solanum tuberosum*, *Solanum lycopersicum*, and 9 other species (**Figure 1**A; Table S7). In total, 1985 gene families were expanded in *L. barbarum*, which were significantly enriched in the amino sugar and nucleotide sugar metabolism and protein export signaling pathways (Table S8). Analysis of transcription factor (TF) expansions and contractions revealed that the AP2 gene family [orthologous to *Arabidopsis thaliana*  *AP2* (At4G13040)], the AGL24 of Type II MADS-box gene family, the RWP-RK gene family (orthologous to *S. lycopersicum* Solyc12g011190), and the NF-YA gene family were expanded in *L. barbarum* (Figures S6−S9; Table S9), whereas the NF-YB gene family was contracted in *L. barbarum* (Figure S10). Collectively, these changes in TFs may contribute to *L. barbarum*’s traits such as stress resistance [13], flowering time regulation [14], and root development [15,16].

For comparative genomic analysis, we constructed a gene set by integrating the longest protein-coding sequences of *L. barbarum* and 11 other species. We identified 438 species-specific gene families (1737 genes) in *L. barbarum* (Figure 1B; Table S10). Compared with *Solanum melongena*, *S. tuberosum*, *S. lycopersicum*, and *Nicotiana sylvestris*, 2367 gene families were specific to *L. barbarum* (Figure 1C). Intergenomic synteny analysis revealed 19,570 (16,921) gene pairs and 626 (713) synteny blocks between *L. barbarum* and *S. lycopersicum* (*S. melongena*) (Figure 1D). Synonymous substitution distribution and synteny analyses indicated that *L. barbarum* underwent an ancient whole-genome triplication (WGT) event shared with other Solanaceae plants (Figure 1A, D, and E).

### Expansion and fruit-specific expression of sugar metabolism-related genes in *L. barbarum*

**Figure 2:**
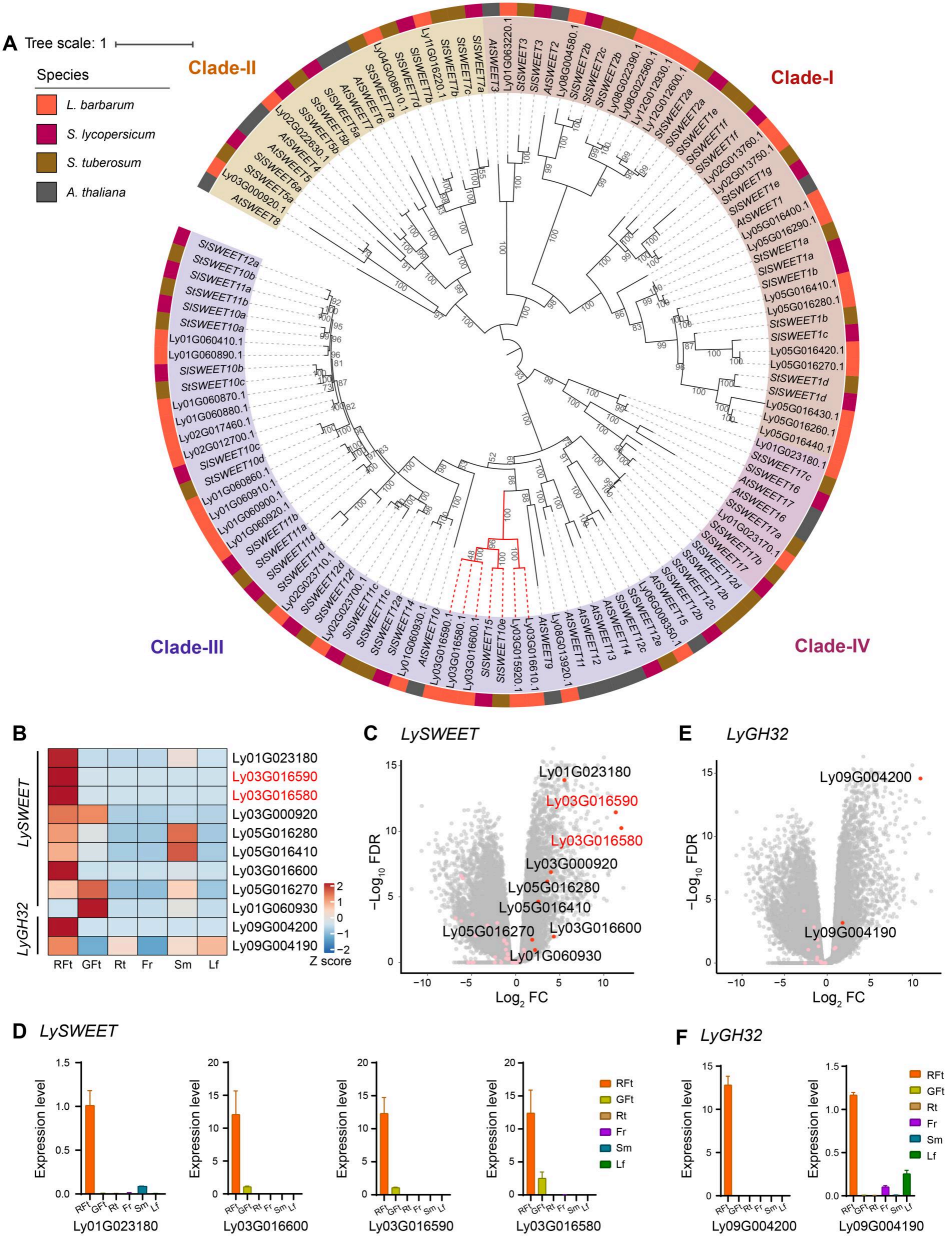
Expansion and fruit-specific expression of sugar transporter genes in *L. barbarum* **A**. Maximum likelihood phylogenetic tree of *SWEET* genes from *A. thaliana*, *S. lycopersicum*, *S. tuberosum*, and *L. barbarum*. The bootstrap confidences are labeled on the branch nodes. Tomato *SlSWEET15* and its orthologs are highlighted with red branches. **B**. Heatmap showing the higher expression of *LySWEET* and *LyGH32* genes in mature (red) fruits. **C**. Dot plot showing the differential expression of *LySWEET* genes in red fruits compared to the average level in all samples. Dots in red and pink denote significantly up-regulated genes and other genes in the family, respectively. **D**. qRT-PCR validation of top differential expression of *LySWEET* genes. Data are represented by mean ± SE (*n* = 3). **E**. Dot plot showing the differential expression of *LyGH32* genes in red fruits compared to the average level in all samples. Dots in red and pink denote significantly up-regulated genes and other genes in the family, respectively. **F**. qRT-PCR validation of top differential expression of *LyGH32* genes. Data are represented by mean ± SE (*n* = 3). SWEET, sugar will eventually be exported transporter; RFt, red fruit; GFt, green fruit; Rt, root; Fr, flower; Sm, stem; Lf, leaf; SE, standard error; FC, fold change; FDR, false discovery rate.

Sugar will eventually be exported transporters (SWEETs), a family of sugar transporters, facilitate sucrose and hexose efflux across cell membranes [17,18]. The SWEET family is enriched among the expanded genes of the *L. barbarum* genome (Figure S11A). This gene family can be divided into four clades and plays diverse roles across a variety of tissues [19,20–23]. We identified 43 genes encoding LySWEET transporters, arranged in several tandem repeat segments (**Figure 2**A, Figure S11D; Table S11), which are susceptible to gene duplication in *L. barbarum*. Phylogenetic analysis indicated that the *L. barbarum* genome contains a higher number of *SWEET* genes than tomato (29 genes) [24], potato (35 genes) [25], and many other plant species. Notably, some of the most differentially expressed genes (DEGs) in red fruit encode expanded SWEET transporters in *L. barbarum* (Figure 2B and C), and their expression levels were validated by quantitative real-time reverse transcription polymerase chain reaction (qRT-PCR) (Figure 2D). In particular, the *L. barbarum* genome contains a series of tandemly repeated genes orthologous to tomato *SlSWEET15* (*NEC1*) (Figure 2A, highlighted in red), which is responsible for fruit development by unloading sucrose [26,27]. This series of genes is highly expressed, with two of the genes also being among the top DEGs between mature fruit and other tissues (Figure 2C). Furthermore, some of the tandemly repeated genes exhibited significantly higher expression in red wolfberry than in the yellow and black fruits (Figure S11B and C).

We also identified 32 sugar transporter genes based on protein annotations. In addition to the small sucrose transporter (SUT) family, most identified proteins were monosaccharide transporters, including polyol transporters (PLTs), sugar facilitator proteins (SFPs)/ED6-like family proteins, inositol transporters (INTs), tonoplast monosaccharide transporters (TMTs), and plastidic glucose transporters (pGlcTs). Several families, including the SUT and SFP, exhibited increased expression in mature fruit compared with other tissues (Figure S11E).

Another group of expanded genes encode invertases, which can be divided into two types according to their optimal pH: neutral/alkaline invertases (also known as cytoplasmic invertases, CINs) and acid invertases, with the latter divided into cell wall invertases (CWINs) and vacuolar invertases (VINs) [28–30]. Our phylogenetic and functional genomic evidence indicates that invertases, including LyCWINs (GH32), LyVINs (GH32), and LyCINs (GH100), play a vital role in regulating carbohydrate partitioning in the ripe fruit of *L. barbarum* (Figures S12A, S13, and S14). Among these, LyCWINs and LyVINs (GH32) are functionally related to the LySWEET family, particularly during fruit development [17,31,32]. Several LyGH32-coding genes formed tandem repeat clusters (Figure S12B and C) and displayed marked red fruit-specific expression patterns among different organs and varieties, *e.g.*, Ly09G004190 and Ly09G004200 (Figure 2E, Figure S13), as confirmed through qRT-PCR (Figure 2F). 

### Tissue-specific expression patterns point to polysaccharide biosynthesis and secondary metabolic pathways in *L. barbarum*

Transcriptomic analyses were performed on samples from six tissues of *L. barbarum* var. *barbarum*: green fruit, red fruit, root, flower, stem, and leaf (Figure S15A), as well as on mature fruits of *L. barbarum* var. *auranticarpum* (yellow wolfberry) and *L. ruthenicum* (black wolfberry). Differential gene expression and Gene Ontology (GO) enrichment analyses in these tissues revealed that the up-regulated DEGs in green fruit and red fruit were significantly enriched in the polysaccharide biosynthesis and terpenoid metabolism pathways, respectively (Figure S15B). As expected, we identified a large number of DEGs specific to each of the varieties, including genes involved in the zeaxanthin dipalmitate and anthocyanin biosynthesis pathways (Figures S16–S18). Overall, transcriptome profiling reveals that DEGs in fruits are closely related to polysaccharide metabolism.

### Genome-wide identification of *L. barbarum* carbohydrate-active enzymes and transcriptomic associations of pectin-related enzyme genes

To identify *L. barbarum*-specific genes involved in LBPP biosynthesis, we systematically profiled the *L. barbarum* genome for genes involved in carbohydrate metabolism, especially polysaccharide biosynthesis and its regulation, using the CAZy database which classifies enzymes related to oligosaccharide and polysaccharide metabolism [33]. By mapping the predicted protein sequences of *L. barbarum*, *S. lycopersicum*, and *S. melogena* to the Hidden Markov Model (HMM) profiles of the CAZy database [33,34], we identified a comparable number of carbohydrate-active enzyme (CAZyme) genes across the three Solanaceae species, with 1180, 1267, and 1249 genes for *S. lycopersicum*, *S. melogena*, and *L. barbarum*, respectively. The predicted CAZyme genes in *L. barbarum* belonged to 110 families (Table S12). Among these, 1218 CAZyme genes were successfully mapped to our chromosome-scale assembly. When compared with the previously published assembly by Cao et al. [11], 675 CAZyme genes were aligned with more than 90% sequence identity, whereas the remaining 543 genes were novel in our assembly. These novel genes were particularly enriched in cell wall organization and biogenesis, polysaccharide metabolism, and glycosylation (Figure S5B and C).

**Figure 3:**
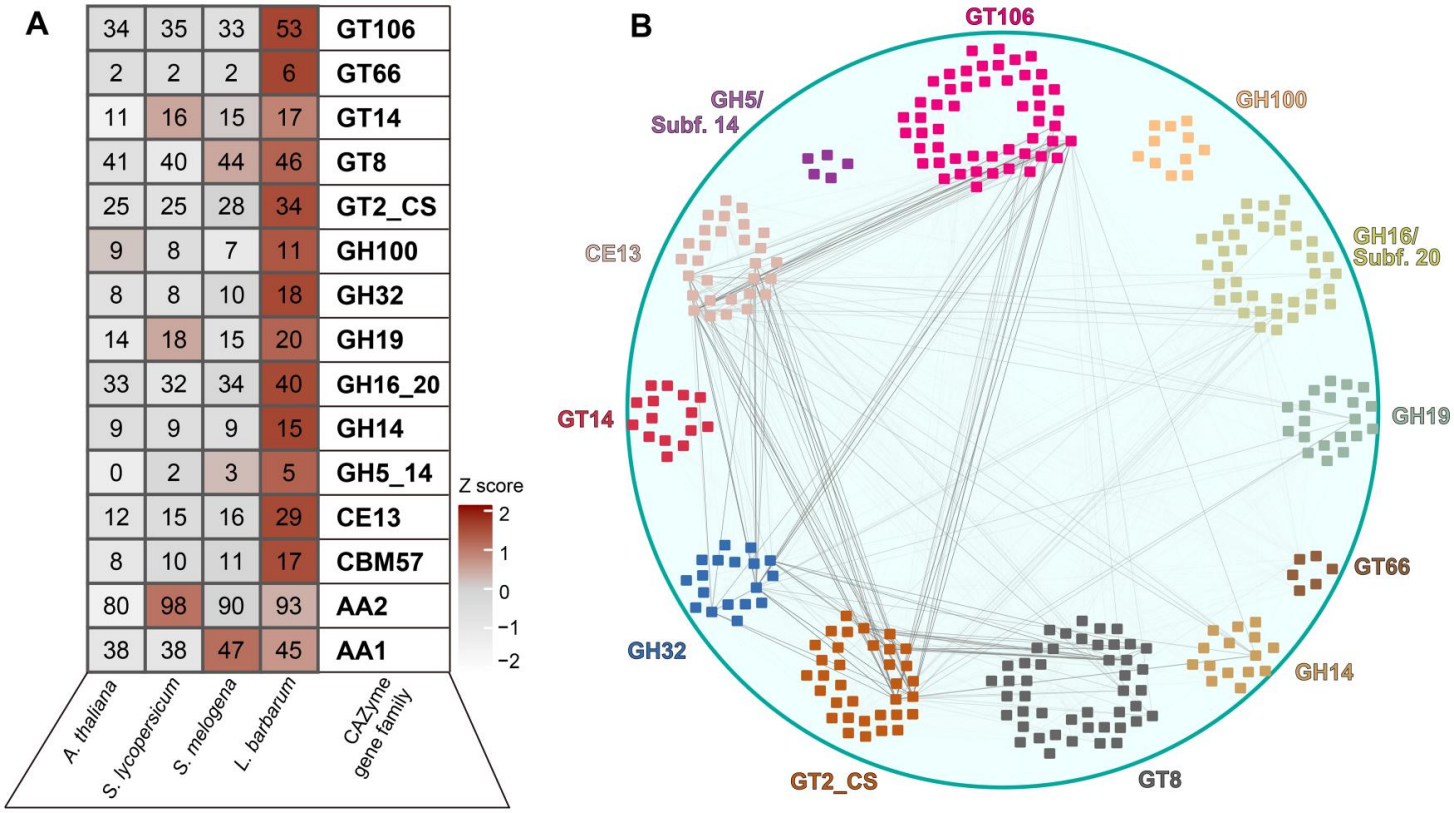
Expansion of CAZme gene family involved in pectin biosynthesis in *L. barbarum* **A**. Expansion of major CAZyme gene families in *L. barbarum* compared with those in *S. lycopersicum*, *S. melongena*, and *A. thaliana*. **B**. WGCNA of expanded CAZyme genes in *L. barbarum* shows strong correlations among cell wall-related CAZyme genes in the families GT106 (glycosyltransferase), CE13 (carbohydrate esterase), GH32 (glycoside hydrolase), GT2, and GT8. Edge colors indicate correlation strength. CAZyme, carbohydrate-active enzyme; WGCNA, weighted gene coexpression network analysis.

Among all the CAZyme gene families identified in *L. barbarum*, 15 exhibited an increased gene count (difference > 2) compared with those in other plants investigated, with small enzyme families (≤ 2 members on average) omitted (**Figure 3**A). Weighted gene coexpression network analysis (WGCNA) [35] revealed a strong correlation between the expression of genes from the CE13, GH32, GT2, GT8, and GT106 families (Figure 3B). Notably, all these families except for the invertase family GH32 were associated with the biosynthesis of pectins and cellulose.

### Pectin biosynthesis pathway in *L. Barbarum* and key enzyme analysis

**Figure 4:**
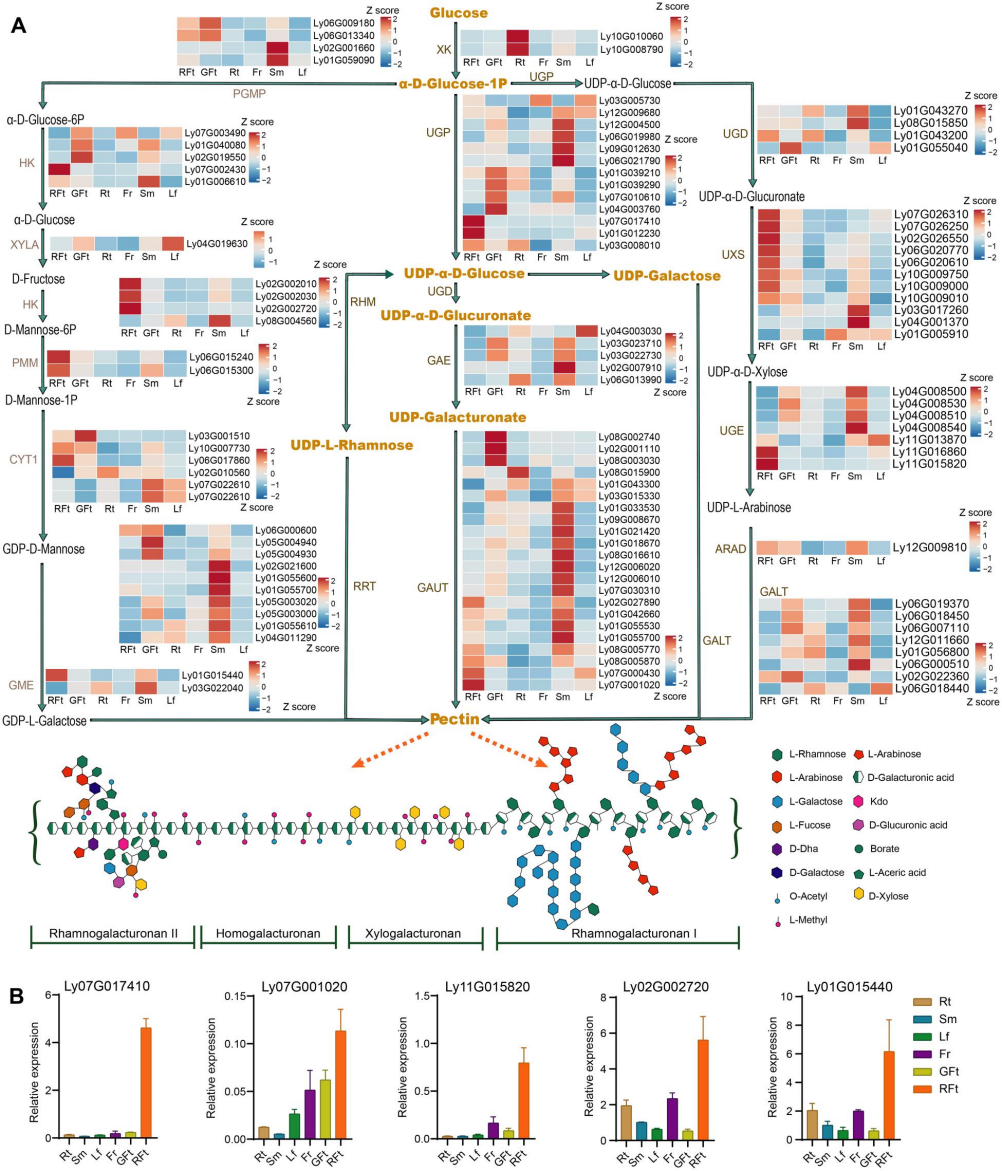
Phylogenetic repertoires for pectin biosynthesis and remodeling **A**. Proposed pectin biosynthesis pathway in *L. barbarum* by integrating genomic and transcriptomic data with the monosaccharide metabolism pathways from the KEGG pathway annotations. Essential enzymes are labeled: α-1,4-galacturonosyltransferase (GAUT), RG-I:rhamnosyltransferase (RRT), arabinan arabinosyltransferase (ARAD), β-1,3-galactosyltransferase (GALT), glycerate 2-kinase (XK), phosphoglucomutase (PGMP), hexokinase (HK), xylose isomerase (XYLA), phosphomannomutase (PMM), mannose-1-phosphate guanylyltransferase 1 (CYT1), GDP-mannose 3,5-epimerase (GME), UTP-glucose-1-phosphate uridylyltransferase (UGP), UDP-glucose 6-dehydrogenase (UGD), UDP-glucuronate 4-epimerase (GAE), trifunctional UDP-glucose 4,6-dehydratase/UDP-4-keto-6-deoxy-D-glucose 3,5-epimerase/UDP-4-keto-L-rhamnose-reductase (RHM), UDP-glucuronic acid decarboxylase (UXS), UDP-glucose 4-epimerase (UGE). The expression level (FPKM) of each gene was log_10_-transformed and normalized to Z score in six organs. **B**. qRT-PCR validation of gene expression for several key enzymes involved in pectin biosynthesis. Data are represented by mean ± SE (*n* = 3). RG-I, rhamnogalacturonan I; FPKM, fragments per kilobase of transcript per million mapped reads.

Pectins, notable for their biocompatibility and biomedical activity, have garnered increasing attention in recent years [11]. Generally, pectin polysaccharides comprise four main structures: rhamnogalacturonan I (RG-I), homogalacturonan (HG), xylogalacturonan (XG), and rhamnogalacturonan II (RG-II) [36]. However, few studies have investigated pectins in *L. barbarum*, especially the genetic information of LBPPs. Here, we outlined the biosynthesis pathway of pectins, and labeled the differential tissue expression of key enzymes in *L. barbarum* (**Figure 4**A), including rhamnosyltransferase (GT106-RRT) [37], α-1,4-galacturonosyltransferase (GT8-GAUT) [38], and pectin acetylesterase (CE13-PAE). RG-I rhamnosyltransferases (RRTs) and GAUTs are principally responsible for extending the saccharide unit [→2)-α-L-Rha-(1 → 4)-α-D-GalUA-(1→], which is the main repeating unit of the RG-I backbone. The Gal*p*A residues of the RG-I backbone may be *O*-acetylated on C-2 and/or C-3 [39]. The degree of acetylation is regulated by pectin acetylesterases (PAEs) [40].

The rhamnose residues of the saccharide unit are branched with α-(1,5)-linked arabinan, β-(1,4)-linked galactan, and diverse arabinogalactan structures [41,42]. *ARAD1* is predicted to regulate the biosynthesis of the arabinan sidechains of RG-I by encoding an arabinan α-1,5-arabinosyltransferase [43]. Arabinose and galactose, the two most abundant monosaccharides in *L. barbarum* polysaccharides, form the main bioactive sidechains of pectins [44]. The biosynthesis pathway of LBPPs involves the branches of arabinan (Figure 4A, right) and galactan (Figure 4A, left), which are catalyzed by LyARAD (GT77) (Figure S19) and galactosyltransferase LyGALT (GT31) (Figure S20), respectively. The genes involved in the biosynthesis of arabinose and galactose branches showed significantly higher expression in mature red fruit, and the expression of several genes, such as *LyRHM* (Ly02G002720) and *LyGAUT* (Ly07G001020), was validated by qRT-PCR in *L. barbarum* (Figure 4B; Table S13). Besides the mainchain, the structural diversity and abundance of RG-I sidechains provide a foundation for the biomedical functions of LBPPs.

### lncRNAs associated with carbohydrate metabolism

**Figure 5:**
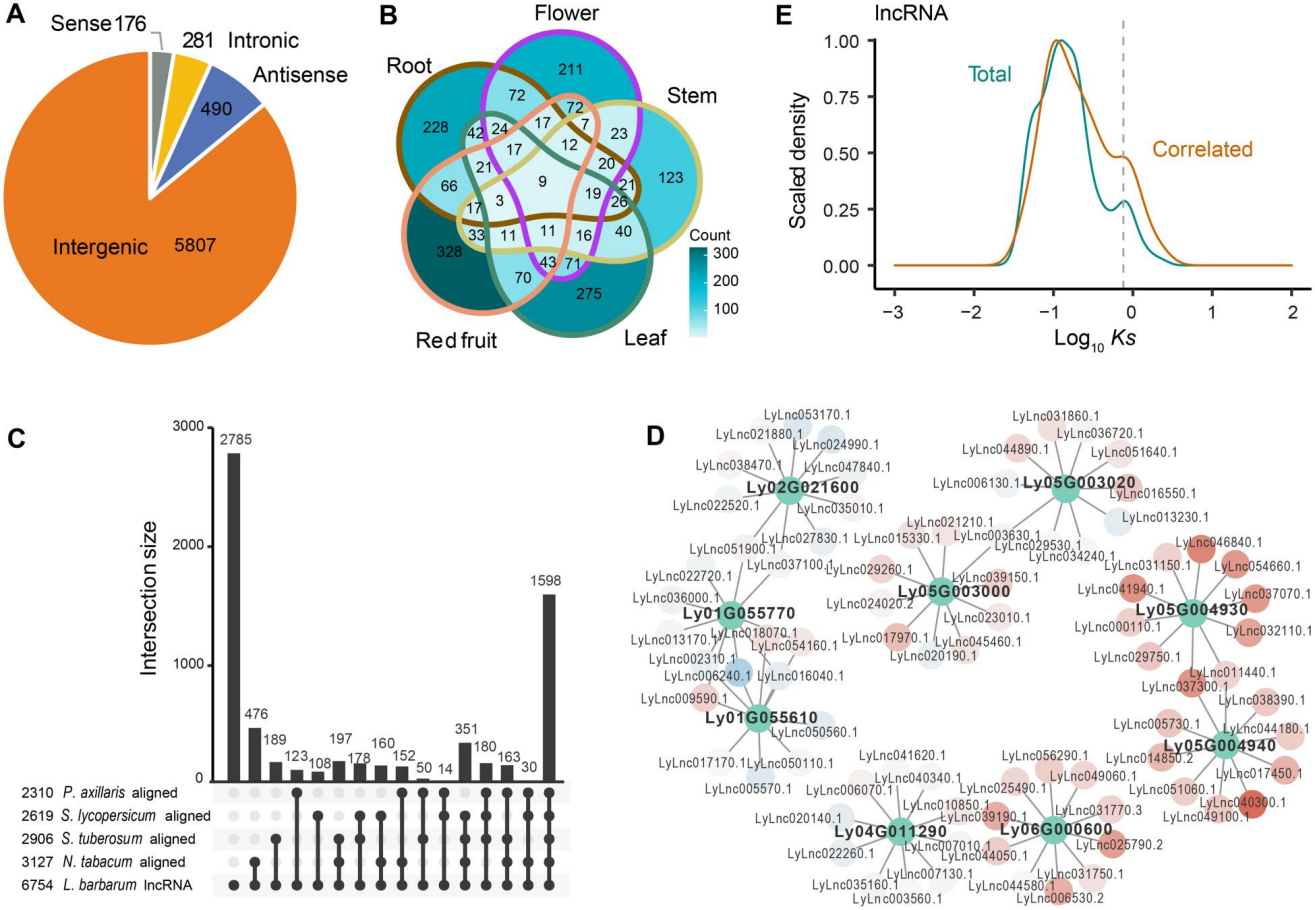
Pectin metabolism-related lncRNAs in *L. barbarum* **A**. Classification of lncRNAs identified in *L. barbarum*. **B**. Venn diagram showing the overlap of up-regulated lncRNAs among root, stem, flower, leaf, and red fruit tissues in *L. barbarum*. **C**. Number of *L. barbarum* lncRNAs aligned to genomes of related species via a BLAST search as well as unaligned lncRNAs. **D**. Ten most correlated lncRNAs for each of the selected *RRT* genes in the WGCNA network. Red indicates up-regulated in immature (green) fruit; blue indicates down-regulated in immature (green) fruit. **E**. *Ks* distribution of all lncRNAs and the top 5 lncRNAs correlated to each CAZyme gene. The dashed line indicates the position of the WGT event. lncRNA, long non-coding RNA.

Accumulating evidence over the past decade has demonstrated that lncRNAs play crucial roles in gene regulation [45]. Although lncRNAs have been extensively annotated and collected in the Noncode database [46], data are not yet available for *L. barbarum*. Non-coding regions account for 98.09% of the *L. barbarum* genome, which is larger than that in many other Solanaceae plants (Table S14), suggesting that the most significant divergence occurs in non-coding regions. We identified 6754 novel lncRNA transcripts from the *L. barbarum* transcriptome (**Figure 5**A and B). A BLAST alignment of the *L. barbarum* genome against those of four other Solanaceae species (*P. axillaris*, *S. lycopersicum*, *S. tuberosum*, and *N. tabacum*) revealed 2785 *L. barbarum*-specific lncRNAs (Figure 5C). We further constructed a WGCNA network and analyzed the transcription correlations between CAZyme genes and lncRNAs, and found that lncRNAs were highly correlated with key *RRT* genes (Figure 5D, Figure S21B). The frequency distribution of synonymous substitutions in all lncRNAs showed a peak related to the Solanaceae WGT event (Figure 5E). However, this WGT peak is more prominent in synonymous substitutions of lncRNAs highly correlated with CAZyme RRT transcription (Figure 5E, Figure S21E and F), indicating that lncRNAs involved in carbohydrate metabolism are more conserved and more likely to be preserved from the Solanaceae WGT event. This observation suggests that potential regulatory lncRNAs involved in polysaccharide metabolism may be linked to the divergence of Solanaceae plants after the WGT event.

We compared the lncRNA expression profiles across different tissues of *L. barbarum* and observed that a large number of the identified lncRNAs were expressed in a tissue-specific manner (Figure 5B). Furthermore, the lncRNA expression displayed higher tissue specificity than that of coding genes (Figure 5B, Figure S21A, C, and D). Additionally, the lncRNA expression patterns of the three variants were highly divergent (Figure S21C).

### *L. barbarum*-specific RRTs expanded the glycosyltransferase family and increased RG-I pectin biosynthesis

**Figure 6:**
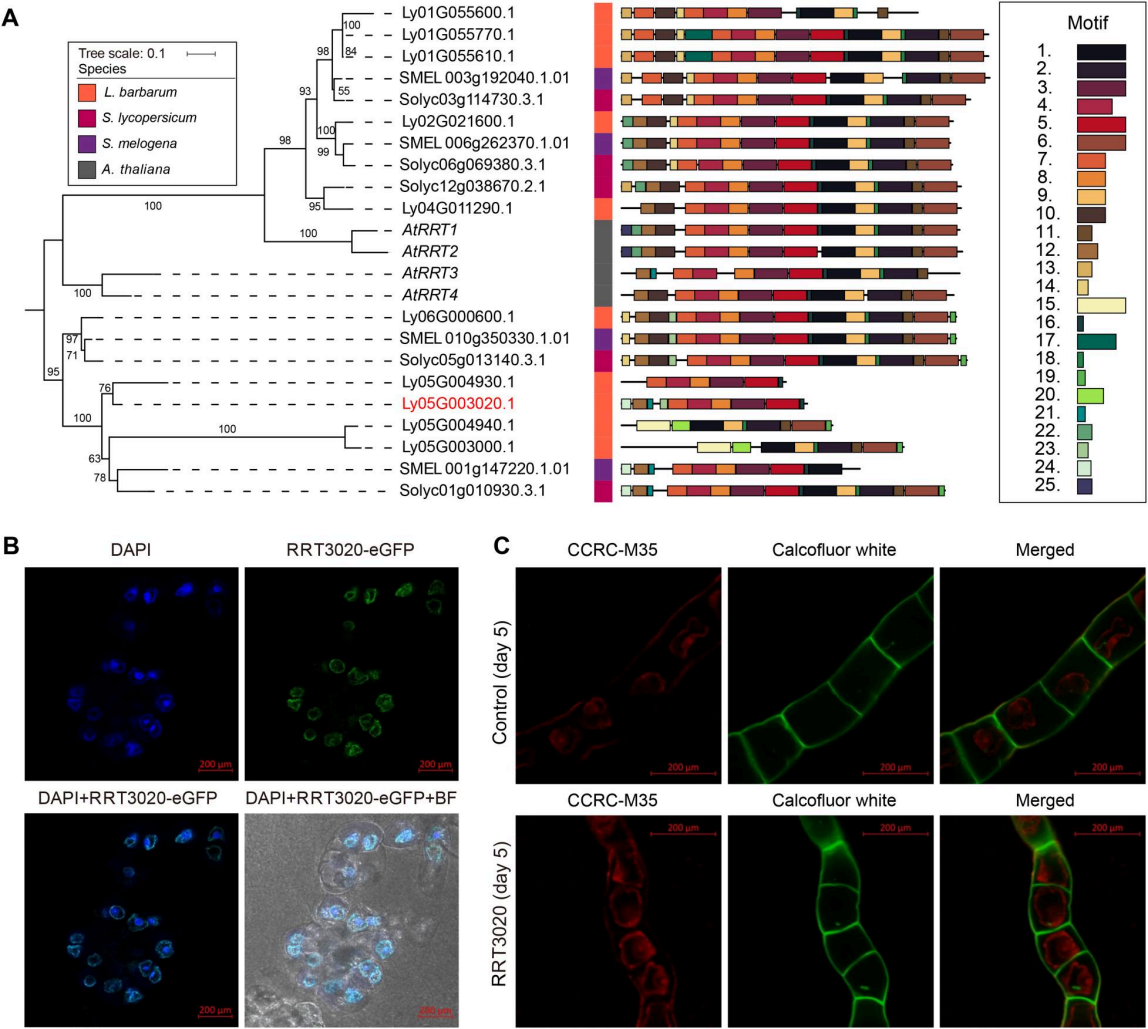
Novel RRTs increase RG-I biosynthesis in *L. barbarum* **A**. Evolution and protein structures of *RRT* genes in Solanaceae. A maximum likelihood phylogenetic tree was constructed for clade *RRT1–4* (GT106) genes from *A. thaliana* and three Solanaceae species. The bootstrap confidences are labeled on the branch nodes. Significantly conserved motifs were identified *de novo* from the list of protein sequences using MEME [47]. **B**. Immunofluorescence images in RRT3020-expressing BY-2 cells with eGFP (green) and DAPI (blue) showing RRT3020 localization and nucleus, respectively. This experiment was repeated three times with similar results. Scale bar, 200 µm. **C**. Whole-mount immunolabeling assays on BY-2 cells expressing RRT3020 as well as BY-2 cells transformed with empty vector (control). RG-I accumulation was detected by CCRC-M35 antibody and cellulose was stained by Calcofluor white. Two independent experiments were performed. Scale bar, 200 µm. eGFP, enhanced green fluorescent protein; DAPI, 4′,6-diamidino-2-phenylindole; BF, bright field.

Both coding and non-coding genes are involved in polysaccharide biosynthesis. Among the CAZyme gene families, GT106 (Figure 3A, Figure S22), a family encoding glycosyltransferases (GTs) including pectin RRTs, exhibited the most significant expansion. This family is responsible for transferring rhamnose residues to the pectic RG-I backbone. Phylogenic analysis revealed that the RRT1–4 (GT106) clade contained 10 genes in *L. barbarum*, compared with 4 in *A. thaliana*, 4 in eggplant, and 5 in tomato (**Figure 6**A, Figure S23A). MEME [47] analysis identified 25 significantly conserved motifs within the RRT protein sequences. Notably, *L. barbarum* RRTs contained novel motifs absent in other Solanaceae RRT enzymes. Four newly identified genes (Ly05G003020, Ly05G004940, Ly05G004930, and Ly05G003000) were discovered on *L. barbarum* chromosome 5 (Figure S23B). These genes retained the conserved sequences of the GT domain (Figure S22A and B) while incorporating motifs unique to *L. barbarum*, *e.g.*, Motif 15 and Motif 20, as validated by agarose gel electrophoresis (Figure S24A), indicating a potential functional divergence.

To validate the translation and function of these newly identified *RRT* genes, we constructed both prokaryotic and eukaryotic expression systems of Ly05G003020 (*RRT3020*) (Figure S24B and C). A 45-kDa TrxA-tagged RRT3020 protein was successfully translated in *E. coli* (Figure S24C). In the eukaryotic expression system, we observed that the eGFP-tagged RRT3020 was predominantly localized in the cytoplasm of BY-2 cells (Figure 6B), consistent with its intracellular sublocalization as a rhamnosyl GT. Moreover, we performed whole-mount immunolabeling assays on BY-2 cells expressing RRT3020 as well as BY-2 cells transformed with empty vector (control) using CCRC-M35 (an antibody specifically recognizes RG-I). The results revealed a significant increase of CCRC-M35 signals in RRT3020-expressing cells compared with the control cells (Figure 6C, Figure S24D). Moreover, the protein structure of RRT3020 predicted by AlphaFold2 contained the conserved region of the GT domain (aligned to *A. thaliana* RRT1) (Figure S24E). Taken together, these findings indicate that the expansion of the novel RRT family genes in *L. barbarum* increases the biosynthesis of RG-I pectins.

Another CAZyme gene family expanded in *L. barbarum* and involved in pectin backbone synthesis is GT8 [48], with the expansion focused on galacturonosyltransferase (*GAUT*) genes. We identified 22 *GAUT* genes in *L. barbarum*, a higher number than in *A. thaliana* (15), eggplant (17), and tomato (18) (Figures S25 and S26A). Notably, clustered gene expansions were observed in the *L. barbarum* members of the GAUT-C clade (*AtGAUT12–15*). Within this clade, *AtGAUT13* and *AtGAUT14* are likely related to RG-I biosynthesis [49,50], whereas *AtGAUT12* is involved in the biosynthesis of RG-I, glucuronoxylan, and pectic HG [49,51].

The CE13 family, encoding PAEs, also exhibited significant expansion in *L. barbarum.* Phylogenetic analysis of CE13 genes revealed a clade present in Solanaceae plants but absent in *A. thaliana* (Figure S27A and B). This clade included 8 genes from eggplant, 8 genes from tomato, and 18 genes from *L. barbarum*, showing high similarity to *A. thaliana* genes *AtPAE7*, *AtPAE8*, and *AtPAE11*, which play important roles in plant growth and development [40]. AtPAE8, in particular, preferentially modulates RG-I [52]. Furthermore, four tandemly duplicated CE13 gene clusters were identified in *L. barbarum* (Figures S26B, S27C and D), suggesting a strong functional expansion of this gene family.

### A model for pectin biosynthesis and sugar transport in *L. barbarum*

**Figure 7:**
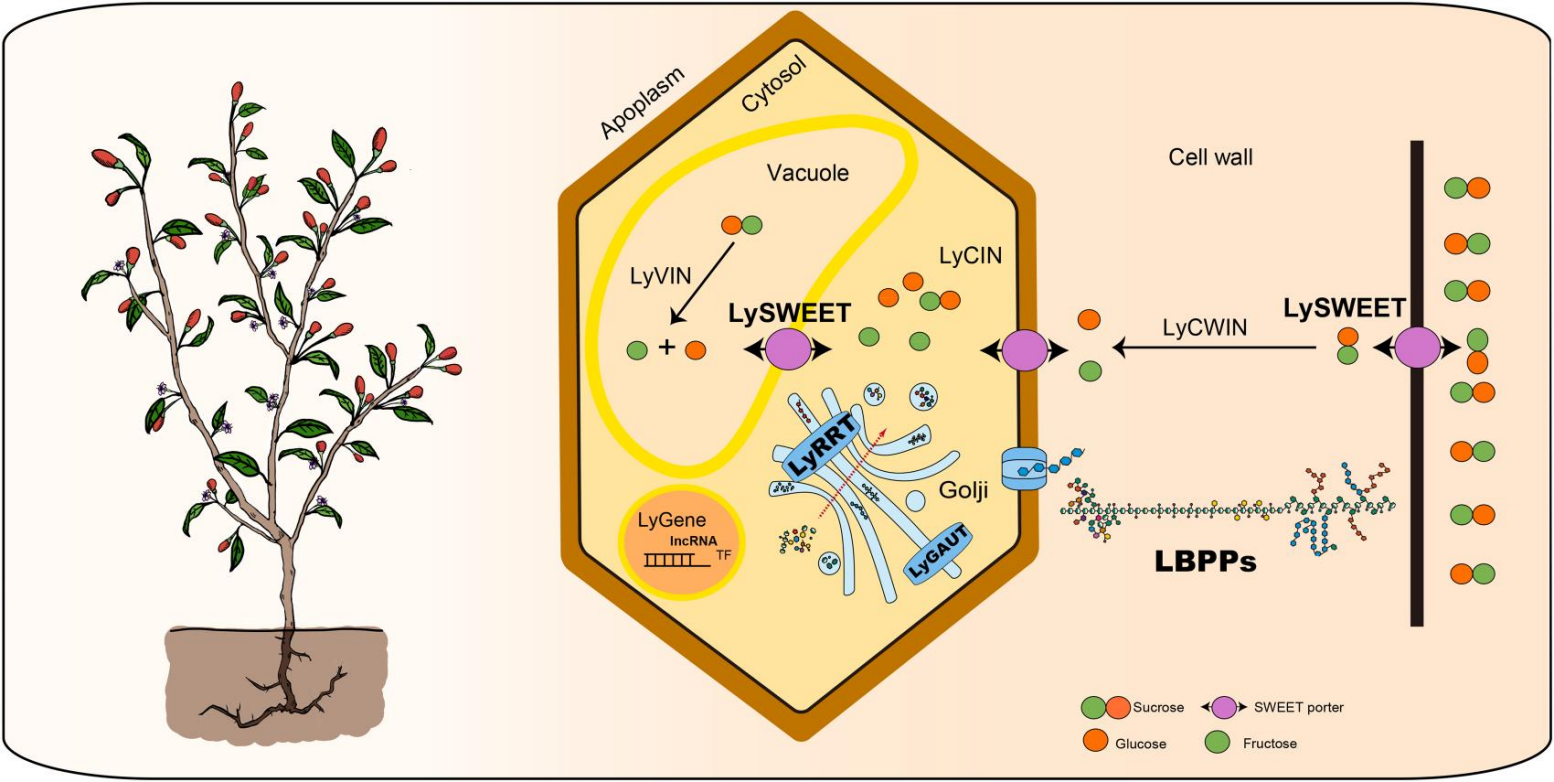
Schematic illustration of pectin biosynthesis and sugar transport in *L. barbarum* VIN, vacuolar invertase; CIN, cytoplasmic invertase; CWIN, cell wall invertase; LBPP, *Lycium barbarum* pectin polysaccharide.

In summary, we propose a model for LBPP biosynthesis and sugar transport during *L. barbarum* development (**Figure 7**). Sucrose is transported into the cell wall, cytosol, and vacuole by LySWEET sugar transporters. Subsequently, enzymes such as LyCWIN, LyVIN, and LyCIN hydrolyze sucrose into fructose and glucose, which serve as the building blocks for pectin biosynthesis. LyRRTs for RG-I and LyGAUTs, localized to the Golgi apparatus, are principally responsible for extending the RG-I backbone. The biosynthesis of LBPPs occurs alongside the incorporation of enzymes that facilitate sidechain modifications. The newly expanded RRTs enable *L. barbarum* to produce special pectins different from other species. All polysaccharide metabolism-related genes, including the lncRNAs and expanded TF genes, are transcribed in the nucleus.

## Discussion

Our comparative analyses provide robust evidence for the role of sugar transport in LBPP biosynthesis. Phylogenetic analysis and expression profiling revealed elevated levels of CAZymes involved in LBPP biosynthesis, with expanded *LyRRT* and *LyGAUT* genes primarily responsible for the extension of the pectic RG-I backbone (Figure 4A). Notably, *L. barbarum*-specific motifs integrated into the GT domain have resulted in novel RRTs, significantly enhancing RG-I synthesis (Figure 6). Additionally, key enzymes involved in arabinan (LyARADs, GT77) and galactan (LyGALTs, GT31) sidechain synthesis are highly expressed in *L. barbarum*, contributing to the structural diversity and abundance of RG-I backbones and sidechains that underpin the biomedical properties of LBPPs.

Pectin biosynthesis involves further modifications of glycosyl residues, such as methyltransferase-catalyzed esterification, *O*-methylation, and acetyltransferase-catalyzed acetylation [42], with the degree of acetylation being regulated by PAEs [40]. Notably, the PAE (CE13) gene family shows significant expansion and high expression in *L. barbarum* but is absent in *A. thaliana* (Figures S26 and S27). These modifications confer *L. barbarum* LBPPs with a broad spectrum of derivatives, contributing to their biomedical potential.

The biomedical properties of pectins, particularly in cancer therapy and drug delivery, are gaining increasing attention [11,53–57]. The current study provides the first comprehensive illustration of the pectin biosynthesis pathway in *L. barbarum* which is supported by genomic and transcriptomic data with experimental verification (Figures 4 and 6). We identified key enzymes involved in pectin backbone elongation, modification, and sidechain synthesis. Moreover, this study is the first to identify polysaccharide metabolism-related lncRNAs in Solanaceae, which have been highly conserved since the WGT event and exhibit high tissue specificity. Varied *GT* and carbohydrate esterase (*CE*) genes may contribute to LBPP biosynthesis and diversification.

The newly identified *RRT* genes and other pivotal genes have enabled *L. barbarum* to synthesize unique pectins with broad applications in food and medicine, distinguishing *L. barbarum* from other species. Combining these with sugar transporters, we propose, for the first time, a model for pectin biosynthesis and sugar transport during *L. barbarum* development (Figure 7). Our findings, together with the reference genome, provide novel insights into the genomic landscape and diversity of *L. barbarum* as well as its various applications as a medicinal and food crop.

## Materials and methods

### Plant materials and genome sequencing

Plant samples were collected from Ningxia Hui Autonomous Region, China. Total DNA was extracted from fresh leaves for genome sequencing. For the genome survey analysis, two paired-end Illumina DNA libraries were generated, producing 268.01 Gb of raw data on the HiSeq X Ten platform. For 10X Genomics sequencing, high-molecular-weight (HMW) genomic DNA was extracted and used to constructed 10X Genomics libraries following the manufacturer’s instructions (Catalog No. PN-120229, 10X Genomics, Pleasanton, CA). Additionally, PacBio SMRTbell libraries with 20 kb inserts were generated, yielding 220.01 Gb of raw data on the PacBio Sequel platform.

### Transcriptome library construction and sequencing

Total RNA was extracted from fresh *L. barbarum* tissues, including roots, stems, leaves, flowers, green fruits, and red fruits. Three independent biological replicates were collected for each tissue type. The Ribo-off rRNA depletion module was employed to enrich RNA, particularly non-coding RNA. The high-quality RNA was then used for library construction, followed by sequencing on the Illumina HiSeq X Ten platform.

To sequence the full-length transcriptome, high-quality RNA was prepared to construct 0−5 kb and 4.5−5 kb SMRTbell libraries following the PacBio Iso-Seq protocol. Long-read sequencing was performed on the PacBio Sequel II system, generating 79.31 Gb of raw data (1.46 million reads) from four SMRT cells.

### BioNano optical maps and hybrid assembly

Tender leaves of *L. barbarum* were protected from light for three days to reduce chloroplast and mitochondrial DNA interference. The label density of Nt.BssSI (a nicking endonuclease) was predicted using a label density calculator based on the preliminary genome assembly. HMW genomic DNA was fluorescently labeled with Nt.BssSI and subsequently loaded onto Saphyr chips for scanning using the BioNano Genomics Saphyr System. A total of 231.65 Gb of BioNano data (average N50: 391.9 kb) were generated, which were then assembled into optical consensus maps using IryView software with default parameters.

### Hi-C library construction and data processing

Hi-C libraries were constructed by combining an in-house protocol [58] with the methodology described by Liu [59]. In brief, fresh young leaves were fixed in 1% (v/v) formaldehyde before the nuclei were isolated. Crosslinked DNA was enzymatically cleaved using MboI, and the resulting sticky ends were filled with biotin-14-dCTP (Catalog No. 19518018, Invitrogen, Carlsbad, CA) to create blunt ends. These ends were ligated, forming chimeric junctions from proximity fragments. The purified DNA was subsequently sheared to a size range of 200–500 bp using a Covaris S2 system. Biotin-labeled chimeric fragments were then captured using streptavidin C1 dynabeads, followed by the construction of two Hi-C libraries.

Two Hi-C libraries were sequenced on the Illumina HiSeq platform, yielding 240 Gb of data (400 million reads). The paired-end reads of the chimeric fragments were aligned to the draft-assembled contigs using Juicer (v1.5.6). After obtaining the “merged_nodups” file, the contigs were clustered into chromosomes and scaffolded using 3D-DNA (v180922).

### Genome size estimation

To identify the characteristics of the *L. barbarum* genome, we employed *k*-mer distribution analysis to estimate genome size and heterozygosity. Briefly, *K*-base sequences were iteratively selected from a continuous sequence, whereby one read of length *L* generated *L*−*K*+1 *k*-mers. The total numbers of bases and *k*-mers were defined as *n_base_* and *n_k-mer_*, respectively. The coverage depths for the bases and *k*-mers were defined as *c_base_* and *c_k-mer_*, respectively. We calculated the frequency and genome size of each 17-mer from the Illumina pair-end reads using the following formulas: *c_base_=c_k-mer_×L/*(*L*−*K+*1), *G=n_k-mer_/c_k-mer_=n_base_/c_base_* [60]. The peak frequency curve was used to represent the overall sequencing depth. The genome size was determined by dividing the total number of *k*-mers by the peak value of the *k*-mer distribution using Jellyfish (v2.3.0) [61].

### Genome assembly

We obtained 220.1 Gb of raw data on the PacBio Sequel platform, with an N50 of ∼ 21.55 kb. The raw reads were first corrected and assembled using CANU (v1.53) (parameters: -correct, saveOverlaps = true, minMemory = 50G, batMemory = 200G, and genomeSize = 5g) to generate contig sequences. The contig-level assembly data were combined with the 10X Genomics data using ARCS (v1.0.1) and LINKS (v1.8.5). Following this step, the contigs were further corrected, and hybrid scaffolds were constructed using BioNano data. The Hi-C data were processed using Juicer, and the chromosome assembly was finalized using 3D-DNA. To improve the assembly’s accuracy, Illumina paired-end reads and Pilon (v1.22) were used to correct missing bases and mutations. Additionally, the genome assembly was aligned to that of Cao et al. [11] using LASTZ (v1.04) on a per-chromosome basis (parameters: E = 150, H = 0, K = 4500, L = 3000, M = 254, O = 600, T = 2, and Y = 15000). Aligned sequence blocks longer than 2500 bp were visualized using the circlize (v0.4.15) package.

Using the assembled genome and Merqury (v1.3), we determined that the optimal *k*-mer length was 21. Subsequently, we utilized Meryl (v1.4.1) to extract 21-mers from the next-generation sequencing data, enabling the calculation of the quality value using Merqury. To calculate the LTR assembly index (LAI), the genome was indexed using the gt suffixerator tool from GenomeTools (v1.6.5). LTR retrotransposons were detected by combining the results from gt ltrharvest and LTR_FINDER_parallel (v1.2), which were further refined and filtered using LTR_retriever. The LAI was then calculated to assess genome assembly quality and completeness, following the recommended LTR_retriever parameters. Additionally, the genome quality was evaluated using Benchmarking Universal Single-Copy Orthologs (BUSCO; embryophyta_odb10).

### Repeat element annotation

A total of 1,479,425,826 bp of repetitive sequences were identified, accounting for 67.75% of the *L. barbarum* genome. Repeat sequences, including transposable elements (TEs), were annotated by combining *de novo* prediction and homology-based prediction. We first created a *de novo* repetitive sequence database using LTR_FINDER (v1.06) and RepeatModeler (http://www.repeatmasker.org/RepeatModeler). Subsequently, the full-length LTR retrotransposons detected by LTR_FINDER were analyzed using RepeatModeler, which employs three *de novo* repeat-finding programs (RECON, RepeatScout, and LTRharvest/LTR_retriever) to identify repeat element boundaries and family relationships. After clustering the redundant results and classifying the families, a high-quality library of TE families was generated for analysis with RepeatMasker (v4.0.7). For homology-based prediction, similar sequences were identified using RepeatMasker (v4.0.7) by aligning against RepBase (v21.12) (http://www.girinst.org/repbase) at the DNA level. RepeatProteinMask (v4.0.7) was used to further confirm the TE-related proteins. In addition, tandem repeats were identified using Tandem Repeats Finder (v4.09) (http://tandem.bu.edu/trf/trf.html) with default parameters.

### Gene structure and function annotation

The genes in the *L. barbarum* genome were annotated by combining transcriptome-based prediction, homology-based prediction, and *de novo* prediction. For homology-based prediction, we aligned the protein sequences of five closely related plants to the *L. barbarum* genome using GeneWise to produce accurate spliced alignments, which were combined with RNA-seq and Iso-Seq reads to create a marker set. We used Augustus (http://bioinf.uni-greifswald.de/augustus/) to generate a *de novo* gene annotation set. We then integrated the marker and *de novo* sets based on the principle that evidence-based results are superior to predicted results. Proteins that consisted of > 50% repeat sequences and proteins that were < 100 amino acids in length were filtered out. We also screened the gene set based on gene expression levels, Iso-Seq support, and supporting evidence in closely related species. Finally, the annotated gene set of *L. barbarum* was obtained.

To infer gene functions, orthologous genes were identified based on sequence similarity, and the functions of newly identified genes were predicted using proteins with defined functions. Functional annotation was performed using the Kyoto Encyclopedia of Genes and Genomes (KEGG), GO, Clusters of Orthologous Groups (COG), and eggNOG5.0 databases. Out of the 31,911 identified protein-coding genes, 30,894 (96.81%) were annotated. Finally, a BLAST (v2.11.0) search against the NR database (E-value < 1E−5; sequence identity > 75%) was performed to annotate the identified protein sequences.

### Transcriptome assembly

Trimmomatic (v0.32) was used to remove adaptor and low-quality reads from the Illumina RNA-seq data. The filtered reads were aligned to the genome using HISAT2. StringTie (v2.1.3) was then used for transcriptome assembly with the default parameters. Gene expression levels were calculated using Cufflinks (v2.2.1).

For PacBio Iso-Seq reads, the standard IsoSeq3 pipeline (https://github.com/PacificBiosciences/IsoSeq3) was applied to obtain high-confidence transcriptomic reads via circular consensus sequencing. The resulting full-length transcripts were annotated using MAKER.

### Transcriptomic analysis

RNA-seq raw reads were processed using Trim Galore (v0.6.4) and Cutadapt (v2.10) [62], followed by quantification in transcripts per million (TPM) using Kallisto (v0.46.1) [63]. DEGs were identified using the edgeR package (v3.30.3) [64] by applying exact tests to normalized gene counts. Genes in each tissue type with false discovery rate (FDR) < 0.05 and fold change > 1.5 or < −1.5 compared to the average level in all samples were classified as DEGs. The enriched GO terms and KEGG pathways were determined via hypergeometric tests using the clusterProfiler package (v3.16.1) [65].

A WGCNA was performed following the method described by Langfelder and Horvath [35]. The gene expression matrix of all identified CAZyme genes was used as the input. The network edge weights were defined as coexpression similarities, measured by expression correlation coefficients raised to a power of 10. Subsets of the network were then visualized using Cytoscape (https://cytoscape.org/).

### Gene family identification and phylogenomic tree construction

For gene family clustering, we analyzed the protein-coding genes from *L. barbarum* and 11 other species: *S. lycopersicum* (ITAG3.2), *S. tuberosum* (v4.03), *Petunia axillaris* (v1.6.2), *S. melongena* (v3.0), *N. sylvestris* (GCF_000393655.1), *Antirrhinum majus* (IGDBv3), *Prunus mume* (v1.0), *A. thaliana* (Araport11), *Vitis vinifera* (v2.1), *Carica papaya* (ASGPBv0.4), and *Oryza sativa* (v7.0). BLASTP was used to perform an all-against-all comparison with default parameters, and OrthoMCL was used to identify the paralogous and orthologous clusters.

A total of 787 single-copy gene families were selected to construct the phylogenetic tree. MUSCLE was used to align the protein sequences from these single-copy gene families, and PhyML was used to construct the phylogenetic tree. Additionally, KaKs_Calculator (v3.0) was used to calculate synonymous (*Ks*) and non-synonymous (*Ka*) substitution rates to infer the WGT events.

### Protein and RNA family annotation

For protein family annotation, the HMM profiles of protein families were downloaded from the Pfam database (http://pfam.xfam.org/) and the *L. barbarum* protein sequences were searched using HMMER (v3.3.1) [66]. Aligned sequences (E-value < 1E−5) were annotated with their corresponding protein family names. Similarly, RNA families were downloaded from the Rfam database (http://rfam.xfam.org/), and the corresponding RNA sequences were annotated using cmscan (v1.1.4) [67].

### Protein structure prediction and visualization

The structures of proteins associated with the *L. barbarum RRT* genes were predicted using AlphaFold (v2.2.0) [68]. While the default parameters were used, the postprediction relaxation procedure was not included. Only the top-ranked result for each protein was retained for subsequent analysis. The predicted structures were aligned and visualized using PyMOL (v2.5.0) (https://github.com/schrodinger/pymol-open-source/).

### Polysaccharide pathway profiling

To identify proteins containing the signature domains of CAZy families, we utilized the *L. barbarum* protein sequences, as well as those of *A. thaliana* (Araport11), *S. lycopersicum* (SL3.0), and *S. melongena* (Eggplant v3), to search against the dbCAN database (HMMdb v9) [34] using HMMER. Candidate proteins were filtered using the recommended dbCAN parameters. Next, we constructed a phylogenetic tree for each identified CAZy family. The sequences were aligned using MAFFT (v7.475) [69], and a maximum-likelihood tree was constructed for each family using IQ-TREE (v2.0.7) (ultrafast bootstrap method with 1000 replicates) [70]. To compare our genome assembly with the previously published genome assembly, the individual CAZyme gene sequences were aligned to the published genome using BLASTN (E-value < 1E–5; sequence identity > 90%).

### lncRNA identification

Novel transcripts were assembled from the mapped reads using StringTie (v2.1.3) [71]. CPAT (v3.0.2) [72] and CNCI (v2) [73] were employed to predict the coding potential of the transcripts. CPAT was trained on known coding and non-coding transcripts from *A. thaliana* and *S. lycopersicum*, and transcripts with a coding potential of < 0.4 were considered potential non-coding transcripts. Using the CNCI software, transcripts with a CNCI score of < 0 were considered. Only transcripts identified by both methods were retained. We then filtered out transcripts that were unexpressed, had only a single exon, overlapped with coding genes, or contained coding domains found in the Pfam database. Additionally, WGCNA was performed on the combined expression matrix of CAZyme genes and all lncRNAs to select top non-coding transcripts that were transcriptionally correlated to each CAZyme gene.

### Culture of tobacco BY-2 cells

Suspended tobacco BY-2 cells were cultured in LS medium at 28°C with continuous shaking (120 r/min) in darkness. The cells were diluted (1:50) on a weekly basis.

### Expression of RRT3020 in prokaryotic cells

The full-length coding sequence of *RRT3020* was amplified using gene-specific primers (Table S13) and cloned into the pET32a vector using the ClonExpress Ultra One Step Cloning Kit (Catalog No. C115, Vazyme, Nanjing, China). The recombinant plasmid was then introduced into *E. coli* BL21(DE3) cells, and protein expression was induced by IPTG for up to 16 h at 16°C. Successful protein expression was confirmed via colloidal Coomassie brilliant blue staining following SDS-PAGE.

### Immunofluorescence

The full-length coding sequences of *RRT3020* and *eGFP* were cloned into the pCAMBIA-1300 vector using the ClonExpress Ultra One Step Cloning Kit (Catalog No. C115, Vazyme). The recombinant plasmid was then introduced into *Agrobacterium tumefaciens* strain EHA105. Subsequently, BY-2 cells were subjected to the *A. tumefaciens*-mediated transformation following the Matsuoka and Nakamura’s method.

The transformed BY-2 cells were cultured at 28°C with continuous shaking (150 r/min) in darkness for 36–72 h, stained with 4′,6-diamidino-2-phenylindole (DAPI), and gently transferred to a confocal culture dish. Images were captured using a microscope equipped with a 20× objective lens and a confocal laser scanning system (LSM700, Carl Zeiss, Jena, Germany).

### Whole-mount immunolabeling assay of RG-I

The BY-2 cells transformed with the vector carrying *RRT3020* as well as the empty vector (control) were collected and washed twice with phosphate-buffered saline (PBS). They were then blocked with 3% (w/v) nonfat milk powder diluted in PBS for 1 h at room temperature. The blocked cells were incubated with the CCRC-M35 antibody (1:10; Catalog No. AS16 3224, AGRISERA, Norcross, GA) for 2–3 h, followed by incubation with Alexa Fluor 594-tagged donkey anti-mouse immunoglobulin G (1:100; Catalog No. R37115, Thermo Fisher Scientific, Waltham, MA) for 1 h at room temperature in PBS containing 3% nonfat milk. The cells were washed with PBS and counterstained with Calcofluor white (405 nm excitation) for 15 min. Fluorescence images were captured using a microscope equipped with a 20× objective lens and a confocal laser scanning system (LSM700, Carl Zeiss).

### Flavonoid pathway annotation

To identify genes involved in the flavonoid pathway, we retrieved putative genes encoding key enzymes from the UniProt database (https://www.uniprot.org/) based on conserved domain information obtained from the Pfam database. We then constructed a phylogenetic tree for each gene family using the protein sequences from *A. thaliana* and *L. barbarum* that contained the same sets of domains, following the method described in the “Polysaccharide pathway profiling” section. Consequently, we identified *L. barbarum* homologs of key *A. thaliana* flavonoid pathway genes, including *4CL*, *BCH*, *CCD*, *CHI*, *CHS*, *CrtISO*, *DXR*, *DXS*, *HDR*, *HDS*, *MCT*, *MDS*, *NCED*, *PAL*, *PDS*, *PSY*, *VDE*, *ZDS*, *ZEP*, and *Z-ISO*. The candidate homologs were further filtered by conducting a BLASTP search against the NR database based on the following criteria: E-value < 1E−5, sequence identity > 75%, and the first search hits from the Solanaceae family with annotations matching the expected functions.

## Supplementary Material

qzae079_Supplementary_Data

## Data Availability

Raw sequences and annotated assemblies generated in this study have been deposited in the BioProject at the National Genomics Data Center (NGDC), Beijing Institute of Genomics (BIG), Chinese Academy of Sciences (CAS) / China National Center for Bioinformation (CNCB) (BioProject: PRJCA010231), and are publicly accessible at https://ngdc.cncb.ac.cn/bioproject. Additional genome annotation files can be accessed at Zenodo (https://zenodo.org/records/13833627). Genome sequences and RNA-seq data have been deposited in the Genome Sequence Archive [74] at the NGDC, BIG, CAS / CNCB (GSA: CRA007471 and CRA007417, respectively), and are publicly accessible at https://ngdc.cncb.ac.cn/gsa. The genome assembly has been deposited in the Genome Warehouse [75] at the NGDC, BIG, CAS / CNCB (GWH: GWHFFME00000000.1), and is publicly accessible at https://ngdc.cncb.ac.cn/gwh. The assembled genome data and gene annotation data, as well as all *L. barbarum* CAZymes identified in this study and the phylogenetic analysis results of CAZymes, have been also deposited in LyBarBase (http://bioinfo.ibp.ac.cn/Gouji/home.html).

## CRediT author statement

**Haiyan Yue:** Conceptualization, Data curation, Formal analysis, Investigation, Methodology, Project administration, Resources, Validation, Visualization, Writing – original draft, Writing – reviewing & editing. **Yiheng Tang:** Data curation, Formal analysis, Methodology, Resources, Software, Validation, Visualization, Writing – original draft, Writing – reviewing & editing. **Aixuan Li:** Data curation, Formal analysis, Investigation, Methodology, Resources, Validation, Visualization, Writing – original draft, Writing – reviewing & editing. **Lili Zhang:** Formal analysis, Methodology, Resources, Software. **Yiwei Niu:** Formal analysis, Methodology, Resources, Software. **Yiming Zhang:** Data curation, Software, Visualization. **Hao Wang:** Formal analysis, Software. **Jianjun Luo:** Resources, Supervision, Writing – reviewing & editing. **Yi Zhao:** Supervision, Writing – reviewing & editing. **Shunmin He:** Resources, Supervision, Writing – reviewing & editing. **Chang Chen:** Conceptualization, Funding acquisition, Project administration, Resources, Supervision, Writing – reviewing & editing. **Runsheng Chen:** Conceptualization, Funding acquisition, Methodology, Project administration, Resources, Supervision, Writing – reviewing & editing. All authors have read and approved the final manuscript.
