# Supplementary material for: Enzyme Repertoires and Genomic Insights into Lycium barbarum Pectin Polysaccharide Biosynthesis
Source: Genomics Proteomics Bioinformatics. 2024 Nov 4;22(6):qzae079. doi: 10.1093/gpbjnl/qzae079 (PMC12011363; doi:10.1093/gpbjnl/qzae079)
Supplement: qzae079_Supplementary_Data [file qzae079_supplementary_data.zip › Supplementary material captions.docx]

Supplementary material

**Figure S1 The morphology and distribution of *L. barbarum***

Fruits and flowers of *L. barbarum*. A sixty-year-old *L. barbarum* individual was selected for whole-genome sequencing. The distribution of *L. barbarum* is recorded in the Global Biodiversity Information Facility (GBIF, https://www.gbif.org/). Yellow hexagonal markers represent the range where *L. barbarum* has been recorded.

**Figure S2 Estimation of *L. barbarum* genome size by *k*-mer analysis**

The 17-mer depth distribution of *L. barbarum* genome. X-axis shows *k*-mer depth, and Y-axis shows *k*-mer frequency. A total of 122,006,636,450 *k*-mers were identified. The *k*-mer depth is 62. Revised genome size is 1967.85 Mb.

**Figure S3 The genome assembly flow chart of *L. barbarum***

PacBio and Illumina sequencing reads were assembled into scaffolds, which were anchored onto chromosomes through BioNano and Hi-C mapping.

**Figure S4 Genome landscape of *L. barbarum***

**A.** The 12 assembled chromosomes (orange). Sizes shown in Mb. **B.** Gene density (green, density interval of 0-15). **C.** The transposable element distribution in 12 chromosomes (orange). **D.**–**F.** Transposable element densities: gypsy (blue), copia (purple), and TRF (pink, density interval: 0-150). **G.** GC content (black, value range: 0.35-0.4). **H.** Intragenomic synteny blocks plotted in a 200 kb sliding window.

**Figure S5 Comparison of genome assemblies**

**A.** Genome alignments between the LyBar assembly and the Lba assembly (Cao et al.). LyBar is shown in green and Lba is in orange. **B.** GO enrichment analysis was performed on novel CAZyme genes identified in the current LyBar assembly but not found in the previous Lba. **C.** Collinear segments larger than 5 kb are shown between LyBar v1.0 chromosome 4 and Lba chromosome 7. The X-axis shows coordinates in the current LyBar assembly, while the Y-axis shows coordinates in the Lba assembly.

**Figure S6** **Maximum likelihood (ML) phylogenetic tree of candidate AP2 genes identified in *L. barbarum***

AP2 genes of *L. barbarum* were identified by hmmsearch, guided by homologous sequences in *A. thaliana* and protein annotations from the Swiss-Prot protein database. A total of 129 genes were obtained and a phylogenetic tree was constructed, incorporating 145 AP2 genes from *A. thaliana*. In the figure, red labels represent *L. barbarum* genes, black labels represent *A. thaliana* genes, and different colors indicate different AP2 subfamilies, which were classified based on protein annotation, homologous sequence searching in *A. thaliana* and the Swiss-Prot protein database.

**Figure S7 Maximum likelihood (ML) phylogenetic tree of MADS-box genes**

Different colors indicate different MADs-box subfamilies, which were classified based on protein annotation, homologous sequence searches in *A. thaliana*, and the Swiss-Prot protein database. MADs-box genes of *L. barbarum* were identified by BLASTp. A total of 60 genes were obtained with a E-value cutoff of 1E–0.5. A phylogenetic tree was constructed, incorporating 109 MADs-box genes from *A. thaliana*. In the figure, red labels represent *L. barbarum* genes, while black labels represent *A. thaliana* genes.

**Figure S8 Maximum likelihood (ML) phylogenetic tree of RWP-RK genes**

A total of 22 RWP-RK genes of *L. barbarum* were identified by hmmsearch, guided by homologous sequence in *A. thaliana* and protein annotation from the Swiss-Prot protein database. A phylogenetic tree was constructed, incorporating 14 RWP-RK genes in *A. thaliana*, 12 RWP-RK genes in *S. lycopersicum*, and 14 RWP-RK genes in *O. sativa*. In the tree, red labels represent *L. barbarum* genes, black labels represent RWP-RK genes from the other three species. The highlighted parts in the phylogenetic tree in pink and green indicate NLP and RKD subfamilies, respectively.

**Figure S9 The expanded transcription factors of the NF-YA gene family in *L. barbarum***

**A.** Phylogenetic tree of NF-YA genes. A total of 18 NF-YA, 6 NF-YB and NF-YC genes were identified in *L. barbarum* using hmmsearch according to homologous sequences in *A. thaliana* and protein annotations from the Swiss-Prot protein database. **B.** HMMER was used to screen the gene families in *L. barbarum* and *S. lycopersicum*. A total of 18 genes were obtained in *L. barbarum* to construct a phylogenetic tree alongside 10 NF-YA genes from *A. thaliana* and 11 NF-YA genes from *S. lycopersicum*. In the tree, red labels represent *L. barbarum* genes, black labels represent *A. thaliana* and *S. lycopersicum* genes.

**Figure S10 The contracted transcription factors of the NF-YB and NF-YC gene families in *L. barbarum***

**A.** Phylogenetic tree of NF-YB genes. HMMER was used to identify the NF-YB gene family in *L. barbarum* and *S. lycopersicum* according to the NF-YB sequences from *A. thaliana*. A total of 6 NF-YB genes were identified in *L. barbarum* to construct a phylogenetic tree incorporating 13 NF-YB genes in *A. thaliana* and 28 NF-YB genes in *S. lycopersicum*. **B.** Phylogenetic tree of NF-YC genes. A total of 6 NF-YC were identified in *L. barbarum* through the same method as for NF-YB. The phylogenetic tree was constructed with 6 NY-FC genes in *L. barbarum,* 13 NF-YC genes in *A. thaliana*, and 18 NF-YC genes in *S. lycopersicum*.

**Figure S11 Gene family expansion and fruit-specific expression of the SWEET family sugar transporters**

**A.** GO enrichment analysis of gene families expanded in *L. barbarum*. **B.** Expression heatmap of SWEET and invertase genes highly expressed in mature fruit tissues. Yft, yellow fruit; Bft, black fruit; Rft, red fruit; Gft, green fruit. **C.** Validation of top differentially expressed SWEET genes between fruits of the three *L. barbarum* varieties using qRT-PCR, data represents means ± SE (N=3). **D.** Maximum likelihood phylogenetic tree of identified *L. barbarum* SWEET genes. Rft, red fruit; Gft, green fruit; Rt, root; Fr, flower; Sm, stem; Lf, leaf. **E.** Expression heatmap of sugar transporter (STP) genes in mature fruit tissues. Yft, yellow fruit; Bft, black fruit; Rft, red fruit; Gft, green fruit.

**Figure S12 Gene family expansion of *L. barbarum* cell wall and vacuolar invertase genes (CAZy family GH32)**

**A.** Maximum likelihood phylogenetic tree of CAZy family GH32 genes across four species. **B.** Genomic locations of CAZy family GH32 genes in *L. barbarum*. **C.** Genomic locations and the average expression levels of the main tandem duplicate clusters labeled in b (I-IV). Two genes at position III (Ly09G004190 and Ly09G004200) are highly expressed in red fruit.

**Figure S13 Phylogenetic characterization of genes encoding *L. barbarum* CWIN/VIN enzymes**

**A.** Phylogenetic tree of *L. barbarum* CWIN/CIN (CAZy family GH32) genes with log_2_(TPM) expression levels. Bootstrap confidence levels are labeled on nodes. Rft, red fruit; Gft, green fruit; Rt, root; Fr, flower; Sm, stem; Lf, leaf. **B.** Genomic collinearity of CAZy family GH32 invertase genes between *L. barbarum* and tomato.

**Figure S14 Gene family expansion of *L. barbarum* alkaline/neutral invertase genes (CAZy family GH100)**

**A.** Maximum likelihood phylogenetic tree of CAZy family GH100 genes across four species. **B.** Phylogenetic tree of *L. barbarum* GH100 genes with log_2_(TPM) expression levels. Bootstrap confidence levels are labeled on nodes. Rft, red fruit; Gft, green fruit; Rt, root; Fr, flower; Sm, stem; Lf, leaf.

**Figure S15 Tissue-specific expression of genes and functional enrichment, KEGG pathways of expansion gene families in *L. barbarum***

**A.** Venn diagram of differentially expressed genes across five *L. barbarum* tissues compared to the average expression of all samples from each variant. **B.** GO enrichment analysis was performed on genes that are differentially expressed between specific tissues and the *L. barbarum* average. The top enriched terms are shown in the figure. GO terms related to polysaccharide metabolism (red), biosynthesis of pigments (purple), and drought tolerance (orange) are highlighted.

**Figure S16 Differential expression genes (DEGs) across eight sequenced tissues**

**A.** Correlation coefficients of expression profiles across all tissues. Rft, red fruit; Gft, green fruit; Rt, root; Fr, flower; Sm, stem; Lf, leaf. **B.** Venn diagram shows differentially expressed genes in fruit tissues of three different variants compared to *L. barbarum* green fruit. **C.** Heatmap of normalized TPM expression values for differentially expressed genes across all tissues.

**Figure S17 The biosynthesis pathways and tissue-specific gene regulation of carotenoids in *L. barbarum***

Genes involved in carotenoid accumulation were identified through phylogenetic trees and BLASTP under strict parameter screening, the identified genes including DXR, DXS, MCT, MDS, HDR, HDS, PDS, PSY, Z-ISO, ZDS, CrtISO, LCY-B, BCH, NCED, CCD, VED, ZEP, and LCY-E. Gene expressions (normalized to TPM) across different tissues are presented by a heatmap.

**Figure S18 The biosynthesis pathways and tissue-specific genes regulation of anthocyanin in *L. barbarum***

Genes involved in anthocyanin metabolism were identified through phylogenetic trees and BLASTP under strict parameter screening, the identified genes including PAL, C4H, 4CL, CHS, CHI, F3H, F3’H, F3’5’H, DFR, ANS, UF3GT, and MT, among others. Gene expressions (normalized to TPM) across different tissues are presented by a heatmap.

**Figure S19 Phylogenetic characterization of *L. barbarum* glycosyltransferase genes of CAZy family GT77**

**A.** Maximum likelihood phylogenetic tree of CAZy family GT77 genes across four species. **B.** Phylogenetic tree of *L. barbarum* GT77 genes with log_2_(TPM) expression levels. Bootstrap confidence levels are labeled on nodes. Rft, red fruit; Gft, green fruit; Rt, root; Fr, flower; Sm, stem; Lf, leaf.

**Figure S20 Phylogenetic characterization of *L. barbarum* glycosyltransferase genes of CAZy family GT31**

**A.** Maximum likelihood phylogenetic tree of CAZy family GT31 genes across four species. **B.** Phylogenetic tree of *L. barbarum* GT31 genes with log_2_(TPM) expression levels. Bootstrap confidence levels are labeled on nodes. Rft, red fruit; Gft, green fruit; Rt, root; Fr, flower; Sm, stem; Lf, leaf.

**Figure S21 Identification and characterization of *L. barbarum* lncRNAs**

**A.** Venn diagram of differentially expressed lncRNAs in fruit tissues of three different variants compared to *L. barbarum* green fruit. **B.** Ten most correlated lncRNAs of selected RRT genes in the WGCNA network. Red, upregulated in mature (red) fruit; blue, downregulated in mature (red) fruit. **C.** Principal component analysis (PCA) of lncRNA expression profiles for all samples. **D.** PCA of coding gene expression profiles. **E.** Distribution of synonymous substitution rate (*Ks*) of all coding genes. **F.** Distribution of synonymous substitution rate (*Ks*) of all CAZyme genes.

**Figure S22 Sequence features of *L. barbarum* CAZy family GT106 and rhamnosyltransferase (RRT) genes**

**A.** Maximum likelihood phylogenetic tree of CAZy family GT106 genes across four species. **B.** Pfam domains of genes in the clade RRT1-4. Two *L. barbarum* RRT genes contain a partial peptidase C48 domain in addition to the shared O-fucosyltransferase (O-FucT) domain.

**Figure S23 Phylogenetic characterization of *L. barbarum* rhamnosyltransferase (RRT) genes**

**A.** Phylogenetic tree of *L. barbarum* CAZy family GT106 genes with log_2_ (TPM) expression levels. Bootstrap confidence levels are labeled on nodes. Rft, red fruit; Gft, green fruit; Rt, root; Fr, flower; Sm, stem; Lf, leaf. **B.** Genomic synteny of genes in the clade RRT1-4 between *L. barbarum* and tomato.

**Figure S24 Intracellular localization and secondary structure of RRT3020**

**A.** Agarose gel electrophoresis of *L. barbarum*-specific motif sequence. **B.** The vector backbone of pCAMBIA1300 carrying the full-length coding sequences of *RRT3020* and *eGFP*. **C.** SDS-PAGE showing the expression of the TrxA-tagged RRT3020 recombinant protein in *E. coli*. The full-length coding sequence of *RRT3020* was inserted into the pET32a vector and then transformed into *E. coli*. Lanes Empty and Empty IPTG: empty PET-32a vector and total proteins induced by 0.5mM IPTG. Lanes RRT3020 and RRT3020 IPTG: recombinant RRT3020 protein and total proteins induced by 0.5mM IPTG. Arrowheads indicate the recombinant protein. **D.** Whole mount immunolabeling of RG-I with the CCRC-M35 antibody in the control and RRT3020-expressing BY-2 cells at day 25, and cellulosic ray structure with the calcofluor white fluorescent dye. This experiment was repeated three times with similar results. Scale bars = 200μm. **E.** RRT3020 protein structure predicted by Alphafold2 (above) and comparison with AtRRT1 (below).

**Figure S25 Phylogenetic characterization of *L. barbarum* galacturonosyltransferase (GAUT) genes**

**A.** Maximum likelihood phylogenetic tree of GAUT genes across four species, showing the three known evolutionary clades. **B.** Phylogenetic tree of *L. barbarum* CAZy family GT8 genes with log_2_(TPM) expression levels. GAUT and GAUT-like (GATL) genes were annotated based on homology with *A. thaliana* genes. Bootstrap confidence levels are labeled on nodes. Rft, red fruit; Gft, green fruit; Rt, root; Fr, flower; Sm, stem; Lf, leaf.

**Figure S26 Genomic synteny of expanded CAZy families between *L. barbarum* and tomato**

**A.** Genomic collinearity of galacturonosyltransferase (GAUT) genes (CAZy family GT8) between *L. barbarum* and tomato. **B.** Genomic collinearity of pectin acetylesterase genes (CAZy family CE13) between *L. barbarum* and tomato.

**Figure S27 Gene family expansion of *L. barbarum* pectin acetylesterase genes (CAZy family CE13)**

**A.** Maximum likelihood phylogenetic tree of CAZy family CE13 genes across four species. **B.** Phylogenetic tree of *L. barbarum* CE13 genes with log_2_(TPM) expression levels. Bootstrap confidence levels are labeled on nodes. Rft, red fruit; Gft, green fruit; Rt, root; Fr, flower; Sm, stem; Lf, leaf. **C.** Genomic locations of CE13 genes in *L. barbarum*. **D.** Genomic locations and average expressions of the main tandem duplicate clusters labeled in c (I-IV).

**Table S1 The genome survey analysis of *Lycium barbarum***

**Table S2 Summary of sequencing data of *Lycium barbarum***

**Table S3 Assembly features of *Lycium barbarum***

**Table S4 BUSCO benchmarking of *Lycium barbarum* genome assembly and annotations**

**Table S5 Annotation features of *Lycium barbarum***

**Table S6 Repetitive elements (REs) of *Lycium barbarum***

**Table S7 Genomic information of the 12 species used in phylogenetic tree**

**Table S8 Expanded and contracted gene families in KEGG**

**Table S9 Expansion TF/TR in *Lycium barbarum***

**Table S10 Statistics on orthogroups per species**

**Table S11 SWEET genes in *Lycium barbarum***

**Table S12 *Lycium barbarum* genes by CAZy family**

**Table S13 The primers used for PCR, qPCR and homologous recombination**

**Table S14 Percentage of non-coding regions in the *Lycium barbarum* genome**

**[1] Gao Y, Wei Y, Wang Y, Gao F, Chen Z. Lycium Barbarum: A Traditional Chinese Herb and A Promising Anti-Aging Agent. Aging Dis 2017;8:778-91.**

**[1] Gao Y, Wei Y, Wang Y, Gao F, Chen Z. Lycium Barbarum: A Traditional Chinese Herb and A Promising Anti-Aging Agent. Aging Dis 2017;8:778-91.**

**[2] Sun C, Chen X, Yang S, Jin C, Ding K, Chen C. LBP1C-2 from Lycium barbarum alleviated age-related bone loss by targeting BMPRIA/BMPRII/Noggin. Carbohydr Polym 2023;310:120725.**

**[1] Gao Y, Wei Y, Wang Y, Gao F, Chen Z. Lycium Barbarum: A Traditional Chinese Herb and A Promising Anti-Aging Agent. Aging Dis 2017;8:778-91.**

**[2] Sun C, Chen X, Yang S, Jin C, Ding K, Chen C. LBP1C-2 from Lycium barbarum alleviated age-related bone loss by targeting BMPRIA/BMPRII/Noggin. Carbohydr Polym 2023;310:120725.**

**[3] Potterat O. Goji (Lycium barbarum and L. chinense): Phytochemistry, pharmacology and safety in the perspective of traditional uses and recent popularity. Planta Med 2010;76:7-19.**

**[1] Gao Y, Wei Y, Wang Y, Gao F, Chen Z. Lycium Barbarum: A Traditional Chinese Herb and A Promising Anti-Aging Agent. Aging Dis 2017;8:778-91.**

**[2] Sun C, Chen X, Yang S, Jin C, Ding K, Chen C. LBP1C-2 from Lycium barbarum alleviated age-related bone loss by targeting BMPRIA/BMPRII/Noggin. Carbohydr Polym 2023;310:120725.**

**[3] Potterat O. Goji (Lycium barbarum and L. chinense): Phytochemistry, pharmacology and safety in the perspective of traditional uses and recent popularity. Planta Med 2010;76:7-19.**

**[1] Gao Y, Wei Y, Wang Y, Gao F, Chen Z. Lycium Barbarum: A Traditional Chinese Herb and A Promising Anti-Aging Agent. Aging Dis 2017;8:778-91.**

**[2] Sun C, Chen X, Yang S, Jin C, Ding K, Chen C. LBP1C-2 from Lycium barbarum alleviated age-related bone loss by targeting BMPRIA/BMPRII/Noggin. Carbohydr Polym 2023;310:120725.**

**[3] Potterat O. Goji (Lycium barbarum and L. chinense): Phytochemistry, pharmacology and safety in the perspective of traditional uses and recent popularity. Planta Med 2010;76:7-19.**

**[4] Zhang XJ, Yu HY, Cai YJ, Ke M. Lycium barbarum polysaccharides inhibit proliferation and migration of bladder cancer cell lines BIU87 by suppressing Pi3K/AKT pathway. Oncotarget 2017;8:5936-42.**

**[1] Gao Y, Wei Y, Wang Y, Gao F, Chen Z. Lycium Barbarum: A Traditional Chinese Herb and A Promising Anti-Aging Agent. Aging Dis 2017;8:778-91.**

**[2] Sun C, Chen X, Yang S, Jin C, Ding K, Chen C. LBP1C-2 from Lycium barbarum alleviated age-related bone loss by targeting BMPRIA/BMPRII/Noggin. Carbohydr Polym 2023;310:120725.**

**[3] Potterat O. Goji (Lycium barbarum and L. chinense): Phytochemistry, pharmacology and safety in the perspective of traditional uses and recent popularity. Planta Med 2010;76:7-19.**

**[4] Zhang XJ, Yu HY, Cai YJ, Ke M. Lycium barbarum polysaccharides inhibit proliferation and migration of bladder cancer cell lines BIU87 by suppressing Pi3K/AKT pathway. Oncotarget 2017;8:5936-42.**

**[5] Tang L, Bao S, Du Y, Jiang Z, Wuliji AO, Ren X, et al. Antioxidant effects of Lycium barbarum polysaccharides on photoreceptor degeneration in the light-exposed mouse retina. Biomed Pharmacother 2018;103:829-37.**

**[1] Gao Y, Wei Y, Wang Y, Gao F, Chen Z. Lycium Barbarum: A Traditional Chinese Herb and A Promising Anti-Aging Agent. Aging Dis 2017;8:778-91.**

**[2] Sun C, Chen X, Yang S, Jin C, Ding K, Chen C. LBP1C-2 from Lycium barbarum alleviated age-related bone loss by targeting BMPRIA/BMPRII/Noggin. Carbohydr Polym 2023;310:120725.**

**[3] Potterat O. Goji (Lycium barbarum and L. chinense): Phytochemistry, pharmacology and safety in the perspective of traditional uses and recent popularity. Planta Med 2010;76:7-19.**

**[4] Zhang XJ, Yu HY, Cai YJ, Ke M. Lycium barbarum polysaccharides inhibit proliferation and migration of bladder cancer cell lines BIU87 by suppressing Pi3K/AKT pathway. Oncotarget 2017;8:5936-42.**

**[5] Tang L, Bao S, Du Y, Jiang Z, Wuliji AO, Ren X, et al. Antioxidant effects of Lycium barbarum polysaccharides on photoreceptor degeneration in the light-exposed mouse retina. Biomed Pharmacother 2018;103:829-37.**

**[6] Zhang W, Zhang J, Ding D, Zhang L, Muehlmann LA, Deng SE, et al. Synthesis and antioxidant properties of Lycium barbarum polysaccharides capped selenium nanoparticles using tea extract. Artif Cells Nanomed Biotechnol 2018;46:1463-70.**

**[1] Gao Y, Wei Y, Wang Y, Gao F, Chen Z. Lycium Barbarum: A Traditional Chinese Herb and A Promising Anti-Aging Agent. Aging Dis 2017;8:778-91.**

**[2] Sun C, Chen X, Yang S, Jin C, Ding K, Chen C. LBP1C-2 from Lycium barbarum alleviated age-related bone loss by targeting BMPRIA/BMPRII/Noggin. Carbohydr Polym 2023;310:120725.**

**[3] Potterat O. Goji (Lycium barbarum and L. chinense): Phytochemistry, pharmacology and safety in the perspective of traditional uses and recent popularity. Planta Med 2010;76:7-19.**

**[4] Zhang XJ, Yu HY, Cai YJ, Ke M. Lycium barbarum polysaccharides inhibit proliferation and migration of bladder cancer cell lines BIU87 by suppressing Pi3K/AKT pathway. Oncotarget 2017;8:5936-42.**

**[5] Tang L, Bao S, Du Y, Jiang Z, Wuliji AO, Ren X, et al. Antioxidant effects of Lycium barbarum polysaccharides on photoreceptor degeneration in the light-exposed mouse retina. Biomed Pharmacother 2018;103:829-37.**

**[6] Zhang W, Zhang J, Ding D, Zhang L, Muehlmann LA, Deng SE, et al. Synthesis and antioxidant properties of Lycium barbarum polysaccharides capped selenium nanoparticles using tea extract. Artif Cells Nanomed Biotechnol 2018;46:1463-70.**

**[7] Ma K, Wang X, Feng S, Xia X, Zhang H, Rahaman A, et al. From the perspective of Traditional Chinese Medicine: Treatment of mental disorders in COVID-19 survivors. Biomed Pharmacother 2020;132:110810.**

**[1] Gao Y, Wei Y, Wang Y, Gao F, Chen Z. Lycium Barbarum: A Traditional Chinese Herb and A Promising Anti-Aging Agent. Aging Dis 2017;8:778-91.**

**[2] Sun C, Chen X, Yang S, Jin C, Ding K, Chen C. LBP1C-2 from Lycium barbarum alleviated age-related bone loss by targeting BMPRIA/BMPRII/Noggin. Carbohydr Polym 2023;310:120725.**

**[3] Potterat O. Goji (Lycium barbarum and L. chinense): Phytochemistry, pharmacology and safety in the perspective of traditional uses and recent popularity. Planta Med 2010;76:7-19.**

**[4] Zhang XJ, Yu HY, Cai YJ, Ke M. Lycium barbarum polysaccharides inhibit proliferation and migration of bladder cancer cell lines BIU87 by suppressing Pi3K/AKT pathway. Oncotarget 2017;8:5936-42.**

**[5] Tang L, Bao S, Du Y, Jiang Z, Wuliji AO, Ren X, et al. Antioxidant effects of Lycium barbarum polysaccharides on photoreceptor degeneration in the light-exposed mouse retina. Biomed Pharmacother 2018;103:829-37.**

**[6] Zhang W, Zhang J, Ding D, Zhang L, Muehlmann LA, Deng SE, et al. Synthesis and antioxidant properties of Lycium barbarum polysaccharides capped selenium nanoparticles using tea extract. Artif Cells Nanomed Biotechnol 2018;46:1463-70.**

**[7] Ma K, Wang X, Feng S, Xia X, Zhang H, Rahaman A, et al. From the perspective of Traditional Chinese Medicine: Treatment of mental disorders in COVID-19 survivors. Biomed Pharmacother 2020;132:110810.**

**[8] Po KK, Leung JW, Chan JN, Fung TK, Sánchez-Vidaña DI, Sin EL, et al. Protective effect of Lycium Barbarum polysaccharides on dextromethorphan-induced mood impairment and neurogenesis suppression. Brain Res Bull 2017;134:10-7.**

**[1] Gao Y, Wei Y, Wang Y, Gao F, Chen Z. Lycium Barbarum: A Traditional Chinese Herb and A Promising Anti-Aging Agent. Aging Dis 2017;8:778-91.**

**[2] Sun C, Chen X, Yang S, Jin C, Ding K, Chen C. LBP1C-2 from Lycium barbarum alleviated age-related bone loss by targeting BMPRIA/BMPRII/Noggin. Carbohydr Polym 2023;310:120725.**

**[3] Potterat O. Goji (Lycium barbarum and L. chinense): Phytochemistry, pharmacology and safety in the perspective of traditional uses and recent popularity. Planta Med 2010;76:7-19.**

**[4] Zhang XJ, Yu HY, Cai YJ, Ke M. Lycium barbarum polysaccharides inhibit proliferation and migration of bladder cancer cell lines BIU87 by suppressing Pi3K/AKT pathway. Oncotarget 2017;8:5936-42.**

**[5] Tang L, Bao S, Du Y, Jiang Z, Wuliji AO, Ren X, et al. Antioxidant effects of Lycium barbarum polysaccharides on photoreceptor degeneration in the light-exposed mouse retina. Biomed Pharmacother 2018;103:829-37.**

**[6] Zhang W, Zhang J, Ding D, Zhang L, Muehlmann LA, Deng SE, et al. Synthesis and antioxidant properties of Lycium barbarum polysaccharides capped selenium nanoparticles using tea extract. Artif Cells Nanomed Biotechnol 2018;46:1463-70.**

**[7] Ma K, Wang X, Feng S, Xia X, Zhang H, Rahaman A, et al. From the perspective of Traditional Chinese Medicine: Treatment of mental disorders in COVID-19 survivors. Biomed Pharmacother 2020;132:110810.**

**[8] Po KK, Leung JW, Chan JN, Fung TK, Sánchez-Vidaña DI, Sin EL, et al. Protective effect of Lycium Barbarum polysaccharides on dextromethorphan-induced mood impairment and neurogenesis suppression. Brain Res Bull 2017;134:10-7.**

**[9] Fang S, Dong L, Liu L, Guo J, Zhao L, Zhang J, et al. HERB: a high-throughput experiment- and reference-guided database of traditional Chinese medicine. Nucleic Acids Res 2021;49:D1197-d206.**

**[1] Gao Y, Wei Y, Wang Y, Gao F, Chen Z. Lycium Barbarum: A Traditional Chinese Herb and A Promising Anti-Aging Agent. Aging Dis 2017;8:778-91.**

**[2] Sun C, Chen X, Yang S, Jin C, Ding K, Chen C. LBP1C-2 from Lycium barbarum alleviated age-related bone loss by targeting BMPRIA/BMPRII/Noggin. Carbohydr Polym 2023;310:120725.**

**[3] Potterat O. Goji (Lycium barbarum and L. chinense): Phytochemistry, pharmacology and safety in the perspective of traditional uses and recent popularity. Planta Med 2010;76:7-19.**

**[4] Zhang XJ, Yu HY, Cai YJ, Ke M. Lycium barbarum polysaccharides inhibit proliferation and migration of bladder cancer cell lines BIU87 by suppressing Pi3K/AKT pathway. Oncotarget 2017;8:5936-42.**

**[5] Tang L, Bao S, Du Y, Jiang Z, Wuliji AO, Ren X, et al. Antioxidant effects of Lycium barbarum polysaccharides on photoreceptor degeneration in the light-exposed mouse retina. Biomed Pharmacother 2018;103:829-37.**

**[6] Zhang W, Zhang J, Ding D, Zhang L, Muehlmann LA, Deng SE, et al. Synthesis and antioxidant properties of Lycium barbarum polysaccharides capped selenium nanoparticles using tea extract. Artif Cells Nanomed Biotechnol 2018;46:1463-70.**

**[7] Ma K, Wang X, Feng S, Xia X, Zhang H, Rahaman A, et al. From the perspective of Traditional Chinese Medicine: Treatment of mental disorders in COVID-19 survivors. Biomed Pharmacother 2020;132:110810.**

**[8] Po KK, Leung JW, Chan JN, Fung TK, Sánchez-Vidaña DI, Sin EL, et al. Protective effect of Lycium Barbarum polysaccharides on dextromethorphan-induced mood impairment and neurogenesis suppression. Brain Res Bull 2017;134:10-7.**

**[9] Fang S, Dong L, Liu L, Guo J, Zhao L, Zhang J, et al. HERB: a high-throughput experiment- and reference-guided database of traditional Chinese medicine. Nucleic Acids Res 2021;49:D1197-d206.**

**[10] Zhang S, He F, Chen X, Ding K. Isolation and structural characterization of a pectin from Lycium ruthenicum Murr and its anti-pancreatic ductal adenocarcinoma cell activity. Carbohydr Polym 2019;223:115104.**

**[1] Gao Y, Wei Y, Wang Y, Gao F, Chen Z. Lycium Barbarum: A Traditional Chinese Herb and A Promising Anti-Aging Agent. Aging Dis 2017;8:778-91.**

**[2] Sun C, Chen X, Yang S, Jin C, Ding K, Chen C. LBP1C-2 from Lycium barbarum alleviated age-related bone loss by targeting BMPRIA/BMPRII/Noggin. Carbohydr Polym 2023;310:120725.**

**[3] Potterat O. Goji (Lycium barbarum and L. chinense): Phytochemistry, pharmacology and safety in the perspective of traditional uses and recent popularity. Planta Med 2010;76:7-19.**

**[4] Zhang XJ, Yu HY, Cai YJ, Ke M. Lycium barbarum polysaccharides inhibit proliferation and migration of bladder cancer cell lines BIU87 by suppressing Pi3K/AKT pathway. Oncotarget 2017;8:5936-42.**

**[5] Tang L, Bao S, Du Y, Jiang Z, Wuliji AO, Ren X, et al. Antioxidant effects of Lycium barbarum polysaccharides on photoreceptor degeneration in the light-exposed mouse retina. Biomed Pharmacother 2018;103:829-37.**

**[6] Zhang W, Zhang J, Ding D, Zhang L, Muehlmann LA, Deng SE, et al. Synthesis and antioxidant properties of Lycium barbarum polysaccharides capped selenium nanoparticles using tea extract. Artif Cells Nanomed Biotechnol 2018;46:1463-70.**

**[7] Ma K, Wang X, Feng S, Xia X, Zhang H, Rahaman A, et al. From the perspective of Traditional Chinese Medicine: Treatment of mental disorders in COVID-19 survivors. Biomed Pharmacother 2020;132:110810.**

**[8] Po KK, Leung JW, Chan JN, Fung TK, Sánchez-Vidaña DI, Sin EL, et al. Protective effect of Lycium Barbarum polysaccharides on dextromethorphan-induced mood impairment and neurogenesis suppression. Brain Res Bull 2017;134:10-7.**

**[9] Fang S, Dong L, Liu L, Guo J, Zhao L, Zhang J, et al. HERB: a high-throughput experiment- and reference-guided database of traditional Chinese medicine. Nucleic Acids Res 2021;49:D1197-d206.**

**[10] Zhang S, He F, Chen X, Ding K. Isolation and structural characterization of a pectin from Lycium ruthenicum Murr and its anti-pancreatic ductal adenocarcinoma cell activity. Carbohydr Polym 2019;223:115104.**

**[11] Cao YL, Li YL, Fan YF, Li Z, Yoshida K, Wang JY, et al. Wolfberry genomes and the evolution of Lycium (Solanaceae). Commun Biol 2021;4:671.**

**[1] Gao Y, Wei Y, Wang Y, Gao F, Chen Z. Lycium Barbarum: A Traditional Chinese Herb and A Promising Anti-Aging Agent. Aging Dis 2017;8:778-91.**

**[2] Sun C, Chen X, Yang S, Jin C, Ding K, Chen C. LBP1C-2 from Lycium barbarum alleviated age-related bone loss by targeting BMPRIA/BMPRII/Noggin. Carbohydr Polym 2023;310:120725.**

**[3] Potterat O. Goji (Lycium barbarum and L. chinense): Phytochemistry, pharmacology and safety in the perspective of traditional uses and recent popularity. Planta Med 2010;76:7-19.**

**[4] Zhang XJ, Yu HY, Cai YJ, Ke M. Lycium barbarum polysaccharides inhibit proliferation and migration of bladder cancer cell lines BIU87 by suppressing Pi3K/AKT pathway. Oncotarget 2017;8:5936-42.**

**[5] Tang L, Bao S, Du Y, Jiang Z, Wuliji AO, Ren X, et al. Antioxidant effects of Lycium barbarum polysaccharides on photoreceptor degeneration in the light-exposed mouse retina. Biomed Pharmacother 2018;103:829-37.**

**[6] Zhang W, Zhang J, Ding D, Zhang L, Muehlmann LA, Deng SE, et al. Synthesis and antioxidant properties of Lycium barbarum polysaccharides capped selenium nanoparticles using tea extract. Artif Cells Nanomed Biotechnol 2018;46:1463-70.**

**[7] Ma K, Wang X, Feng S, Xia X, Zhang H, Rahaman A, et al. From the perspective of Traditional Chinese Medicine: Treatment of mental disorders in COVID-19 survivors. Biomed Pharmacother 2020;132:110810.**

**[8] Po KK, Leung JW, Chan JN, Fung TK, Sánchez-Vidaña DI, Sin EL, et al. Protective effect of Lycium Barbarum polysaccharides on dextromethorphan-induced mood impairment and neurogenesis suppression. Brain Res Bull 2017;134:10-7.**

**[9] Fang S, Dong L, Liu L, Guo J, Zhao L, Zhang J, et al. HERB: a high-throughput experiment- and reference-guided database of traditional Chinese medicine. Nucleic Acids Res 2021;49:D1197-d206.**

**[10] Zhang S, He F, Chen X, Ding K. Isolation and structural characterization of a pectin from Lycium ruthenicum Murr and its anti-pancreatic ductal adenocarcinoma cell activity. Carbohydr Polym 2019;223:115104.**

**[11] Cao YL, Li YL, Fan YF, Li Z, Yoshida K, Wang JY, et al. Wolfberry genomes and the evolution of Lycium (Solanaceae). Commun Biol 2021;4:671.**

**[12] Chen J, Liu X, Zhu L, Wang Y. Nuclear genome size estimation and karyotype analysis of Lycium species (Solanaceae). Scientia Horticulturae 2013;151:46-50.**

**[1] Gao Y, Wei Y, Wang Y, Gao F, Chen Z. Lycium Barbarum: A Traditional Chinese Herb and A Promising Anti-Aging Agent. Aging Dis 2017;8:778-91.**

**[2] Sun C, Chen X, Yang S, Jin C, Ding K, Chen C. LBP1C-2 from Lycium barbarum alleviated age-related bone loss by targeting BMPRIA/BMPRII/Noggin. Carbohydr Polym 2023;310:120725.**

**[3] Potterat O. Goji (Lycium barbarum and L. chinense): Phytochemistry, pharmacology and safety in the perspective of traditional uses and recent popularity. Planta Med 2010;76:7-19.**

**[4] Zhang XJ, Yu HY, Cai YJ, Ke M. Lycium barbarum polysaccharides inhibit proliferation and migration of bladder cancer cell lines BIU87 by suppressing Pi3K/AKT pathway. Oncotarget 2017;8:5936-42.**

**[5] Tang L, Bao S, Du Y, Jiang Z, Wuliji AO, Ren X, et al. Antioxidant effects of Lycium barbarum polysaccharides on photoreceptor degeneration in the light-exposed mouse retina. Biomed Pharmacother 2018;103:829-37.**

**[6] Zhang W, Zhang J, Ding D, Zhang L, Muehlmann LA, Deng SE, et al. Synthesis and antioxidant properties of Lycium barbarum polysaccharides capped selenium nanoparticles using tea extract. Artif Cells Nanomed Biotechnol 2018;46:1463-70.**

**[7] Ma K, Wang X, Feng S, Xia X, Zhang H, Rahaman A, et al. From the perspective of Traditional Chinese Medicine: Treatment of mental disorders in COVID-19 survivors. Biomed Pharmacother 2020;132:110810.**

**[8] Po KK, Leung JW, Chan JN, Fung TK, Sánchez-Vidaña DI, Sin EL, et al. Protective effect of Lycium Barbarum polysaccharides on dextromethorphan-induced mood impairment and neurogenesis suppression. Brain Res Bull 2017;134:10-7.**

**[9] Fang S, Dong L, Liu L, Guo J, Zhao L, Zhang J, et al. HERB: a high-throughput experiment- and reference-guided database of traditional Chinese medicine. Nucleic Acids Res 2021;49:D1197-d206.**

**[10] Zhang S, He F, Chen X, Ding K. Isolation and structural characterization of a pectin from Lycium ruthenicum Murr and its anti-pancreatic ductal adenocarcinoma cell activity. Carbohydr Polym 2019;223:115104.**

**[11] Cao YL, Li YL, Fan YF, Li Z, Yoshida K, Wang JY, et al. Wolfberry genomes and the evolution of Lycium (Solanaceae). Commun Biol 2021;4:671.**

**[12] Chen J, Liu X, Zhu L, Wang Y. Nuclear genome size estimation and karyotype analysis of Lycium species (Solanaceae). Scientia Horticulturae 2013;151:46-50.**

**[13] Giri MK, Swain S, Gautam JK, Singh S, Singh N, Bhattacharjee L, et al. The Arabidopsis thaliana At4g13040 gene, a unique member of the AP2/EREBP family, is a positive regulator for salicylic acid accumulation and basal defense against bacterial pathogens. J Plant Physiol 2014;171:860-7.**

**[1] Gao Y, Wei Y, Wang Y, Gao F, Chen Z. Lycium Barbarum: A Traditional Chinese Herb and A Promising Anti-Aging Agent. Aging Dis 2017;8:778-91.**

**[2] Sun C, Chen X, Yang S, Jin C, Ding K, Chen C. LBP1C-2 from Lycium barbarum alleviated age-related bone loss by targeting BMPRIA/BMPRII/Noggin. Carbohydr Polym 2023;310:120725.**

**[3] Potterat O. Goji (Lycium barbarum and L. chinense): Phytochemistry, pharmacology and safety in the perspective of traditional uses and recent popularity. Planta Med 2010;76:7-19.**

**[4] Zhang XJ, Yu HY, Cai YJ, Ke M. Lycium barbarum polysaccharides inhibit proliferation and migration of bladder cancer cell lines BIU87 by suppressing Pi3K/AKT pathway. Oncotarget 2017;8:5936-42.**

**[5] Tang L, Bao S, Du Y, Jiang Z, Wuliji AO, Ren X, et al. Antioxidant effects of Lycium barbarum polysaccharides on photoreceptor degeneration in the light-exposed mouse retina. Biomed Pharmacother 2018;103:829-37.**

**[6] Zhang W, Zhang J, Ding D, Zhang L, Muehlmann LA, Deng SE, et al. Synthesis and antioxidant properties of Lycium barbarum polysaccharides capped selenium nanoparticles using tea extract. Artif Cells Nanomed Biotechnol 2018;46:1463-70.**

**[7] Ma K, Wang X, Feng S, Xia X, Zhang H, Rahaman A, et al. From the perspective of Traditional Chinese Medicine: Treatment of mental disorders in COVID-19 survivors. Biomed Pharmacother 2020;132:110810.**

**[8] Po KK, Leung JW, Chan JN, Fung TK, Sánchez-Vidaña DI, Sin EL, et al. Protective effect of Lycium Barbarum polysaccharides on dextromethorphan-induced mood impairment and neurogenesis suppression. Brain Res Bull 2017;134:10-7.**

**[9] Fang S, Dong L, Liu L, Guo J, Zhao L, Zhang J, et al. HERB: a high-throughput experiment- and reference-guided database of traditional Chinese medicine. Nucleic Acids Res 2021;49:D1197-d206.**

**[10] Zhang S, He F, Chen X, Ding K. Isolation and structural characterization of a pectin from Lycium ruthenicum Murr and its anti-pancreatic ductal adenocarcinoma cell activity. Carbohydr Polym 2019;223:115104.**

**[11] Cao YL, Li YL, Fan YF, Li Z, Yoshida K, Wang JY, et al. Wolfberry genomes and the evolution of Lycium (Solanaceae). Commun Biol 2021;4:671.**

**[12] Chen J, Liu X, Zhu L, Wang Y. Nuclear genome size estimation and karyotype analysis of Lycium species (Solanaceae). Scientia Horticulturae 2013;151:46-50.**

**[13] Giri MK, Swain S, Gautam JK, Singh S, Singh N, Bhattacharjee L, et al. The Arabidopsis thaliana At4g13040 gene, a unique member of the AP2/EREBP family, is a positive regulator for salicylic acid accumulation and basal defense against bacterial pathogens. J Plant Physiol 2014;171:860-7.**

**[14] Michaels SD, Ditta G, Gustafson-Brown C, Pelaz S, Yanofsky M, Amasino RM. AGL24 acts as a promoter of flowering in Arabidopsis and is positively regulated by vernalization. Plant J 2003;33:867-74.**

**[1] Gao Y, Wei Y, Wang Y, Gao F, Chen Z. Lycium Barbarum: A Traditional Chinese Herb and A Promising Anti-Aging Agent. Aging Dis 2017;8:778-91.**

**[2] Sun C, Chen X, Yang S, Jin C, Ding K, Chen C. LBP1C-2 from Lycium barbarum alleviated age-related bone loss by targeting BMPRIA/BMPRII/Noggin. Carbohydr Polym 2023;310:120725.**

**[3] Potterat O. Goji (Lycium barbarum and L. chinense): Phytochemistry, pharmacology and safety in the perspective of traditional uses and recent popularity. Planta Med 2010;76:7-19.**

**[4] Zhang XJ, Yu HY, Cai YJ, Ke M. Lycium barbarum polysaccharides inhibit proliferation and migration of bladder cancer cell lines BIU87 by suppressing Pi3K/AKT pathway. Oncotarget 2017;8:5936-42.**

**[5] Tang L, Bao S, Du Y, Jiang Z, Wuliji AO, Ren X, et al. Antioxidant effects of Lycium barbarum polysaccharides on photoreceptor degeneration in the light-exposed mouse retina. Biomed Pharmacother 2018;103:829-37.**

**[6] Zhang W, Zhang J, Ding D, Zhang L, Muehlmann LA, Deng SE, et al. Synthesis and antioxidant properties of Lycium barbarum polysaccharides capped selenium nanoparticles using tea extract. Artif Cells Nanomed Biotechnol 2018;46:1463-70.**

**[7] Ma K, Wang X, Feng S, Xia X, Zhang H, Rahaman A, et al. From the perspective of Traditional Chinese Medicine: Treatment of mental disorders in COVID-19 survivors. Biomed Pharmacother 2020;132:110810.**

**[8] Po KK, Leung JW, Chan JN, Fung TK, Sánchez-Vidaña DI, Sin EL, et al. Protective effect of Lycium Barbarum polysaccharides on dextromethorphan-induced mood impairment and neurogenesis suppression. Brain Res Bull 2017;134:10-7.**

**[9] Fang S, Dong L, Liu L, Guo J, Zhao L, Zhang J, et al. HERB: a high-throughput experiment- and reference-guided database of traditional Chinese medicine. Nucleic Acids Res 2021;49:D1197-d206.**

**[10] Zhang S, He F, Chen X, Ding K. Isolation and structural characterization of a pectin from Lycium ruthenicum Murr and its anti-pancreatic ductal adenocarcinoma cell activity. Carbohydr Polym 2019;223:115104.**

**[11] Cao YL, Li YL, Fan YF, Li Z, Yoshida K, Wang JY, et al. Wolfberry genomes and the evolution of Lycium (Solanaceae). Commun Biol 2021;4:671.**

**[12] Chen J, Liu X, Zhu L, Wang Y. Nuclear genome size estimation and karyotype analysis of Lycium species (Solanaceae). Scientia Horticulturae 2013;151:46-50.**

**[13] Giri MK, Swain S, Gautam JK, Singh S, Singh N, Bhattacharjee L, et al. The Arabidopsis thaliana At4g13040 gene, a unique member of the AP2/EREBP family, is a positive regulator for salicylic acid accumulation and basal defense against bacterial pathogens. J Plant Physiol 2014;171:860-7.**

**[14] Michaels SD, Ditta G, Gustafson-Brown C, Pelaz S, Yanofsky M, Amasino RM. AGL24 acts as a promoter of flowering in Arabidopsis and is positively regulated by vernalization. Plant J 2003;33:867-74.**

**[15] Schauser L, Roussis A, Stiller J, Stougaard J. A plant regulator controlling development of symbiotic root nodules. Nature 1999;402:191-5.**

**[1] Gao Y, Wei Y, Wang Y, Gao F, Chen Z. Lycium Barbarum: A Traditional Chinese Herb and A Promising Anti-Aging Agent. Aging Dis 2017;8:778-91.**

**[2] Sun C, Chen X, Yang S, Jin C, Ding K, Chen C. LBP1C-2 from Lycium barbarum alleviated age-related bone loss by targeting BMPRIA/BMPRII/Noggin. Carbohydr Polym 2023;310:120725.**

**[3] Potterat O. Goji (Lycium barbarum and L. chinense): Phytochemistry, pharmacology and safety in the perspective of traditional uses and recent popularity. Planta Med 2010;76:7-19.**

**[4] Zhang XJ, Yu HY, Cai YJ, Ke M. Lycium barbarum polysaccharides inhibit proliferation and migration of bladder cancer cell lines BIU87 by suppressing Pi3K/AKT pathway. Oncotarget 2017;8:5936-42.**

**[5] Tang L, Bao S, Du Y, Jiang Z, Wuliji AO, Ren X, et al. Antioxidant effects of Lycium barbarum polysaccharides on photoreceptor degeneration in the light-exposed mouse retina. Biomed Pharmacother 2018;103:829-37.**

**[6] Zhang W, Zhang J, Ding D, Zhang L, Muehlmann LA, Deng SE, et al. Synthesis and antioxidant properties of Lycium barbarum polysaccharides capped selenium nanoparticles using tea extract. Artif Cells Nanomed Biotechnol 2018;46:1463-70.**

**[7] Ma K, Wang X, Feng S, Xia X, Zhang H, Rahaman A, et al. From the perspective of Traditional Chinese Medicine: Treatment of mental disorders in COVID-19 survivors. Biomed Pharmacother 2020;132:110810.**

**[8] Po KK, Leung JW, Chan JN, Fung TK, Sánchez-Vidaña DI, Sin EL, et al. Protective effect of Lycium Barbarum polysaccharides on dextromethorphan-induced mood impairment and neurogenesis suppression. Brain Res Bull 2017;134:10-7.**

**[9] Fang S, Dong L, Liu L, Guo J, Zhao L, Zhang J, et al. HERB: a high-throughput experiment- and reference-guided database of traditional Chinese medicine. Nucleic Acids Res 2021;49:D1197-d206.**

**[10] Zhang S, He F, Chen X, Ding K. Isolation and structural characterization of a pectin from Lycium ruthenicum Murr and its anti-pancreatic ductal adenocarcinoma cell activity. Carbohydr Polym 2019;223:115104.**

**[11] Cao YL, Li YL, Fan YF, Li Z, Yoshida K, Wang JY, et al. Wolfberry genomes and the evolution of Lycium (Solanaceae). Commun Biol 2021;4:671.**

**[12] Chen J, Liu X, Zhu L, Wang Y. Nuclear genome size estimation and karyotype analysis of Lycium species (Solanaceae). Scientia Horticulturae 2013;151:46-50.**

**[13] Giri MK, Swain S, Gautam JK, Singh S, Singh N, Bhattacharjee L, et al. The Arabidopsis thaliana At4g13040 gene, a unique member of the AP2/EREBP family, is a positive regulator for salicylic acid accumulation and basal defense against bacterial pathogens. J Plant Physiol 2014;171:860-7.**

**[14] Michaels SD, Ditta G, Gustafson-Brown C, Pelaz S, Yanofsky M, Amasino RM. AGL24 acts as a promoter of flowering in Arabidopsis and is positively regulated by vernalization. Plant J 2003;33:867-74.**

**[15] Schauser L, Roussis A, Stiller J, Stougaard J. A plant regulator controlling development of symbiotic root nodules. Nature 1999;402:191-5.**

**[16] Hou X, Zhou J, Liu C, Liu L, Shen L, Yu H. Nuclear factor Y-mediated H3K27me3 demethylation of the SOC1 locus orchestrates flowering responses of Arabidopsis. Nat Commun 2014;5:4601.**

**[1] Gao Y, Wei Y, Wang Y, Gao F, Chen Z. Lycium Barbarum: A Traditional Chinese Herb and A Promising Anti-Aging Agent. Aging Dis 2017;8:778-91.**

**[2] Sun C, Chen X, Yang S, Jin C, Ding K, Chen C. LBP1C-2 from Lycium barbarum alleviated age-related bone loss by targeting BMPRIA/BMPRII/Noggin. Carbohydr Polym 2023;310:120725.**

**[3] Potterat O. Goji (Lycium barbarum and L. chinense): Phytochemistry, pharmacology and safety in the perspective of traditional uses and recent popularity. Planta Med 2010;76:7-19.**

**[4] Zhang XJ, Yu HY, Cai YJ, Ke M. Lycium barbarum polysaccharides inhibit proliferation and migration of bladder cancer cell lines BIU87 by suppressing Pi3K/AKT pathway. Oncotarget 2017;8:5936-42.**

**[5] Tang L, Bao S, Du Y, Jiang Z, Wuliji AO, Ren X, et al. Antioxidant effects of Lycium barbarum polysaccharides on photoreceptor degeneration in the light-exposed mouse retina. Biomed Pharmacother 2018;103:829-37.**

**[6] Zhang W, Zhang J, Ding D, Zhang L, Muehlmann LA, Deng SE, et al. Synthesis and antioxidant properties of Lycium barbarum polysaccharides capped selenium nanoparticles using tea extract. Artif Cells Nanomed Biotechnol 2018;46:1463-70.**

**[7] Ma K, Wang X, Feng S, Xia X, Zhang H, Rahaman A, et al. From the perspective of Traditional Chinese Medicine: Treatment of mental disorders in COVID-19 survivors. Biomed Pharmacother 2020;132:110810.**

**[8] Po KK, Leung JW, Chan JN, Fung TK, Sánchez-Vidaña DI, Sin EL, et al. Protective effect of Lycium Barbarum polysaccharides on dextromethorphan-induced mood impairment and neurogenesis suppression. Brain Res Bull 2017;134:10-7.**

**[9] Fang S, Dong L, Liu L, Guo J, Zhao L, Zhang J, et al. HERB: a high-throughput experiment- and reference-guided database of traditional Chinese medicine. Nucleic Acids Res 2021;49:D1197-d206.**

**[10] Zhang S, He F, Chen X, Ding K. Isolation and structural characterization of a pectin from Lycium ruthenicum Murr and its anti-pancreatic ductal adenocarcinoma cell activity. Carbohydr Polym 2019;223:115104.**

**[11] Cao YL, Li YL, Fan YF, Li Z, Yoshida K, Wang JY, et al. Wolfberry genomes and the evolution of Lycium (Solanaceae). Commun Biol 2021;4:671.**

**[12] Chen J, Liu X, Zhu L, Wang Y. Nuclear genome size estimation and karyotype analysis of Lycium species (Solanaceae). Scientia Horticulturae 2013;151:46-50.**

**[13] Giri MK, Swain S, Gautam JK, Singh S, Singh N, Bhattacharjee L, et al. The Arabidopsis thaliana At4g13040 gene, a unique member of the AP2/EREBP family, is a positive regulator for salicylic acid accumulation and basal defense against bacterial pathogens. J Plant Physiol 2014;171:860-7.**

**[14] Michaels SD, Ditta G, Gustafson-Brown C, Pelaz S, Yanofsky M, Amasino RM. AGL24 acts as a promoter of flowering in Arabidopsis and is positively regulated by vernalization. Plant J 2003;33:867-74.**

**[15] Schauser L, Roussis A, Stiller J, Stougaard J. A plant regulator controlling development of symbiotic root nodules. Nature 1999;402:191-5.**

**[16] Hou X, Zhou J, Liu C, Liu L, Shen L, Yu H. Nuclear factor Y-mediated H3K27me3 demethylation of the SOC1 locus orchestrates flowering responses of Arabidopsis. Nat Commun 2014;5:4601.**

**[17] Ru L, He Y, Zhu Z, Patrick JW, Ruan YL. Integrating Sugar Metabolism With Transport: Elevation of Endogenous Cell Wall Invertase Activity Up-Regulates SlHT2 and SlSWEET12c Expression for Early Fruit Development in Tomato. Front Genet 2020;11:592596.**

**[1] Gao Y, Wei Y, Wang Y, Gao F, Chen Z. Lycium Barbarum: A Traditional Chinese Herb and A Promising Anti-Aging Agent. Aging Dis 2017;8:778-91.**

**[2] Sun C, Chen X, Yang S, Jin C, Ding K, Chen C. LBP1C-2 from Lycium barbarum alleviated age-related bone loss by targeting BMPRIA/BMPRII/Noggin. Carbohydr Polym 2023;310:120725.**

**[3] Potterat O. Goji (Lycium barbarum and L. chinense): Phytochemistry, pharmacology and safety in the perspective of traditional uses and recent popularity. Planta Med 2010;76:7-19.**

**[4] Zhang XJ, Yu HY, Cai YJ, Ke M. Lycium barbarum polysaccharides inhibit proliferation and migration of bladder cancer cell lines BIU87 by suppressing Pi3K/AKT pathway. Oncotarget 2017;8:5936-42.**

**[5] Tang L, Bao S, Du Y, Jiang Z, Wuliji AO, Ren X, et al. Antioxidant effects of Lycium barbarum polysaccharides on photoreceptor degeneration in the light-exposed mouse retina. Biomed Pharmacother 2018;103:829-37.**

**[6] Zhang W, Zhang J, Ding D, Zhang L, Muehlmann LA, Deng SE, et al. Synthesis and antioxidant properties of Lycium barbarum polysaccharides capped selenium nanoparticles using tea extract. Artif Cells Nanomed Biotechnol 2018;46:1463-70.**

**[7] Ma K, Wang X, Feng S, Xia X, Zhang H, Rahaman A, et al. From the perspective of Traditional Chinese Medicine: Treatment of mental disorders in COVID-19 survivors. Biomed Pharmacother 2020;132:110810.**

**[8] Po KK, Leung JW, Chan JN, Fung TK, Sánchez-Vidaña DI, Sin EL, et al. Protective effect of Lycium Barbarum polysaccharides on dextromethorphan-induced mood impairment and neurogenesis suppression. Brain Res Bull 2017;134:10-7.**

**[9] Fang S, Dong L, Liu L, Guo J, Zhao L, Zhang J, et al. HERB: a high-throughput experiment- and reference-guided database of traditional Chinese medicine. Nucleic Acids Res 2021;49:D1197-d206.**

**[10] Zhang S, He F, Chen X, Ding K. Isolation and structural characterization of a pectin from Lycium ruthenicum Murr and its anti-pancreatic ductal adenocarcinoma cell activity. Carbohydr Polym 2019;223:115104.**

**[11] Cao YL, Li YL, Fan YF, Li Z, Yoshida K, Wang JY, et al. Wolfberry genomes and the evolution of Lycium (Solanaceae). Commun Biol 2021;4:671.**

**[12] Chen J, Liu X, Zhu L, Wang Y. Nuclear genome size estimation and karyotype analysis of Lycium species (Solanaceae). Scientia Horticulturae 2013;151:46-50.**

**[13] Giri MK, Swain S, Gautam JK, Singh S, Singh N, Bhattacharjee L, et al. The Arabidopsis thaliana At4g13040 gene, a unique member of the AP2/EREBP family, is a positive regulator for salicylic acid accumulation and basal defense against bacterial pathogens. J Plant Physiol 2014;171:860-7.**

**[14] Michaels SD, Ditta G, Gustafson-Brown C, Pelaz S, Yanofsky M, Amasino RM. AGL24 acts as a promoter of flowering in Arabidopsis and is positively regulated by vernalization. Plant J 2003;33:867-74.**

**[15] Schauser L, Roussis A, Stiller J, Stougaard J. A plant regulator controlling development of symbiotic root nodules. Nature 1999;402:191-5.**

**[16] Hou X, Zhou J, Liu C, Liu L, Shen L, Yu H. Nuclear factor Y-mediated H3K27me3 demethylation of the SOC1 locus orchestrates flowering responses of Arabidopsis. Nat Commun 2014;5:4601.**

**[17] Ru L, He Y, Zhu Z, Patrick JW, Ruan YL. Integrating Sugar Metabolism With Transport: Elevation of Endogenous Cell Wall Invertase Activity Up-Regulates SlHT2 and SlSWEET12c Expression for Early Fruit Development in Tomato. Front Genet 2020;11:592596.**

**[18] Breia R, Conde A, Badim H, Fortes AM, Gerós H, Granell A. Plant SWEETs: from sugar transport to plant-pathogen interaction and more unexpected physiological roles. Plant Physiol 2021;186:836-52.**

**[1] Gao Y, Wei Y, Wang Y, Gao F, Chen Z. Lycium Barbarum: A Traditional Chinese Herb and A Promising Anti-Aging Agent. Aging Dis 2017;8:778-91.**

**[2] Sun C, Chen X, Yang S, Jin C, Ding K, Chen C. LBP1C-2 from Lycium barbarum alleviated age-related bone loss by targeting BMPRIA/BMPRII/Noggin. Carbohydr Polym 2023;310:120725.**

**[3] Potterat O. Goji (Lycium barbarum and L. chinense): Phytochemistry, pharmacology and safety in the perspective of traditional uses and recent popularity. Planta Med 2010;76:7-19.**

**[4] Zhang XJ, Yu HY, Cai YJ, Ke M. Lycium barbarum polysaccharides inhibit proliferation and migration of bladder cancer cell lines BIU87 by suppressing Pi3K/AKT pathway. Oncotarget 2017;8:5936-42.**

**[5] Tang L, Bao S, Du Y, Jiang Z, Wuliji AO, Ren X, et al. Antioxidant effects of Lycium barbarum polysaccharides on photoreceptor degeneration in the light-exposed mouse retina. Biomed Pharmacother 2018;103:829-37.**

**[6] Zhang W, Zhang J, Ding D, Zhang L, Muehlmann LA, Deng SE, et al. Synthesis and antioxidant properties of Lycium barbarum polysaccharides capped selenium nanoparticles using tea extract. Artif Cells Nanomed Biotechnol 2018;46:1463-70.**

**[7] Ma K, Wang X, Feng S, Xia X, Zhang H, Rahaman A, et al. From the perspective of Traditional Chinese Medicine: Treatment of mental disorders in COVID-19 survivors. Biomed Pharmacother 2020;132:110810.**

**[8] Po KK, Leung JW, Chan JN, Fung TK, Sánchez-Vidaña DI, Sin EL, et al. Protective effect of Lycium Barbarum polysaccharides on dextromethorphan-induced mood impairment and neurogenesis suppression. Brain Res Bull 2017;134:10-7.**

**[9] Fang S, Dong L, Liu L, Guo J, Zhao L, Zhang J, et al. HERB: a high-throughput experiment- and reference-guided database of traditional Chinese medicine. Nucleic Acids Res 2021;49:D1197-d206.**

**[10] Zhang S, He F, Chen X, Ding K. Isolation and structural characterization of a pectin from Lycium ruthenicum Murr and its anti-pancreatic ductal adenocarcinoma cell activity. Carbohydr Polym 2019;223:115104.**

**[11] Cao YL, Li YL, Fan YF, Li Z, Yoshida K, Wang JY, et al. Wolfberry genomes and the evolution of Lycium (Solanaceae). Commun Biol 2021;4:671.**

**[12] Chen J, Liu X, Zhu L, Wang Y. Nuclear genome size estimation and karyotype analysis of Lycium species (Solanaceae). Scientia Horticulturae 2013;151:46-50.**

**[13] Giri MK, Swain S, Gautam JK, Singh S, Singh N, Bhattacharjee L, et al. The Arabidopsis thaliana At4g13040 gene, a unique member of the AP2/EREBP family, is a positive regulator for salicylic acid accumulation and basal defense against bacterial pathogens. J Plant Physiol 2014;171:860-7.**

**[14] Michaels SD, Ditta G, Gustafson-Brown C, Pelaz S, Yanofsky M, Amasino RM. AGL24 acts as a promoter of flowering in Arabidopsis and is positively regulated by vernalization. Plant J 2003;33:867-74.**

**[15] Schauser L, Roussis A, Stiller J, Stougaard J. A plant regulator controlling development of symbiotic root nodules. Nature 1999;402:191-5.**

**[16] Hou X, Zhou J, Liu C, Liu L, Shen L, Yu H. Nuclear factor Y-mediated H3K27me3 demethylation of the SOC1 locus orchestrates flowering responses of Arabidopsis. Nat Commun 2014;5:4601.**

**[17] Ru L, He Y, Zhu Z, Patrick JW, Ruan YL. Integrating Sugar Metabolism With Transport: Elevation of Endogenous Cell Wall Invertase Activity Up-Regulates SlHT2 and SlSWEET12c Expression for Early Fruit Development in Tomato. Front Genet 2020;11:592596.**

**[18] Breia R, Conde A, Badim H, Fortes AM, Gerós H, Granell A. Plant SWEETs: from sugar transport to plant-pathogen interaction and more unexpected physiological roles. Plant Physiol 2021;186:836-52.**

**[19] Chen LQ, Hou BH, Lalonde S, Takanaga H, Hartung ML, Qu XQ, et al. Sugar transporters for intercellular exchange and nutrition of pathogens. Nature 2010;468:527-32.**

**[1] Gao Y, Wei Y, Wang Y, Gao F, Chen Z. Lycium Barbarum: A Traditional Chinese Herb and A Promising Anti-Aging Agent. Aging Dis 2017;8:778-91.**

**[2] Sun C, Chen X, Yang S, Jin C, Ding K, Chen C. LBP1C-2 from Lycium barbarum alleviated age-related bone loss by targeting BMPRIA/BMPRII/Noggin. Carbohydr Polym 2023;310:120725.**

**[3] Potterat O. Goji (Lycium barbarum and L. chinense): Phytochemistry, pharmacology and safety in the perspective of traditional uses and recent popularity. Planta Med 2010;76:7-19.**

**[4] Zhang XJ, Yu HY, Cai YJ, Ke M. Lycium barbarum polysaccharides inhibit proliferation and migration of bladder cancer cell lines BIU87 by suppressing Pi3K/AKT pathway. Oncotarget 2017;8:5936-42.**

**[5] Tang L, Bao S, Du Y, Jiang Z, Wuliji AO, Ren X, et al. Antioxidant effects of Lycium barbarum polysaccharides on photoreceptor degeneration in the light-exposed mouse retina. Biomed Pharmacother 2018;103:829-37.**

**[6] Zhang W, Zhang J, Ding D, Zhang L, Muehlmann LA, Deng SE, et al. Synthesis and antioxidant properties of Lycium barbarum polysaccharides capped selenium nanoparticles using tea extract. Artif Cells Nanomed Biotechnol 2018;46:1463-70.**

**[7] Ma K, Wang X, Feng S, Xia X, Zhang H, Rahaman A, et al. From the perspective of Traditional Chinese Medicine: Treatment of mental disorders in COVID-19 survivors. Biomed Pharmacother 2020;132:110810.**

**[8] Po KK, Leung JW, Chan JN, Fung TK, Sánchez-Vidaña DI, Sin EL, et al. Protective effect of Lycium Barbarum polysaccharides on dextromethorphan-induced mood impairment and neurogenesis suppression. Brain Res Bull 2017;134:10-7.**

**[9] Fang S, Dong L, Liu L, Guo J, Zhao L, Zhang J, et al. HERB: a high-throughput experiment- and reference-guided database of traditional Chinese medicine. Nucleic Acids Res 2021;49:D1197-d206.**

**[10] Zhang S, He F, Chen X, Ding K. Isolation and structural characterization of a pectin from Lycium ruthenicum Murr and its anti-pancreatic ductal adenocarcinoma cell activity. Carbohydr Polym 2019;223:115104.**

**[11] Cao YL, Li YL, Fan YF, Li Z, Yoshida K, Wang JY, et al. Wolfberry genomes and the evolution of Lycium (Solanaceae). Commun Biol 2021;4:671.**

**[12] Chen J, Liu X, Zhu L, Wang Y. Nuclear genome size estimation and karyotype analysis of Lycium species (Solanaceae). Scientia Horticulturae 2013;151:46-50.**

**[13] Giri MK, Swain S, Gautam JK, Singh S, Singh N, Bhattacharjee L, et al. The Arabidopsis thaliana At4g13040 gene, a unique member of the AP2/EREBP family, is a positive regulator for salicylic acid accumulation and basal defense against bacterial pathogens. J Plant Physiol 2014;171:860-7.**

**[14] Michaels SD, Ditta G, Gustafson-Brown C, Pelaz S, Yanofsky M, Amasino RM. AGL24 acts as a promoter of flowering in Arabidopsis and is positively regulated by vernalization. Plant J 2003;33:867-74.**

**[15] Schauser L, Roussis A, Stiller J, Stougaard J. A plant regulator controlling development of symbiotic root nodules. Nature 1999;402:191-5.**

**[16] Hou X, Zhou J, Liu C, Liu L, Shen L, Yu H. Nuclear factor Y-mediated H3K27me3 demethylation of the SOC1 locus orchestrates flowering responses of Arabidopsis. Nat Commun 2014;5:4601.**

**[17] Ru L, He Y, Zhu Z, Patrick JW, Ruan YL. Integrating Sugar Metabolism With Transport: Elevation of Endogenous Cell Wall Invertase Activity Up-Regulates SlHT2 and SlSWEET12c Expression for Early Fruit Development in Tomato. Front Genet 2020;11:592596.**

**[18] Breia R, Conde A, Badim H, Fortes AM, Gerós H, Granell A. Plant SWEETs: from sugar transport to plant-pathogen interaction and more unexpected physiological roles. Plant Physiol 2021;186:836-52.**

**[19] Chen LQ, Hou BH, Lalonde S, Takanaga H, Hartung ML, Qu XQ, et al. Sugar transporters for intercellular exchange and nutrition of pathogens. Nature 2010;468:527-32.**

**[20] Eom JS, Chen LQ, Sosso D, Julius BT, Lin IW, Qu XQ, et al. SWEETs, transporters for intracellular and intercellular sugar translocation. Curr Opin Plant Biol 2015;25:53-62.**

**[1] Gao Y, Wei Y, Wang Y, Gao F, Chen Z. Lycium Barbarum: A Traditional Chinese Herb and A Promising Anti-Aging Agent. Aging Dis 2017;8:778-91.**

**[2] Sun C, Chen X, Yang S, Jin C, Ding K, Chen C. LBP1C-2 from Lycium barbarum alleviated age-related bone loss by targeting BMPRIA/BMPRII/Noggin. Carbohydr Polym 2023;310:120725.**

**[3] Potterat O. Goji (Lycium barbarum and L. chinense): Phytochemistry, pharmacology and safety in the perspective of traditional uses and recent popularity. Planta Med 2010;76:7-19.**

**[4] Zhang XJ, Yu HY, Cai YJ, Ke M. Lycium barbarum polysaccharides inhibit proliferation and migration of bladder cancer cell lines BIU87 by suppressing Pi3K/AKT pathway. Oncotarget 2017;8:5936-42.**

**[5] Tang L, Bao S, Du Y, Jiang Z, Wuliji AO, Ren X, et al. Antioxidant effects of Lycium barbarum polysaccharides on photoreceptor degeneration in the light-exposed mouse retina. Biomed Pharmacother 2018;103:829-37.**

**[6] Zhang W, Zhang J, Ding D, Zhang L, Muehlmann LA, Deng SE, et al. Synthesis and antioxidant properties of Lycium barbarum polysaccharides capped selenium nanoparticles using tea extract. Artif Cells Nanomed Biotechnol 2018;46:1463-70.**

**[7] Ma K, Wang X, Feng S, Xia X, Zhang H, Rahaman A, et al. From the perspective of Traditional Chinese Medicine: Treatment of mental disorders in COVID-19 survivors. Biomed Pharmacother 2020;132:110810.**

**[8] Po KK, Leung JW, Chan JN, Fung TK, Sánchez-Vidaña DI, Sin EL, et al. Protective effect of Lycium Barbarum polysaccharides on dextromethorphan-induced mood impairment and neurogenesis suppression. Brain Res Bull 2017;134:10-7.**

**[9] Fang S, Dong L, Liu L, Guo J, Zhao L, Zhang J, et al. HERB: a high-throughput experiment- and reference-guided database of traditional Chinese medicine. Nucleic Acids Res 2021;49:D1197-d206.**

**[10] Zhang S, He F, Chen X, Ding K. Isolation and structural characterization of a pectin from Lycium ruthenicum Murr and its anti-pancreatic ductal adenocarcinoma cell activity. Carbohydr Polym 2019;223:115104.**

**[11] Cao YL, Li YL, Fan YF, Li Z, Yoshida K, Wang JY, et al. Wolfberry genomes and the evolution of Lycium (Solanaceae). Commun Biol 2021;4:671.**

**[12] Chen J, Liu X, Zhu L, Wang Y. Nuclear genome size estimation and karyotype analysis of Lycium species (Solanaceae). Scientia Horticulturae 2013;151:46-50.**

**[13] Giri MK, Swain S, Gautam JK, Singh S, Singh N, Bhattacharjee L, et al. The Arabidopsis thaliana At4g13040 gene, a unique member of the AP2/EREBP family, is a positive regulator for salicylic acid accumulation and basal defense against bacterial pathogens. J Plant Physiol 2014;171:860-7.**

**[14] Michaels SD, Ditta G, Gustafson-Brown C, Pelaz S, Yanofsky M, Amasino RM. AGL24 acts as a promoter of flowering in Arabidopsis and is positively regulated by vernalization. Plant J 2003;33:867-74.**

**[15] Schauser L, Roussis A, Stiller J, Stougaard J. A plant regulator controlling development of symbiotic root nodules. Nature 1999;402:191-5.**

**[16] Hou X, Zhou J, Liu C, Liu L, Shen L, Yu H. Nuclear factor Y-mediated H3K27me3 demethylation of the SOC1 locus orchestrates flowering responses of Arabidopsis. Nat Commun 2014;5:4601.**

**[17] Ru L, He Y, Zhu Z, Patrick JW, Ruan YL. Integrating Sugar Metabolism With Transport: Elevation of Endogenous Cell Wall Invertase Activity Up-Regulates SlHT2 and SlSWEET12c Expression for Early Fruit Development in Tomato. Front Genet 2020;11:592596.**

**[18] Breia R, Conde A, Badim H, Fortes AM, Gerós H, Granell A. Plant SWEETs: from sugar transport to plant-pathogen interaction and more unexpected physiological roles. Plant Physiol 2021;186:836-52.**

**[19] Chen LQ, Hou BH, Lalonde S, Takanaga H, Hartung ML, Qu XQ, et al. Sugar transporters for intercellular exchange and nutrition of pathogens. Nature 2010;468:527-32.**

**[20] Eom JS, Chen LQ, Sosso D, Julius BT, Lin IW, Qu XQ, et al. SWEETs, transporters for intracellular and intercellular sugar translocation. Curr Opin Plant Biol 2015;25:53-62.**

**[21] Li Y, Feng S, Ma S, Sui X, Zhang Z. Spatiotemporal Expression and Substrate Specificity Analysis of the Cucumber SWEET Gene Family. Front Plant Sci 2017;8:1855.**

**[1] Gao Y, Wei Y, Wang Y, Gao F, Chen Z. Lycium Barbarum: A Traditional Chinese Herb and A Promising Anti-Aging Agent. Aging Dis 2017;8:778-91.**

**[2] Sun C, Chen X, Yang S, Jin C, Ding K, Chen C. LBP1C-2 from Lycium barbarum alleviated age-related bone loss by targeting BMPRIA/BMPRII/Noggin. Carbohydr Polym 2023;310:120725.**

**[3] Potterat O. Goji (Lycium barbarum and L. chinense): Phytochemistry, pharmacology and safety in the perspective of traditional uses and recent popularity. Planta Med 2010;76:7-19.**

**[4] Zhang XJ, Yu HY, Cai YJ, Ke M. Lycium barbarum polysaccharides inhibit proliferation and migration of bladder cancer cell lines BIU87 by suppressing Pi3K/AKT pathway. Oncotarget 2017;8:5936-42.**

**[5] Tang L, Bao S, Du Y, Jiang Z, Wuliji AO, Ren X, et al. Antioxidant effects of Lycium barbarum polysaccharides on photoreceptor degeneration in the light-exposed mouse retina. Biomed Pharmacother 2018;103:829-37.**

**[6] Zhang W, Zhang J, Ding D, Zhang L, Muehlmann LA, Deng SE, et al. Synthesis and antioxidant properties of Lycium barbarum polysaccharides capped selenium nanoparticles using tea extract. Artif Cells Nanomed Biotechnol 2018;46:1463-70.**

**[7] Ma K, Wang X, Feng S, Xia X, Zhang H, Rahaman A, et al. From the perspective of Traditional Chinese Medicine: Treatment of mental disorders in COVID-19 survivors. Biomed Pharmacother 2020;132:110810.**

**[8] Po KK, Leung JW, Chan JN, Fung TK, Sánchez-Vidaña DI, Sin EL, et al. Protective effect of Lycium Barbarum polysaccharides on dextromethorphan-induced mood impairment and neurogenesis suppression. Brain Res Bull 2017;134:10-7.**

**[9] Fang S, Dong L, Liu L, Guo J, Zhao L, Zhang J, et al. HERB: a high-throughput experiment- and reference-guided database of traditional Chinese medicine. Nucleic Acids Res 2021;49:D1197-d206.**

**[10] Zhang S, He F, Chen X, Ding K. Isolation and structural characterization of a pectin from Lycium ruthenicum Murr and its anti-pancreatic ductal adenocarcinoma cell activity. Carbohydr Polym 2019;223:115104.**

**[11] Cao YL, Li YL, Fan YF, Li Z, Yoshida K, Wang JY, et al. Wolfberry genomes and the evolution of Lycium (Solanaceae). Commun Biol 2021;4:671.**

**[12] Chen J, Liu X, Zhu L, Wang Y. Nuclear genome size estimation and karyotype analysis of Lycium species (Solanaceae). Scientia Horticulturae 2013;151:46-50.**

**[13] Giri MK, Swain S, Gautam JK, Singh S, Singh N, Bhattacharjee L, et al. The Arabidopsis thaliana At4g13040 gene, a unique member of the AP2/EREBP family, is a positive regulator for salicylic acid accumulation and basal defense against bacterial pathogens. J Plant Physiol 2014;171:860-7.**

**[14] Michaels SD, Ditta G, Gustafson-Brown C, Pelaz S, Yanofsky M, Amasino RM. AGL24 acts as a promoter of flowering in Arabidopsis and is positively regulated by vernalization. Plant J 2003;33:867-74.**

**[15] Schauser L, Roussis A, Stiller J, Stougaard J. A plant regulator controlling development of symbiotic root nodules. Nature 1999;402:191-5.**

**[16] Hou X, Zhou J, Liu C, Liu L, Shen L, Yu H. Nuclear factor Y-mediated H3K27me3 demethylation of the SOC1 locus orchestrates flowering responses of Arabidopsis. Nat Commun 2014;5:4601.**

**[17] Ru L, He Y, Zhu Z, Patrick JW, Ruan YL. Integrating Sugar Metabolism With Transport: Elevation of Endogenous Cell Wall Invertase Activity Up-Regulates SlHT2 and SlSWEET12c Expression for Early Fruit Development in Tomato. Front Genet 2020;11:592596.**

**[18] Breia R, Conde A, Badim H, Fortes AM, Gerós H, Granell A. Plant SWEETs: from sugar transport to plant-pathogen interaction and more unexpected physiological roles. Plant Physiol 2021;186:836-52.**

**[19] Chen LQ, Hou BH, Lalonde S, Takanaga H, Hartung ML, Qu XQ, et al. Sugar transporters for intercellular exchange and nutrition of pathogens. Nature 2010;468:527-32.**

**[20] Eom JS, Chen LQ, Sosso D, Julius BT, Lin IW, Qu XQ, et al. SWEETs, transporters for intracellular and intercellular sugar translocation. Curr Opin Plant Biol 2015;25:53-62.**

**[21] Li Y, Feng S, Ma S, Sui X, Zhang Z. Spatiotemporal Expression and Substrate Specificity Analysis of the Cucumber SWEET Gene Family. Front Plant Sci 2017;8:1855.**

**[22] Patil G, Valliyodan B, Deshmukh R, Prince S, Nicander B, Zhao M, et al. Soybean (Glycine max) SWEET gene family: insights through comparative genomics, transcriptome profiling and whole genome re-sequence analysis. BMC Genomics 2015;16:520.**

**[1] Gao Y, Wei Y, Wang Y, Gao F, Chen Z. Lycium Barbarum: A Traditional Chinese Herb and A Promising Anti-Aging Agent. Aging Dis 2017;8:778-91.**

**[2] Sun C, Chen X, Yang S, Jin C, Ding K, Chen C. LBP1C-2 from Lycium barbarum alleviated age-related bone loss by targeting BMPRIA/BMPRII/Noggin. Carbohydr Polym 2023;310:120725.**

**[3] Potterat O. Goji (Lycium barbarum and L. chinense): Phytochemistry, pharmacology and safety in the perspective of traditional uses and recent popularity. Planta Med 2010;76:7-19.**

**[4] Zhang XJ, Yu HY, Cai YJ, Ke M. Lycium barbarum polysaccharides inhibit proliferation and migration of bladder cancer cell lines BIU87 by suppressing Pi3K/AKT pathway. Oncotarget 2017;8:5936-42.**

**[5] Tang L, Bao S, Du Y, Jiang Z, Wuliji AO, Ren X, et al. Antioxidant effects of Lycium barbarum polysaccharides on photoreceptor degeneration in the light-exposed mouse retina. Biomed Pharmacother 2018;103:829-37.**

**[6] Zhang W, Zhang J, Ding D, Zhang L, Muehlmann LA, Deng SE, et al. Synthesis and antioxidant properties of Lycium barbarum polysaccharides capped selenium nanoparticles using tea extract. Artif Cells Nanomed Biotechnol 2018;46:1463-70.**

**[7] Ma K, Wang X, Feng S, Xia X, Zhang H, Rahaman A, et al. From the perspective of Traditional Chinese Medicine: Treatment of mental disorders in COVID-19 survivors. Biomed Pharmacother 2020;132:110810.**

**[8] Po KK, Leung JW, Chan JN, Fung TK, Sánchez-Vidaña DI, Sin EL, et al. Protective effect of Lycium Barbarum polysaccharides on dextromethorphan-induced mood impairment and neurogenesis suppression. Brain Res Bull 2017;134:10-7.**

**[9] Fang S, Dong L, Liu L, Guo J, Zhao L, Zhang J, et al. HERB: a high-throughput experiment- and reference-guided database of traditional Chinese medicine. Nucleic Acids Res 2021;49:D1197-d206.**

**[10] Zhang S, He F, Chen X, Ding K. Isolation and structural characterization of a pectin from Lycium ruthenicum Murr and its anti-pancreatic ductal adenocarcinoma cell activity. Carbohydr Polym 2019;223:115104.**

**[11] Cao YL, Li YL, Fan YF, Li Z, Yoshida K, Wang JY, et al. Wolfberry genomes and the evolution of Lycium (Solanaceae). Commun Biol 2021;4:671.**

**[12] Chen J, Liu X, Zhu L, Wang Y. Nuclear genome size estimation and karyotype analysis of Lycium species (Solanaceae). Scientia Horticulturae 2013;151:46-50.**

**[13] Giri MK, Swain S, Gautam JK, Singh S, Singh N, Bhattacharjee L, et al. The Arabidopsis thaliana At4g13040 gene, a unique member of the AP2/EREBP family, is a positive regulator for salicylic acid accumulation and basal defense against bacterial pathogens. J Plant Physiol 2014;171:860-7.**

**[14] Michaels SD, Ditta G, Gustafson-Brown C, Pelaz S, Yanofsky M, Amasino RM. AGL24 acts as a promoter of flowering in Arabidopsis and is positively regulated by vernalization. Plant J 2003;33:867-74.**

**[15] Schauser L, Roussis A, Stiller J, Stougaard J. A plant regulator controlling development of symbiotic root nodules. Nature 1999;402:191-5.**

**[16] Hou X, Zhou J, Liu C, Liu L, Shen L, Yu H. Nuclear factor Y-mediated H3K27me3 demethylation of the SOC1 locus orchestrates flowering responses of Arabidopsis. Nat Commun 2014;5:4601.**

**[17] Ru L, He Y, Zhu Z, Patrick JW, Ruan YL. Integrating Sugar Metabolism With Transport: Elevation of Endogenous Cell Wall Invertase Activity Up-Regulates SlHT2 and SlSWEET12c Expression for Early Fruit Development in Tomato. Front Genet 2020;11:592596.**

**[18] Breia R, Conde A, Badim H, Fortes AM, Gerós H, Granell A. Plant SWEETs: from sugar transport to plant-pathogen interaction and more unexpected physiological roles. Plant Physiol 2021;186:836-52.**

**[19] Chen LQ, Hou BH, Lalonde S, Takanaga H, Hartung ML, Qu XQ, et al. Sugar transporters for intercellular exchange and nutrition of pathogens. Nature 2010;468:527-32.**

**[20] Eom JS, Chen LQ, Sosso D, Julius BT, Lin IW, Qu XQ, et al. SWEETs, transporters for intracellular and intercellular sugar translocation. Curr Opin Plant Biol 2015;25:53-62.**

**[21] Li Y, Feng S, Ma S, Sui X, Zhang Z. Spatiotemporal Expression and Substrate Specificity Analysis of the Cucumber SWEET Gene Family. Front Plant Sci 2017;8:1855.**

**[22] Patil G, Valliyodan B, Deshmukh R, Prince S, Nicander B, Zhao M, et al. Soybean (Glycine max) SWEET gene family: insights through comparative genomics, transcriptome profiling and whole genome re-sequence analysis. BMC Genomics 2015;16:520.**

**[23] Zhang W, Wang S, Yu F, Tang J, Shan X, Bao K, et al. Genome-wide characterization and expression profiling of SWEET genes in cabbage (Brassica oleracea var. capitata L.) reveal their roles in chilling and clubroot disease responses. BMC Genomics 2019;20:93.**

**[1] Gao Y, Wei Y, Wang Y, Gao F, Chen Z. Lycium Barbarum: A Traditional Chinese Herb and A Promising Anti-Aging Agent. Aging Dis 2017;8:778-91.**

**[2] Sun C, Chen X, Yang S, Jin C, Ding K, Chen C. LBP1C-2 from Lycium barbarum alleviated age-related bone loss by targeting BMPRIA/BMPRII/Noggin. Carbohydr Polym 2023;310:120725.**

**[3] Potterat O. Goji (Lycium barbarum and L. chinense): Phytochemistry, pharmacology and safety in the perspective of traditional uses and recent popularity. Planta Med 2010;76:7-19.**

**[4] Zhang XJ, Yu HY, Cai YJ, Ke M. Lycium barbarum polysaccharides inhibit proliferation and migration of bladder cancer cell lines BIU87 by suppressing Pi3K/AKT pathway. Oncotarget 2017;8:5936-42.**

**[5] Tang L, Bao S, Du Y, Jiang Z, Wuliji AO, Ren X, et al. Antioxidant effects of Lycium barbarum polysaccharides on photoreceptor degeneration in the light-exposed mouse retina. Biomed Pharmacother 2018;103:829-37.**

**[6] Zhang W, Zhang J, Ding D, Zhang L, Muehlmann LA, Deng SE, et al. Synthesis and antioxidant properties of Lycium barbarum polysaccharides capped selenium nanoparticles using tea extract. Artif Cells Nanomed Biotechnol 2018;46:1463-70.**

**[7] Ma K, Wang X, Feng S, Xia X, Zhang H, Rahaman A, et al. From the perspective of Traditional Chinese Medicine: Treatment of mental disorders in COVID-19 survivors. Biomed Pharmacother 2020;132:110810.**

**[8] Po KK, Leung JW, Chan JN, Fung TK, Sánchez-Vidaña DI, Sin EL, et al. Protective effect of Lycium Barbarum polysaccharides on dextromethorphan-induced mood impairment and neurogenesis suppression. Brain Res Bull 2017;134:10-7.**

**[9] Fang S, Dong L, Liu L, Guo J, Zhao L, Zhang J, et al. HERB: a high-throughput experiment- and reference-guided database of traditional Chinese medicine. Nucleic Acids Res 2021;49:D1197-d206.**

**[10] Zhang S, He F, Chen X, Ding K. Isolation and structural characterization of a pectin from Lycium ruthenicum Murr and its anti-pancreatic ductal adenocarcinoma cell activity. Carbohydr Polym 2019;223:115104.**

**[11] Cao YL, Li YL, Fan YF, Li Z, Yoshida K, Wang JY, et al. Wolfberry genomes and the evolution of Lycium (Solanaceae). Commun Biol 2021;4:671.**

**[12] Chen J, Liu X, Zhu L, Wang Y. Nuclear genome size estimation and karyotype analysis of Lycium species (Solanaceae). Scientia Horticulturae 2013;151:46-50.**

**[13] Giri MK, Swain S, Gautam JK, Singh S, Singh N, Bhattacharjee L, et al. The Arabidopsis thaliana At4g13040 gene, a unique member of the AP2/EREBP family, is a positive regulator for salicylic acid accumulation and basal defense against bacterial pathogens. J Plant Physiol 2014;171:860-7.**

**[14] Michaels SD, Ditta G, Gustafson-Brown C, Pelaz S, Yanofsky M, Amasino RM. AGL24 acts as a promoter of flowering in Arabidopsis and is positively regulated by vernalization. Plant J 2003;33:867-74.**

**[15] Schauser L, Roussis A, Stiller J, Stougaard J. A plant regulator controlling development of symbiotic root nodules. Nature 1999;402:191-5.**

**[16] Hou X, Zhou J, Liu C, Liu L, Shen L, Yu H. Nuclear factor Y-mediated H3K27me3 demethylation of the SOC1 locus orchestrates flowering responses of Arabidopsis. Nat Commun 2014;5:4601.**

**[17] Ru L, He Y, Zhu Z, Patrick JW, Ruan YL. Integrating Sugar Metabolism With Transport: Elevation of Endogenous Cell Wall Invertase Activity Up-Regulates SlHT2 and SlSWEET12c Expression for Early Fruit Development in Tomato. Front Genet 2020;11:592596.**

**[18] Breia R, Conde A, Badim H, Fortes AM, Gerós H, Granell A. Plant SWEETs: from sugar transport to plant-pathogen interaction and more unexpected physiological roles. Plant Physiol 2021;186:836-52.**

**[19] Chen LQ, Hou BH, Lalonde S, Takanaga H, Hartung ML, Qu XQ, et al. Sugar transporters for intercellular exchange and nutrition of pathogens. Nature 2010;468:527-32.**

**[20] Eom JS, Chen LQ, Sosso D, Julius BT, Lin IW, Qu XQ, et al. SWEETs, transporters for intracellular and intercellular sugar translocation. Curr Opin Plant Biol 2015;25:53-62.**

**[21] Li Y, Feng S, Ma S, Sui X, Zhang Z. Spatiotemporal Expression and Substrate Specificity Analysis of the Cucumber SWEET Gene Family. Front Plant Sci 2017;8:1855.**

**[23] Zhang W, Wang S, Yu F, Tang J, Shan X, Bao K, et al. Genome-wide characterization and expression profiling of SWEET genes in cabbage (Brassica oleracea var. capitata L.) reveal their roles in chilling and clubroot disease responses. BMC Genomics 2019;20:93.**

**[24] Feng CY, Han JX, Han XX, Jiang J. Genome-wide identification, phylogeny, and expression analysis of the SWEET gene family in tomato. Gene 2015;573:261-72.**

**[1] Gao Y, Wei Y, Wang Y, Gao F, Chen Z. Lycium Barbarum: A Traditional Chinese Herb and A Promising Anti-Aging Agent. Aging Dis 2017;8:778-91.**

**[2] Sun C, Chen X, Yang S, Jin C, Ding K, Chen C. LBP1C-2 from Lycium barbarum alleviated age-related bone loss by targeting BMPRIA/BMPRII/Noggin. Carbohydr Polym 2023;310:120725.**

**[3] Potterat O. Goji (Lycium barbarum and L. chinense): Phytochemistry, pharmacology and safety in the perspective of traditional uses and recent popularity. Planta Med 2010;76:7-19.**

**[4] Zhang XJ, Yu HY, Cai YJ, Ke M. Lycium barbarum polysaccharides inhibit proliferation and migration of bladder cancer cell lines BIU87 by suppressing Pi3K/AKT pathway. Oncotarget 2017;8:5936-42.**

**[5] Tang L, Bao S, Du Y, Jiang Z, Wuliji AO, Ren X, et al. Antioxidant effects of Lycium barbarum polysaccharides on photoreceptor degeneration in the light-exposed mouse retina. Biomed Pharmacother 2018;103:829-37.**

**[6] Zhang W, Zhang J, Ding D, Zhang L, Muehlmann LA, Deng SE, et al. Synthesis and antioxidant properties of Lycium barbarum polysaccharides capped selenium nanoparticles using tea extract. Artif Cells Nanomed Biotechnol 2018;46:1463-70.**

**[7] Ma K, Wang X, Feng S, Xia X, Zhang H, Rahaman A, et al. From the perspective of Traditional Chinese Medicine: Treatment of mental disorders in COVID-19 survivors. Biomed Pharmacother 2020;132:110810.**

**[8] Po KK, Leung JW, Chan JN, Fung TK, Sánchez-Vidaña DI, Sin EL, et al. Protective effect of Lycium Barbarum polysaccharides on dextromethorphan-induced mood impairment and neurogenesis suppression. Brain Res Bull 2017;134:10-7.**

**[9] Fang S, Dong L, Liu L, Guo J, Zhao L, Zhang J, et al. HERB: a high-throughput experiment- and reference-guided database of traditional Chinese medicine. Nucleic Acids Res 2021;49:D1197-d206.**

**[10] Zhang S, He F, Chen X, Ding K. Isolation and structural characterization of a pectin from Lycium ruthenicum Murr and its anti-pancreatic ductal adenocarcinoma cell activity. Carbohydr Polym 2019;223:115104.**

**[11] Cao YL, Li YL, Fan YF, Li Z, Yoshida K, Wang JY, et al. Wolfberry genomes and the evolution of Lycium (Solanaceae). Commun Biol 2021;4:671.**

**[12] Chen J, Liu X, Zhu L, Wang Y. Nuclear genome size estimation and karyotype analysis of Lycium species (Solanaceae). Scientia Horticulturae 2013;151:46-50.**

**[13] Giri MK, Swain S, Gautam JK, Singh S, Singh N, Bhattacharjee L, et al. The Arabidopsis thaliana At4g13040 gene, a unique member of the AP2/EREBP family, is a positive regulator for salicylic acid accumulation and basal defense against bacterial pathogens. J Plant Physiol 2014;171:860-7.**

**[14] Michaels SD, Ditta G, Gustafson-Brown C, Pelaz S, Yanofsky M, Amasino RM. AGL24 acts as a promoter of flowering in Arabidopsis and is positively regulated by vernalization. Plant J 2003;33:867-74.**

**[15] Schauser L, Roussis A, Stiller J, Stougaard J. A plant regulator controlling development of symbiotic root nodules. Nature 1999;402:191-5.**

**[16] Hou X, Zhou J, Liu C, Liu L, Shen L, Yu H. Nuclear factor Y-mediated H3K27me3 demethylation of the SOC1 locus orchestrates flowering responses of Arabidopsis. Nat Commun 2014;5:4601.**

**[17] Ru L, He Y, Zhu Z, Patrick JW, Ruan YL. Integrating Sugar Metabolism With Transport: Elevation of Endogenous Cell Wall Invertase Activity Up-Regulates SlHT2 and SlSWEET12c Expression for Early Fruit Development in Tomato. Front Genet 2020;11:592596.**

**[18] Breia R, Conde A, Badim H, Fortes AM, Gerós H, Granell A. Plant SWEETs: from sugar transport to plant-pathogen interaction and more unexpected physiological roles. Plant Physiol 2021;186:836-52.**

**[19] Chen LQ, Hou BH, Lalonde S, Takanaga H, Hartung ML, Qu XQ, et al. Sugar transporters for intercellular exchange and nutrition of pathogens. Nature 2010;468:527-32.**

**[20] Eom JS, Chen LQ, Sosso D, Julius BT, Lin IW, Qu XQ, et al. SWEETs, transporters for intracellular and intercellular sugar translocation. Curr Opin Plant Biol 2015;25:53-62.**

**[21] Li Y, Feng S, Ma S, Sui X, Zhang Z. Spatiotemporal Expression and Substrate Specificity Analysis of the Cucumber SWEET Gene Family. Front Plant Sci 2017;8:1855.**

**[22] Patil G, Valliyodan B, Deshmukh R, Prince S, Nicander B, Zhao M, et al. Soybean (Glycine max) SWEET gene family: insights through comparative genomics, transcriptome profiling and whole genome re-sequence analysis. BMC Genomics 2015;16:520.**

**[23] Zhang W, Wang S, Yu F, Tang J, Shan X, Bao K, et al. Genome-wide characterization and expression profiling of SWEET genes in cabbage (Brassica oleracea var. capitata L.) reveal their roles in chilling and clubroot disease responses. BMC Genomics 2019;20:93.**

**[24] Feng CY, Han JX, Han XX, Jiang J. Genome-wide identification, phylogeny, and expression analysis of the SWEET gene family in tomato. Gene 2015;573:261-72.**

**[25] Manck-Götzenberger J, Requena N. Arbuscular mycorrhiza Symbiosis Induces a Major Transcriptional Reprogramming of the Potato SWEET Sugar Transporter Family. Front Plant Sci 2016;7:487.**

**[1] Gao Y, Wei Y, Wang Y, Gao F, Chen Z. Lycium Barbarum: A Traditional Chinese Herb and A Promising Anti-Aging Agent. Aging Dis 2017;8:778-91.**

**[2] Sun C, Chen X, Yang S, Jin C, Ding K, Chen C. LBP1C-2 from Lycium barbarum alleviated age-related bone loss by targeting BMPRIA/BMPRII/Noggin. Carbohydr Polym 2023;310:120725.**

**[3] Potterat O. Goji (Lycium barbarum and L. chinense): Phytochemistry, pharmacology and safety in the perspective of traditional uses and recent popularity. Planta Med 2010;76:7-19.**

**[4] Zhang XJ, Yu HY, Cai YJ, Ke M. Lycium barbarum polysaccharides inhibit proliferation and migration of bladder cancer cell lines BIU87 by suppressing Pi3K/AKT pathway. Oncotarget 2017;8:5936-42.**

**[5] Tang L, Bao S, Du Y, Jiang Z, Wuliji AO, Ren X, et al. Antioxidant effects of Lycium barbarum polysaccharides on photoreceptor degeneration in the light-exposed mouse retina. Biomed Pharmacother 2018;103:829-37.**

**[6] Zhang W, Zhang J, Ding D, Zhang L, Muehlmann LA, Deng SE, et al. Synthesis and antioxidant properties of Lycium barbarum polysaccharides capped selenium nanoparticles using tea extract. Artif Cells Nanomed Biotechnol 2018;46:1463-70.**

**[7] Ma K, Wang X, Feng S, Xia X, Zhang H, Rahaman A, et al. From the perspective of Traditional Chinese Medicine: Treatment of mental disorders in COVID-19 survivors. Biomed Pharmacother 2020;132:110810.**

**[8] Po KK, Leung JW, Chan JN, Fung TK, Sánchez-Vidaña DI, Sin EL, et al. Protective effect of Lycium Barbarum polysaccharides on dextromethorphan-induced mood impairment and neurogenesis suppression. Brain Res Bull 2017;134:10-7.**

**[9] Fang S, Dong L, Liu L, Guo J, Zhao L, Zhang J, et al. HERB: a high-throughput experiment- and reference-guided database of traditional Chinese medicine. Nucleic Acids Res 2021;49:D1197-d206.**

**[10] Zhang S, He F, Chen X, Ding K. Isolation and structural characterization of a pectin from Lycium ruthenicum Murr and its anti-pancreatic ductal adenocarcinoma cell activity. Carbohydr Polym 2019;223:115104.**

**[11] Cao YL, Li YL, Fan YF, Li Z, Yoshida K, Wang JY, et al. Wolfberry genomes and the evolution of Lycium (Solanaceae). Commun Biol 2021;4:671.**

**[12] Chen J, Liu X, Zhu L, Wang Y. Nuclear genome size estimation and karyotype analysis of Lycium species (Solanaceae). Scientia Horticulturae 2013;151:46-50.**

**[13] Giri MK, Swain S, Gautam JK, Singh S, Singh N, Bhattacharjee L, et al. The Arabidopsis thaliana At4g13040 gene, a unique member of the AP2/EREBP family, is a positive regulator for salicylic acid accumulation and basal defense against bacterial pathogens. J Plant Physiol 2014;171:860-7.**

**[14] Michaels SD, Ditta G, Gustafson-Brown C, Pelaz S, Yanofsky M, Amasino RM. AGL24 acts as a promoter of flowering in Arabidopsis and is positively regulated by vernalization. Plant J 2003;33:867-74.**

**[15] Schauser L, Roussis A, Stiller J, Stougaard J. A plant regulator controlling development of symbiotic root nodules. Nature 1999;402:191-5.**

**[16] Hou X, Zhou J, Liu C, Liu L, Shen L, Yu H. Nuclear factor Y-mediated H3K27me3 demethylation of the SOC1 locus orchestrates flowering responses of Arabidopsis. Nat Commun 2014;5:4601.**

**[17] Ru L, He Y, Zhu Z, Patrick JW, Ruan YL. Integrating Sugar Metabolism With Transport: Elevation of Endogenous Cell Wall Invertase Activity Up-Regulates SlHT2 and SlSWEET12c Expression for Early Fruit Development in Tomato. Front Genet 2020;11:592596.**

**[18] Breia R, Conde A, Badim H, Fortes AM, Gerós H, Granell A. Plant SWEETs: from sugar transport to plant-pathogen interaction and more unexpected physiological roles. Plant Physiol 2021;186:836-52.**

**[19] Chen LQ, Hou BH, Lalonde S, Takanaga H, Hartung ML, Qu XQ, et al. Sugar transporters for intercellular exchange and nutrition of pathogens. Nature 2010;468:527-32.**

**[20] Eom JS, Chen LQ, Sosso D, Julius BT, Lin IW, Qu XQ, et al. SWEETs, transporters for intracellular and intercellular sugar translocation. Curr Opin Plant Biol 2015;25:53-62.**

**[21] Li Y, Feng S, Ma S, Sui X, Zhang Z. Spatiotemporal Expression and Substrate Specificity Analysis of the Cucumber SWEET Gene Family. Front Plant Sci 2017;8:1855.**

**[22] Patil G, Valliyodan B, Deshmukh R, Prince S, Nicander B, Zhao M, et al. Soybean (Glycine max) SWEET gene family: insights through comparative genomics, transcriptome profiling and whole genome re-sequence analysis. BMC Genomics 2015;16:520.**

**[23] Zhang W, Wang S, Yu F, Tang J, Shan X, Bao K, et al. Genome-wide characterization and expression profiling of SWEET genes in cabbage (Brassica oleracea var. capitata L.) reveal their roles in chilling and clubroot disease responses. BMC Genomics 2019;20:93.**

**[24] Feng CY, Han JX, Han XX, Jiang J. Genome-wide identification, phylogeny, and expression analysis of the SWEET gene family in tomato. Gene 2015;573:261-72.**

**[25] Manck-Götzenberger J, Requena N. Arbuscular mycorrhiza Symbiosis Induces a Major Transcriptional Reprogramming of the Potato SWEET Sugar Transporter Family. Front Plant Sci 2016;7:487.**

**[26] Ko HY, Ho LH, Neuhaus HE, Guo WJ. Transporter SlSWEET15 unloads sucrose from phloem and seed coat for fruit and seed development in tomato. Plant Physiol 2021;187:2230-45.**

**[1] Gao Y, Wei Y, Wang Y, Gao F, Chen Z. Lycium Barbarum: A Traditional Chinese Herb and A Promising Anti-Aging Agent. Aging Dis 2017;8:778-91.**

**[2] Sun C, Chen X, Yang S, Jin C, Ding K, Chen C. LBP1C-2 from Lycium barbarum alleviated age-related bone loss by targeting BMPRIA/BMPRII/Noggin. Carbohydr Polym 2023;310:120725.**

**[4] Zhang XJ, Yu HY, Cai YJ, Ke M. Lycium barbarum polysaccharides inhibit proliferation and migration of bladder cancer cell lines BIU87 by suppressing Pi3K/AKT pathway. Oncotarget 2017;8:5936-42.**

**[5] Tang L, Bao S, Du Y, Jiang Z, Wuliji AO, Ren X, et al. Antioxidant effects of Lycium barbarum polysaccharides on photoreceptor degeneration in the light-exposed mouse retina. Biomed Pharmacother 2018;103:829-37.**

**[6] Zhang W, Zhang J, Ding D, Zhang L, Muehlmann LA, Deng SE, et al. Synthesis and antioxidant properties of Lycium barbarum polysaccharides capped selenium nanoparticles using tea extract. Artif Cells Nanomed Biotechnol 2018;46:1463-70.**

**[7] Ma K, Wang X, Feng S, Xia X, Zhang H, Rahaman A, et al. From the perspective of Traditional Chinese Medicine: Treatment of mental disorders in COVID-19 survivors. Biomed Pharmacother 2020;132:110810.**

**[8] Po KK, Leung JW, Chan JN, Fung TK, Sánchez-Vidaña DI, Sin EL, et al. Protective effect of Lycium Barbarum polysaccharides on dextromethorphan-induced mood impairment and neurogenesis suppression. Brain Res Bull 2017;134:10-7.**

**[9] Fang S, Dong L, Liu L, Guo J, Zhao L, Zhang J, et al. HERB: a high-throughput experiment- and reference-guided database of traditional Chinese medicine. Nucleic Acids Res 2021;49:D1197-d206.**

**[10] Zhang S, He F, Chen X, Ding K. Isolation and structural characterization of a pectin from Lycium ruthenicum Murr and its anti-pancreatic ductal adenocarcinoma cell activity. Carbohydr Polym 2019;223:115104.**

**[11] Cao YL, Li YL, Fan YF, Li Z, Yoshida K, Wang JY, et al. Wolfberry genomes and the evolution of Lycium (Solanaceae). Commun Biol 2021;4:671.**

**[12] Chen J, Liu X, Zhu L, Wang Y. Nuclear genome size estimation and karyotype analysis of Lycium species (Solanaceae). Scientia Horticulturae 2013;151:46-50.**

**[13] Giri MK, Swain S, Gautam JK, Singh S, Singh N, Bhattacharjee L, et al. The Arabidopsis thaliana At4g13040 gene, a unique member of the AP2/EREBP family, is a positive regulator for salicylic acid accumulation and basal defense against bacterial pathogens. J Plant Physiol 2014;171:860-7.**

**[14] Michaels SD, Ditta G, Gustafson-Brown C, Pelaz S, Yanofsky M, Amasino RM. AGL24 acts as a promoter of flowering in Arabidopsis and is positively regulated by vernalization. Plant J 2003;33:867-74.**

**[15] Schauser L, Roussis A, Stiller J, Stougaard J. A plant regulator controlling development of symbiotic root nodules. Nature 1999;402:191-5.**

**[16] Hou X, Zhou J, Liu C, Liu L, Shen L, Yu H. Nuclear factor Y-mediated H3K27me3 demethylation of the SOC1 locus orchestrates flowering responses of Arabidopsis. Nat Commun 2014;5:4601.**

**[17] Ru L, He Y, Zhu Z, Patrick JW, Ruan YL. Integrating Sugar Metabolism With Transport: Elevation of Endogenous Cell Wall Invertase Activity Up-Regulates SlHT2 and SlSWEET12c Expression for Early Fruit Development in Tomato. Front Genet 2020;11:592596.**

**[18] Breia R, Conde A, Badim H, Fortes AM, Gerós H, Granell A. Plant SWEETs: from sugar transport to plant-pathogen interaction and more unexpected physiological roles. Plant Physiol 2021;186:836-52.**

**[19] Chen LQ, Hou BH, Lalonde S, Takanaga H, Hartung ML, Qu XQ, et al. Sugar transporters for intercellular exchange and nutrition of pathogens. Nature 2010;468:527-32.**

**[20] Eom JS, Chen LQ, Sosso D, Julius BT, Lin IW, Qu XQ, et al. SWEETs, transporters for intracellular and intercellular sugar translocation. Curr Opin Plant Biol 2015;25:53-62.**

**[21] Li Y, Feng S, Ma S, Sui X, Zhang Z. Spatiotemporal Expression and Substrate Specificity Analysis of the Cucumber SWEET Gene Family. Front Plant Sci 2017;8:1855.**

**[22] Patil G, Valliyodan B, Deshmukh R, Prince S, Nicander B, Zhao M, et al. Soybean (Glycine max) SWEET gene family: insights through comparative genomics, transcriptome profiling and whole genome re-sequence analysis. BMC Genomics 2015;16:520.**

**[23] Zhang W, Wang S, Yu F, Tang J, Shan X, Bao K, et al. Genome-wide characterization and expression profiling of SWEET genes in cabbage (Brassica oleracea var. capitata L.) reveal their roles in chilling and clubroot disease responses. BMC Genomics 2019;20:93.**

**[24] Feng CY, Han JX, Han XX, Jiang J. Genome-wide identification, phylogeny, and expression analysis of the SWEET gene family in tomato. Gene 2015;573:261-72.**

**[25] Manck-Götzenberger J, Requena N. Arbuscular mycorrhiza Symbiosis Induces a Major Transcriptional Reprogramming of the Potato SWEET Sugar Transporter Family. Front Plant Sci 2016;7:487.**

**[26] Ko HY, Ho LH, Neuhaus HE, Guo WJ. Transporter SlSWEET15 unloads sucrose from phloem and seed coat for fruit and seed development in tomato. Plant Physiol 2021;187:2230-45.**

**[27] Zhang X, Feng C, Wang M, Li T, Liu X, Jiang J. Plasma membrane-localized SlSWEET7a and SlSWEET14 regulate sugar transport and storage in tomato fruits. Hortic Res 2021;8:186.**

**[1] Gao Y, Wei Y, Wang Y, Gao F, Chen Z. Lycium Barbarum: A Traditional Chinese Herb and A Promising Anti-Aging Agent. Aging Dis 2017;8:778-91.**

**[2] Sun C, Chen X, Yang S, Jin C, Ding K, Chen C. LBP1C-2 from Lycium barbarum alleviated age-related bone loss by targeting BMPRIA/BMPRII/Noggin. Carbohydr Polym 2023;310:120725.**

**[3] Potterat O. Goji (Lycium barbarum and L. chinense): Phytochemistry, pharmacology and safety in the perspective of traditional uses and recent popularity. Planta Med 2010;76:7-19.**

**[4] Zhang XJ, Yu HY, Cai YJ, Ke M. Lycium barbarum polysaccharides inhibit proliferation and migration of bladder cancer cell lines BIU87 by suppressing Pi3K/AKT pathway. Oncotarget 2017;8:5936-42.**

**[5] Tang L, Bao S, Du Y, Jiang Z, Wuliji AO, Ren X, et al. Antioxidant effects of Lycium barbarum polysaccharides on photoreceptor degeneration in the light-exposed mouse retina. Biomed Pharmacother 2018;103:829-37.**

**[6] Zhang W, Zhang J, Ding D, Zhang L, Muehlmann LA, Deng SE, et al. Synthesis and antioxidant properties of Lycium barbarum polysaccharides capped selenium nanoparticles using tea extract. Artif Cells Nanomed Biotechnol 2018;46:1463-70.**

**[7] Ma K, Wang X, Feng S, Xia X, Zhang H, Rahaman A, et al. From the perspective of Traditional Chinese Medicine: Treatment of mental disorders in COVID-19 survivors. Biomed Pharmacother 2020;132:110810.**

**[8] Po KK, Leung JW, Chan JN, Fung TK, Sánchez-Vidaña DI, Sin EL, et al. Protective effect of Lycium Barbarum polysaccharides on dextromethorphan-induced mood impairment and neurogenesis suppression. Brain Res Bull 2017;134:10-7.**

**[9] Fang S, Dong L, Liu L, Guo J, Zhao L, Zhang J, et al. HERB: a high-throughput experiment- and reference-guided database of traditional Chinese medicine. Nucleic Acids Res 2021;49:D1197-d206.**

**[10] Zhang S, He F, Chen X, Ding K. Isolation and structural characterization of a pectin from Lycium ruthenicum Murr and its anti-pancreatic ductal adenocarcinoma cell activity. Carbohydr Polym 2019;223:115104.**

**[11] Cao YL, Li YL, Fan YF, Li Z, Yoshida K, Wang JY, et al. Wolfberry genomes and the evolution of Lycium (Solanaceae). Commun Biol 2021;4:671.**

**[12] Chen J, Liu X, Zhu L, Wang Y. Nuclear genome size estimation and karyotype analysis of Lycium species (Solanaceae). Scientia Horticulturae 2013;151:46-50.**

**[13] Giri MK, Swain S, Gautam JK, Singh S, Singh N, Bhattacharjee L, et al. The Arabidopsis thaliana At4g13040 gene, a unique member of the AP2/EREBP family, is a positive regulator for salicylic acid accumulation and basal defense against bacterial pathogens. J Plant Physiol 2014;171:860-7.**

**[14] Michaels SD, Ditta G, Gustafson-Brown C, Pelaz S, Yanofsky M, Amasino RM. AGL24 acts as a promoter of flowering in Arabidopsis and is positively regulated by vernalization. Plant J 2003;33:867-74.**

**[15] Schauser L, Roussis A, Stiller J, Stougaard J. A plant regulator controlling development of symbiotic root nodules. Nature 1999;402:191-5.**

**[16] Hou X, Zhou J, Liu C, Liu L, Shen L, Yu H. Nuclear factor Y-mediated H3K27me3 demethylation of the SOC1 locus orchestrates flowering responses of Arabidopsis. Nat Commun 2014;5:4601.**

**[17] Ru L, He Y, Zhu Z, Patrick JW, Ruan YL. Integrating Sugar Metabolism With Transport: Elevation of Endogenous Cell Wall Invertase Activity Up-Regulates SlHT2 and SlSWEET12c Expression for Early Fruit Development in Tomato. Front Genet 2020;11:592596.**

**[18] Breia R, Conde A, Badim H, Fortes AM, Gerós H, Granell A. Plant SWEETs: from sugar transport to plant-pathogen interaction and more unexpected physiological roles. Plant Physiol 2021;186:836-52.**

**[19] Chen LQ, Hou BH, Lalonde S, Takanaga H, Hartung ML, Qu XQ, et al. Sugar transporters for intercellular exchange and nutrition of pathogens. Nature 2010;468:527-32.**

**[20] Eom JS, Chen LQ, Sosso D, Julius BT, Lin IW, Qu XQ, et al. SWEETs, transporters for intracellular and intercellular sugar translocation. Curr Opin Plant Biol 2015;25:53-62.**

**[21] Li Y, Feng S, Ma S, Sui X, Zhang Z. Spatiotemporal Expression and Substrate Specificity Analysis of the Cucumber SWEET Gene Family. Front Plant Sci 2017;8:1855.**

**[22] Patil G, Valliyodan B, Deshmukh R, Prince S, Nicander B, Zhao M, et al. Soybean (Glycine max) SWEET gene family: insights through comparative genomics, transcriptome profiling and whole genome re-sequence analysis. BMC Genomics 2015;16:520.**

**[23] Zhang W, Wang S, Yu F, Tang J, Shan X, Bao K, et al. Genome-wide characterization and expression profiling of SWEET genes in cabbage (Brassica oleracea var. capitata L.) reveal their roles in chilling and clubroot disease responses. BMC Genomics 2019;20:93.**

**[24] Feng CY, Han JX, Han XX, Jiang J. Genome-wide identification, phylogeny, and expression analysis of the SWEET gene family in tomato. Gene 2015;573:261-72.**

**[25] Manck-Götzenberger J, Requena N. Arbuscular mycorrhiza Symbiosis Induces a Major Transcriptional Reprogramming of the Potato SWEET Sugar Transporter Family. Front Plant Sci 2016;7:487.**

**[26] Ko HY, Ho LH, Neuhaus HE, Guo WJ. Transporter SlSWEET15 unloads sucrose from phloem and seed coat for fruit and seed development in tomato. Plant Physiol 2021;187:2230-45.**

**[27] Zhang X, Feng C, Wang M, Li T, Liu X, Jiang J. Plasma membrane-localized SlSWEET7a and SlSWEET14 regulate sugar transport and storage in tomato fruits. Hortic Res 2021;8:186.**

**[28] Pan L, Guo Q, Chai S, Cheng Y, Ruan M, Ye Q, et al. Evolutionary Conservation and Expression Patterns of Neutral/Alkaline Invertases in Solanum. Biomolecules 2019;9.**

**[1] Gao Y, Wei Y, Wang Y, Gao F, Chen Z. Lycium Barbarum: A Traditional Chinese Herb and A Promising Anti-Aging Agent. Aging Dis 2017;8:778-91.**

**[2] Sun C, Chen X, Yang S, Jin C, Ding K, Chen C. LBP1C-2 from Lycium barbarum alleviated age-related bone loss by targeting BMPRIA/BMPRII/Noggin. Carbohydr Polym 2023;310:120725.**

**[3] Potterat O. Goji (Lycium barbarum and L. chinense): Phytochemistry, pharmacology and safety in the perspective of traditional uses and recent popularity. Planta Med 2010;76:7-19.**

**[4] Zhang XJ, Yu HY, Cai YJ, Ke M. Lycium barbarum polysaccharides inhibit proliferation and migration of bladder cancer cell lines BIU87 by suppressing Pi3K/AKT pathway. Oncotarget 2017;8:5936-42.**

**[5] Tang L, Bao S, Du Y, Jiang Z, Wuliji AO, Ren X, et al. Antioxidant effects of Lycium barbarum polysaccharides on photoreceptor degeneration in the light-exposed mouse retina. Biomed Pharmacother 2018;103:829-37.**

**[6] Zhang W, Zhang J, Ding D, Zhang L, Muehlmann LA, Deng SE, et al. Synthesis and antioxidant properties of Lycium barbarum polysaccharides capped selenium nanoparticles using tea extract. Artif Cells Nanomed Biotechnol 2018;46:1463-70.**

**[7] Ma K, Wang X, Feng S, Xia X, Zhang H, Rahaman A, et al. From the perspective of Traditional Chinese Medicine: Treatment of mental disorders in COVID-19 survivors. Biomed Pharmacother 2020;132:110810.**

**[8] Po KK, Leung JW, Chan JN, Fung TK, Sánchez-Vidaña DI, Sin EL, et al. Protective effect of Lycium Barbarum polysaccharides on dextromethorphan-induced mood impairment and neurogenesis suppression. Brain Res Bull 2017;134:10-7.**

**[9] Fang S, Dong L, Liu L, Guo J, Zhao L, Zhang J, et al. HERB: a high-throughput experiment- and reference-guided database of traditional Chinese medicine. Nucleic Acids Res 2021;49:D1197-d206.**

**[10] Zhang S, He F, Chen X, Ding K. Isolation and structural characterization of a pectin from Lycium ruthenicum Murr and its anti-pancreatic ductal adenocarcinoma cell activity. Carbohydr Polym 2019;223:115104.**

**[11] Cao YL, Li YL, Fan YF, Li Z, Yoshida K, Wang JY, et al. Wolfberry genomes and the evolution of Lycium (Solanaceae). Commun Biol 2021;4:671.**

**[12] Chen J, Liu X, Zhu L, Wang Y. Nuclear genome size estimation and karyotype analysis of Lycium species (Solanaceae). Scientia Horticulturae 2013;151:46-50.**

**[13] Giri MK, Swain S, Gautam JK, Singh S, Singh N, Bhattacharjee L, et al. The Arabidopsis thaliana At4g13040 gene, a unique member of the AP2/EREBP family, is a positive regulator for salicylic acid accumulation and basal defense against bacterial pathogens. J Plant Physiol 2014;171:860-7.**

**[14] Michaels SD, Ditta G, Gustafson-Brown C, Pelaz S, Yanofsky M, Amasino RM. AGL24 acts as a promoter of flowering in Arabidopsis and is positively regulated by vernalization. Plant J 2003;33:867-74.**

**[15] Schauser L, Roussis A, Stiller J, Stougaard J. A plant regulator controlling development of symbiotic root nodules. Nature 1999;402:191-5.**

**[16] Hou X, Zhou J, Liu C, Liu L, Shen L, Yu H. Nuclear factor Y-mediated H3K27me3 demethylation of the SOC1 locus orchestrates flowering responses of Arabidopsis. Nat Commun 2014;5:4601.**

**[17] Ru L, He Y, Zhu Z, Patrick JW, Ruan YL. Integrating Sugar Metabolism With Transport: Elevation of Endogenous Cell Wall Invertase Activity Up-Regulates SlHT2 and SlSWEET12c Expression for Early Fruit Development in Tomato. Front Genet 2020;11:592596.**

**[18] Breia R, Conde A, Badim H, Fortes AM, Gerós H, Granell A. Plant SWEETs: from sugar transport to plant-pathogen interaction and more unexpected physiological roles. Plant Physiol 2021;186:836-52.**

**[19] Chen LQ, Hou BH, Lalonde S, Takanaga H, Hartung ML, Qu XQ, et al. Sugar transporters for intercellular exchange and nutrition of pathogens. Nature 2010;468:527-32.**

**[20] Eom JS, Chen LQ, Sosso D, Julius BT, Lin IW, Qu XQ, et al. SWEETs, transporters for intracellular and intercellular sugar translocation. Curr Opin Plant Biol 2015;25:53-62.**

**[21] Li Y, Feng S, Ma S, Sui X, Zhang Z. Spatiotemporal Expression and Substrate Specificity Analysis of the Cucumber SWEET Gene Family. Front Plant Sci 2017;8:1855.**

**[22] Patil G, Valliyodan B, Deshmukh R, Prince S, Nicander B, Zhao M, et al. Soybean (Glycine max) SWEET gene family: insights through comparative genomics, transcriptome profiling and whole genome re-sequence analysis. BMC Genomics 2015;16:520.**

**[23] Zhang W, Wang S, Yu F, Tang J, Shan X, Bao K, et al. Genome-wide characterization and expression profiling of SWEET genes in cabbage (Brassica oleracea var. capitata L.) reveal their roles in chilling and clubroot disease responses. BMC Genomics 2019;20:93.**

**[24] Feng CY, Han JX, Han XX, Jiang J. Genome-wide identification, phylogeny, and expression analysis of the SWEET gene family in tomato. Gene 2015;573:261-72.**

**[26] Ko HY, Ho LH, Neuhaus HE, Guo WJ. Transporter SlSWEET15 unloads sucrose from phloem and seed coat for fruit and seed development in tomato. Plant Physiol 2021;187:2230-45.**

**[27] Zhang X, Feng C, Wang M, Li T, Liu X, Jiang J. Plasma membrane-localized SlSWEET7a and SlSWEET14 regulate sugar transport and storage in tomato fruits. Hortic Res 2021;8:186.**

**[28] Pan L, Guo Q, Chai S, Cheng Y, Ruan M, Ye Q, et al. Evolutionary Conservation and Expression Patterns of Neutral/Alkaline Invertases in Solanum. Biomolecules 2019;9.**

**[29] Veillet F, Gaillard C, Coutos-Thévenot P, La Camera S. Targeting the AtCWIN1 Gene to Explore the Role of Invertases in Sucrose Transport in Roots and during Botrytis cinerea Infection. Front Plant Sci 2016;7:1899.**

**[1] Gao Y, Wei Y, Wang Y, Gao F, Chen Z. Lycium Barbarum: A Traditional Chinese Herb and A Promising Anti-Aging Agent. Aging Dis 2017;8:778-91.**

**[2] Sun C, Chen X, Yang S, Jin C, Ding K, Chen C. LBP1C-2 from Lycium barbarum alleviated age-related bone loss by targeting BMPRIA/BMPRII/Noggin. Carbohydr Polym 2023;310:120725.**

**[3] Potterat O. Goji (Lycium barbarum and L. chinense): Phytochemistry, pharmacology and safety in the perspective of traditional uses and recent popularity. Planta Med 2010;76:7-19.**

**[4] Zhang XJ, Yu HY, Cai YJ, Ke M. Lycium barbarum polysaccharides inhibit proliferation and migration of bladder cancer cell lines BIU87 by suppressing Pi3K/AKT pathway. Oncotarget 2017;8:5936-42.**

**[5] Tang L, Bao S, Du Y, Jiang Z, Wuliji AO, Ren X, et al. Antioxidant effects of Lycium barbarum polysaccharides on photoreceptor degeneration in the light-exposed mouse retina. Biomed Pharmacother 2018;103:829-37.**

**[6] Zhang W, Zhang J, Ding D, Zhang L, Muehlmann LA, Deng SE, et al. Synthesis and antioxidant properties of Lycium barbarum polysaccharides capped selenium nanoparticles using tea extract. Artif Cells Nanomed Biotechnol 2018;46:1463-70.**

**[7] Ma K, Wang X, Feng S, Xia X, Zhang H, Rahaman A, et al. From the perspective of Traditional Chinese Medicine: Treatment of mental disorders in COVID-19 survivors. Biomed Pharmacother 2020;132:110810.**

**[8] Po KK, Leung JW, Chan JN, Fung TK, Sánchez-Vidaña DI, Sin EL, et al. Protective effect of Lycium Barbarum polysaccharides on dextromethorphan-induced mood impairment and neurogenesis suppression. Brain Res Bull 2017;134:10-7.**

**[9] Fang S, Dong L, Liu L, Guo J, Zhao L, Zhang J, et al. HERB: a high-throughput experiment- and reference-guided database of traditional Chinese medicine. Nucleic Acids Res 2021;49:D1197-d206.**

**[10] Zhang S, He F, Chen X, Ding K. Isolation and structural characterization of a pectin from Lycium ruthenicum Murr and its anti-pancreatic ductal adenocarcinoma cell activity. Carbohydr Polym 2019;223:115104.**

**[11] Cao YL, Li YL, Fan YF, Li Z, Yoshida K, Wang JY, et al. Wolfberry genomes and the evolution of Lycium (Solanaceae). Commun Biol 2021;4:671.**

**[12] Chen J, Liu X, Zhu L, Wang Y. Nuclear genome size estimation and karyotype analysis of Lycium species (Solanaceae). Scientia Horticulturae 2013;151:46-50.**

**[13] Giri MK, Swain S, Gautam JK, Singh S, Singh N, Bhattacharjee L, et al. The Arabidopsis thaliana At4g13040 gene, a unique member of the AP2/EREBP family, is a positive regulator for salicylic acid accumulation and basal defense against bacterial pathogens. J Plant Physiol 2014;171:860-7.**

**[14] Michaels SD, Ditta G, Gustafson-Brown C, Pelaz S, Yanofsky M, Amasino RM. AGL24 acts as a promoter of flowering in Arabidopsis and is positively regulated by vernalization. Plant J 2003;33:867-74.**

**[15] Schauser L, Roussis A, Stiller J, Stougaard J. A plant regulator controlling development of symbiotic root nodules. Nature 1999;402:191-5.**

**[16] Hou X, Zhou J, Liu C, Liu L, Shen L, Yu H. Nuclear factor Y-mediated H3K27me3 demethylation of the SOC1 locus orchestrates flowering responses of Arabidopsis. Nat Commun 2014;5:4601.**

**[17] Ru L, He Y, Zhu Z, Patrick JW, Ruan YL. Integrating Sugar Metabolism With Transport: Elevation of Endogenous Cell Wall Invertase Activity Up-Regulates SlHT2 and SlSWEET12c Expression for Early Fruit Development in Tomato. Front Genet 2020;11:592596.**

**[18] Breia R, Conde A, Badim H, Fortes AM, Gerós H, Granell A. Plant SWEETs: from sugar transport to plant-pathogen interaction and more unexpected physiological roles. Plant Physiol 2021;186:836-52.**

**[19] Chen LQ, Hou BH, Lalonde S, Takanaga H, Hartung ML, Qu XQ, et al. Sugar transporters for intercellular exchange and nutrition of pathogens. Nature 2010;468:527-32.**

**[20] Eom JS, Chen LQ, Sosso D, Julius BT, Lin IW, Qu XQ, et al. SWEETs, transporters for intracellular and intercellular sugar translocation. Curr Opin Plant Biol 2015;25:53-62.**

**[21] Li Y, Feng S, Ma S, Sui X, Zhang Z. Spatiotemporal Expression and Substrate Specificity Analysis of the Cucumber SWEET Gene Family. Front Plant Sci 2017;8:1855.**

**[22] Patil G, Valliyodan B, Deshmukh R, Prince S, Nicander B, Zhao M, et al. Soybean (Glycine max) SWEET gene family: insights through comparative genomics, transcriptome profiling and whole genome re-sequence analysis. BMC Genomics 2015;16:520.**

**[23] Zhang W, Wang S, Yu F, Tang J, Shan X, Bao K, et al. Genome-wide characterization and expression profiling of SWEET genes in cabbage (Brassica oleracea var. capitata L.) reveal their roles in chilling and clubroot disease responses. BMC Genomics 2019;20:93.**

**[24] Feng CY, Han JX, Han XX, Jiang J. Genome-wide identification, phylogeny, and expression analysis of the SWEET gene family in tomato. Gene 2015;573:261-72.**

**[25] Manck-Götzenberger J, Requena N. Arbuscular mycorrhiza Symbiosis Induces a Major Transcriptional Reprogramming of the Potato SWEET Sugar Transporter Family. Front Plant Sci 2016;7:487.**

**[26] Ko HY, Ho LH, Neuhaus HE, Guo WJ. Transporter SlSWEET15 unloads sucrose from phloem and seed coat for fruit and seed development in tomato. Plant Physiol 2021;187:2230-45.**

**[27] Zhang X, Feng C, Wang M, Li T, Liu X, Jiang J. Plasma membrane-localized SlSWEET7a and SlSWEET14 regulate sugar transport and storage in tomato fruits. Hortic Res 2021;8:186.**

**[28] Pan L, Guo Q, Chai S, Cheng Y, Ruan M, Ye Q, et al. Evolutionary Conservation and Expression Patterns of Neutral/Alkaline Invertases in Solanum. Biomolecules 2019;9.**

**[29] Veillet F, Gaillard C, Coutos-Thévenot P, La Camera S. Targeting the AtCWIN1 Gene to Explore the Role of Invertases in Sucrose Transport in Roots and during Botrytis cinerea Infection. Front Plant Sci 2016;7:1899.**

**[30] Wan H, Wu L, Yang Y, Zhou G, Ruan YL. Evolution of Sucrose Metabolism: The Dichotomy of Invertases and Beyond. Trends Plant Sci 2018;23:163-77.**

**[1] Gao Y, Wei Y, Wang Y, Gao F, Chen Z. Lycium Barbarum: A Traditional Chinese Herb and A Promising Anti-Aging Agent. Aging Dis 2017;8:778-91.**

**[2] Sun C, Chen X, Yang S, Jin C, Ding K, Chen C. LBP1C-2 from Lycium barbarum alleviated age-related bone loss by targeting BMPRIA/BMPRII/Noggin. Carbohydr Polym 2023;310:120725.**

**[3] Potterat O. Goji (Lycium barbarum and L. chinense): Phytochemistry, pharmacology and safety in the perspective of traditional uses and recent popularity. Planta Med 2010;76:7-19.**

**[4] Zhang XJ, Yu HY, Cai YJ, Ke M. Lycium barbarum polysaccharides inhibit proliferation and migration of bladder cancer cell lines BIU87 by suppressing Pi3K/AKT pathway. Oncotarget 2017;8:5936-42.**

**[5] Tang L, Bao S, Du Y, Jiang Z, Wuliji AO, Ren X, et al. Antioxidant effects of Lycium barbarum polysaccharides on photoreceptor degeneration in the light-exposed mouse retina. Biomed Pharmacother 2018;103:829-37.**

**[6] Zhang W, Zhang J, Ding D, Zhang L, Muehlmann LA, Deng SE, et al. Synthesis and antioxidant properties of Lycium barbarum polysaccharides capped selenium nanoparticles using tea extract. Artif Cells Nanomed Biotechnol 2018;46:1463-70.**

**[7] Ma K, Wang X, Feng S, Xia X, Zhang H, Rahaman A, et al. From the perspective of Traditional Chinese Medicine: Treatment of mental disorders in COVID-19 survivors. Biomed Pharmacother 2020;132:110810.**

**[8] Po KK, Leung JW, Chan JN, Fung TK, Sánchez-Vidaña DI, Sin EL, et al. Protective effect of Lycium Barbarum polysaccharides on dextromethorphan-induced mood impairment and neurogenesis suppression. Brain Res Bull 2017;134:10-7.**

**[9] Fang S, Dong L, Liu L, Guo J, Zhao L, Zhang J, et al. HERB: a high-throughput experiment- and reference-guided database of traditional Chinese medicine. Nucleic Acids Res 2021;49:D1197-d206.**

**[10] Zhang S, He F, Chen X, Ding K. Isolation and structural characterization of a pectin from Lycium ruthenicum Murr and its anti-pancreatic ductal adenocarcinoma cell activity. Carbohydr Polym 2019;223:115104.**

**[11] Cao YL, Li YL, Fan YF, Li Z, Yoshida K, Wang JY, et al. Wolfberry genomes and the evolution of Lycium (Solanaceae). Commun Biol 2021;4:671.**

**[12] Chen J, Liu X, Zhu L, Wang Y. Nuclear genome size estimation and karyotype analysis of Lycium species (Solanaceae). Scientia Horticulturae 2013;151:46-50.**

**[13] Giri MK, Swain S, Gautam JK, Singh S, Singh N, Bhattacharjee L, et al. The Arabidopsis thaliana At4g13040 gene, a unique member of the AP2/EREBP family, is a positive regulator for salicylic acid accumulation and basal defense against bacterial pathogens. J Plant Physiol 2014;171:860-7.**

**[14] Michaels SD, Ditta G, Gustafson-Brown C, Pelaz S, Yanofsky M, Amasino RM. AGL24 acts as a promoter of flowering in Arabidopsis and is positively regulated by vernalization. Plant J 2003;33:867-74.**

**[15] Schauser L, Roussis A, Stiller J, Stougaard J. A plant regulator controlling development of symbiotic root nodules. Nature 1999;402:191-5.**

**[16] Hou X, Zhou J, Liu C, Liu L, Shen L, Yu H. Nuclear factor Y-mediated H3K27me3 demethylation of the SOC1 locus orchestrates flowering responses of Arabidopsis. Nat Commun 2014;5:4601.**

**[17] Ru L, He Y, Zhu Z, Patrick JW, Ruan YL. Integrating Sugar Metabolism With Transport: Elevation of Endogenous Cell Wall Invertase Activity Up-Regulates SlHT2 and SlSWEET12c Expression for Early Fruit Development in Tomato. Front Genet 2020;11:592596.**

**[18] Breia R, Conde A, Badim H, Fortes AM, Gerós H, Granell A. Plant SWEETs: from sugar transport to plant-pathogen interaction and more unexpected physiological roles. Plant Physiol 2021;186:836-52.**

**[19] Chen LQ, Hou BH, Lalonde S, Takanaga H, Hartung ML, Qu XQ, et al. Sugar transporters for intercellular exchange and nutrition of pathogens. Nature 2010;468:527-32.**

**[20] Eom JS, Chen LQ, Sosso D, Julius BT, Lin IW, Qu XQ, et al. SWEETs, transporters for intracellular and intercellular sugar translocation. Curr Opin Plant Biol 2015;25:53-62.**

**[21] Li Y, Feng S, Ma S, Sui X, Zhang Z. Spatiotemporal Expression and Substrate Specificity Analysis of the Cucumber SWEET Gene Family. Front Plant Sci 2017;8:1855.**

**[22] Patil G, Valliyodan B, Deshmukh R, Prince S, Nicander B, Zhao M, et al. Soybean (Glycine max) SWEET gene family: insights through comparative genomics, transcriptome profiling and whole genome re-sequence analysis. BMC Genomics 2015;16:520.**

**[23] Zhang W, Wang S, Yu F, Tang J, Shan X, Bao K, et al. Genome-wide characterization and expression profiling of SWEET genes in cabbage (Brassica oleracea var. capitata L.) reveal their roles in chilling and clubroot disease responses. BMC Genomics 2019;20:93.**

**[24] Feng CY, Han JX, Han XX, Jiang J. Genome-wide identification, phylogeny, and expression analysis of the SWEET gene family in tomato. Gene 2015;573:261-72.**

**[25] Manck-Götzenberger J, Requena N. Arbuscular mycorrhiza Symbiosis Induces a Major Transcriptional Reprogramming of the Potato SWEET Sugar Transporter Family. Front Plant Sci 2016;7:487.**

**[26] Ko HY, Ho LH, Neuhaus HE, Guo WJ. Transporter SlSWEET15 unloads sucrose from phloem and seed coat for fruit and seed development in tomato. Plant Physiol 2021;187:2230-45.**

**[27] Zhang X, Feng C, Wang M, Li T, Liu X, Jiang J. Plasma membrane-localized SlSWEET7a and SlSWEET14 regulate sugar transport and storage in tomato fruits. Hortic Res 2021;8:186.**

**[28] Pan L, Guo Q, Chai S, Cheng Y, Ruan M, Ye Q, et al. Evolutionary Conservation and Expression Patterns of Neutral/Alkaline Invertases in Solanum. Biomolecules 2019;9.**

**[29] Veillet F, Gaillard C, Coutos-Thévenot P, La Camera S. Targeting the AtCWIN1 Gene to Explore the Role of Invertases in Sucrose Transport in Roots and during Botrytis cinerea Infection. Front Plant Sci 2016;7:1899.**

**[30] Wan H, Wu L, Yang Y, Zhou G, Ruan YL. Evolution of Sucrose Metabolism: The Dichotomy of Invertases and Beyond. Trends Plant Sci 2018;23:163-77.**

**[1] Gao Y, Wei Y, Wang Y, Gao F, Chen Z. Lycium Barbarum: A Traditional Chinese Herb and A Promising Anti-Aging Agent. Aging Dis 2017;8:778-91.**

**[2] Sun C, Chen X, Yang S, Jin C, Ding K, Chen C. LBP1C-2 from Lycium barbarum alleviated age-related bone loss by targeting BMPRIA/BMPRII/Noggin. Carbohydr Polym 2023;310:120725.**

**[3] Potterat O. Goji (Lycium barbarum and L. chinense): Phytochemistry, pharmacology and safety in the perspective of traditional uses and recent popularity. Planta Med 2010;76:7-19.**

**[4] Zhang XJ, Yu HY, Cai YJ, Ke M. Lycium barbarum polysaccharides inhibit proliferation and migration of bladder cancer cell lines BIU87 by suppressing Pi3K/AKT pathway. Oncotarget 2017;8:5936-42.**

**[5] Tang L, Bao S, Du Y, Jiang Z, Wuliji AO, Ren X, et al. Antioxidant effects of Lycium barbarum polysaccharides on photoreceptor degeneration in the light-exposed mouse retina. Biomed Pharmacother 2018;103:829-37.**

**[6] Zhang W, Zhang J, Ding D, Zhang L, Muehlmann LA, Deng SE, et al. Synthesis and antioxidant properties of Lycium barbarum polysaccharides capped selenium nanoparticles using tea extract. Artif Cells Nanomed Biotechnol 2018;46:1463-70.**

**[7] Ma K, Wang X, Feng S, Xia X, Zhang H, Rahaman A, et al. From the perspective of Traditional Chinese Medicine: Treatment of mental disorders in COVID-19 survivors. Biomed Pharmacother 2020;132:110810.**

**[8] Po KK, Leung JW, Chan JN, Fung TK, Sánchez-Vidaña DI, Sin EL, et al. Protective effect of Lycium Barbarum polysaccharides on dextromethorphan-induced mood impairment and neurogenesis suppression. Brain Res Bull 2017;134:10-7.**

**[9] Fang S, Dong L, Liu L, Guo J, Zhao L, Zhang J, et al. HERB: a high-throughput experiment- and reference-guided database of traditional Chinese medicine. Nucleic Acids Res 2021;49:D1197-d206.**

**[10] Zhang S, He F, Chen X, Ding K. Isolation and structural characterization of a pectin from Lycium ruthenicum Murr and its anti-pancreatic ductal adenocarcinoma cell activity. Carbohydr Polym 2019;223:115104.**

**[11] Cao YL, Li YL, Fan YF, Li Z, Yoshida K, Wang JY, et al. Wolfberry genomes and the evolution of Lycium (Solanaceae). Commun Biol 2021;4:671.**

**[12] Chen J, Liu X, Zhu L, Wang Y. Nuclear genome size estimation and karyotype analysis of Lycium species (Solanaceae). Scientia Horticulturae 2013;151:46-50.**

**[13] Giri MK, Swain S, Gautam JK, Singh S, Singh N, Bhattacharjee L, et al. The Arabidopsis thaliana At4g13040 gene, a unique member of the AP2/EREBP family, is a positive regulator for salicylic acid accumulation and basal defense against bacterial pathogens. J Plant Physiol 2014;171:860-7.**

**[14] Michaels SD, Ditta G, Gustafson-Brown C, Pelaz S, Yanofsky M, Amasino RM. AGL24 acts as a promoter of flowering in Arabidopsis and is positively regulated by vernalization. Plant J 2003;33:867-74.**

**[15] Schauser L, Roussis A, Stiller J, Stougaard J. A plant regulator controlling development of symbiotic root nodules. Nature 1999;402:191-5.**

**[16] Hou X, Zhou J, Liu C, Liu L, Shen L, Yu H. Nuclear factor Y-mediated H3K27me3 demethylation of the SOC1 locus orchestrates flowering responses of Arabidopsis. Nat Commun 2014;5:4601.**

**[17] Ru L, He Y, Zhu Z, Patrick JW, Ruan YL. Integrating Sugar Metabolism With Transport: Elevation of Endogenous Cell Wall Invertase Activity Up-Regulates SlHT2 and SlSWEET12c Expression for Early Fruit Development in Tomato. Front Genet 2020;11:592596.**

**[18] Breia R, Conde A, Badim H, Fortes AM, Gerós H, Granell A. Plant SWEETs: from sugar transport to plant-pathogen interaction and more unexpected physiological roles. Plant Physiol 2021;186:836-52.**

**[19] Chen LQ, Hou BH, Lalonde S, Takanaga H, Hartung ML, Qu XQ, et al. Sugar transporters for intercellular exchange and nutrition of pathogens. Nature 2010;468:527-32.**

**[20] Eom JS, Chen LQ, Sosso D, Julius BT, Lin IW, Qu XQ, et al. SWEETs, transporters for intracellular and intercellular sugar translocation. Curr Opin Plant Biol 2015;25:53-62.**

**[21] Li Y, Feng S, Ma S, Sui X, Zhang Z. Spatiotemporal Expression and Substrate Specificity Analysis of the Cucumber SWEET Gene Family. Front Plant Sci 2017;8:1855.**

**[22] Patil G, Valliyodan B, Deshmukh R, Prince S, Nicander B, Zhao M, et al. Soybean (Glycine max) SWEET gene family: insights through comparative genomics, transcriptome profiling and whole genome re-sequence analysis. BMC Genomics 2015;16:520.**

**[23] Zhang W, Wang S, Yu F, Tang J, Shan X, Bao K, et al. Genome-wide characterization and expression profiling of SWEET genes in cabbage (Brassica oleracea var. capitata L.) reveal their roles in chilling and clubroot disease responses. BMC Genomics 2019;20:93.**

**[24] Feng CY, Han JX, Han XX, Jiang J. Genome-wide identification, phylogeny, and expression analysis of the SWEET gene family in tomato. Gene 2015;573:261-72.**

**[25] Manck-Götzenberger J, Requena N. Arbuscular mycorrhiza Symbiosis Induces a Major Transcriptional Reprogramming of the Potato SWEET Sugar Transporter Family. Front Plant Sci 2016;7:487.**

**[26] Ko HY, Ho LH, Neuhaus HE, Guo WJ. Transporter SlSWEET15 unloads sucrose from phloem and seed coat for fruit and seed development in tomato. Plant Physiol 2021;187:2230-45.**

**[27] Zhang X, Feng C, Wang M, Li T, Liu X, Jiang J. Plasma membrane-localized SlSWEET7a and SlSWEET14 regulate sugar transport and storage in tomato fruits. Hortic Res 2021;8:186.**

**[28] Pan L, Guo Q, Chai S, Cheng Y, Ruan M, Ye Q, et al. Evolutionary Conservation and Expression Patterns of Neutral/Alkaline Invertases in Solanum. Biomolecules 2019;9.**

**[29] Veillet F, Gaillard C, Coutos-Thévenot P, La Camera S. Targeting the AtCWIN1 Gene to Explore the Role of Invertases in Sucrose Transport in Roots and during Botrytis cinerea Infection. Front Plant Sci 2016;7:1899.**

**[30] Wan H, Wu L, Yang Y, Zhou G, Ruan YL. Evolution of Sucrose Metabolism: The Dichotomy of Invertases and Beyond. Trends Plant Sci 2018;23:163-77.**

**[31] Jin Y, Ni DA, Ruan YL. Posttranslational elevation of cell wall invertase activity by silencing its inhibitor in tomato delays leaf senescence and increases seed weight and fruit hexose level. Plant Cell 2009;21:2072-89.**

**[1] Gao Y, Wei Y, Wang Y, Gao F, Chen Z. Lycium Barbarum: A Traditional Chinese Herb and A Promising Anti-Aging Agent. Aging Dis 2017;8:778-91.**

**[2] Sun C, Chen X, Yang S, Jin C, Ding K, Chen C. LBP1C-2 from Lycium barbarum alleviated age-related bone loss by targeting BMPRIA/BMPRII/Noggin. Carbohydr Polym 2023;310:120725.**

**[3] Potterat O. Goji (Lycium barbarum and L. chinense): Phytochemistry, pharmacology and safety in the perspective of traditional uses and recent popularity. Planta Med 2010;76:7-19.**

**[4] Zhang XJ, Yu HY, Cai YJ, Ke M. Lycium barbarum polysaccharides inhibit proliferation and migration of bladder cancer cell lines BIU87 by suppressing Pi3K/AKT pathway. Oncotarget 2017;8:5936-42.**

**[5] Tang L, Bao S, Du Y, Jiang Z, Wuliji AO, Ren X, et al. Antioxidant effects of Lycium barbarum polysaccharides on photoreceptor degeneration in the light-exposed mouse retina. Biomed Pharmacother 2018;103:829-37.**

**[6] Zhang W, Zhang J, Ding D, Zhang L, Muehlmann LA, Deng SE, et al. Synthesis and antioxidant properties of Lycium barbarum polysaccharides capped selenium nanoparticles using tea extract. Artif Cells Nanomed Biotechnol 2018;46:1463-70.**

**[7] Ma K, Wang X, Feng S, Xia X, Zhang H, Rahaman A, et al. From the perspective of Traditional Chinese Medicine: Treatment of mental disorders in COVID-19 survivors. Biomed Pharmacother 2020;132:110810.**

**[8] Po KK, Leung JW, Chan JN, Fung TK, Sánchez-Vidaña DI, Sin EL, et al. Protective effect of Lycium Barbarum polysaccharides on dextromethorphan-induced mood impairment and neurogenesis suppression. Brain Res Bull 2017;134:10-7.**

**[9] Fang S, Dong L, Liu L, Guo J, Zhao L, Zhang J, et al. HERB: a high-throughput experiment- and reference-guided database of traditional Chinese medicine. Nucleic Acids Res 2021;49:D1197-d206.**

**[10] Zhang S, He F, Chen X, Ding K. Isolation and structural characterization of a pectin from Lycium ruthenicum Murr and its anti-pancreatic ductal adenocarcinoma cell activity. Carbohydr Polym 2019;223:115104.**

**[11] Cao YL, Li YL, Fan YF, Li Z, Yoshida K, Wang JY, et al. Wolfberry genomes and the evolution of Lycium (Solanaceae). Commun Biol 2021;4:671.**

**[12] Chen J, Liu X, Zhu L, Wang Y. Nuclear genome size estimation and karyotype analysis of Lycium species (Solanaceae). Scientia Horticulturae 2013;151:46-50.**

**[13] Giri MK, Swain S, Gautam JK, Singh S, Singh N, Bhattacharjee L, et al. The Arabidopsis thaliana At4g13040 gene, a unique member of the AP2/EREBP family, is a positive regulator for salicylic acid accumulation and basal defense against bacterial pathogens. J Plant Physiol 2014;171:860-7.**

**[14] Michaels SD, Ditta G, Gustafson-Brown C, Pelaz S, Yanofsky M, Amasino RM. AGL24 acts as a promoter of flowering in Arabidopsis and is positively regulated by vernalization. Plant J 2003;33:867-74.**

**[15] Schauser L, Roussis A, Stiller J, Stougaard J. A plant regulator controlling development of symbiotic root nodules. Nature 1999;402:191-5.**

**[16] Hou X, Zhou J, Liu C, Liu L, Shen L, Yu H. Nuclear factor Y-mediated H3K27me3 demethylation of the SOC1 locus orchestrates flowering responses of Arabidopsis. Nat Commun 2014;5:4601.**

**[17] Ru L, He Y, Zhu Z, Patrick JW, Ruan YL. Integrating Sugar Metabolism With Transport: Elevation of Endogenous Cell Wall Invertase Activity Up-Regulates SlHT2 and SlSWEET12c Expression for Early Fruit Development in Tomato. Front Genet 2020;11:592596.**

**[18] Breia R, Conde A, Badim H, Fortes AM, Gerós H, Granell A. Plant SWEETs: from sugar transport to plant-pathogen interaction and more unexpected physiological roles. Plant Physiol 2021;186:836-52.**

**[19] Chen LQ, Hou BH, Lalonde S, Takanaga H, Hartung ML, Qu XQ, et al. Sugar transporters for intercellular exchange and nutrition of pathogens. Nature 2010;468:527-32.**

**[20] Eom JS, Chen LQ, Sosso D, Julius BT, Lin IW, Qu XQ, et al. SWEETs, transporters for intracellular and intercellular sugar translocation. Curr Opin Plant Biol 2015;25:53-62.**

**[21] Li Y, Feng S, Ma S, Sui X, Zhang Z. Spatiotemporal Expression and Substrate Specificity Analysis of the Cucumber SWEET Gene Family. Front Plant Sci 2017;8:1855.**

**[22] Patil G, Valliyodan B, Deshmukh R, Prince S, Nicander B, Zhao M, et al. Soybean (Glycine max) SWEET gene family: insights through comparative genomics, transcriptome profiling and whole genome re-sequence analysis. BMC Genomics 2015;16:520.**

**[23] Zhang W, Wang S, Yu F, Tang J, Shan X, Bao K, et al. Genome-wide characterization and expression profiling of SWEET genes in cabbage (Brassica oleracea var. capitata L.) reveal their roles in chilling and clubroot disease responses. BMC Genomics 2019;20:93.**

**[24] Feng CY, Han JX, Han XX, Jiang J. Genome-wide identification, phylogeny, and expression analysis of the SWEET gene family in tomato. Gene 2015;573:261-72.**

**[26] Ko HY, Ho LH, Neuhaus HE, Guo WJ. Transporter SlSWEET15 unloads sucrose from phloem and seed coat for fruit and seed development in tomato. Plant Physiol 2021;187:2230-45.**

**[27] Zhang X, Feng C, Wang M, Li T, Liu X, Jiang J. Plasma membrane-localized SlSWEET7a and SlSWEET14 regulate sugar transport and storage in tomato fruits. Hortic Res 2021;8:186.**

**[28] Pan L, Guo Q, Chai S, Cheng Y, Ruan M, Ye Q, et al. Evolutionary Conservation and Expression Patterns of Neutral/Alkaline Invertases in Solanum. Biomolecules 2019;9.**

**[29] Veillet F, Gaillard C, Coutos-Thévenot P, La Camera S. Targeting the AtCWIN1 Gene to Explore the Role of Invertases in Sucrose Transport in Roots and during Botrytis cinerea Infection. Front Plant Sci 2016;7:1899.**

**[30] Wan H, Wu L, Yang Y, Zhou G, Ruan YL. Evolution of Sucrose Metabolism: The Dichotomy of Invertases and Beyond. Trends Plant Sci 2018;23:163-77.**

**[31] Jin Y, Ni DA, Ruan YL. Posttranslational elevation of cell wall invertase activity by silencing its inhibitor in tomato delays leaf senescence and increases seed weight and fruit hexose level. Plant Cell 2009;21:2072-89.**

**[32] Shen S, Ma S, Liu Y, Liao S, Li J, Wu L, et al. Cell Wall Invertase and Sugar Transporters Are Differentially Activated in Tomato Styles and Ovaries During Pollination and Fertilization. Front Plant Sci 2019;10:506.**

**[1] Gao Y, Wei Y, Wang Y, Gao F, Chen Z. Lycium Barbarum: A Traditional Chinese Herb and A Promising Anti-Aging Agent. Aging Dis 2017;8:778-91.**

**[2] Sun C, Chen X, Yang S, Jin C, Ding K, Chen C. LBP1C-2 from Lycium barbarum alleviated age-related bone loss by targeting BMPRIA/BMPRII/Noggin. Carbohydr Polym 2023;310:120725.**

**[3] Potterat O. Goji (Lycium barbarum and L. chinense): Phytochemistry, pharmacology and safety in the perspective of traditional uses and recent popularity. Planta Med 2010;76:7-19.**

**[4] Zhang XJ, Yu HY, Cai YJ, Ke M. Lycium barbarum polysaccharides inhibit proliferation and migration of bladder cancer cell lines BIU87 by suppressing Pi3K/AKT pathway. Oncotarget 2017;8:5936-42.**

**[5] Tang L, Bao S, Du Y, Jiang Z, Wuliji AO, Ren X, et al. Antioxidant effects of Lycium barbarum polysaccharides on photoreceptor degeneration in the light-exposed mouse retina. Biomed Pharmacother 2018;103:829-37.**

**[6] Zhang W, Zhang J, Ding D, Zhang L, Muehlmann LA, Deng SE, et al. Synthesis and antioxidant properties of Lycium barbarum polysaccharides capped selenium nanoparticles using tea extract. Artif Cells Nanomed Biotechnol 2018;46:1463-70.**

**[7] Ma K, Wang X, Feng S, Xia X, Zhang H, Rahaman A, et al. From the perspective of Traditional Chinese Medicine: Treatment of mental disorders in COVID-19 survivors. Biomed Pharmacother 2020;132:110810.**

**[8] Po KK, Leung JW, Chan JN, Fung TK, Sánchez-Vidaña DI, Sin EL, et al. Protective effect of Lycium Barbarum polysaccharides on dextromethorphan-induced mood impairment and neurogenesis suppression. Brain Res Bull 2017;134:10-7.**

**[9] Fang S, Dong L, Liu L, Guo J, Zhao L, Zhang J, et al. HERB: a high-throughput experiment- and reference-guided database of traditional Chinese medicine. Nucleic Acids Res 2021;49:D1197-d206.**

**[10] Zhang S, He F, Chen X, Ding K. Isolation and structural characterization of a pectin from Lycium ruthenicum Murr and its anti-pancreatic ductal adenocarcinoma cell activity. Carbohydr Polym 2019;223:115104.**

**[12] Chen J, Liu X, Zhu L, Wang Y. Nuclear genome size estimation and karyotype analysis of Lycium species (Solanaceae). Scientia Horticulturae 2013;151:46-50.**

**[13] Giri MK, Swain S, Gautam JK, Singh S, Singh N, Bhattacharjee L, et al. The Arabidopsis thaliana At4g13040 gene, a unique member of the AP2/EREBP family, is a positive regulator for salicylic acid accumulation and basal defense against bacterial pathogens. J Plant Physiol 2014;171:860-7.**

**[14] Michaels SD, Ditta G, Gustafson-Brown C, Pelaz S, Yanofsky M, Amasino RM. AGL24 acts as a promoter of flowering in Arabidopsis and is positively regulated by vernalization. Plant J 2003;33:867-74.**

**[15] Schauser L, Roussis A, Stiller J, Stougaard J. A plant regulator controlling development of symbiotic root nodules. Nature 1999;402:191-5.**

**[16] Hou X, Zhou J, Liu C, Liu L, Shen L, Yu H. Nuclear factor Y-mediated H3K27me3 demethylation of the SOC1 locus orchestrates flowering responses of Arabidopsis. Nat Commun 2014;5:4601.**

**[17] Ru L, He Y, Zhu Z, Patrick JW, Ruan YL. Integrating Sugar Metabolism With Transport: Elevation of Endogenous Cell Wall Invertase Activity Up-Regulates SlHT2 and SlSWEET12c Expression for Early Fruit Development in Tomato. Front Genet 2020;11:592596.**

**[18] Breia R, Conde A, Badim H, Fortes AM, Gerós H, Granell A. Plant SWEETs: from sugar transport to plant-pathogen interaction and more unexpected physiological roles. Plant Physiol 2021;186:836-52.**

**[19] Chen LQ, Hou BH, Lalonde S, Takanaga H, Hartung ML, Qu XQ, et al. Sugar transporters for intercellular exchange and nutrition of pathogens. Nature 2010;468:527-32.**

**[20] Eom JS, Chen LQ, Sosso D, Julius BT, Lin IW, Qu XQ, et al. SWEETs, transporters for intracellular and intercellular sugar translocation. Curr Opin Plant Biol 2015;25:53-62.**

**[21] Li Y, Feng S, Ma S, Sui X, Zhang Z. Spatiotemporal Expression and Substrate Specificity Analysis of the Cucumber SWEET Gene Family. Front Plant Sci 2017;8:1855.**

**[22] Patil G, Valliyodan B, Deshmukh R, Prince S, Nicander B, Zhao M, et al. Soybean (Glycine max) SWEET gene family: insights through comparative genomics, transcriptome profiling and whole genome re-sequence analysis. BMC Genomics 2015;16:520.**

**[23] Zhang W, Wang S, Yu F, Tang J, Shan X, Bao K, et al. Genome-wide characterization and expression profiling of SWEET genes in cabbage (Brassica oleracea var. capitata L.) reveal their roles in chilling and clubroot disease responses. BMC Genomics 2019;20:93.**

**[24] Feng CY, Han JX, Han XX, Jiang J. Genome-wide identification, phylogeny, and expression analysis of the SWEET gene family in tomato. Gene 2015;573:261-72.**

**[25] Manck-Götzenberger J, Requena N. Arbuscular mycorrhiza Symbiosis Induces a Major Transcriptional Reprogramming of the Potato SWEET Sugar Transporter Family. Front Plant Sci 2016;7:487.**

**[26] Ko HY, Ho LH, Neuhaus HE, Guo WJ. Transporter SlSWEET15 unloads sucrose from phloem and seed coat for fruit and seed development in tomato. Plant Physiol 2021;187:2230-45.**

**[27] Zhang X, Feng C, Wang M, Li T, Liu X, Jiang J. Plasma membrane-localized SlSWEET7a and SlSWEET14 regulate sugar transport and storage in tomato fruits. Hortic Res 2021;8:186.**

**[28] Pan L, Guo Q, Chai S, Cheng Y, Ruan M, Ye Q, et al. Evolutionary Conservation and Expression Patterns of Neutral/Alkaline Invertases in Solanum. Biomolecules 2019;9.**

**[29] Veillet F, Gaillard C, Coutos-Thévenot P, La Camera S. Targeting the AtCWIN1 Gene to Explore the Role of Invertases in Sucrose Transport in Roots and during Botrytis cinerea Infection. Front Plant Sci 2016;7:1899.**

**[30] Wan H, Wu L, Yang Y, Zhou G, Ruan YL. Evolution of Sucrose Metabolism: The Dichotomy of Invertases and Beyond. Trends Plant Sci 2018;23:163-77.**

**[31] Jin Y, Ni DA, Ruan YL. Posttranslational elevation of cell wall invertase activity by silencing its inhibitor in tomato delays leaf senescence and increases seed weight and fruit hexose level. Plant Cell 2009;21:2072-89.**

**[32] Shen S, Ma S, Liu Y, Liao S, Li J, Wu L, et al. Cell Wall Invertase and Sugar Transporters Are Differentially Activated in Tomato Styles and Ovaries During Pollination and Fertilization. Front Plant Sci 2019;10:506.**

**[33] Lombard V, Golaconda Ramulu H, Drula E, Coutinho PM, Henrissat B. The carbohydrate-active enzymes database (CAZy) in 2013. Nucleic Acids Res 2014;42:D490-5.**

**[1] Gao Y, Wei Y, Wang Y, Gao F, Chen Z. Lycium Barbarum: A Traditional Chinese Herb and A Promising Anti-Aging Agent. Aging Dis 2017;8:778-91.**

**[2] Sun C, Chen X, Yang S, Jin C, Ding K, Chen C. LBP1C-2 from Lycium barbarum alleviated age-related bone loss by targeting BMPRIA/BMPRII/Noggin. Carbohydr Polym 2023;310:120725.**

**[3] Potterat O. Goji (Lycium barbarum and L. chinense): Phytochemistry, pharmacology and safety in the perspective of traditional uses and recent popularity. Planta Med 2010;76:7-19.**

**[4] Zhang XJ, Yu HY, Cai YJ, Ke M. Lycium barbarum polysaccharides inhibit proliferation and migration of bladder cancer cell lines BIU87 by suppressing Pi3K/AKT pathway. Oncotarget 2017;8:5936-42.**

**[5] Tang L, Bao S, Du Y, Jiang Z, Wuliji AO, Ren X, et al. Antioxidant effects of Lycium barbarum polysaccharides on photoreceptor degeneration in the light-exposed mouse retina. Biomed Pharmacother 2018;103:829-37.**

**[6] Zhang W, Zhang J, Ding D, Zhang L, Muehlmann LA, Deng SE, et al. Synthesis and antioxidant properties of Lycium barbarum polysaccharides capped selenium nanoparticles using tea extract. Artif Cells Nanomed Biotechnol 2018;46:1463-70.**

**[7] Ma K, Wang X, Feng S, Xia X, Zhang H, Rahaman A, et al. From the perspective of Traditional Chinese Medicine: Treatment of mental disorders in COVID-19 survivors. Biomed Pharmacother 2020;132:110810.**

**[8] Po KK, Leung JW, Chan JN, Fung TK, Sánchez-Vidaña DI, Sin EL, et al. Protective effect of Lycium Barbarum polysaccharides on dextromethorphan-induced mood impairment and neurogenesis suppression. Brain Res Bull 2017;134:10-7.**

**[9] Fang S, Dong L, Liu L, Guo J, Zhao L, Zhang J, et al. HERB: a high-throughput experiment- and reference-guided database of traditional Chinese medicine. Nucleic Acids Res 2021;49:D1197-d206.**

**[10] Zhang S, He F, Chen X, Ding K. Isolation and structural characterization of a pectin from Lycium ruthenicum Murr and its anti-pancreatic ductal adenocarcinoma cell activity. Carbohydr Polym 2019;223:115104.**

**[11] Cao YL, Li YL, Fan YF, Li Z, Yoshida K, Wang JY, et al. Wolfberry genomes and the evolution of Lycium (Solanaceae). Commun Biol 2021;4:671.**

**[12] Chen J, Liu X, Zhu L, Wang Y. Nuclear genome size estimation and karyotype analysis of Lycium species (Solanaceae). Scientia Horticulturae 2013;151:46-50.**

**[13] Giri MK, Swain S, Gautam JK, Singh S, Singh N, Bhattacharjee L, et al. The Arabidopsis thaliana At4g13040 gene, a unique member of the AP2/EREBP family, is a positive regulator for salicylic acid accumulation and basal defense against bacterial pathogens. J Plant Physiol 2014;171:860-7.**

**[14] Michaels SD, Ditta G, Gustafson-Brown C, Pelaz S, Yanofsky M, Amasino RM. AGL24 acts as a promoter of flowering in Arabidopsis and is positively regulated by vernalization. Plant J 2003;33:867-74.**

**[15] Schauser L, Roussis A, Stiller J, Stougaard J. A plant regulator controlling development of symbiotic root nodules. Nature 1999;402:191-5.**

**[16] Hou X, Zhou J, Liu C, Liu L, Shen L, Yu H. Nuclear factor Y-mediated H3K27me3 demethylation of the SOC1 locus orchestrates flowering responses of Arabidopsis. Nat Commun 2014;5:4601.**

**[17] Ru L, He Y, Zhu Z, Patrick JW, Ruan YL. Integrating Sugar Metabolism With Transport: Elevation of Endogenous Cell Wall Invertase Activity Up-Regulates SlHT2 and SlSWEET12c Expression for Early Fruit Development in Tomato. Front Genet 2020;11:592596.**

**[18] Breia R, Conde A, Badim H, Fortes AM, Gerós H, Granell A. Plant SWEETs: from sugar transport to plant-pathogen interaction and more unexpected physiological roles. Plant Physiol 2021;186:836-52.**

**[19] Chen LQ, Hou BH, Lalonde S, Takanaga H, Hartung ML, Qu XQ, et al. Sugar transporters for intercellular exchange and nutrition of pathogens. Nature 2010;468:527-32.**

**[20] Eom JS, Chen LQ, Sosso D, Julius BT, Lin IW, Qu XQ, et al. SWEETs, transporters for intracellular and intercellular sugar translocation. Curr Opin Plant Biol 2015;25:53-62.**

**[21] Li Y, Feng S, Ma S, Sui X, Zhang Z. Spatiotemporal Expression and Substrate Specificity Analysis of the Cucumber SWEET Gene Family. Front Plant Sci 2017;8:1855.**

**[22] Patil G, Valliyodan B, Deshmukh R, Prince S, Nicander B, Zhao M, et al. Soybean (Glycine max) SWEET gene family: insights through comparative genomics, transcriptome profiling and whole genome re-sequence analysis. BMC Genomics 2015;16:520.**

**[23] Zhang W, Wang S, Yu F, Tang J, Shan X, Bao K, et al. Genome-wide characterization and expression profiling of SWEET genes in cabbage (Brassica oleracea var. capitata L.) reveal their roles in chilling and clubroot disease responses. BMC Genomics 2019;20:93.**

**[24] Feng CY, Han JX, Han XX, Jiang J. Genome-wide identification, phylogeny, and expression analysis of the SWEET gene family in tomato. Gene 2015;573:261-72.**

**[25] Manck-Götzenberger J, Requena N. Arbuscular mycorrhiza Symbiosis Induces a Major Transcriptional Reprogramming of the Potato SWEET Sugar Transporter Family. Front Plant Sci 2016;7:487.**

**[26] Ko HY, Ho LH, Neuhaus HE, Guo WJ. Transporter SlSWEET15 unloads sucrose from phloem and seed coat for fruit and seed development in tomato. Plant Physiol 2021;187:2230-45.**

**[27] Zhang X, Feng C, Wang M, Li T, Liu X, Jiang J. Plasma membrane-localized SlSWEET7a and SlSWEET14 regulate sugar transport and storage in tomato fruits. Hortic Res 2021;8:186.**

**[28] Pan L, Guo Q, Chai S, Cheng Y, Ruan M, Ye Q, et al. Evolutionary Conservation and Expression Patterns of Neutral/Alkaline Invertases in Solanum. Biomolecules 2019;9.**

**[29] Veillet F, Gaillard C, Coutos-Thévenot P, La Camera S. Targeting the AtCWIN1 Gene to Explore the Role of Invertases in Sucrose Transport in Roots and during Botrytis cinerea Infection. Front Plant Sci 2016;7:1899.**

**[30] Wan H, Wu L, Yang Y, Zhou G, Ruan YL. Evolution of Sucrose Metabolism: The Dichotomy of Invertases and Beyond. Trends Plant Sci 2018;23:163-77.**

**[31] Jin Y, Ni DA, Ruan YL. Posttranslational elevation of cell wall invertase activity by silencing its inhibitor in tomato delays leaf senescence and increases seed weight and fruit hexose level. Plant Cell 2009;21:2072-89.**

**[32] Shen S, Ma S, Liu Y, Liao S, Li J, Wu L, et al. Cell Wall Invertase and Sugar Transporters Are Differentially Activated in Tomato Styles and Ovaries During Pollination and Fertilization. Front Plant Sci 2019;10:506.**

**[33] Lombard V, Golaconda Ramulu H, Drula E, Coutinho PM, Henrissat B. The carbohydrate-active enzymes database (CAZy) in 2013. Nucleic Acids Res 2014;42:D490-5.**

**[34] Zhang H, Yohe T, Huang L, Entwistle S, Wu P, Yang Z, et al. dbCAN2: a meta server for automated carbohydrate-active enzyme annotation. Nucleic Acids Res 2018;46:W95-w101.**

**[1] Gao Y, Wei Y, Wang Y, Gao F, Chen Z. Lycium Barbarum: A Traditional Chinese Herb and A Promising Anti-Aging Agent. Aging Dis 2017;8:778-91.**

**[2] Sun C, Chen X, Yang S, Jin C, Ding K, Chen C. LBP1C-2 from Lycium barbarum alleviated age-related bone loss by targeting BMPRIA/BMPRII/Noggin. Carbohydr Polym 2023;310:120725.**

**[3] Potterat O. Goji (Lycium barbarum and L. chinense): Phytochemistry, pharmacology and safety in the perspective of traditional uses and recent popularity. Planta Med 2010;76:7-19.**

**[4] Zhang XJ, Yu HY, Cai YJ, Ke M. Lycium barbarum polysaccharides inhibit proliferation and migration of bladder cancer cell lines BIU87 by suppressing Pi3K/AKT pathway. Oncotarget 2017;8:5936-42.**

**[5] Tang L, Bao S, Du Y, Jiang Z, Wuliji AO, Ren X, et al. Antioxidant effects of Lycium barbarum polysaccharides on photoreceptor degeneration in the light-exposed mouse retina. Biomed Pharmacother 2018;103:829-37.**

**[6] Zhang W, Zhang J, Ding D, Zhang L, Muehlmann LA, Deng SE, et al. Synthesis and antioxidant properties of Lycium barbarum polysaccharides capped selenium nanoparticles using tea extract. Artif Cells Nanomed Biotechnol 2018;46:1463-70.**

**[7] Ma K, Wang X, Feng S, Xia X, Zhang H, Rahaman A, et al. From the perspective of Traditional Chinese Medicine: Treatment of mental disorders in COVID-19 survivors. Biomed Pharmacother 2020;132:110810.**

**[8] Po KK, Leung JW, Chan JN, Fung TK, Sánchez-Vidaña DI, Sin EL, et al. Protective effect of Lycium Barbarum polysaccharides on dextromethorphan-induced mood impairment and neurogenesis suppression. Brain Res Bull 2017;134:10-7.**

**[9] Fang S, Dong L, Liu L, Guo J, Zhao L, Zhang J, et al. HERB: a high-throughput experiment- and reference-guided database of traditional Chinese medicine. Nucleic Acids Res 2021;49:D1197-d206.**

**[10] Zhang S, He F, Chen X, Ding K. Isolation and structural characterization of a pectin from Lycium ruthenicum Murr and its anti-pancreatic ductal adenocarcinoma cell activity. Carbohydr Polym 2019;223:115104.**

**[11] Cao YL, Li YL, Fan YF, Li Z, Yoshida K, Wang JY, et al. Wolfberry genomes and the evolution of Lycium (Solanaceae). Commun Biol 2021;4:671.**

**[12] Chen J, Liu X, Zhu L, Wang Y. Nuclear genome size estimation and karyotype analysis of Lycium species (Solanaceae). Scientia Horticulturae 2013;151:46-50.**

**[13] Giri MK, Swain S, Gautam JK, Singh S, Singh N, Bhattacharjee L, et al. The Arabidopsis thaliana At4g13040 gene, a unique member of the AP2/EREBP family, is a positive regulator for salicylic acid accumulation and basal defense against bacterial pathogens. J Plant Physiol 2014;171:860-7.**

**[14] Michaels SD, Ditta G, Gustafson-Brown C, Pelaz S, Yanofsky M, Amasino RM. AGL24 acts as a promoter of flowering in Arabidopsis and is positively regulated by vernalization. Plant J 2003;33:867-74.**

**[15] Schauser L, Roussis A, Stiller J, Stougaard J. A plant regulator controlling development of symbiotic root nodules. Nature 1999;402:191-5.**

**[16] Hou X, Zhou J, Liu C, Liu L, Shen L, Yu H. Nuclear factor Y-mediated H3K27me3 demethylation of the SOC1 locus orchestrates flowering responses of Arabidopsis. Nat Commun 2014;5:4601.**

**[17] Ru L, He Y, Zhu Z, Patrick JW, Ruan YL. Integrating Sugar Metabolism With Transport: Elevation of Endogenous Cell Wall Invertase Activity Up-Regulates SlHT2 and SlSWEET12c Expression for Early Fruit Development in Tomato. Front Genet 2020;11:592596.**

**[18] Breia R, Conde A, Badim H, Fortes AM, Gerós H, Granell A. Plant SWEETs: from sugar transport to plant-pathogen interaction and more unexpected physiological roles. Plant Physiol 2021;186:836-52.**

**[19] Chen LQ, Hou BH, Lalonde S, Takanaga H, Hartung ML, Qu XQ, et al. Sugar transporters for intercellular exchange and nutrition of pathogens. Nature 2010;468:527-32.**

**[20] Eom JS, Chen LQ, Sosso D, Julius BT, Lin IW, Qu XQ, et al. SWEETs, transporters for intracellular and intercellular sugar translocation. Curr Opin Plant Biol 2015;25:53-62.**

**[21] Li Y, Feng S, Ma S, Sui X, Zhang Z. Spatiotemporal Expression and Substrate Specificity Analysis of the Cucumber SWEET Gene Family. Front Plant Sci 2017;8:1855.**

**[22] Patil G, Valliyodan B, Deshmukh R, Prince S, Nicander B, Zhao M, et al. Soybean (Glycine max) SWEET gene family: insights through comparative genomics, transcriptome profiling and whole genome re-sequence analysis. BMC Genomics 2015;16:520.**

**[23] Zhang W, Wang S, Yu F, Tang J, Shan X, Bao K, et al. Genome-wide characterization and expression profiling of SWEET genes in cabbage (Brassica oleracea var. capitata L.) reveal their roles in chilling and clubroot disease responses. BMC Genomics 2019;20:93.**

**[24] Feng CY, Han JX, Han XX, Jiang J. Genome-wide identification, phylogeny, and expression analysis of the SWEET gene family in tomato. Gene 2015;573:261-72.**

**[25] Manck-Götzenberger J, Requena N. Arbuscular mycorrhiza Symbiosis Induces a Major Transcriptional Reprogramming of the Potato SWEET Sugar Transporter Family. Front Plant Sci 2016;7:487.**

**[26] Ko HY, Ho LH, Neuhaus HE, Guo WJ. Transporter SlSWEET15 unloads sucrose from phloem and seed coat for fruit and seed development in tomato. Plant Physiol 2021;187:2230-45.**

**[27] Zhang X, Feng C, Wang M, Li T, Liu X, Jiang J. Plasma membrane-localized SlSWEET7a and SlSWEET14 regulate sugar transport and storage in tomato fruits. Hortic Res 2021;8:186.**

**[28] Pan L, Guo Q, Chai S, Cheng Y, Ruan M, Ye Q, et al. Evolutionary Conservation and Expression Patterns of Neutral/Alkaline Invertases in Solanum. Biomolecules 2019;9.**

**[29] Veillet F, Gaillard C, Coutos-Thévenot P, La Camera S. Targeting the AtCWIN1 Gene to Explore the Role of Invertases in Sucrose Transport in Roots and during Botrytis cinerea Infection. Front Plant Sci 2016;7:1899.**

**[30] Wan H, Wu L, Yang Y, Zhou G, Ruan YL. Evolution of Sucrose Metabolism: The Dichotomy of Invertases and Beyond. Trends Plant Sci 2018;23:163-77.**

**[31] Jin Y, Ni DA, Ruan YL. Posttranslational elevation of cell wall invertase activity by silencing its inhibitor in tomato delays leaf senescence and increases seed weight and fruit hexose level. Plant Cell 2009;21:2072-89.**

**[32] Shen S, Ma S, Liu Y, Liao S, Li J, Wu L, et al. Cell Wall Invertase and Sugar Transporters Are Differentially Activated in Tomato Styles and Ovaries During Pollination and Fertilization. Front Plant Sci 2019;10:506.**

**[33] Lombard V, Golaconda Ramulu H, Drula E, Coutinho PM, Henrissat B. The carbohydrate-active enzymes database (CAZy) in 2013. Nucleic Acids Res 2014;42:D490-5.**

**[34] Zhang H, Yohe T, Huang L, Entwistle S, Wu P, Yang Z, et al. dbCAN2: a meta server for automated carbohydrate-active enzyme annotation. Nucleic Acids Res 2018;46:W95-w101.**

**[35] Langfelder P, Horvath S. WGCNA: an R package for weighted correlation network analysis. BMC Bioinformatics 2008;9:559.**

**[1] Gao Y, Wei Y, Wang Y, Gao F, Chen Z. Lycium Barbarum: A Traditional Chinese Herb and A Promising Anti-Aging Agent. Aging Dis 2017;8:778-91.**

**[2] Sun C, Chen X, Yang S, Jin C, Ding K, Chen C. LBP1C-2 from Lycium barbarum alleviated age-related bone loss by targeting BMPRIA/BMPRII/Noggin. Carbohydr Polym 2023;310:120725.**

**[3] Potterat O. Goji (Lycium barbarum and L. chinense): Phytochemistry, pharmacology and safety in the perspective of traditional uses and recent popularity. Planta Med 2010;76:7-19.**

**[4] Zhang XJ, Yu HY, Cai YJ, Ke M. Lycium barbarum polysaccharides inhibit proliferation and migration of bladder cancer cell lines BIU87 by suppressing Pi3K/AKT pathway. Oncotarget 2017;8:5936-42.**

**[5] Tang L, Bao S, Du Y, Jiang Z, Wuliji AO, Ren X, et al. Antioxidant effects of Lycium barbarum polysaccharides on photoreceptor degeneration in the light-exposed mouse retina. Biomed Pharmacother 2018;103:829-37.**

**[6] Zhang W, Zhang J, Ding D, Zhang L, Muehlmann LA, Deng SE, et al. Synthesis and antioxidant properties of Lycium barbarum polysaccharides capped selenium nanoparticles using tea extract. Artif Cells Nanomed Biotechnol 2018;46:1463-70.**

**[7] Ma K, Wang X, Feng S, Xia X, Zhang H, Rahaman A, et al. From the perspective of Traditional Chinese Medicine: Treatment of mental disorders in COVID-19 survivors. Biomed Pharmacother 2020;132:110810.**

**[8] Po KK, Leung JW, Chan JN, Fung TK, Sánchez-Vidaña DI, Sin EL, et al. Protective effect of Lycium Barbarum polysaccharides on dextromethorphan-induced mood impairment and neurogenesis suppression. Brain Res Bull 2017;134:10-7.**

**[9] Fang S, Dong L, Liu L, Guo J, Zhao L, Zhang J, et al. HERB: a high-throughput experiment- and reference-guided database of traditional Chinese medicine. Nucleic Acids Res 2021;49:D1197-d206.**

**[10] Zhang S, He F, Chen X, Ding K. Isolation and structural characterization of a pectin from Lycium ruthenicum Murr and its anti-pancreatic ductal adenocarcinoma cell activity. Carbohydr Polym 2019;223:115104.**

**[11] Cao YL, Li YL, Fan YF, Li Z, Yoshida K, Wang JY, et al. Wolfberry genomes and the evolution of Lycium (Solanaceae). Commun Biol 2021;4:671.**

**[12] Chen J, Liu X, Zhu L, Wang Y. Nuclear genome size estimation and karyotype analysis of Lycium species (Solanaceae). Scientia Horticulturae 2013;151:46-50.**

**[13] Giri MK, Swain S, Gautam JK, Singh S, Singh N, Bhattacharjee L, et al. The Arabidopsis thaliana At4g13040 gene, a unique member of the AP2/EREBP family, is a positive regulator for salicylic acid accumulation and basal defense against bacterial pathogens. J Plant Physiol 2014;171:860-7.**

**[14] Michaels SD, Ditta G, Gustafson-Brown C, Pelaz S, Yanofsky M, Amasino RM. AGL24 acts as a promoter of flowering in Arabidopsis and is positively regulated by vernalization. Plant J 2003;33:867-74.**

**[15] Schauser L, Roussis A, Stiller J, Stougaard J. A plant regulator controlling development of symbiotic root nodules. Nature 1999;402:191-5.**

**[16] Hou X, Zhou J, Liu C, Liu L, Shen L, Yu H. Nuclear factor Y-mediated H3K27me3 demethylation of the SOC1 locus orchestrates flowering responses of Arabidopsis. Nat Commun 2014;5:4601.**

**[17] Ru L, He Y, Zhu Z, Patrick JW, Ruan YL. Integrating Sugar Metabolism With Transport: Elevation of Endogenous Cell Wall Invertase Activity Up-Regulates SlHT2 and SlSWEET12c Expression for Early Fruit Development in Tomato. Front Genet 2020;11:592596.**

**[18] Breia R, Conde A, Badim H, Fortes AM, Gerós H, Granell A. Plant SWEETs: from sugar transport to plant-pathogen interaction and more unexpected physiological roles. Plant Physiol 2021;186:836-52.**

**[19] Chen LQ, Hou BH, Lalonde S, Takanaga H, Hartung ML, Qu XQ, et al. Sugar transporters for intercellular exchange and nutrition of pathogens. Nature 2010;468:527-32.**

**[20] Eom JS, Chen LQ, Sosso D, Julius BT, Lin IW, Qu XQ, et al. SWEETs, transporters for intracellular and intercellular sugar translocation. Curr Opin Plant Biol 2015;25:53-62.**

**[21] Li Y, Feng S, Ma S, Sui X, Zhang Z. Spatiotemporal Expression and Substrate Specificity Analysis of the Cucumber SWEET Gene Family. Front Plant Sci 2017;8:1855.**

**[22] Patil G, Valliyodan B, Deshmukh R, Prince S, Nicander B, Zhao M, et al. Soybean (Glycine max) SWEET gene family: insights through comparative genomics, transcriptome profiling and whole genome re-sequence analysis. BMC Genomics 2015;16:520.**

**[23] Zhang W, Wang S, Yu F, Tang J, Shan X, Bao K, et al. Genome-wide characterization and expression profiling of SWEET genes in cabbage (Brassica oleracea var. capitata L.) reveal their roles in chilling and clubroot disease responses. BMC Genomics 2019;20:93.**

**[24] Feng CY, Han JX, Han XX, Jiang J. Genome-wide identification, phylogeny, and expression analysis of the SWEET gene family in tomato. Gene 2015;573:261-72.**

**[25] Manck-Götzenberger J, Requena N. Arbuscular mycorrhiza Symbiosis Induces a Major Transcriptional Reprogramming of the Potato SWEET Sugar Transporter Family. Front Plant Sci 2016;7:487.**

**[26] Ko HY, Ho LH, Neuhaus HE, Guo WJ. Transporter SlSWEET15 unloads sucrose from phloem and seed coat for fruit and seed development in tomato. Plant Physiol 2021;187:2230-45.**

**[27] Zhang X, Feng C, Wang M, Li T, Liu X, Jiang J. Plasma membrane-localized SlSWEET7a and SlSWEET14 regulate sugar transport and storage in tomato fruits. Hortic Res 2021;8:186.**

**[28] Pan L, Guo Q, Chai S, Cheng Y, Ruan M, Ye Q, et al. Evolutionary Conservation and Expression Patterns of Neutral/Alkaline Invertases in Solanum. Biomolecules 2019;9.**

**[29] Veillet F, Gaillard C, Coutos-Thévenot P, La Camera S. Targeting the AtCWIN1 Gene to Explore the Role of Invertases in Sucrose Transport in Roots and during Botrytis cinerea Infection. Front Plant Sci 2016;7:1899.**

**[30] Wan H, Wu L, Yang Y, Zhou G, Ruan YL. Evolution of Sucrose Metabolism: The Dichotomy of Invertases and Beyond. Trends Plant Sci 2018;23:163-77.**

**[31] Jin Y, Ni DA, Ruan YL. Posttranslational elevation of cell wall invertase activity by silencing its inhibitor in tomato delays leaf senescence and increases seed weight and fruit hexose level. Plant Cell 2009;21:2072-89.**

**[32] Shen S, Ma S, Liu Y, Liao S, Li J, Wu L, et al. Cell Wall Invertase and Sugar Transporters Are Differentially Activated in Tomato Styles and Ovaries During Pollination and Fertilization. Front Plant Sci 2019;10:506.**

**[33] Lombard V, Golaconda Ramulu H, Drula E, Coutinho PM, Henrissat B. The carbohydrate-active enzymes database (CAZy) in 2013. Nucleic Acids Res 2014;42:D490-5.**

**[34] Zhang H, Yohe T, Huang L, Entwistle S, Wu P, Yang Z, et al. dbCAN2: a meta server for automated carbohydrate-active enzyme annotation. Nucleic Acids Res 2018;46:W95-w101.**

**[35] Langfelder P, Horvath S. WGCNA: an R package for weighted correlation network analysis. BMC Bioinformatics 2008;9:559.**

**[1] Gao Y, Wei Y, Wang Y, Gao F, Chen Z. Lycium Barbarum: A Traditional Chinese Herb and A Promising Anti-Aging Agent. Aging Dis 2017;8:778-91.**

**[2] Sun C, Chen X, Yang S, Jin C, Ding K, Chen C. LBP1C-2 from Lycium barbarum alleviated age-related bone loss by targeting BMPRIA/BMPRII/Noggin. Carbohydr Polym 2023;310:120725.**

**[3] Potterat O. Goji (Lycium barbarum and L. chinense): Phytochemistry, pharmacology and safety in the perspective of traditional uses and recent popularity. Planta Med 2010;76:7-19.**

**[4] Zhang XJ, Yu HY, Cai YJ, Ke M. Lycium barbarum polysaccharides inhibit proliferation and migration of bladder cancer cell lines BIU87 by suppressing Pi3K/AKT pathway. Oncotarget 2017;8:5936-42.**

**[5] Tang L, Bao S, Du Y, Jiang Z, Wuliji AO, Ren X, et al. Antioxidant effects of Lycium barbarum polysaccharides on photoreceptor degeneration in the light-exposed mouse retina. Biomed Pharmacother 2018;103:829-37.**

**[6] Zhang W, Zhang J, Ding D, Zhang L, Muehlmann LA, Deng SE, et al. Synthesis and antioxidant properties of Lycium barbarum polysaccharides capped selenium nanoparticles using tea extract. Artif Cells Nanomed Biotechnol 2018;46:1463-70.**

**[7] Ma K, Wang X, Feng S, Xia X, Zhang H, Rahaman A, et al. From the perspective of Traditional Chinese Medicine: Treatment of mental disorders in COVID-19 survivors. Biomed Pharmacother 2020;132:110810.**

**[8] Po KK, Leung JW, Chan JN, Fung TK, Sánchez-Vidaña DI, Sin EL, et al. Protective effect of Lycium Barbarum polysaccharides on dextromethorphan-induced mood impairment and neurogenesis suppression. Brain Res Bull 2017;134:10-7.**

**[9] Fang S, Dong L, Liu L, Guo J, Zhao L, Zhang J, et al. HERB: a high-throughput experiment- and reference-guided database of traditional Chinese medicine. Nucleic Acids Res 2021;49:D1197-d206.**

**[10] Zhang S, He F, Chen X, Ding K. Isolation and structural characterization of a pectin from Lycium ruthenicum Murr and its anti-pancreatic ductal adenocarcinoma cell activity. Carbohydr Polym 2019;223:115104.**

**[11] Cao YL, Li YL, Fan YF, Li Z, Yoshida K, Wang JY, et al. Wolfberry genomes and the evolution of Lycium (Solanaceae). Commun Biol 2021;4:671.**

**[12] Chen J, Liu X, Zhu L, Wang Y. Nuclear genome size estimation and karyotype analysis of Lycium species (Solanaceae). Scientia Horticulturae 2013;151:46-50.**

**[13] Giri MK, Swain S, Gautam JK, Singh S, Singh N, Bhattacharjee L, et al. The Arabidopsis thaliana At4g13040 gene, a unique member of the AP2/EREBP family, is a positive regulator for salicylic acid accumulation and basal defense against bacterial pathogens. J Plant Physiol 2014;171:860-7.**

**[14] Michaels SD, Ditta G, Gustafson-Brown C, Pelaz S, Yanofsky M, Amasino RM. AGL24 acts as a promoter of flowering in Arabidopsis and is positively regulated by vernalization. Plant J 2003;33:867-74.**

**[15] Schauser L, Roussis A, Stiller J, Stougaard J. A plant regulator controlling development of symbiotic root nodules. Nature 1999;402:191-5.**

**[16] Hou X, Zhou J, Liu C, Liu L, Shen L, Yu H. Nuclear factor Y-mediated H3K27me3 demethylation of the SOC1 locus orchestrates flowering responses of Arabidopsis. Nat Commun 2014;5:4601.**

**[17] Ru L, He Y, Zhu Z, Patrick JW, Ruan YL. Integrating Sugar Metabolism With Transport: Elevation of Endogenous Cell Wall Invertase Activity Up-Regulates SlHT2 and SlSWEET12c Expression for Early Fruit Development in Tomato. Front Genet 2020;11:592596.**

**[18] Breia R, Conde A, Badim H, Fortes AM, Gerós H, Granell A. Plant SWEETs: from sugar transport to plant-pathogen interaction and more unexpected physiological roles. Plant Physiol 2021;186:836-52.**

**[19] Chen LQ, Hou BH, Lalonde S, Takanaga H, Hartung ML, Qu XQ, et al. Sugar transporters for intercellular exchange and nutrition of pathogens. Nature 2010;468:527-32.**

**[20] Eom JS, Chen LQ, Sosso D, Julius BT, Lin IW, Qu XQ, et al. SWEETs, transporters for intracellular and intercellular sugar translocation. Curr Opin Plant Biol 2015;25:53-62.**

**[21] Li Y, Feng S, Ma S, Sui X, Zhang Z. Spatiotemporal Expression and Substrate Specificity Analysis of the Cucumber SWEET Gene Family. Front Plant Sci 2017;8:1855.**

**[22] Patil G, Valliyodan B, Deshmukh R, Prince S, Nicander B, Zhao M, et al. Soybean (Glycine max) SWEET gene family: insights through comparative genomics, transcriptome profiling and whole genome re-sequence analysis. BMC Genomics 2015;16:520.**

**[23] Zhang W, Wang S, Yu F, Tang J, Shan X, Bao K, et al. Genome-wide characterization and expression profiling of SWEET genes in cabbage (Brassica oleracea var. capitata L.) reveal their roles in chilling and clubroot disease responses. BMC Genomics 2019;20:93.**

**[24] Feng CY, Han JX, Han XX, Jiang J. Genome-wide identification, phylogeny, and expression analysis of the SWEET gene family in tomato. Gene 2015;573:261-72.**

**[25] Manck-Götzenberger J, Requena N. Arbuscular mycorrhiza Symbiosis Induces a Major Transcriptional Reprogramming of the Potato SWEET Sugar Transporter Family. Front Plant Sci 2016;7:487.**

**[26] Ko HY, Ho LH, Neuhaus HE, Guo WJ. Transporter SlSWEET15 unloads sucrose from phloem and seed coat for fruit and seed development in tomato. Plant Physiol 2021;187:2230-45.**

**[27] Zhang X, Feng C, Wang M, Li T, Liu X, Jiang J. Plasma membrane-localized SlSWEET7a and SlSWEET14 regulate sugar transport and storage in tomato fruits. Hortic Res 2021;8:186.**

**[28] Pan L, Guo Q, Chai S, Cheng Y, Ruan M, Ye Q, et al. Evolutionary Conservation and Expression Patterns of Neutral/Alkaline Invertases in Solanum. Biomolecules 2019;9.**

**[29] Veillet F, Gaillard C, Coutos-Thévenot P, La Camera S. Targeting the AtCWIN1 Gene to Explore the Role of Invertases in Sucrose Transport in Roots and during Botrytis cinerea Infection. Front Plant Sci 2016;7:1899.**

**[30] Wan H, Wu L, Yang Y, Zhou G, Ruan YL. Evolution of Sucrose Metabolism: The Dichotomy of Invertases and Beyond. Trends Plant Sci 2018;23:163-77.**

**[31] Jin Y, Ni DA, Ruan YL. Posttranslational elevation of cell wall invertase activity by silencing its inhibitor in tomato delays leaf senescence and increases seed weight and fruit hexose level. Plant Cell 2009;21:2072-89.**

**[32] Shen S, Ma S, Liu Y, Liao S, Li J, Wu L, et al. Cell Wall Invertase and Sugar Transporters Are Differentially Activated in Tomato Styles and Ovaries During Pollination and Fertilization. Front Plant Sci 2019;10:506.**

**[33] Lombard V, Golaconda Ramulu H, Drula E, Coutinho PM, Henrissat B. The carbohydrate-active enzymes database (CAZy) in 2013. Nucleic Acids Res 2014;42:D490-5.**

**[34] Zhang H, Yohe T, Huang L, Entwistle S, Wu P, Yang Z, et al. dbCAN2: a meta server for automated carbohydrate-active enzyme annotation. Nucleic Acids Res 2018;46:W95-w101.**

**[35] Langfelder P, Horvath S. WGCNA: an R package for weighted correlation network analysis. BMC Bioinformatics 2008;9:559.**

**[36] Kaczmarska A, Pieczywek PM, Cybulska J, Zdunek A. Structure and functionality of Rhamnogalacturonan I in the cell wall and in solution: A review. Carbohydr Polym 2022;278:118909.**

**[1] Gao Y, Wei Y, Wang Y, Gao F, Chen Z. Lycium Barbarum: A Traditional Chinese Herb and A Promising Anti-Aging Agent. Aging Dis 2017;8:778-91.**

**[2] Sun C, Chen X, Yang S, Jin C, Ding K, Chen C. LBP1C-2 from Lycium barbarum alleviated age-related bone loss by targeting BMPRIA/BMPRII/Noggin. Carbohydr Polym 2023;310:120725.**

**[3] Potterat O. Goji (Lycium barbarum and L. chinense): Phytochemistry, pharmacology and safety in the perspective of traditional uses and recent popularity. Planta Med 2010;76:7-19.**

**[4] Zhang XJ, Yu HY, Cai YJ, Ke M. Lycium barbarum polysaccharides inhibit proliferation and migration of bladder cancer cell lines BIU87 by suppressing Pi3K/AKT pathway. Oncotarget 2017;8:5936-42.**

**[5] Tang L, Bao S, Du Y, Jiang Z, Wuliji AO, Ren X, et al. Antioxidant effects of Lycium barbarum polysaccharides on photoreceptor degeneration in the light-exposed mouse retina. Biomed Pharmacother 2018;103:829-37.**

**[6] Zhang W, Zhang J, Ding D, Zhang L, Muehlmann LA, Deng SE, et al. Synthesis and antioxidant properties of Lycium barbarum polysaccharides capped selenium nanoparticles using tea extract. Artif Cells Nanomed Biotechnol 2018;46:1463-70.**

**[7] Ma K, Wang X, Feng S, Xia X, Zhang H, Rahaman A, et al. From the perspective of Traditional Chinese Medicine: Treatment of mental disorders in COVID-19 survivors. Biomed Pharmacother 2020;132:110810.**

**[8] Po KK, Leung JW, Chan JN, Fung TK, Sánchez-Vidaña DI, Sin EL, et al. Protective effect of Lycium Barbarum polysaccharides on dextromethorphan-induced mood impairment and neurogenesis suppression. Brain Res Bull 2017;134:10-7.**

**[9] Fang S, Dong L, Liu L, Guo J, Zhao L, Zhang J, et al. HERB: a high-throughput experiment- and reference-guided database of traditional Chinese medicine. Nucleic Acids Res 2021;49:D1197-d206.**

**[10] Zhang S, He F, Chen X, Ding K. Isolation and structural characterization of a pectin from Lycium ruthenicum Murr and its anti-pancreatic ductal adenocarcinoma cell activity. Carbohydr Polym 2019;223:115104.**

**[11] Cao YL, Li YL, Fan YF, Li Z, Yoshida K, Wang JY, et al. Wolfberry genomes and the evolution of Lycium (Solanaceae). Commun Biol 2021;4:671.**

**[12] Chen J, Liu X, Zhu L, Wang Y. Nuclear genome size estimation and karyotype analysis of Lycium species (Solanaceae). Scientia Horticulturae 2013;151:46-50.**

**[13] Giri MK, Swain S, Gautam JK, Singh S, Singh N, Bhattacharjee L, et al. The Arabidopsis thaliana At4g13040 gene, a unique member of the AP2/EREBP family, is a positive regulator for salicylic acid accumulation and basal defense against bacterial pathogens. J Plant Physiol 2014;171:860-7.**

**[14] Michaels SD, Ditta G, Gustafson-Brown C, Pelaz S, Yanofsky M, Amasino RM. AGL24 acts as a promoter of flowering in Arabidopsis and is positively regulated by vernalization. Plant J 2003;33:867-74.**

**[15] Schauser L, Roussis A, Stiller J, Stougaard J. A plant regulator controlling development of symbiotic root nodules. Nature 1999;402:191-5.**

**[16] Hou X, Zhou J, Liu C, Liu L, Shen L, Yu H. Nuclear factor Y-mediated H3K27me3 demethylation of the SOC1 locus orchestrates flowering responses of Arabidopsis. Nat Commun 2014;5:4601.**

**[17] Ru L, He Y, Zhu Z, Patrick JW, Ruan YL. Integrating Sugar Metabolism With Transport: Elevation of Endogenous Cell Wall Invertase Activity Up-Regulates SlHT2 and SlSWEET12c Expression for Early Fruit Development in Tomato. Front Genet 2020;11:592596.**

**[18] Breia R, Conde A, Badim H, Fortes AM, Gerós H, Granell A. Plant SWEETs: from sugar transport to plant-pathogen interaction and more unexpected physiological roles. Plant Physiol 2021;186:836-52.**

**[19] Chen LQ, Hou BH, Lalonde S, Takanaga H, Hartung ML, Qu XQ, et al. Sugar transporters for intercellular exchange and nutrition of pathogens. Nature 2010;468:527-32.**

**[20] Eom JS, Chen LQ, Sosso D, Julius BT, Lin IW, Qu XQ, et al. SWEETs, transporters for intracellular and intercellular sugar translocation. Curr Opin Plant Biol 2015;25:53-62.**

**[21] Li Y, Feng S, Ma S, Sui X, Zhang Z. Spatiotemporal Expression and Substrate Specificity Analysis of the Cucumber SWEET Gene Family. Front Plant Sci 2017;8:1855.**

**[22] Patil G, Valliyodan B, Deshmukh R, Prince S, Nicander B, Zhao M, et al. Soybean (Glycine max) SWEET gene family: insights through comparative genomics, transcriptome profiling and whole genome re-sequence analysis. BMC Genomics 2015;16:520.**

**[23] Zhang W, Wang S, Yu F, Tang J, Shan X, Bao K, et al. Genome-wide characterization and expression profiling of SWEET genes in cabbage (Brassica oleracea var. capitata L.) reveal their roles in chilling and clubroot disease responses. BMC Genomics 2019;20:93.**

**[24] Feng CY, Han JX, Han XX, Jiang J. Genome-wide identification, phylogeny, and expression analysis of the SWEET gene family in tomato. Gene 2015;573:261-72.**

**[25] Manck-Götzenberger J, Requena N. Arbuscular mycorrhiza Symbiosis Induces a Major Transcriptional Reprogramming of the Potato SWEET Sugar Transporter Family. Front Plant Sci 2016;7:487.**

**[26] Ko HY, Ho LH, Neuhaus HE, Guo WJ. Transporter SlSWEET15 unloads sucrose from phloem and seed coat for fruit and seed development in tomato. Plant Physiol 2021;187:2230-45.**

**[27] Zhang X, Feng C, Wang M, Li T, Liu X, Jiang J. Plasma membrane-localized SlSWEET7a and SlSWEET14 regulate sugar transport and storage in tomato fruits. Hortic Res 2021;8:186.**

**[28] Pan L, Guo Q, Chai S, Cheng Y, Ruan M, Ye Q, et al. Evolutionary Conservation and Expression Patterns of Neutral/Alkaline Invertases in Solanum. Biomolecules 2019;9.**

**[29] Veillet F, Gaillard C, Coutos-Thévenot P, La Camera S. Targeting the AtCWIN1 Gene to Explore the Role of Invertases in Sucrose Transport in Roots and during Botrytis cinerea Infection. Front Plant Sci 2016;7:1899.**

**[30] Wan H, Wu L, Yang Y, Zhou G, Ruan YL. Evolution of Sucrose Metabolism: The Dichotomy of Invertases and Beyond. Trends Plant Sci 2018;23:163-77.**

**[31] Jin Y, Ni DA, Ruan YL. Posttranslational elevation of cell wall invertase activity by silencing its inhibitor in tomato delays leaf senescence and increases seed weight and fruit hexose level. Plant Cell 2009;21:2072-89.**

**[32] Shen S, Ma S, Liu Y, Liao S, Li J, Wu L, et al. Cell Wall Invertase and Sugar Transporters Are Differentially Activated in Tomato Styles and Ovaries During Pollination and Fertilization. Front Plant Sci 2019;10:506.**

**[33] Lombard V, Golaconda Ramulu H, Drula E, Coutinho PM, Henrissat B. The carbohydrate-active enzymes database (CAZy) in 2013. Nucleic Acids Res 2014;42:D490-5.**

**[34] Zhang H, Yohe T, Huang L, Entwistle S, Wu P, Yang Z, et al. dbCAN2: a meta server for automated carbohydrate-active enzyme annotation. Nucleic Acids Res 2018;46:W95-w101.**

**[35] Langfelder P, Horvath S. WGCNA: an R package for weighted correlation network analysis. BMC Bioinformatics 2008;9:559.**

**[36] Kaczmarska A, Pieczywek PM, Cybulska J, Zdunek A. Structure and functionality of Rhamnogalacturonan I in the cell wall and in solution: A review. Carbohydr Polym 2022;278:118909.**

**[1] Gao Y, Wei Y, Wang Y, Gao F, Chen Z. Lycium Barbarum: A Traditional Chinese Herb and A Promising Anti-Aging Agent. Aging Dis 2017;8:778-91.**

**[2] Sun C, Chen X, Yang S, Jin C, Ding K, Chen C. LBP1C-2 from Lycium barbarum alleviated age-related bone loss by targeting BMPRIA/BMPRII/Noggin. Carbohydr Polym 2023;310:120725.**

**[3] Potterat O. Goji (Lycium barbarum and L. chinense): Phytochemistry, pharmacology and safety in the perspective of traditional uses and recent popularity. Planta Med 2010;76:7-19.**

**[4] Zhang XJ, Yu HY, Cai YJ, Ke M. Lycium barbarum polysaccharides inhibit proliferation and migration of bladder cancer cell lines BIU87 by suppressing Pi3K/AKT pathway. Oncotarget 2017;8:5936-42.**

**[5] Tang L, Bao S, Du Y, Jiang Z, Wuliji AO, Ren X, et al. Antioxidant effects of Lycium barbarum polysaccharides on photoreceptor degeneration in the light-exposed mouse retina. Biomed Pharmacother 2018;103:829-37.**

**[6] Zhang W, Zhang J, Ding D, Zhang L, Muehlmann LA, Deng SE, et al. Synthesis and antioxidant properties of Lycium barbarum polysaccharides capped selenium nanoparticles using tea extract. Artif Cells Nanomed Biotechnol 2018;46:1463-70.**

**[7] Ma K, Wang X, Feng S, Xia X, Zhang H, Rahaman A, et al. From the perspective of Traditional Chinese Medicine: Treatment of mental disorders in COVID-19 survivors. Biomed Pharmacother 2020;132:110810.**

**[8] Po KK, Leung JW, Chan JN, Fung TK, Sánchez-Vidaña DI, Sin EL, et al. Protective effect of Lycium Barbarum polysaccharides on dextromethorphan-induced mood impairment and neurogenesis suppression. Brain Res Bull 2017;134:10-7.**

**[9] Fang S, Dong L, Liu L, Guo J, Zhao L, Zhang J, et al. HERB: a high-throughput experiment- and reference-guided database of traditional Chinese medicine. Nucleic Acids Res 2021;49:D1197-d206.**

**[10] Zhang S, He F, Chen X, Ding K. Isolation and structural characterization of a pectin from Lycium ruthenicum Murr and its anti-pancreatic ductal adenocarcinoma cell activity. Carbohydr Polym 2019;223:115104.**

**[11] Cao YL, Li YL, Fan YF, Li Z, Yoshida K, Wang JY, et al. Wolfberry genomes and the evolution of Lycium (Solanaceae). Commun Biol 2021;4:671.**

**[12] Chen J, Liu X, Zhu L, Wang Y. Nuclear genome size estimation and karyotype analysis of Lycium species (Solanaceae). Scientia Horticulturae 2013;151:46-50.**

**[13] Giri MK, Swain S, Gautam JK, Singh S, Singh N, Bhattacharjee L, et al. The Arabidopsis thaliana At4g13040 gene, a unique member of the AP2/EREBP family, is a positive regulator for salicylic acid accumulation and basal defense against bacterial pathogens. J Plant Physiol 2014;171:860-7.**

**[14] Michaels SD, Ditta G, Gustafson-Brown C, Pelaz S, Yanofsky M, Amasino RM. AGL24 acts as a promoter of flowering in Arabidopsis and is positively regulated by vernalization. Plant J 2003;33:867-74.**

**[15] Schauser L, Roussis A, Stiller J, Stougaard J. A plant regulator controlling development of symbiotic root nodules. Nature 1999;402:191-5.**

**[16] Hou X, Zhou J, Liu C, Liu L, Shen L, Yu H. Nuclear factor Y-mediated H3K27me3 demethylation of the SOC1 locus orchestrates flowering responses of Arabidopsis. Nat Commun 2014;5:4601.**

**[17] Ru L, He Y, Zhu Z, Patrick JW, Ruan YL. Integrating Sugar Metabolism With Transport: Elevation of Endogenous Cell Wall Invertase Activity Up-Regulates SlHT2 and SlSWEET12c Expression for Early Fruit Development in Tomato. Front Genet 2020;11:592596.**

**[18] Breia R, Conde A, Badim H, Fortes AM, Gerós H, Granell A. Plant SWEETs: from sugar transport to plant-pathogen interaction and more unexpected physiological roles. Plant Physiol 2021;186:836-52.**

**[19] Chen LQ, Hou BH, Lalonde S, Takanaga H, Hartung ML, Qu XQ, et al. Sugar transporters for intercellular exchange and nutrition of pathogens. Nature 2010;468:527-32.**

**[20] Eom JS, Chen LQ, Sosso D, Julius BT, Lin IW, Qu XQ, et al. SWEETs, transporters for intracellular and intercellular sugar translocation. Curr Opin Plant Biol 2015;25:53-62.**

**[21] Li Y, Feng S, Ma S, Sui X, Zhang Z. Spatiotemporal Expression and Substrate Specificity Analysis of the Cucumber SWEET Gene Family. Front Plant Sci 2017;8:1855.**

**[22] Patil G, Valliyodan B, Deshmukh R, Prince S, Nicander B, Zhao M, et al. Soybean (Glycine max) SWEET gene family: insights through comparative genomics, transcriptome profiling and whole genome re-sequence analysis. BMC Genomics 2015;16:520.**

**[23] Zhang W, Wang S, Yu F, Tang J, Shan X, Bao K, et al. Genome-wide characterization and expression profiling of SWEET genes in cabbage (Brassica oleracea var. capitata L.) reveal their roles in chilling and clubroot disease responses. BMC Genomics 2019;20:93.**

**[24] Feng CY, Han JX, Han XX, Jiang J. Genome-wide identification, phylogeny, and expression analysis of the SWEET gene family in tomato. Gene 2015;573:261-72.**

**[25] Manck-Götzenberger J, Requena N. Arbuscular mycorrhiza Symbiosis Induces a Major Transcriptional Reprogramming of the Potato SWEET Sugar Transporter Family. Front Plant Sci 2016;7:487.**

**[26] Ko HY, Ho LH, Neuhaus HE, Guo WJ. Transporter SlSWEET15 unloads sucrose from phloem and seed coat for fruit and seed development in tomato. Plant Physiol 2021;187:2230-45.**

**[27] Zhang X, Feng C, Wang M, Li T, Liu X, Jiang J. Plasma membrane-localized SlSWEET7a and SlSWEET14 regulate sugar transport and storage in tomato fruits. Hortic Res 2021;8:186.**

**[28] Pan L, Guo Q, Chai S, Cheng Y, Ruan M, Ye Q, et al. Evolutionary Conservation and Expression Patterns of Neutral/Alkaline Invertases in Solanum. Biomolecules 2019;9.**

**[29] Veillet F, Gaillard C, Coutos-Thévenot P, La Camera S. Targeting the AtCWIN1 Gene to Explore the Role of Invertases in Sucrose Transport in Roots and during Botrytis cinerea Infection. Front Plant Sci 2016;7:1899.**

**[30] Wan H, Wu L, Yang Y, Zhou G, Ruan YL. Evolution of Sucrose Metabolism: The Dichotomy of Invertases and Beyond. Trends Plant Sci 2018;23:163-77.**

**[31] Jin Y, Ni DA, Ruan YL. Posttranslational elevation of cell wall invertase activity by silencing its inhibitor in tomato delays leaf senescence and increases seed weight and fruit hexose level. Plant Cell 2009;21:2072-89.**

**[32] Shen S, Ma S, Liu Y, Liao S, Li J, Wu L, et al. Cell Wall Invertase and Sugar Transporters Are Differentially Activated in Tomato Styles and Ovaries During Pollination and Fertilization. Front Plant Sci 2019;10:506.**

**[33] Lombard V, Golaconda Ramulu H, Drula E, Coutinho PM, Henrissat B. The carbohydrate-active enzymes database (CAZy) in 2013. Nucleic Acids Res 2014;42:D490-5.**

**[34] Zhang H, Yohe T, Huang L, Entwistle S, Wu P, Yang Z, et al. dbCAN2: a meta server for automated carbohydrate-active enzyme annotation. Nucleic Acids Res 2018;46:W95-w101.**

**[35] Langfelder P, Horvath S. WGCNA: an R package for weighted correlation network analysis. BMC Bioinformatics 2008;9:559.**

**[36] Kaczmarska A, Pieczywek PM, Cybulska J, Zdunek A. Structure and functionality of Rhamnogalacturonan I in the cell wall and in solution: A review. Carbohydr Polym 2022;278:118909.**

**[37] Wachananawat B, Kuroha T, Takenaka Y, Kajiura H, Naramoto S, Yokoyama R, et al. Diversity of Pectin Rhamnogalacturonan I Rhamnosyltransferases in Glycosyltransferase Family 106. Front Plant Sci 2020;11:997.**

**[38] Atmodjo MA, Sakuragi Y, Zhu X, Burrell AJ, Mohanty SS, Atwood JA, 3rd, et al. Galacturonosyltransferase (GAUT)1 and GAUT7 are the core of a plant cell wall pectin biosynthetic homogalacturonan:galacturonosyltransferase complex. Proc Natl Acad Sci U S A 2011;108:20225-30.**

**[2] Sun C, Chen X, Yang S, Jin C, Ding K, Chen C. LBP1C-2 from Lycium barbarum alleviated age-related bone loss by targeting BMPRIA/BMPRII/Noggin. Carbohydr Polym 2023;310:120725.**

**[3] Potterat O. Goji (Lycium barbarum and L. chinense): Phytochemistry, pharmacology and safety in the perspective of traditional uses and recent popularity. Planta Med 2010;76:7-19.**

**[4] Zhang XJ, Yu HY, Cai YJ, Ke M. Lycium barbarum polysaccharides inhibit proliferation and migration of bladder cancer cell lines BIU87 by suppressing Pi3K/AKT pathway. Oncotarget 2017;8:5936-42.**

**[5] Tang L, Bao S, Du Y, Jiang Z, Wuliji AO, Ren X, et al. Antioxidant effects of Lycium barbarum polysaccharides on photoreceptor degeneration in the light-exposed mouse retina. Biomed Pharmacother 2018;103:829-37.**

**[6] Zhang W, Zhang J, Ding D, Zhang L, Muehlmann LA, Deng SE, et al. Synthesis and antioxidant properties of Lycium barbarum polysaccharides capped selenium nanoparticles using tea extract. Artif Cells Nanomed Biotechnol 2018;46:1463-70.**

**[7] Ma K, Wang X, Feng S, Xia X, Zhang H, Rahaman A, et al. From the perspective of Traditional Chinese Medicine: Treatment of mental disorders in COVID-19 survivors. Biomed Pharmacother 2020;132:110810.**

**[8] Po KK, Leung JW, Chan JN, Fung TK, Sánchez-Vidaña DI, Sin EL, et al. Protective effect of Lycium Barbarum polysaccharides on dextromethorphan-induced mood impairment and neurogenesis suppression. Brain Res Bull 2017;134:10-7.**

**[9] Fang S, Dong L, Liu L, Guo J, Zhao L, Zhang J, et al. HERB: a high-throughput experiment- and reference-guided database of traditional Chinese medicine. Nucleic Acids Res 2021;49:D1197-d206.**

**[10] Zhang S, He F, Chen X, Ding K. Isolation and structural characterization of a pectin from Lycium ruthenicum Murr and its anti-pancreatic ductal adenocarcinoma cell activity. Carbohydr Polym 2019;223:115104.**

**[11] Cao YL, Li YL, Fan YF, Li Z, Yoshida K, Wang JY, et al. Wolfberry genomes and the evolution of Lycium (Solanaceae). Commun Biol 2021;4:671.**

**[12] Chen J, Liu X, Zhu L, Wang Y. Nuclear genome size estimation and karyotype analysis of Lycium species (Solanaceae). Scientia Horticulturae 2013;151:46-50.**

**[13] Giri MK, Swain S, Gautam JK, Singh S, Singh N, Bhattacharjee L, et al. The Arabidopsis thaliana At4g13040 gene, a unique member of the AP2/EREBP family, is a positive regulator for salicylic acid accumulation and basal defense against bacterial pathogens. J Plant Physiol 2014;171:860-7.**

**[14] Michaels SD, Ditta G, Gustafson-Brown C, Pelaz S, Yanofsky M, Amasino RM. AGL24 acts as a promoter of flowering in Arabidopsis and is positively regulated by vernalization. Plant J 2003;33:867-74.**

**[15] Schauser L, Roussis A, Stiller J, Stougaard J. A plant regulator controlling development of symbiotic root nodules. Nature 1999;402:191-5.**

**[16] Hou X, Zhou J, Liu C, Liu L, Shen L, Yu H. Nuclear factor Y-mediated H3K27me3 demethylation of the SOC1 locus orchestrates flowering responses of Arabidopsis. Nat Commun 2014;5:4601.**

**[17] Ru L, He Y, Zhu Z, Patrick JW, Ruan YL. Integrating Sugar Metabolism With Transport: Elevation of Endogenous Cell Wall Invertase Activity Up-Regulates SlHT2 and SlSWEET12c Expression for Early Fruit Development in Tomato. Front Genet 2020;11:592596.**

**[18] Breia R, Conde A, Badim H, Fortes AM, Gerós H, Granell A. Plant SWEETs: from sugar transport to plant-pathogen interaction and more unexpected physiological roles. Plant Physiol 2021;186:836-52.**

**[19] Chen LQ, Hou BH, Lalonde S, Takanaga H, Hartung ML, Qu XQ, et al. Sugar transporters for intercellular exchange and nutrition of pathogens. Nature 2010;468:527-32.**

**[20] Eom JS, Chen LQ, Sosso D, Julius BT, Lin IW, Qu XQ, et al. SWEETs, transporters for intracellular and intercellular sugar translocation. Curr Opin Plant Biol 2015;25:53-62.**

**[21] Li Y, Feng S, Ma S, Sui X, Zhang Z. Spatiotemporal Expression and Substrate Specificity Analysis of the Cucumber SWEET Gene Family. Front Plant Sci 2017;8:1855.**

**[22] Patil G, Valliyodan B, Deshmukh R, Prince S, Nicander B, Zhao M, et al. Soybean (Glycine max) SWEET gene family: insights through comparative genomics, transcriptome profiling and whole genome re-sequence analysis. BMC Genomics 2015;16:520.**

**[23] Zhang W, Wang S, Yu F, Tang J, Shan X, Bao K, et al. Genome-wide characterization and expression profiling of SWEET genes in cabbage (Brassica oleracea var. capitata L.) reveal their roles in chilling and clubroot disease responses. BMC Genomics 2019;20:93.**

**[24] Feng CY, Han JX, Han XX, Jiang J. Genome-wide identification, phylogeny, and expression analysis of the SWEET gene family in tomato. Gene 2015;573:261-72.**

**[25] Manck-Götzenberger J, Requena N. Arbuscular mycorrhiza Symbiosis Induces a Major Transcriptional Reprogramming of the Potato SWEET Sugar Transporter Family. Front Plant Sci 2016;7:487.**

**[26] Ko HY, Ho LH, Neuhaus HE, Guo WJ. Transporter SlSWEET15 unloads sucrose from phloem and seed coat for fruit and seed development in tomato. Plant Physiol 2021;187:2230-45.**

**[27] Zhang X, Feng C, Wang M, Li T, Liu X, Jiang J. Plasma membrane-localized SlSWEET7a and SlSWEET14 regulate sugar transport and storage in tomato fruits. Hortic Res 2021;8:186.**

**[28] Pan L, Guo Q, Chai S, Cheng Y, Ruan M, Ye Q, et al. Evolutionary Conservation and Expression Patterns of Neutral/Alkaline Invertases in Solanum. Biomolecules 2019;9.**

**[29] Veillet F, Gaillard C, Coutos-Thévenot P, La Camera S. Targeting the AtCWIN1 Gene to Explore the Role of Invertases in Sucrose Transport in Roots and during Botrytis cinerea Infection. Front Plant Sci 2016;7:1899.**

**[31] Jin Y, Ni DA, Ruan YL. Posttranslational elevation of cell wall invertase activity by silencing its inhibitor in tomato delays leaf senescence and increases seed weight and fruit hexose level. Plant Cell 2009;21:2072-89.**

**[32] Shen S, Ma S, Liu Y, Liao S, Li J, Wu L, et al. Cell Wall Invertase and Sugar Transporters Are Differentially Activated in Tomato Styles and Ovaries During Pollination and Fertilization. Front Plant Sci 2019;10:506.**

**[33] Lombard V, Golaconda Ramulu H, Drula E, Coutinho PM, Henrissat B. The carbohydrate-active enzymes database (CAZy) in 2013. Nucleic Acids Res 2014;42:D490-5.**

**[34] Zhang H, Yohe T, Huang L, Entwistle S, Wu P, Yang Z, et al. dbCAN2: a meta server for automated carbohydrate-active enzyme annotation. Nucleic Acids Res 2018;46:W95-w101.**

**[35] Langfelder P, Horvath S. WGCNA: an R package for weighted correlation network analysis. BMC Bioinformatics 2008;9:559.**

**[36] Kaczmarska A, Pieczywek PM, Cybulska J, Zdunek A. Structure and functionality of Rhamnogalacturonan I in the cell wall and in solution: A review. Carbohydr Polym 2022;278:118909.**

**[37] Wachananawat B, Kuroha T, Takenaka Y, Kajiura H, Naramoto S, Yokoyama R, et al. Diversity of Pectin Rhamnogalacturonan I Rhamnosyltransferases in Glycosyltransferase Family 106. Front Plant Sci 2020;11:997.**

**[38] Atmodjo MA, Sakuragi Y, Zhu X, Burrell AJ, Mohanty SS, Atwood JA, 3rd, et al. Galacturonosyltransferase (GAUT)1 and GAUT7 are the core of a plant cell wall pectin biosynthetic homogalacturonan:galacturonosyltransferase complex. Proc Natl Acad Sci U S A 2011;108:20225-30.**

**[39] Ridley BL, O'Neill MA, Mohnen D. Pectins: structure, biosynthesis, and oligogalacturonide-related signaling. Phytochemistry 2001;57:929-67.**

**[1] Gao Y, Wei Y, Wang Y, Gao F, Chen Z. Lycium Barbarum: A Traditional Chinese Herb and A Promising Anti-Aging Agent. Aging Dis 2017;8:778-91.**

**[2] Sun C, Chen X, Yang S, Jin C, Ding K, Chen C. LBP1C-2 from Lycium barbarum alleviated age-related bone loss by targeting BMPRIA/BMPRII/Noggin. Carbohydr Polym 2023;310:120725.**

**[3] Potterat O. Goji (Lycium barbarum and L. chinense): Phytochemistry, pharmacology and safety in the perspective of traditional uses and recent popularity. Planta Med 2010;76:7-19.**

**[4] Zhang XJ, Yu HY, Cai YJ, Ke M. Lycium barbarum polysaccharides inhibit proliferation and migration of bladder cancer cell lines BIU87 by suppressing Pi3K/AKT pathway. Oncotarget 2017;8:5936-42.**

**[5] Tang L, Bao S, Du Y, Jiang Z, Wuliji AO, Ren X, et al. Antioxidant effects of Lycium barbarum polysaccharides on photoreceptor degeneration in the light-exposed mouse retina. Biomed Pharmacother 2018;103:829-37.**

**[6] Zhang W, Zhang J, Ding D, Zhang L, Muehlmann LA, Deng SE, et al. Synthesis and antioxidant properties of Lycium barbarum polysaccharides capped selenium nanoparticles using tea extract. Artif Cells Nanomed Biotechnol 2018;46:1463-70.**

**[7] Ma K, Wang X, Feng S, Xia X, Zhang H, Rahaman A, et al. From the perspective of Traditional Chinese Medicine: Treatment of mental disorders in COVID-19 survivors. Biomed Pharmacother 2020;132:110810.**

**[8] Po KK, Leung JW, Chan JN, Fung TK, Sánchez-Vidaña DI, Sin EL, et al. Protective effect of Lycium Barbarum polysaccharides on dextromethorphan-induced mood impairment and neurogenesis suppression. Brain Res Bull 2017;134:10-7.**

**[9] Fang S, Dong L, Liu L, Guo J, Zhao L, Zhang J, et al. HERB: a high-throughput experiment- and reference-guided database of traditional Chinese medicine. Nucleic Acids Res 2021;49:D1197-d206.**

**[10] Zhang S, He F, Chen X, Ding K. Isolation and structural characterization of a pectin from Lycium ruthenicum Murr and its anti-pancreatic ductal adenocarcinoma cell activity. Carbohydr Polym 2019;223:115104.**

**[11] Cao YL, Li YL, Fan YF, Li Z, Yoshida K, Wang JY, et al. Wolfberry genomes and the evolution of Lycium (Solanaceae). Commun Biol 2021;4:671.**

**[12] Chen J, Liu X, Zhu L, Wang Y. Nuclear genome size estimation and karyotype analysis of Lycium species (Solanaceae). Scientia Horticulturae 2013;151:46-50.**

**[13] Giri MK, Swain S, Gautam JK, Singh S, Singh N, Bhattacharjee L, et al. The Arabidopsis thaliana At4g13040 gene, a unique member of the AP2/EREBP family, is a positive regulator for salicylic acid accumulation and basal defense against bacterial pathogens. J Plant Physiol 2014;171:860-7.**

**[14] Michaels SD, Ditta G, Gustafson-Brown C, Pelaz S, Yanofsky M, Amasino RM. AGL24 acts as a promoter of flowering in Arabidopsis and is positively regulated by vernalization. Plant J 2003;33:867-74.**

**[15] Schauser L, Roussis A, Stiller J, Stougaard J. A plant regulator controlling development of symbiotic root nodules. Nature 1999;402:191-5.**

**[16] Hou X, Zhou J, Liu C, Liu L, Shen L, Yu H. Nuclear factor Y-mediated H3K27me3 demethylation of the SOC1 locus orchestrates flowering responses of Arabidopsis. Nat Commun 2014;5:4601.**

**[17] Ru L, He Y, Zhu Z, Patrick JW, Ruan YL. Integrating Sugar Metabolism With Transport: Elevation of Endogenous Cell Wall Invertase Activity Up-Regulates SlHT2 and SlSWEET12c Expression for Early Fruit Development in Tomato. Front Genet 2020;11:592596.**

**[18] Breia R, Conde A, Badim H, Fortes AM, Gerós H, Granell A. Plant SWEETs: from sugar transport to plant-pathogen interaction and more unexpected physiological roles. Plant Physiol 2021;186:836-52.**

**[19] Chen LQ, Hou BH, Lalonde S, Takanaga H, Hartung ML, Qu XQ, et al. Sugar transporters for intercellular exchange and nutrition of pathogens. Nature 2010;468:527-32.**

**[20] Eom JS, Chen LQ, Sosso D, Julius BT, Lin IW, Qu XQ, et al. SWEETs, transporters for intracellular and intercellular sugar translocation. Curr Opin Plant Biol 2015;25:53-62.**

**[22] Patil G, Valliyodan B, Deshmukh R, Prince S, Nicander B, Zhao M, et al. Soybean (Glycine max) SWEET gene family: insights through comparative genomics, transcriptome profiling and whole genome re-sequence analysis. BMC Genomics 2015;16:520.**

**[23] Zhang W, Wang S, Yu F, Tang J, Shan X, Bao K, et al. Genome-wide characterization and expression profiling of SWEET genes in cabbage (Brassica oleracea var. capitata L.) reveal their roles in chilling and clubroot disease responses. BMC Genomics 2019;20:93.**

**[24] Feng CY, Han JX, Han XX, Jiang J. Genome-wide identification, phylogeny, and expression analysis of the SWEET gene family in tomato. Gene 2015;573:261-72.**

**[25] Manck-Götzenberger J, Requena N. Arbuscular mycorrhiza Symbiosis Induces a Major Transcriptional Reprogramming of the Potato SWEET Sugar Transporter Family. Front Plant Sci 2016;7:487.**

**[26] Ko HY, Ho LH, Neuhaus HE, Guo WJ. Transporter SlSWEET15 unloads sucrose from phloem and seed coat for fruit and seed development in tomato. Plant Physiol 2021;187:2230-45.**

**[27] Zhang X, Feng C, Wang M, Li T, Liu X, Jiang J. Plasma membrane-localized SlSWEET7a and SlSWEET14 regulate sugar transport and storage in tomato fruits. Hortic Res 2021;8:186.**

**[28] Pan L, Guo Q, Chai S, Cheng Y, Ruan M, Ye Q, et al. Evolutionary Conservation and Expression Patterns of Neutral/Alkaline Invertases in Solanum. Biomolecules 2019;9.**

**[29] Veillet F, Gaillard C, Coutos-Thévenot P, La Camera S. Targeting the AtCWIN1 Gene to Explore the Role of Invertases in Sucrose Transport in Roots and during Botrytis cinerea Infection. Front Plant Sci 2016;7:1899.**

**[30] Wan H, Wu L, Yang Y, Zhou G, Ruan YL. Evolution of Sucrose Metabolism: The Dichotomy of Invertases and Beyond. Trends Plant Sci 2018;23:163-77.**

**[31] Jin Y, Ni DA, Ruan YL. Posttranslational elevation of cell wall invertase activity by silencing its inhibitor in tomato delays leaf senescence and increases seed weight and fruit hexose level. Plant Cell 2009;21:2072-89.**

**[33] Lombard V, Golaconda Ramulu H, Drula E, Coutinho PM, Henrissat B. The carbohydrate-active enzymes database (CAZy) in 2013. Nucleic Acids Res 2014;42:D490-5.**

**[34] Zhang H, Yohe T, Huang L, Entwistle S, Wu P, Yang Z, et al. dbCAN2: a meta server for automated carbohydrate-active enzyme annotation. Nucleic Acids Res 2018;46:W95-w101.**

**[35] Langfelder P, Horvath S. WGCNA: an R package for weighted correlation network analysis. BMC Bioinformatics 2008;9:559.**

**[36] Kaczmarska A, Pieczywek PM, Cybulska J, Zdunek A. Structure and functionality of Rhamnogalacturonan I in the cell wall and in solution: A review. Carbohydr Polym 2022;278:118909.**

**[37] Wachananawat B, Kuroha T, Takenaka Y, Kajiura H, Naramoto S, Yokoyama R, et al. Diversity of Pectin Rhamnogalacturonan I Rhamnosyltransferases in Glycosyltransferase Family 106. Front Plant Sci 2020;11:997.**

**[38] Atmodjo MA, Sakuragi Y, Zhu X, Burrell AJ, Mohanty SS, Atwood JA, 3rd, et al. Galacturonosyltransferase (GAUT)1 and GAUT7 are the core of a plant cell wall pectin biosynthetic homogalacturonan:galacturonosyltransferase complex. Proc Natl Acad Sci U S A 2011;108:20225-30.**

**[39] Ridley BL, O'Neill MA, Mohnen D. Pectins: structure, biosynthesis, and oligogalacturonide-related signaling. Phytochemistry 2001;57:929-67.**

**[40] Philippe F, Pelloux J, Rayon C. Plant pectin acetylesterase structure and function: new insights from bioinformatic analysis. BMC Genomics 2017;18:456.**

**[1] Gao Y, Wei Y, Wang Y, Gao F, Chen Z. Lycium Barbarum: A Traditional Chinese Herb and A Promising Anti-Aging Agent. Aging Dis 2017;8:778-91.**

**[2] Sun C, Chen X, Yang S, Jin C, Ding K, Chen C. LBP1C-2 from Lycium barbarum alleviated age-related bone loss by targeting BMPRIA/BMPRII/Noggin. Carbohydr Polym 2023;310:120725.**

**[3] Potterat O. Goji (Lycium barbarum and L. chinense): Phytochemistry, pharmacology and safety in the perspective of traditional uses and recent popularity. Planta Med 2010;76:7-19.**

**[4] Zhang XJ, Yu HY, Cai YJ, Ke M. Lycium barbarum polysaccharides inhibit proliferation and migration of bladder cancer cell lines BIU87 by suppressing Pi3K/AKT pathway. Oncotarget 2017;8:5936-42.**

**[5] Tang L, Bao S, Du Y, Jiang Z, Wuliji AO, Ren X, et al. Antioxidant effects of Lycium barbarum polysaccharides on photoreceptor degeneration in the light-exposed mouse retina. Biomed Pharmacother 2018;103:829-37.**

**[6] Zhang W, Zhang J, Ding D, Zhang L, Muehlmann LA, Deng SE, et al. Synthesis and antioxidant properties of Lycium barbarum polysaccharides capped selenium nanoparticles using tea extract. Artif Cells Nanomed Biotechnol 2018;46:1463-70.**

**[7] Ma K, Wang X, Feng S, Xia X, Zhang H, Rahaman A, et al. From the perspective of Traditional Chinese Medicine: Treatment of mental disorders in COVID-19 survivors. Biomed Pharmacother 2020;132:110810.**

**[8] Po KK, Leung JW, Chan JN, Fung TK, Sánchez-Vidaña DI, Sin EL, et al. Protective effect of Lycium Barbarum polysaccharides on dextromethorphan-induced mood impairment and neurogenesis suppression. Brain Res Bull 2017;134:10-7.**

**[9] Fang S, Dong L, Liu L, Guo J, Zhao L, Zhang J, et al. HERB: a high-throughput experiment- and reference-guided database of traditional Chinese medicine. Nucleic Acids Res 2021;49:D1197-d206.**

**[10] Zhang S, He F, Chen X, Ding K. Isolation and structural characterization of a pectin from Lycium ruthenicum Murr and its anti-pancreatic ductal adenocarcinoma cell activity. Carbohydr Polym 2019;223:115104.**

**[11] Cao YL, Li YL, Fan YF, Li Z, Yoshida K, Wang JY, et al. Wolfberry genomes and the evolution of Lycium (Solanaceae). Commun Biol 2021;4:671.**

**[12] Chen J, Liu X, Zhu L, Wang Y. Nuclear genome size estimation and karyotype analysis of Lycium species (Solanaceae). Scientia Horticulturae 2013;151:46-50.**

**[13] Giri MK, Swain S, Gautam JK, Singh S, Singh N, Bhattacharjee L, et al. The Arabidopsis thaliana At4g13040 gene, a unique member of the AP2/EREBP family, is a positive regulator for salicylic acid accumulation and basal defense against bacterial pathogens. J Plant Physiol 2014;171:860-7.**

**[14] Michaels SD, Ditta G, Gustafson-Brown C, Pelaz S, Yanofsky M, Amasino RM. AGL24 acts as a promoter of flowering in Arabidopsis and is positively regulated by vernalization. Plant J 2003;33:867-74.**

**[15] Schauser L, Roussis A, Stiller J, Stougaard J. A plant regulator controlling development of symbiotic root nodules. Nature 1999;402:191-5.**

**[16] Hou X, Zhou J, Liu C, Liu L, Shen L, Yu H. Nuclear factor Y-mediated H3K27me3 demethylation of the SOC1 locus orchestrates flowering responses of Arabidopsis. Nat Commun 2014;5:4601.**

**[17] Ru L, He Y, Zhu Z, Patrick JW, Ruan YL. Integrating Sugar Metabolism With Transport: Elevation of Endogenous Cell Wall Invertase Activity Up-Regulates SlHT2 and SlSWEET12c Expression for Early Fruit Development in Tomato. Front Genet 2020;11:592596.**

**[18] Breia R, Conde A, Badim H, Fortes AM, Gerós H, Granell A. Plant SWEETs: from sugar transport to plant-pathogen interaction and more unexpected physiological roles. Plant Physiol 2021;186:836-52.**

**[19] Chen LQ, Hou BH, Lalonde S, Takanaga H, Hartung ML, Qu XQ, et al. Sugar transporters for intercellular exchange and nutrition of pathogens. Nature 2010;468:527-32.**

**[20] Eom JS, Chen LQ, Sosso D, Julius BT, Lin IW, Qu XQ, et al. SWEETs, transporters for intracellular and intercellular sugar translocation. Curr Opin Plant Biol 2015;25:53-62.**

**[21] Li Y, Feng S, Ma S, Sui X, Zhang Z. Spatiotemporal Expression and Substrate Specificity Analysis of the Cucumber SWEET Gene Family. Front Plant Sci 2017;8:1855.**

**[22] Patil G, Valliyodan B, Deshmukh R, Prince S, Nicander B, Zhao M, et al. Soybean (Glycine max) SWEET gene family: insights through comparative genomics, transcriptome profiling and whole genome re-sequence analysis. BMC Genomics 2015;16:520.**

**[23] Zhang W, Wang S, Yu F, Tang J, Shan X, Bao K, et al. Genome-wide characterization and expression profiling of SWEET genes in cabbage (Brassica oleracea var. capitata L.) reveal their roles in chilling and clubroot disease responses. BMC Genomics 2019;20:93.**

**[24] Feng CY, Han JX, Han XX, Jiang J. Genome-wide identification, phylogeny, and expression analysis of the SWEET gene family in tomato. Gene 2015;573:261-72.**

**[25] Manck-Götzenberger J, Requena N. Arbuscular mycorrhiza Symbiosis Induces a Major Transcriptional Reprogramming of the Potato SWEET Sugar Transporter Family. Front Plant Sci 2016;7:487.**

**[26] Ko HY, Ho LH, Neuhaus HE, Guo WJ. Transporter SlSWEET15 unloads sucrose from phloem and seed coat for fruit and seed development in tomato. Plant Physiol 2021;187:2230-45.**

**[27] Zhang X, Feng C, Wang M, Li T, Liu X, Jiang J. Plasma membrane-localized SlSWEET7a and SlSWEET14 regulate sugar transport and storage in tomato fruits. Hortic Res 2021;8:186.**

**[28] Pan L, Guo Q, Chai S, Cheng Y, Ruan M, Ye Q, et al. Evolutionary Conservation and Expression Patterns of Neutral/Alkaline Invertases in Solanum. Biomolecules 2019;9.**

**[29] Veillet F, Gaillard C, Coutos-Thévenot P, La Camera S. Targeting the AtCWIN1 Gene to Explore the Role of Invertases in Sucrose Transport in Roots and during Botrytis cinerea Infection. Front Plant Sci 2016;7:1899.**

**[30] Wan H, Wu L, Yang Y, Zhou G, Ruan YL. Evolution of Sucrose Metabolism: The Dichotomy of Invertases and Beyond. Trends Plant Sci 2018;23:163-77.**

**[31] Jin Y, Ni DA, Ruan YL. Posttranslational elevation of cell wall invertase activity by silencing its inhibitor in tomato delays leaf senescence and increases seed weight and fruit hexose level. Plant Cell 2009;21:2072-89.**

**[32] Shen S, Ma S, Liu Y, Liao S, Li J, Wu L, et al. Cell Wall Invertase and Sugar Transporters Are Differentially Activated in Tomato Styles and Ovaries During Pollination and Fertilization. Front Plant Sci 2019;10:506.**

**[33] Lombard V, Golaconda Ramulu H, Drula E, Coutinho PM, Henrissat B. The carbohydrate-active enzymes database (CAZy) in 2013. Nucleic Acids Res 2014;42:D490-5.**

**[34] Zhang H, Yohe T, Huang L, Entwistle S, Wu P, Yang Z, et al. dbCAN2: a meta server for automated carbohydrate-active enzyme annotation. Nucleic Acids Res 2018;46:W95-w101.**

**[36] Kaczmarska A, Pieczywek PM, Cybulska J, Zdunek A. Structure and functionality of Rhamnogalacturonan I in the cell wall and in solution: A review. Carbohydr Polym 2022;278:118909.**

**[37] Wachananawat B, Kuroha T, Takenaka Y, Kajiura H, Naramoto S, Yokoyama R, et al. Diversity of Pectin Rhamnogalacturonan I Rhamnosyltransferases in Glycosyltransferase Family 106. Front Plant Sci 2020;11:997.**

**[38] Atmodjo MA, Sakuragi Y, Zhu X, Burrell AJ, Mohanty SS, Atwood JA, 3rd, et al. Galacturonosyltransferase (GAUT)1 and GAUT7 are the core of a plant cell wall pectin biosynthetic homogalacturonan:galacturonosyltransferase complex. Proc Natl Acad Sci U S A 2011;108:20225-30.**

**[39] Ridley BL, O'Neill MA, Mohnen D. Pectins: structure, biosynthesis, and oligogalacturonide-related signaling. Phytochemistry 2001;57:929-67.**

**[40] Philippe F, Pelloux J, Rayon C. Plant pectin acetylesterase structure and function: new insights from bioinformatic analysis. BMC Genomics 2017;18:456.**

**[41] Carpita NC, Gibeaut DM. Structural models of primary cell walls in flowering plants: consistency of molecular structure with the physical properties of the walls during growth. Plant J 1993;3:1-30.**

**[1] Gao Y, Wei Y, Wang Y, Gao F, Chen Z. Lycium Barbarum: A Traditional Chinese Herb and A Promising Anti-Aging Agent. Aging Dis 2017;8:778-91.**

**[2] Sun C, Chen X, Yang S, Jin C, Ding K, Chen C. LBP1C-2 from Lycium barbarum alleviated age-related bone loss by targeting BMPRIA/BMPRII/Noggin. Carbohydr Polym 2023;310:120725.**

**[3] Potterat O. Goji (Lycium barbarum and L. chinense): Phytochemistry, pharmacology and safety in the perspective of traditional uses and recent popularity. Planta Med 2010;76:7-19.**

**[4] Zhang XJ, Yu HY, Cai YJ, Ke M. Lycium barbarum polysaccharides inhibit proliferation and migration of bladder cancer cell lines BIU87 by suppressing Pi3K/AKT pathway. Oncotarget 2017;8:5936-42.**

**[5] Tang L, Bao S, Du Y, Jiang Z, Wuliji AO, Ren X, et al. Antioxidant effects of Lycium barbarum polysaccharides on photoreceptor degeneration in the light-exposed mouse retina. Biomed Pharmacother 2018;103:829-37.**

**[6] Zhang W, Zhang J, Ding D, Zhang L, Muehlmann LA, Deng SE, et al. Synthesis and antioxidant properties of Lycium barbarum polysaccharides capped selenium nanoparticles using tea extract. Artif Cells Nanomed Biotechnol 2018;46:1463-70.**

**[7] Ma K, Wang X, Feng S, Xia X, Zhang H, Rahaman A, et al. From the perspective of Traditional Chinese Medicine: Treatment of mental disorders in COVID-19 survivors. Biomed Pharmacother 2020;132:110810.**

**[8] Po KK, Leung JW, Chan JN, Fung TK, Sánchez-Vidaña DI, Sin EL, et al. Protective effect of Lycium Barbarum polysaccharides on dextromethorphan-induced mood impairment and neurogenesis suppression. Brain Res Bull 2017;134:10-7.**

**[9] Fang S, Dong L, Liu L, Guo J, Zhao L, Zhang J, et al. HERB: a high-throughput experiment- and reference-guided database of traditional Chinese medicine. Nucleic Acids Res 2021;49:D1197-d206.**

**[10] Zhang S, He F, Chen X, Ding K. Isolation and structural characterization of a pectin from Lycium ruthenicum Murr and its anti-pancreatic ductal adenocarcinoma cell activity. Carbohydr Polym 2019;223:115104.**

**[11] Cao YL, Li YL, Fan YF, Li Z, Yoshida K, Wang JY, et al. Wolfberry genomes and the evolution of Lycium (Solanaceae). Commun Biol 2021;4:671.**

**[12] Chen J, Liu X, Zhu L, Wang Y. Nuclear genome size estimation and karyotype analysis of Lycium species (Solanaceae). Scientia Horticulturae 2013;151:46-50.**

**[13] Giri MK, Swain S, Gautam JK, Singh S, Singh N, Bhattacharjee L, et al. The Arabidopsis thaliana At4g13040 gene, a unique member of the AP2/EREBP family, is a positive regulator for salicylic acid accumulation and basal defense against bacterial pathogens. J Plant Physiol 2014;171:860-7.**

**[14] Michaels SD, Ditta G, Gustafson-Brown C, Pelaz S, Yanofsky M, Amasino RM. AGL24 acts as a promoter of flowering in Arabidopsis and is positively regulated by vernalization. Plant J 2003;33:867-74.**

**[15] Schauser L, Roussis A, Stiller J, Stougaard J. A plant regulator controlling development of symbiotic root nodules. Nature 1999;402:191-5.**

**[16] Hou X, Zhou J, Liu C, Liu L, Shen L, Yu H. Nuclear factor Y-mediated H3K27me3 demethylation of the SOC1 locus orchestrates flowering responses of Arabidopsis. Nat Commun 2014;5:4601.**

**[17] Ru L, He Y, Zhu Z, Patrick JW, Ruan YL. Integrating Sugar Metabolism With Transport: Elevation of Endogenous Cell Wall Invertase Activity Up-Regulates SlHT2 and SlSWEET12c Expression for Early Fruit Development in Tomato. Front Genet 2020;11:592596.**

**[18] Breia R, Conde A, Badim H, Fortes AM, Gerós H, Granell A. Plant SWEETs: from sugar transport to plant-pathogen interaction and more unexpected physiological roles. Plant Physiol 2021;186:836-52.**

**[19] Chen LQ, Hou BH, Lalonde S, Takanaga H, Hartung ML, Qu XQ, et al. Sugar transporters for intercellular exchange and nutrition of pathogens. Nature 2010;468:527-32.**

**[20] Eom JS, Chen LQ, Sosso D, Julius BT, Lin IW, Qu XQ, et al. SWEETs, transporters for intracellular and intercellular sugar translocation. Curr Opin Plant Biol 2015;25:53-62.**

**[21] Li Y, Feng S, Ma S, Sui X, Zhang Z. Spatiotemporal Expression and Substrate Specificity Analysis of the Cucumber SWEET Gene Family. Front Plant Sci 2017;8:1855.**

**[22] Patil G, Valliyodan B, Deshmukh R, Prince S, Nicander B, Zhao M, et al. Soybean (Glycine max) SWEET gene family: insights through comparative genomics, transcriptome profiling and whole genome re-sequence analysis. BMC Genomics 2015;16:520.**

**[23] Zhang W, Wang S, Yu F, Tang J, Shan X, Bao K, et al. Genome-wide characterization and expression profiling of SWEET genes in cabbage (Brassica oleracea var. capitata L.) reveal their roles in chilling and clubroot disease responses. BMC Genomics 2019;20:93.**

**[24] Feng CY, Han JX, Han XX, Jiang J. Genome-wide identification, phylogeny, and expression analysis of the SWEET gene family in tomato. Gene 2015;573:261-72.**

**[25] Manck-Götzenberger J, Requena N. Arbuscular mycorrhiza Symbiosis Induces a Major Transcriptional Reprogramming of the Potato SWEET Sugar Transporter Family. Front Plant Sci 2016;7:487.**

**[26] Ko HY, Ho LH, Neuhaus HE, Guo WJ. Transporter SlSWEET15 unloads sucrose from phloem and seed coat for fruit and seed development in tomato. Plant Physiol 2021;187:2230-45.**

**[27] Zhang X, Feng C, Wang M, Li T, Liu X, Jiang J. Plasma membrane-localized SlSWEET7a and SlSWEET14 regulate sugar transport and storage in tomato fruits. Hortic Res 2021;8:186.**

**[28] Pan L, Guo Q, Chai S, Cheng Y, Ruan M, Ye Q, et al. Evolutionary Conservation and Expression Patterns of Neutral/Alkaline Invertases in Solanum. Biomolecules 2019;9.**

**[29] Veillet F, Gaillard C, Coutos-Thévenot P, La Camera S. Targeting the AtCWIN1 Gene to Explore the Role of Invertases in Sucrose Transport in Roots and during Botrytis cinerea Infection. Front Plant Sci 2016;7:1899.**

**[30] Wan H, Wu L, Yang Y, Zhou G, Ruan YL. Evolution of Sucrose Metabolism: The Dichotomy of Invertases and Beyond. Trends Plant Sci 2018;23:163-77.**

**[31] Jin Y, Ni DA, Ruan YL. Posttranslational elevation of cell wall invertase activity by silencing its inhibitor in tomato delays leaf senescence and increases seed weight and fruit hexose level. Plant Cell 2009;21:2072-89.**

**[32] Shen S, Ma S, Liu Y, Liao S, Li J, Wu L, et al. Cell Wall Invertase and Sugar Transporters Are Differentially Activated in Tomato Styles and Ovaries During Pollination and Fertilization. Front Plant Sci 2019;10:506.**

**[33] Lombard V, Golaconda Ramulu H, Drula E, Coutinho PM, Henrissat B. The carbohydrate-active enzymes database (CAZy) in 2013. Nucleic Acids Res 2014;42:D490-5.**

**[35] Langfelder P, Horvath S. WGCNA: an R package for weighted correlation network analysis. BMC Bioinformatics 2008;9:559.**

**[36] Kaczmarska A, Pieczywek PM, Cybulska J, Zdunek A. Structure and functionality of Rhamnogalacturonan I in the cell wall and in solution: A review. Carbohydr Polym 2022;278:118909.**

**[37] Wachananawat B, Kuroha T, Takenaka Y, Kajiura H, Naramoto S, Yokoyama R, et al. Diversity of Pectin Rhamnogalacturonan I Rhamnosyltransferases in Glycosyltransferase Family 106. Front Plant Sci 2020;11:997.**

**[38] Atmodjo MA, Sakuragi Y, Zhu X, Burrell AJ, Mohanty SS, Atwood JA, 3rd, et al. Galacturonosyltransferase (GAUT)1 and GAUT7 are the core of a plant cell wall pectin biosynthetic homogalacturonan:galacturonosyltransferase complex. Proc Natl Acad Sci U S A 2011;108:20225-30.**

**[39] Ridley BL, O'Neill MA, Mohnen D. Pectins: structure, biosynthesis, and oligogalacturonide-related signaling. Phytochemistry 2001;57:929-67.**

**[40] Philippe F, Pelloux J, Rayon C. Plant pectin acetylesterase structure and function: new insights from bioinformatic analysis. BMC Genomics 2017;18:456.**

**[41] Carpita NC, Gibeaut DM. Structural models of primary cell walls in flowering plants: consistency of molecular structure with the physical properties of the walls during growth. Plant J 1993;3:1-30.**

**[42] Mohnen D. Pectin structure and biosynthesis. Curr Opin Plant Biol 2008;11:266-77.**

**[1] Gao Y, Wei Y, Wang Y, Gao F, Chen Z. Lycium Barbarum: A Traditional Chinese Herb and A Promising Anti-Aging Agent. Aging Dis 2017;8:778-91.**

**[2] Sun C, Chen X, Yang S, Jin C, Ding K, Chen C. LBP1C-2 from Lycium barbarum alleviated age-related bone loss by targeting BMPRIA/BMPRII/Noggin. Carbohydr Polym 2023;310:120725.**

**[3] Potterat O. Goji (Lycium barbarum and L. chinense): Phytochemistry, pharmacology and safety in the perspective of traditional uses and recent popularity. Planta Med 2010;76:7-19.**

**[4] Zhang XJ, Yu HY, Cai YJ, Ke M. Lycium barbarum polysaccharides inhibit proliferation and migration of bladder cancer cell lines BIU87 by suppressing Pi3K/AKT pathway. Oncotarget 2017;8:5936-42.**

**[5] Tang L, Bao S, Du Y, Jiang Z, Wuliji AO, Ren X, et al. Antioxidant effects of Lycium barbarum polysaccharides on photoreceptor degeneration in the light-exposed mouse retina. Biomed Pharmacother 2018;103:829-37.**

**[6] Zhang W, Zhang J, Ding D, Zhang L, Muehlmann LA, Deng SE, et al. Synthesis and antioxidant properties of Lycium barbarum polysaccharides capped selenium nanoparticles using tea extract. Artif Cells Nanomed Biotechnol 2018;46:1463-70.**

**[7] Ma K, Wang X, Feng S, Xia X, Zhang H, Rahaman A, et al. From the perspective of Traditional Chinese Medicine: Treatment of mental disorders in COVID-19 survivors. Biomed Pharmacother 2020;132:110810.**

**[8] Po KK, Leung JW, Chan JN, Fung TK, Sánchez-Vidaña DI, Sin EL, et al. Protective effect of Lycium Barbarum polysaccharides on dextromethorphan-induced mood impairment and neurogenesis suppression. Brain Res Bull 2017;134:10-7.**

**[9] Fang S, Dong L, Liu L, Guo J, Zhao L, Zhang J, et al. HERB: a high-throughput experiment- and reference-guided database of traditional Chinese medicine. Nucleic Acids Res 2021;49:D1197-d206.**

**[10] Zhang S, He F, Chen X, Ding K. Isolation and structural characterization of a pectin from Lycium ruthenicum Murr and its anti-pancreatic ductal adenocarcinoma cell activity. Carbohydr Polym 2019;223:115104.**

**[11] Cao YL, Li YL, Fan YF, Li Z, Yoshida K, Wang JY, et al. Wolfberry genomes and the evolution of Lycium (Solanaceae). Commun Biol 2021;4:671.**

**[12] Chen J, Liu X, Zhu L, Wang Y. Nuclear genome size estimation and karyotype analysis of Lycium species (Solanaceae). Scientia Horticulturae 2013;151:46-50.**

**[13] Giri MK, Swain S, Gautam JK, Singh S, Singh N, Bhattacharjee L, et al. The Arabidopsis thaliana At4g13040 gene, a unique member of the AP2/EREBP family, is a positive regulator for salicylic acid accumulation and basal defense against bacterial pathogens. J Plant Physiol 2014;171:860-7.**

**[14] Michaels SD, Ditta G, Gustafson-Brown C, Pelaz S, Yanofsky M, Amasino RM. AGL24 acts as a promoter of flowering in Arabidopsis and is positively regulated by vernalization. Plant J 2003;33:867-74.**

**[15] Schauser L, Roussis A, Stiller J, Stougaard J. A plant regulator controlling development of symbiotic root nodules. Nature 1999;402:191-5.**

**[16] Hou X, Zhou J, Liu C, Liu L, Shen L, Yu H. Nuclear factor Y-mediated H3K27me3 demethylation of the SOC1 locus orchestrates flowering responses of Arabidopsis. Nat Commun 2014;5:4601.**

**[17] Ru L, He Y, Zhu Z, Patrick JW, Ruan YL. Integrating Sugar Metabolism With Transport: Elevation of Endogenous Cell Wall Invertase Activity Up-Regulates SlHT2 and SlSWEET12c Expression for Early Fruit Development in Tomato. Front Genet 2020;11:592596.**

**[18] Breia R, Conde A, Badim H, Fortes AM, Gerós H, Granell A. Plant SWEETs: from sugar transport to plant-pathogen interaction and more unexpected physiological roles. Plant Physiol 2021;186:836-52.**

**[19] Chen LQ, Hou BH, Lalonde S, Takanaga H, Hartung ML, Qu XQ, et al. Sugar transporters for intercellular exchange and nutrition of pathogens. Nature 2010;468:527-32.**

**[20] Eom JS, Chen LQ, Sosso D, Julius BT, Lin IW, Qu XQ, et al. SWEETs, transporters for intracellular and intercellular sugar translocation. Curr Opin Plant Biol 2015;25:53-62.**

**[21] Li Y, Feng S, Ma S, Sui X, Zhang Z. Spatiotemporal Expression and Substrate Specificity Analysis of the Cucumber SWEET Gene Family. Front Plant Sci 2017;8:1855.**

**[22] Patil G, Valliyodan B, Deshmukh R, Prince S, Nicander B, Zhao M, et al. Soybean (Glycine max) SWEET gene family: insights through comparative genomics, transcriptome profiling and whole genome re-sequence analysis. BMC Genomics 2015;16:520.**

**[23] Zhang W, Wang S, Yu F, Tang J, Shan X, Bao K, et al. Genome-wide characterization and expression profiling of SWEET genes in cabbage (Brassica oleracea var. capitata L.) reveal their roles in chilling and clubroot disease responses. BMC Genomics 2019;20:93.**

**[24] Feng CY, Han JX, Han XX, Jiang J. Genome-wide identification, phylogeny, and expression analysis of the SWEET gene family in tomato. Gene 2015;573:261-72.**

**[25] Manck-Götzenberger J, Requena N. Arbuscular mycorrhiza Symbiosis Induces a Major Transcriptional Reprogramming of the Potato SWEET Sugar Transporter Family. Front Plant Sci 2016;7:487.**

**[26] Ko HY, Ho LH, Neuhaus HE, Guo WJ. Transporter SlSWEET15 unloads sucrose from phloem and seed coat for fruit and seed development in tomato. Plant Physiol 2021;187:2230-45.**

**[27] Zhang X, Feng C, Wang M, Li T, Liu X, Jiang J. Plasma membrane-localized SlSWEET7a and SlSWEET14 regulate sugar transport and storage in tomato fruits. Hortic Res 2021;8:186.**

**[28] Pan L, Guo Q, Chai S, Cheng Y, Ruan M, Ye Q, et al. Evolutionary Conservation and Expression Patterns of Neutral/Alkaline Invertases in Solanum. Biomolecules 2019;9.**

**[29] Veillet F, Gaillard C, Coutos-Thévenot P, La Camera S. Targeting the AtCWIN1 Gene to Explore the Role of Invertases in Sucrose Transport in Roots and during Botrytis cinerea Infection. Front Plant Sci 2016;7:1899.**

**[30] Wan H, Wu L, Yang Y, Zhou G, Ruan YL. Evolution of Sucrose Metabolism: The Dichotomy of Invertases and Beyond. Trends Plant Sci 2018;23:163-77.**

**[31] Jin Y, Ni DA, Ruan YL. Posttranslational elevation of cell wall invertase activity by silencing its inhibitor in tomato delays leaf senescence and increases seed weight and fruit hexose level. Plant Cell 2009;21:2072-89.**

**[32] Shen S, Ma S, Liu Y, Liao S, Li J, Wu L, et al. Cell Wall Invertase and Sugar Transporters Are Differentially Activated in Tomato Styles and Ovaries During Pollination and Fertilization. Front Plant Sci 2019;10:506.**

**[33] Lombard V, Golaconda Ramulu H, Drula E, Coutinho PM, Henrissat B. The carbohydrate-active enzymes database (CAZy) in 2013. Nucleic Acids Res 2014;42:D490-5.**

**[34] Zhang H, Yohe T, Huang L, Entwistle S, Wu P, Yang Z, et al. dbCAN2: a meta server for automated carbohydrate-active enzyme annotation. Nucleic Acids Res 2018;46:W95-w101.**

**[35] Langfelder P, Horvath S. WGCNA: an R package for weighted correlation network analysis. BMC Bioinformatics 2008;9:559.**

**[36] Kaczmarska A, Pieczywek PM, Cybulska J, Zdunek A. Structure and functionality of Rhamnogalacturonan I in the cell wall and in solution: A review. Carbohydr Polym 2022;278:118909.**

**[37] Wachananawat B, Kuroha T, Takenaka Y, Kajiura H, Naramoto S, Yokoyama R, et al. Diversity of Pectin Rhamnogalacturonan I Rhamnosyltransferases in Glycosyltransferase Family 106. Front Plant Sci 2020;11:997.**

**[38] Atmodjo MA, Sakuragi Y, Zhu X, Burrell AJ, Mohanty SS, Atwood JA, 3rd, et al. Galacturonosyltransferase (GAUT)1 and GAUT7 are the core of a plant cell wall pectin biosynthetic homogalacturonan:galacturonosyltransferase complex. Proc Natl Acad Sci U S A 2011;108:20225-30.**

**[39] Ridley BL, O'Neill MA, Mohnen D. Pectins: structure, biosynthesis, and oligogalacturonide-related signaling. Phytochemistry 2001;57:929-67.**

**[40] Philippe F, Pelloux J, Rayon C. Plant pectin acetylesterase structure and function: new insights from bioinformatic analysis. BMC Genomics 2017;18:456.**

**[41] Carpita NC, Gibeaut DM. Structural models of primary cell walls in flowering plants: consistency of molecular structure with the physical properties of the walls during growth. Plant J 1993;3:1-30.**

**[42] Mohnen D. Pectin structure and biosynthesis. Curr Opin Plant Biol 2008;11:266-77.**

**[43] Harholt J, Jensen JK, Sørensen SO, Orfila C, Pauly M, Scheller HV. ARABINAN DEFICIENT 1 is a putative arabinosyltransferase involved in biosynthesis of pectic arabinan in Arabidopsis. Plant Physiol 2006;140:49-58.**

**[1] Gao Y, Wei Y, Wang Y, Gao F, Chen Z. Lycium Barbarum: A Traditional Chinese Herb and A Promising Anti-Aging Agent. Aging Dis 2017;8:778-91.**

**[2] Sun C, Chen X, Yang S, Jin C, Ding K, Chen C. LBP1C-2 from Lycium barbarum alleviated age-related bone loss by targeting BMPRIA/BMPRII/Noggin. Carbohydr Polym 2023;310:120725.**

**[3] Potterat O. Goji (Lycium barbarum and L. chinense): Phytochemistry, pharmacology and safety in the perspective of traditional uses and recent popularity. Planta Med 2010;76:7-19.**

**[4] Zhang XJ, Yu HY, Cai YJ, Ke M. Lycium barbarum polysaccharides inhibit proliferation and migration of bladder cancer cell lines BIU87 by suppressing Pi3K/AKT pathway. Oncotarget 2017;8:5936-42.**

**[5] Tang L, Bao S, Du Y, Jiang Z, Wuliji AO, Ren X, et al. Antioxidant effects of Lycium barbarum polysaccharides on photoreceptor degeneration in the light-exposed mouse retina. Biomed Pharmacother 2018;103:829-37.**

**[6] Zhang W, Zhang J, Ding D, Zhang L, Muehlmann LA, Deng SE, et al. Synthesis and antioxidant properties of Lycium barbarum polysaccharides capped selenium nanoparticles using tea extract. Artif Cells Nanomed Biotechnol 2018;46:1463-70.**

**[7] Ma K, Wang X, Feng S, Xia X, Zhang H, Rahaman A, et al. From the perspective of Traditional Chinese Medicine: Treatment of mental disorders in COVID-19 survivors. Biomed Pharmacother 2020;132:110810.**

**[8] Po KK, Leung JW, Chan JN, Fung TK, Sánchez-Vidaña DI, Sin EL, et al. Protective effect of Lycium Barbarum polysaccharides on dextromethorphan-induced mood impairment and neurogenesis suppression. Brain Res Bull 2017;134:10-7.**

**[9] Fang S, Dong L, Liu L, Guo J, Zhao L, Zhang J, et al. HERB: a high-throughput experiment- and reference-guided database of traditional Chinese medicine. Nucleic Acids Res 2021;49:D1197-d206.**

**[10] Zhang S, He F, Chen X, Ding K. Isolation and structural characterization of a pectin from Lycium ruthenicum Murr and its anti-pancreatic ductal adenocarcinoma cell activity. Carbohydr Polym 2019;223:115104.**

**[11] Cao YL, Li YL, Fan YF, Li Z, Yoshida K, Wang JY, et al. Wolfberry genomes and the evolution of Lycium (Solanaceae). Commun Biol 2021;4:671.**

**[12] Chen J, Liu X, Zhu L, Wang Y. Nuclear genome size estimation and karyotype analysis of Lycium species (Solanaceae). Scientia Horticulturae 2013;151:46-50.**

**[13] Giri MK, Swain S, Gautam JK, Singh S, Singh N, Bhattacharjee L, et al. The Arabidopsis thaliana At4g13040 gene, a unique member of the AP2/EREBP family, is a positive regulator for salicylic acid accumulation and basal defense against bacterial pathogens. J Plant Physiol 2014;171:860-7.**

**[14] Michaels SD, Ditta G, Gustafson-Brown C, Pelaz S, Yanofsky M, Amasino RM. AGL24 acts as a promoter of flowering in Arabidopsis and is positively regulated by vernalization. Plant J 2003;33:867-74.**

**[15] Schauser L, Roussis A, Stiller J, Stougaard J. A plant regulator controlling development of symbiotic root nodules. Nature 1999;402:191-5.**

**[16] Hou X, Zhou J, Liu C, Liu L, Shen L, Yu H. Nuclear factor Y-mediated H3K27me3 demethylation of the SOC1 locus orchestrates flowering responses of Arabidopsis. Nat Commun 2014;5:4601.**

**[17] Ru L, He Y, Zhu Z, Patrick JW, Ruan YL. Integrating Sugar Metabolism With Transport: Elevation of Endogenous Cell Wall Invertase Activity Up-Regulates SlHT2 and SlSWEET12c Expression for Early Fruit Development in Tomato. Front Genet 2020;11:592596.**

**[18] Breia R, Conde A, Badim H, Fortes AM, Gerós H, Granell A. Plant SWEETs: from sugar transport to plant-pathogen interaction and more unexpected physiological roles. Plant Physiol 2021;186:836-52.**

**[19] Chen LQ, Hou BH, Lalonde S, Takanaga H, Hartung ML, Qu XQ, et al. Sugar transporters for intercellular exchange and nutrition of pathogens. Nature 2010;468:527-32.**

**[20] Eom JS, Chen LQ, Sosso D, Julius BT, Lin IW, Qu XQ, et al. SWEETs, transporters for intracellular and intercellular sugar translocation. Curr Opin Plant Biol 2015;25:53-62.**

**[21] Li Y, Feng S, Ma S, Sui X, Zhang Z. Spatiotemporal Expression and Substrate Specificity Analysis of the Cucumber SWEET Gene Family. Front Plant Sci 2017;8:1855.**

**[22] Patil G, Valliyodan B, Deshmukh R, Prince S, Nicander B, Zhao M, et al. Soybean (Glycine max) SWEET gene family: insights through comparative genomics, transcriptome profiling and whole genome re-sequence analysis. BMC Genomics 2015;16:520.**

**[23] Zhang W, Wang S, Yu F, Tang J, Shan X, Bao K, et al. Genome-wide characterization and expression profiling of SWEET genes in cabbage (Brassica oleracea var. capitata L.) reveal their roles in chilling and clubroot disease responses. BMC Genomics 2019;20:93.**

**[24] Feng CY, Han JX, Han XX, Jiang J. Genome-wide identification, phylogeny, and expression analysis of the SWEET gene family in tomato. Gene 2015;573:261-72.**

**[25] Manck-Götzenberger J, Requena N. Arbuscular mycorrhiza Symbiosis Induces a Major Transcriptional Reprogramming of the Potato SWEET Sugar Transporter Family. Front Plant Sci 2016;7:487.**

**[26] Ko HY, Ho LH, Neuhaus HE, Guo WJ. Transporter SlSWEET15 unloads sucrose from phloem and seed coat for fruit and seed development in tomato. Plant Physiol 2021;187:2230-45.**

**[27] Zhang X, Feng C, Wang M, Li T, Liu X, Jiang J. Plasma membrane-localized SlSWEET7a and SlSWEET14 regulate sugar transport and storage in tomato fruits. Hortic Res 2021;8:186.**

**[28] Pan L, Guo Q, Chai S, Cheng Y, Ruan M, Ye Q, et al. Evolutionary Conservation and Expression Patterns of Neutral/Alkaline Invertases in Solanum. Biomolecules 2019;9.**

**[29] Veillet F, Gaillard C, Coutos-Thévenot P, La Camera S. Targeting the AtCWIN1 Gene to Explore the Role of Invertases in Sucrose Transport in Roots and during Botrytis cinerea Infection. Front Plant Sci 2016;7:1899.**

**[30] Wan H, Wu L, Yang Y, Zhou G, Ruan YL. Evolution of Sucrose Metabolism: The Dichotomy of Invertases and Beyond. Trends Plant Sci 2018;23:163-77.**

**[31] Jin Y, Ni DA, Ruan YL. Posttranslational elevation of cell wall invertase activity by silencing its inhibitor in tomato delays leaf senescence and increases seed weight and fruit hexose level. Plant Cell 2009;21:2072-89.**

**[32] Shen S, Ma S, Liu Y, Liao S, Li J, Wu L, et al. Cell Wall Invertase and Sugar Transporters Are Differentially Activated in Tomato Styles and Ovaries During Pollination and Fertilization. Front Plant Sci 2019;10:506.**

**[33] Lombard V, Golaconda Ramulu H, Drula E, Coutinho PM, Henrissat B. The carbohydrate-active enzymes database (CAZy) in 2013. Nucleic Acids Res 2014;42:D490-5.**

**[34] Zhang H, Yohe T, Huang L, Entwistle S, Wu P, Yang Z, et al. dbCAN2: a meta server for automated carbohydrate-active enzyme annotation. Nucleic Acids Res 2018;46:W95-w101.**

**[35] Langfelder P, Horvath S. WGCNA: an R package for weighted correlation network analysis. BMC Bioinformatics 2008;9:559.**

**[36] Kaczmarska A, Pieczywek PM, Cybulska J, Zdunek A. Structure and functionality of Rhamnogalacturonan I in the cell wall and in solution: A review. Carbohydr Polym 2022;278:118909.**

**[38] Atmodjo MA, Sakuragi Y, Zhu X, Burrell AJ, Mohanty SS, Atwood JA, 3rd, et al. Galacturonosyltransferase (GAUT)1 and GAUT7 are the core of a plant cell wall pectin biosynthetic homogalacturonan:galacturonosyltransferase complex. Proc Natl Acad Sci U S A 2011;108:20225-30.**

**[39] Ridley BL, O'Neill MA, Mohnen D. Pectins: structure, biosynthesis, and oligogalacturonide-related signaling. Phytochemistry 2001;57:929-67.**

**[40] Philippe F, Pelloux J, Rayon C. Plant pectin acetylesterase structure and function: new insights from bioinformatic analysis. BMC Genomics 2017;18:456.**

**[41] Carpita NC, Gibeaut DM. Structural models of primary cell walls in flowering plants: consistency of molecular structure with the physical properties of the walls during growth. Plant J 1993;3:1-30.**

**[42] Mohnen D. Pectin structure and biosynthesis. Curr Opin Plant Biol 2008;11:266-77.**

**[43] Harholt J, Jensen JK, Sørensen SO, Orfila C, Pauly M, Scheller HV. ARABINAN DEFICIENT 1 is a putative arabinosyltransferase involved in biosynthesis of pectic arabinan in Arabidopsis. Plant Physiol 2006;140:49-58.**

**[44] Redgwell RJ, Curti D, Wang J, Dobruchowska JM, Gerwig GJ, Kamerling JP, et al. Cell wall polysaccharides of Chinese Wolfberry (Lycium barbarum): Part 2. Characterisation of arabinogalactan-proteins. Carbohydrate Polymers 2011;84:1075-83.**

**[1] Gao Y, Wei Y, Wang Y, Gao F, Chen Z. Lycium Barbarum: A Traditional Chinese Herb and A Promising Anti-Aging Agent. Aging Dis 2017;8:778-91.**

**[2] Sun C, Chen X, Yang S, Jin C, Ding K, Chen C. LBP1C-2 from Lycium barbarum alleviated age-related bone loss by targeting BMPRIA/BMPRII/Noggin. Carbohydr Polym 2023;310:120725.**

**[3] Potterat O. Goji (Lycium barbarum and L. chinense): Phytochemistry, pharmacology and safety in the perspective of traditional uses and recent popularity. Planta Med 2010;76:7-19.**

**[4] Zhang XJ, Yu HY, Cai YJ, Ke M. Lycium barbarum polysaccharides inhibit proliferation and migration of bladder cancer cell lines BIU87 by suppressing Pi3K/AKT pathway. Oncotarget 2017;8:5936-42.**

**[5] Tang L, Bao S, Du Y, Jiang Z, Wuliji AO, Ren X, et al. Antioxidant effects of Lycium barbarum polysaccharides on photoreceptor degeneration in the light-exposed mouse retina. Biomed Pharmacother 2018;103:829-37.**

**[6] Zhang W, Zhang J, Ding D, Zhang L, Muehlmann LA, Deng SE, et al. Synthesis and antioxidant properties of Lycium barbarum polysaccharides capped selenium nanoparticles using tea extract. Artif Cells Nanomed Biotechnol 2018;46:1463-70.**

**[7] Ma K, Wang X, Feng S, Xia X, Zhang H, Rahaman A, et al. From the perspective of Traditional Chinese Medicine: Treatment of mental disorders in COVID-19 survivors. Biomed Pharmacother 2020;132:110810.**

**[8] Po KK, Leung JW, Chan JN, Fung TK, Sánchez-Vidaña DI, Sin EL, et al. Protective effect of Lycium Barbarum polysaccharides on dextromethorphan-induced mood impairment and neurogenesis suppression. Brain Res Bull 2017;134:10-7.**

**[9] Fang S, Dong L, Liu L, Guo J, Zhao L, Zhang J, et al. HERB: a high-throughput experiment- and reference-guided database of traditional Chinese medicine. Nucleic Acids Res 2021;49:D1197-d206.**

**[10] Zhang S, He F, Chen X, Ding K. Isolation and structural characterization of a pectin from Lycium ruthenicum Murr and its anti-pancreatic ductal adenocarcinoma cell activity. Carbohydr Polym 2019;223:115104.**

**[11] Cao YL, Li YL, Fan YF, Li Z, Yoshida K, Wang JY, et al. Wolfberry genomes and the evolution of Lycium (Solanaceae). Commun Biol 2021;4:671.**

**[12] Chen J, Liu X, Zhu L, Wang Y. Nuclear genome size estimation and karyotype analysis of Lycium species (Solanaceae). Scientia Horticulturae 2013;151:46-50.**

**[13] Giri MK, Swain S, Gautam JK, Singh S, Singh N, Bhattacharjee L, et al. The Arabidopsis thaliana At4g13040 gene, a unique member of the AP2/EREBP family, is a positive regulator for salicylic acid accumulation and basal defense against bacterial pathogens. J Plant Physiol 2014;171:860-7.**

**[14] Michaels SD, Ditta G, Gustafson-Brown C, Pelaz S, Yanofsky M, Amasino RM. AGL24 acts as a promoter of flowering in Arabidopsis and is positively regulated by vernalization. Plant J 2003;33:867-74.**

**[15] Schauser L, Roussis A, Stiller J, Stougaard J. A plant regulator controlling development of symbiotic root nodules. Nature 1999;402:191-5.**

**[16] Hou X, Zhou J, Liu C, Liu L, Shen L, Yu H. Nuclear factor Y-mediated H3K27me3 demethylation of the SOC1 locus orchestrates flowering responses of Arabidopsis. Nat Commun 2014;5:4601.**

**[17] Ru L, He Y, Zhu Z, Patrick JW, Ruan YL. Integrating Sugar Metabolism With Transport: Elevation of Endogenous Cell Wall Invertase Activity Up-Regulates SlHT2 and SlSWEET12c Expression for Early Fruit Development in Tomato. Front Genet 2020;11:592596.**

**[18] Breia R, Conde A, Badim H, Fortes AM, Gerós H, Granell A. Plant SWEETs: from sugar transport to plant-pathogen interaction and more unexpected physiological roles. Plant Physiol 2021;186:836-52.**

**[19] Chen LQ, Hou BH, Lalonde S, Takanaga H, Hartung ML, Qu XQ, et al. Sugar transporters for intercellular exchange and nutrition of pathogens. Nature 2010;468:527-32.**

**[20] Eom JS, Chen LQ, Sosso D, Julius BT, Lin IW, Qu XQ, et al. SWEETs, transporters for intracellular and intercellular sugar translocation. Curr Opin Plant Biol 2015;25:53-62.**

**[21] Li Y, Feng S, Ma S, Sui X, Zhang Z. Spatiotemporal Expression and Substrate Specificity Analysis of the Cucumber SWEET Gene Family. Front Plant Sci 2017;8:1855.**

**[22] Patil G, Valliyodan B, Deshmukh R, Prince S, Nicander B, Zhao M, et al. Soybean (Glycine max) SWEET gene family: insights through comparative genomics, transcriptome profiling and whole genome re-sequence analysis. BMC Genomics 2015;16:520.**

**[23] Zhang W, Wang S, Yu F, Tang J, Shan X, Bao K, et al. Genome-wide characterization and expression profiling of SWEET genes in cabbage (Brassica oleracea var. capitata L.) reveal their roles in chilling and clubroot disease responses. BMC Genomics 2019;20:93.**

**[24] Feng CY, Han JX, Han XX, Jiang J. Genome-wide identification, phylogeny, and expression analysis of the SWEET gene family in tomato. Gene 2015;573:261-72.**

**[25] Manck-Götzenberger J, Requena N. Arbuscular mycorrhiza Symbiosis Induces a Major Transcriptional Reprogramming of the Potato SWEET Sugar Transporter Family. Front Plant Sci 2016;7:487.**

**[26] Ko HY, Ho LH, Neuhaus HE, Guo WJ. Transporter SlSWEET15 unloads sucrose from phloem and seed coat for fruit and seed development in tomato. Plant Physiol 2021;187:2230-45.**

**[27] Zhang X, Feng C, Wang M, Li T, Liu X, Jiang J. Plasma membrane-localized SlSWEET7a and SlSWEET14 regulate sugar transport and storage in tomato fruits. Hortic Res 2021;8:186.**

**[28] Pan L, Guo Q, Chai S, Cheng Y, Ruan M, Ye Q, et al. Evolutionary Conservation and Expression Patterns of Neutral/Alkaline Invertases in Solanum. Biomolecules 2019;9.**

**[29] Veillet F, Gaillard C, Coutos-Thévenot P, La Camera S. Targeting the AtCWIN1 Gene to Explore the Role of Invertases in Sucrose Transport in Roots and during Botrytis cinerea Infection. Front Plant Sci 2016;7:1899.**

**[30] Wan H, Wu L, Yang Y, Zhou G, Ruan YL. Evolution of Sucrose Metabolism: The Dichotomy of Invertases and Beyond. Trends Plant Sci 2018;23:163-77.**

**[31] Jin Y, Ni DA, Ruan YL. Posttranslational elevation of cell wall invertase activity by silencing its inhibitor in tomato delays leaf senescence and increases seed weight and fruit hexose level. Plant Cell 2009;21:2072-89.**

**[32] Shen S, Ma S, Liu Y, Liao S, Li J, Wu L, et al. Cell Wall Invertase and Sugar Transporters Are Differentially Activated in Tomato Styles and Ovaries During Pollination and Fertilization. Front Plant Sci 2019;10:506.**

**[33] Lombard V, Golaconda Ramulu H, Drula E, Coutinho PM, Henrissat B. The carbohydrate-active enzymes database (CAZy) in 2013. Nucleic Acids Res 2014;42:D490-5.**

**[34] Zhang H, Yohe T, Huang L, Entwistle S, Wu P, Yang Z, et al. dbCAN2: a meta server for automated carbohydrate-active enzyme annotation. Nucleic Acids Res 2018;46:W95-w101.**

**[35] Langfelder P, Horvath S. WGCNA: an R package for weighted correlation network analysis. BMC Bioinformatics 2008;9:559.**

**[36] Kaczmarska A, Pieczywek PM, Cybulska J, Zdunek A. Structure and functionality of Rhamnogalacturonan I in the cell wall and in solution: A review. Carbohydr Polym 2022;278:118909.**

**[37] Wachananawat B, Kuroha T, Takenaka Y, Kajiura H, Naramoto S, Yokoyama R, et al. Diversity of Pectin Rhamnogalacturonan I Rhamnosyltransferases in Glycosyltransferase Family 106. Front Plant Sci 2020;11:997.**

**[38] Atmodjo MA, Sakuragi Y, Zhu X, Burrell AJ, Mohanty SS, Atwood JA, 3rd, et al. Galacturonosyltransferase (GAUT)1 and GAUT7 are the core of a plant cell wall pectin biosynthetic homogalacturonan:galacturonosyltransferase complex. Proc Natl Acad Sci U S A 2011;108:20225-30.**

**[39] Ridley BL, O'Neill MA, Mohnen D. Pectins: structure, biosynthesis, and oligogalacturonide-related signaling. Phytochemistry 2001;57:929-67.**

**[40] Philippe F, Pelloux J, Rayon C. Plant pectin acetylesterase structure and function: new insights from bioinformatic analysis. BMC Genomics 2017;18:456.**

**[41] Carpita NC, Gibeaut DM. Structural models of primary cell walls in flowering plants: consistency of molecular structure with the physical properties of the walls during growth. Plant J 1993;3:1-30.**

**[42] Mohnen D. Pectin structure and biosynthesis. Curr Opin Plant Biol 2008;11:266-77.**

**[43] Harholt J, Jensen JK, Sørensen SO, Orfila C, Pauly M, Scheller HV. ARABINAN DEFICIENT 1 is a putative arabinosyltransferase involved in biosynthesis of pectic arabinan in Arabidopsis. Plant Physiol 2006;140:49-58.**

**[44] Redgwell RJ, Curti D, Wang J, Dobruchowska JM, Gerwig GJ, Kamerling JP, et al. Cell wall polysaccharides of Chinese Wolfberry (Lycium barbarum): Part 2. Characterisation of arabinogalactan-proteins. Carbohydrate Polymers 2011;84:1075-83.**

**[45] Statello L, Guo CJ, Chen LL, Huarte M. Gene regulation by long non-coding RNAs and its biological functions. Nat Rev Mol Cell Biol 2021;22:96-118.**

**[1] Gao Y, Wei Y, Wang Y, Gao F, Chen Z. Lycium Barbarum: A Traditional Chinese Herb and A Promising Anti-Aging Agent. Aging Dis 2017;8:778-91.**

**[2] Sun C, Chen X, Yang S, Jin C, Ding K, Chen C. LBP1C-2 from Lycium barbarum alleviated age-related bone loss by targeting BMPRIA/BMPRII/Noggin. Carbohydr Polym 2023;310:120725.**

**[3] Potterat O. Goji (Lycium barbarum and L. chinense): Phytochemistry, pharmacology and safety in the perspective of traditional uses and recent popularity. Planta Med 2010;76:7-19.**

**[4] Zhang XJ, Yu HY, Cai YJ, Ke M. Lycium barbarum polysaccharides inhibit proliferation and migration of bladder cancer cell lines BIU87 by suppressing Pi3K/AKT pathway. Oncotarget 2017;8:5936-42.**

**[5] Tang L, Bao S, Du Y, Jiang Z, Wuliji AO, Ren X, et al. Antioxidant effects of Lycium barbarum polysaccharides on photoreceptor degeneration in the light-exposed mouse retina. Biomed Pharmacother 2018;103:829-37.**

**[6] Zhang W, Zhang J, Ding D, Zhang L, Muehlmann LA, Deng SE, et al. Synthesis and antioxidant properties of Lycium barbarum polysaccharides capped selenium nanoparticles using tea extract. Artif Cells Nanomed Biotechnol 2018;46:1463-70.**

**[7] Ma K, Wang X, Feng S, Xia X, Zhang H, Rahaman A, et al. From the perspective of Traditional Chinese Medicine: Treatment of mental disorders in COVID-19 survivors. Biomed Pharmacother 2020;132:110810.**

**[8] Po KK, Leung JW, Chan JN, Fung TK, Sánchez-Vidaña DI, Sin EL, et al. Protective effect of Lycium Barbarum polysaccharides on dextromethorphan-induced mood impairment and neurogenesis suppression. Brain Res Bull 2017;134:10-7.**

**[9] Fang S, Dong L, Liu L, Guo J, Zhao L, Zhang J, et al. HERB: a high-throughput experiment- and reference-guided database of traditional Chinese medicine. Nucleic Acids Res 2021;49:D1197-d206.**

**[10] Zhang S, He F, Chen X, Ding K. Isolation and structural characterization of a pectin from Lycium ruthenicum Murr and its anti-pancreatic ductal adenocarcinoma cell activity. Carbohydr Polym 2019;223:115104.**

**[11] Cao YL, Li YL, Fan YF, Li Z, Yoshida K, Wang JY, et al. Wolfberry genomes and the evolution of Lycium (Solanaceae). Commun Biol 2021;4:671.**

**[12] Chen J, Liu X, Zhu L, Wang Y. Nuclear genome size estimation and karyotype analysis of Lycium species (Solanaceae). Scientia Horticulturae 2013;151:46-50.**

**[13] Giri MK, Swain S, Gautam JK, Singh S, Singh N, Bhattacharjee L, et al. The Arabidopsis thaliana At4g13040 gene, a unique member of the AP2/EREBP family, is a positive regulator for salicylic acid accumulation and basal defense against bacterial pathogens. J Plant Physiol 2014;171:860-7.**

**[15] Schauser L, Roussis A, Stiller J, Stougaard J. A plant regulator controlling development of symbiotic root nodules. Nature 1999;402:191-5.**

**[16] Hou X, Zhou J, Liu C, Liu L, Shen L, Yu H. Nuclear factor Y-mediated H3K27me3 demethylation of the SOC1 locus orchestrates flowering responses of Arabidopsis. Nat Commun 2014;5:4601.**

**[17] Ru L, He Y, Zhu Z, Patrick JW, Ruan YL. Integrating Sugar Metabolism With Transport: Elevation of Endogenous Cell Wall Invertase Activity Up-Regulates SlHT2 and SlSWEET12c Expression for Early Fruit Development in Tomato. Front Genet 2020;11:592596.**

**[18] Breia R, Conde A, Badim H, Fortes AM, Gerós H, Granell A. Plant SWEETs: from sugar transport to plant-pathogen interaction and more unexpected physiological roles. Plant Physiol 2021;186:836-52.**

**[20] Eom JS, Chen LQ, Sosso D, Julius BT, Lin IW, Qu XQ, et al. SWEETs, transporters for intracellular and intercellular sugar translocation. Curr Opin Plant Biol 2015;25:53-62.**

**[21] Li Y, Feng S, Ma S, Sui X, Zhang Z. Spatiotemporal Expression and Substrate Specificity Analysis of the Cucumber SWEET Gene Family. Front Plant Sci 2017;8:1855.**

**[22] Patil G, Valliyodan B, Deshmukh R, Prince S, Nicander B, Zhao M, et al. Soybean (Glycine max) SWEET gene family: insights through comparative genomics, transcriptome profiling and whole genome re-sequence analysis. BMC Genomics 2015;16:520.**

**[23] Zhang W, Wang S, Yu F, Tang J, Shan X, Bao K, et al. Genome-wide characterization and expression profiling of SWEET genes in cabbage (Brassica oleracea var. capitata L.) reveal their roles in chilling and clubroot disease responses. BMC Genomics 2019;20:93.**

**[24] Feng CY, Han JX, Han XX, Jiang J. Genome-wide identification, phylogeny, and expression analysis of the SWEET gene family in tomato. Gene 2015;573:261-72.**

**[25] Manck-Götzenberger J, Requena N. Arbuscular mycorrhiza Symbiosis Induces a Major Transcriptional Reprogramming of the Potato SWEET Sugar Transporter Family. Front Plant Sci 2016;7:487.**

**[26] Ko HY, Ho LH, Neuhaus HE, Guo WJ. Transporter SlSWEET15 unloads sucrose from phloem and seed coat for fruit and seed development in tomato. Plant Physiol 2021;187:2230-45.**

**[27] Zhang X, Feng C, Wang M, Li T, Liu X, Jiang J. Plasma membrane-localized SlSWEET7a and SlSWEET14 regulate sugar transport and storage in tomato fruits. Hortic Res 2021;8:186.**

**[28] Pan L, Guo Q, Chai S, Cheng Y, Ruan M, Ye Q, et al. Evolutionary Conservation and Expression Patterns of Neutral/Alkaline Invertases in Solanum. Biomolecules 2019;9.**

**[29] Veillet F, Gaillard C, Coutos-Thévenot P, La Camera S. Targeting the AtCWIN1 Gene to Explore the Role of Invertases in Sucrose Transport in Roots and during Botrytis cinerea Infection. Front Plant Sci 2016;7:1899.**

**[30] Wan H, Wu L, Yang Y, Zhou G, Ruan YL. Evolution of Sucrose Metabolism: The Dichotomy of Invertases and Beyond. Trends Plant Sci 2018;23:163-77.**

**[31] Jin Y, Ni DA, Ruan YL. Posttranslational elevation of cell wall invertase activity by silencing its inhibitor in tomato delays leaf senescence and increases seed weight and fruit hexose level. Plant Cell 2009;21:2072-89.**

**[32] Shen S, Ma S, Liu Y, Liao S, Li J, Wu L, et al. Cell Wall Invertase and Sugar Transporters Are Differentially Activated in Tomato Styles and Ovaries During Pollination and Fertilization. Front Plant Sci 2019;10:506.**

**[33] Lombard V, Golaconda Ramulu H, Drula E, Coutinho PM, Henrissat B. The carbohydrate-active enzymes database (CAZy) in 2013. Nucleic Acids Res 2014;42:D490-5.**

**[34] Zhang H, Yohe T, Huang L, Entwistle S, Wu P, Yang Z, et al. dbCAN2: a meta server for automated carbohydrate-active enzyme annotation. Nucleic Acids Res 2018;46:W95-w101.**

**[35] Langfelder P, Horvath S. WGCNA: an R package for weighted correlation network analysis. BMC Bioinformatics 2008;9:559.**

**[36] Kaczmarska A, Pieczywek PM, Cybulska J, Zdunek A. Structure and functionality of Rhamnogalacturonan I in the cell wall and in solution: A review. Carbohydr Polym 2022;278:118909.**

**[37] Wachananawat B, Kuroha T, Takenaka Y, Kajiura H, Naramoto S, Yokoyama R, et al. Diversity of Pectin Rhamnogalacturonan I Rhamnosyltransferases in Glycosyltransferase Family 106. Front Plant Sci 2020;11:997.**

**[38] Atmodjo MA, Sakuragi Y, Zhu X, Burrell AJ, Mohanty SS, Atwood JA, 3rd, et al. Galacturonosyltransferase (GAUT)1 and GAUT7 are the core of a plant cell wall pectin biosynthetic homogalacturonan:galacturonosyltransferase complex. Proc Natl Acad Sci U S A 2011;108:20225-30.**

**[39] Ridley BL, O'Neill MA, Mohnen D. Pectins: structure, biosynthesis, and oligogalacturonide-related signaling. Phytochemistry 2001;57:929-67.**

**[40] Philippe F, Pelloux J, Rayon C. Plant pectin acetylesterase structure and function: new insights from bioinformatic analysis. BMC Genomics 2017;18:456.**

**[41] Carpita NC, Gibeaut DM. Structural models of primary cell walls in flowering plants: consistency of molecular structure with the physical properties of the walls during growth. Plant J 1993;3:1-30.**

**[42] Mohnen D. Pectin structure and biosynthesis. Curr Opin Plant Biol 2008;11:266-77.**

**[43] Harholt J, Jensen JK, Sørensen SO, Orfila C, Pauly M, Scheller HV. ARABINAN DEFICIENT 1 is a putative arabinosyltransferase involved in biosynthesis of pectic arabinan in Arabidopsis. Plant Physiol 2006;140:49-58.**

**[44] Redgwell RJ, Curti D, Wang J, Dobruchowska JM, Gerwig GJ, Kamerling JP, et al. Cell wall polysaccharides of Chinese Wolfberry (Lycium barbarum): Part 2. Characterisation of arabinogalactan-proteins. Carbohydrate Polymers 2011;84:1075-83.**

**[45] Statello L, Guo CJ, Chen LL, Huarte M. Gene regulation by long non-coding RNAs and its biological functions. Nat Rev Mol Cell Biol 2021;22:96-118.**

**[46] Zhao L, Wang J, Li Y, Song T, Wu Y, Fang S, et al. NONCODEV6: an updated database dedicated to long non-coding RNA annotation in both animals and plants. Nucleic Acids Res 2021;49:D165-d71.**

**[1] Gao Y, Wei Y, Wang Y, Gao F, Chen Z. Lycium Barbarum: A Traditional Chinese Herb and A Promising Anti-Aging Agent. Aging Dis 2017;8:778-91.**

**[2] Sun C, Chen X, Yang S, Jin C, Ding K, Chen C. LBP1C-2 from Lycium barbarum alleviated age-related bone loss by targeting BMPRIA/BMPRII/Noggin. Carbohydr Polym 2023;310:120725.**

**[3] Potterat O. Goji (Lycium barbarum and L. chinense): Phytochemistry, pharmacology and safety in the perspective of traditional uses and recent popularity. Planta Med 2010;76:7-19.**

**[4] Zhang XJ, Yu HY, Cai YJ, Ke M. Lycium barbarum polysaccharides inhibit proliferation and migration of bladder cancer cell lines BIU87 by suppressing Pi3K/AKT pathway. Oncotarget 2017;8:5936-42.**

**[5] Tang L, Bao S, Du Y, Jiang Z, Wuliji AO, Ren X, et al. Antioxidant effects of Lycium barbarum polysaccharides on photoreceptor degeneration in the light-exposed mouse retina. Biomed Pharmacother 2018;103:829-37.**

**[6] Zhang W, Zhang J, Ding D, Zhang L, Muehlmann LA, Deng SE, et al. Synthesis and antioxidant properties of Lycium barbarum polysaccharides capped selenium nanoparticles using tea extract. Artif Cells Nanomed Biotechnol 2018;46:1463-70.**

**[7] Ma K, Wang X, Feng S, Xia X, Zhang H, Rahaman A, et al. From the perspective of Traditional Chinese Medicine: Treatment of mental disorders in COVID-19 survivors. Biomed Pharmacother 2020;132:110810.**

**[8] Po KK, Leung JW, Chan JN, Fung TK, Sánchez-Vidaña DI, Sin EL, et al. Protective effect of Lycium Barbarum polysaccharides on dextromethorphan-induced mood impairment and neurogenesis suppression. Brain Res Bull 2017;134:10-7.**

**[9] Fang S, Dong L, Liu L, Guo J, Zhao L, Zhang J, et al. HERB: a high-throughput experiment- and reference-guided database of traditional Chinese medicine. Nucleic Acids Res 2021;49:D1197-d206.**

**[10] Zhang S, He F, Chen X, Ding K. Isolation and structural characterization of a pectin from Lycium ruthenicum Murr and its anti-pancreatic ductal adenocarcinoma cell activity. Carbohydr Polym 2019;223:115104.**

**[11] Cao YL, Li YL, Fan YF, Li Z, Yoshida K, Wang JY, et al. Wolfberry genomes and the evolution of Lycium (Solanaceae). Commun Biol 2021;4:671.**

**[12] Chen J, Liu X, Zhu L, Wang Y. Nuclear genome size estimation and karyotype analysis of Lycium species (Solanaceae). Scientia Horticulturae 2013;151:46-50.**

**[13] Giri MK, Swain S, Gautam JK, Singh S, Singh N, Bhattacharjee L, et al. The Arabidopsis thaliana At4g13040 gene, a unique member of the AP2/EREBP family, is a positive regulator for salicylic acid accumulation and basal defense against bacterial pathogens. J Plant Physiol 2014;171:860-7.**

**[14] Michaels SD, Ditta G, Gustafson-Brown C, Pelaz S, Yanofsky M, Amasino RM. AGL24 acts as a promoter of flowering in Arabidopsis and is positively regulated by vernalization. Plant J 2003;33:867-74.**

**[15] Schauser L, Roussis A, Stiller J, Stougaard J. A plant regulator controlling development of symbiotic root nodules. Nature 1999;402:191-5.**

**[16] Hou X, Zhou J, Liu C, Liu L, Shen L, Yu H. Nuclear factor Y-mediated H3K27me3 demethylation of the SOC1 locus orchestrates flowering responses of Arabidopsis. Nat Commun 2014;5:4601.**

**[17] Ru L, He Y, Zhu Z, Patrick JW, Ruan YL. Integrating Sugar Metabolism With Transport: Elevation of Endogenous Cell Wall Invertase Activity Up-Regulates SlHT2 and SlSWEET12c Expression for Early Fruit Development in Tomato. Front Genet 2020;11:592596.**

**[18] Breia R, Conde A, Badim H, Fortes AM, Gerós H, Granell A. Plant SWEETs: from sugar transport to plant-pathogen interaction and more unexpected physiological roles. Plant Physiol 2021;186:836-52.**

**[19] Chen LQ, Hou BH, Lalonde S, Takanaga H, Hartung ML, Qu XQ, et al. Sugar transporters for intercellular exchange and nutrition of pathogens. Nature 2010;468:527-32.**

**[20] Eom JS, Chen LQ, Sosso D, Julius BT, Lin IW, Qu XQ, et al. SWEETs, transporters for intracellular and intercellular sugar translocation. Curr Opin Plant Biol 2015;25:53-62.**

**[21] Li Y, Feng S, Ma S, Sui X, Zhang Z. Spatiotemporal Expression and Substrate Specificity Analysis of the Cucumber SWEET Gene Family. Front Plant Sci 2017;8:1855.**

**[22] Patil G, Valliyodan B, Deshmukh R, Prince S, Nicander B, Zhao M, et al. Soybean (Glycine max) SWEET gene family: insights through comparative genomics, transcriptome profiling and whole genome re-sequence analysis. BMC Genomics 2015;16:520.**

**[23] Zhang W, Wang S, Yu F, Tang J, Shan X, Bao K, et al. Genome-wide characterization and expression profiling of SWEET genes in cabbage (Brassica oleracea var. capitata L.) reveal their roles in chilling and clubroot disease responses. BMC Genomics 2019;20:93.**

**[24] Feng CY, Han JX, Han XX, Jiang J. Genome-wide identification, phylogeny, and expression analysis of the SWEET gene family in tomato. Gene 2015;573:261-72.**

**[25] Manck-Götzenberger J, Requena N. Arbuscular mycorrhiza Symbiosis Induces a Major Transcriptional Reprogramming of the Potato SWEET Sugar Transporter Family. Front Plant Sci 2016;7:487.**

**[26] Ko HY, Ho LH, Neuhaus HE, Guo WJ. Transporter SlSWEET15 unloads sucrose from phloem and seed coat for fruit and seed development in tomato. Plant Physiol 2021;187:2230-45.**

**[27] Zhang X, Feng C, Wang M, Li T, Liu X, Jiang J. Plasma membrane-localized SlSWEET7a and SlSWEET14 regulate sugar transport and storage in tomato fruits. Hortic Res 2021;8:186.**

**[28] Pan L, Guo Q, Chai S, Cheng Y, Ruan M, Ye Q, et al. Evolutionary Conservation and Expression Patterns of Neutral/Alkaline Invertases in Solanum. Biomolecules 2019;9.**

**[29] Veillet F, Gaillard C, Coutos-Thévenot P, La Camera S. Targeting the AtCWIN1 Gene to Explore the Role of Invertases in Sucrose Transport in Roots and during Botrytis cinerea Infection. Front Plant Sci 2016;7:1899.**

**[30] Wan H, Wu L, Yang Y, Zhou G, Ruan YL. Evolution of Sucrose Metabolism: The Dichotomy of Invertases and Beyond. Trends Plant Sci 2018;23:163-77.**

**[31] Jin Y, Ni DA, Ruan YL. Posttranslational elevation of cell wall invertase activity by silencing its inhibitor in tomato delays leaf senescence and increases seed weight and fruit hexose level. Plant Cell 2009;21:2072-89.**

**[32] Shen S, Ma S, Liu Y, Liao S, Li J, Wu L, et al. Cell Wall Invertase and Sugar Transporters Are Differentially Activated in Tomato Styles and Ovaries During Pollination and Fertilization. Front Plant Sci 2019;10:506.**

**[33] Lombard V, Golaconda Ramulu H, Drula E, Coutinho PM, Henrissat B. The carbohydrate-active enzymes database (CAZy) in 2013. Nucleic Acids Res 2014;42:D490-5.**

**[34] Zhang H, Yohe T, Huang L, Entwistle S, Wu P, Yang Z, et al. dbCAN2: a meta server for automated carbohydrate-active enzyme annotation. Nucleic Acids Res 2018;46:W95-w101.**

**[35] Langfelder P, Horvath S. WGCNA: an R package for weighted correlation network analysis. BMC Bioinformatics 2008;9:559.**

**[36] Kaczmarska A, Pieczywek PM, Cybulska J, Zdunek A. Structure and functionality of Rhamnogalacturonan I in the cell wall and in solution: A review. Carbohydr Polym 2022;278:118909.**

**[37] Wachananawat B, Kuroha T, Takenaka Y, Kajiura H, Naramoto S, Yokoyama R, et al. Diversity of Pectin Rhamnogalacturonan I Rhamnosyltransferases in Glycosyltransferase Family 106. Front Plant Sci 2020;11:997.**

**[38] Atmodjo MA, Sakuragi Y, Zhu X, Burrell AJ, Mohanty SS, Atwood JA, 3rd, et al. Galacturonosyltransferase (GAUT)1 and GAUT7 are the core of a plant cell wall pectin biosynthetic homogalacturonan:galacturonosyltransferase complex. Proc Natl Acad Sci U S A 2011;108:20225-30.**

**[39] Ridley BL, O'Neill MA, Mohnen D. Pectins: structure, biosynthesis, and oligogalacturonide-related signaling. Phytochemistry 2001;57:929-67.**

**[40] Philippe F, Pelloux J, Rayon C. Plant pectin acetylesterase structure and function: new insights from bioinformatic analysis. BMC Genomics 2017;18:456.**

**[41] Carpita NC, Gibeaut DM. Structural models of primary cell walls in flowering plants: consistency of molecular structure with the physical properties of the walls during growth. Plant J 1993;3:1-30.**

**[42] Mohnen D. Pectin structure and biosynthesis. Curr Opin Plant Biol 2008;11:266-77.**

**[43] Harholt J, Jensen JK, Sørensen SO, Orfila C, Pauly M, Scheller HV. ARABINAN DEFICIENT 1 is a putative arabinosyltransferase involved in biosynthesis of pectic arabinan in Arabidopsis. Plant Physiol 2006;140:49-58.**

**[44] Redgwell RJ, Curti D, Wang J, Dobruchowska JM, Gerwig GJ, Kamerling JP, et al. Cell wall polysaccharides of Chinese Wolfberry (Lycium barbarum): Part 2. Characterisation of arabinogalactan-proteins. Carbohydrate Polymers 2011;84:1075-83.**

**[45] Statello L, Guo CJ, Chen LL, Huarte M. Gene regulation by long non-coding RNAs and its biological functions. Nat Rev Mol Cell Biol 2021;22:96-118.**

**[46] Zhao L, Wang J, Li Y, Song T, Wu Y, Fang S, et al. NONCODEV6: an updated database dedicated to long non-coding RNA annotation in both animals and plants. Nucleic Acids Res 2021;49:D165-d71.**

**[47] Bailey TL, Johnson J, Grant CE, Noble WS. The MEME Suite. Nucleic Acids Res 2015;43:W39-49.**

**[1] Gao Y, Wei Y, Wang Y, Gao F, Chen Z. Lycium Barbarum: A Traditional Chinese Herb and A Promising Anti-Aging Agent. Aging Dis 2017;8:778-91.**

**[2] Sun C, Chen X, Yang S, Jin C, Ding K, Chen C. LBP1C-2 from Lycium barbarum alleviated age-related bone loss by targeting BMPRIA/BMPRII/Noggin. Carbohydr Polym 2023;310:120725.**

**[3] Potterat O. Goji (Lycium barbarum and L. chinense): Phytochemistry, pharmacology and safety in the perspective of traditional uses and recent popularity. Planta Med 2010;76:7-19.**

**[4] Zhang XJ, Yu HY, Cai YJ, Ke M. Lycium barbarum polysaccharides inhibit proliferation and migration of bladder cancer cell lines BIU87 by suppressing Pi3K/AKT pathway. Oncotarget 2017;8:5936-42.**

**[5] Tang L, Bao S, Du Y, Jiang Z, Wuliji AO, Ren X, et al. Antioxidant effects of Lycium barbarum polysaccharides on photoreceptor degeneration in the light-exposed mouse retina. Biomed Pharmacother 2018;103:829-37.**

**[6] Zhang W, Zhang J, Ding D, Zhang L, Muehlmann LA, Deng SE, et al. Synthesis and antioxidant properties of Lycium barbarum polysaccharides capped selenium nanoparticles using tea extract. Artif Cells Nanomed Biotechnol 2018;46:1463-70.**

**[7] Ma K, Wang X, Feng S, Xia X, Zhang H, Rahaman A, et al. From the perspective of Traditional Chinese Medicine: Treatment of mental disorders in COVID-19 survivors. Biomed Pharmacother 2020;132:110810.**

**[8] Po KK, Leung JW, Chan JN, Fung TK, Sánchez-Vidaña DI, Sin EL, et al. Protective effect of Lycium Barbarum polysaccharides on dextromethorphan-induced mood impairment and neurogenesis suppression. Brain Res Bull 2017;134:10-7.**

**[9] Fang S, Dong L, Liu L, Guo J, Zhao L, Zhang J, et al. HERB: a high-throughput experiment- and reference-guided database of traditional Chinese medicine. Nucleic Acids Res 2021;49:D1197-d206.**

**[10] Zhang S, He F, Chen X, Ding K. Isolation and structural characterization of a pectin from Lycium ruthenicum Murr and its anti-pancreatic ductal adenocarcinoma cell activity. Carbohydr Polym 2019;223:115104.**

**[11] Cao YL, Li YL, Fan YF, Li Z, Yoshida K, Wang JY, et al. Wolfberry genomes and the evolution of Lycium (Solanaceae). Commun Biol 2021;4:671.**

**[12] Chen J, Liu X, Zhu L, Wang Y. Nuclear genome size estimation and karyotype analysis of Lycium species (Solanaceae). Scientia Horticulturae 2013;151:46-50.**

**[13] Giri MK, Swain S, Gautam JK, Singh S, Singh N, Bhattacharjee L, et al. The Arabidopsis thaliana At4g13040 gene, a unique member of the AP2/EREBP family, is a positive regulator for salicylic acid accumulation and basal defense against bacterial pathogens. J Plant Physiol 2014;171:860-7.**

**[14] Michaels SD, Ditta G, Gustafson-Brown C, Pelaz S, Yanofsky M, Amasino RM. AGL24 acts as a promoter of flowering in Arabidopsis and is positively regulated by vernalization. Plant J 2003;33:867-74.**

**[15] Schauser L, Roussis A, Stiller J, Stougaard J. A plant regulator controlling development of symbiotic root nodules. Nature 1999;402:191-5.**

**[16] Hou X, Zhou J, Liu C, Liu L, Shen L, Yu H. Nuclear factor Y-mediated H3K27me3 demethylation of the SOC1 locus orchestrates flowering responses of Arabidopsis. Nat Commun 2014;5:4601.**

**[17] Ru L, He Y, Zhu Z, Patrick JW, Ruan YL. Integrating Sugar Metabolism With Transport: Elevation of Endogenous Cell Wall Invertase Activity Up-Regulates SlHT2 and SlSWEET12c Expression for Early Fruit Development in Tomato. Front Genet 2020;11:592596.**

**[18] Breia R, Conde A, Badim H, Fortes AM, Gerós H, Granell A. Plant SWEETs: from sugar transport to plant-pathogen interaction and more unexpected physiological roles. Plant Physiol 2021;186:836-52.**

**[19] Chen LQ, Hou BH, Lalonde S, Takanaga H, Hartung ML, Qu XQ, et al. Sugar transporters for intercellular exchange and nutrition of pathogens. Nature 2010;468:527-32.**

**[20] Eom JS, Chen LQ, Sosso D, Julius BT, Lin IW, Qu XQ, et al. SWEETs, transporters for intracellular and intercellular sugar translocation. Curr Opin Plant Biol 2015;25:53-62.**

**[21] Li Y, Feng S, Ma S, Sui X, Zhang Z. Spatiotemporal Expression and Substrate Specificity Analysis of the Cucumber SWEET Gene Family. Front Plant Sci 2017;8:1855.**

**[22] Patil G, Valliyodan B, Deshmukh R, Prince S, Nicander B, Zhao M, et al. Soybean (Glycine max) SWEET gene family: insights through comparative genomics, transcriptome profiling and whole genome re-sequence analysis. BMC Genomics 2015;16:520.**

**[23] Zhang W, Wang S, Yu F, Tang J, Shan X, Bao K, et al. Genome-wide characterization and expression profiling of SWEET genes in cabbage (Brassica oleracea var. capitata L.) reveal their roles in chilling and clubroot disease responses. BMC Genomics 2019;20:93.**

**[24] Feng CY, Han JX, Han XX, Jiang J. Genome-wide identification, phylogeny, and expression analysis of the SWEET gene family in tomato. Gene 2015;573:261-72.**

**[25] Manck-Götzenberger J, Requena N. Arbuscular mycorrhiza Symbiosis Induces a Major Transcriptional Reprogramming of the Potato SWEET Sugar Transporter Family. Front Plant Sci 2016;7:487.**

**[26] Ko HY, Ho LH, Neuhaus HE, Guo WJ. Transporter SlSWEET15 unloads sucrose from phloem and seed coat for fruit and seed development in tomato. Plant Physiol 2021;187:2230-45.**

**[27] Zhang X, Feng C, Wang M, Li T, Liu X, Jiang J. Plasma membrane-localized SlSWEET7a and SlSWEET14 regulate sugar transport and storage in tomato fruits. Hortic Res 2021;8:186.**

**[28] Pan L, Guo Q, Chai S, Cheng Y, Ruan M, Ye Q, et al. Evolutionary Conservation and Expression Patterns of Neutral/Alkaline Invertases in Solanum. Biomolecules 2019;9.**

**[30] Wan H, Wu L, Yang Y, Zhou G, Ruan YL. Evolution of Sucrose Metabolism: The Dichotomy of Invertases and Beyond. Trends Plant Sci 2018;23:163-77.**

**[31] Jin Y, Ni DA, Ruan YL. Posttranslational elevation of cell wall invertase activity by silencing its inhibitor in tomato delays leaf senescence and increases seed weight and fruit hexose level. Plant Cell 2009;21:2072-89.**

**[32] Shen S, Ma S, Liu Y, Liao S, Li J, Wu L, et al. Cell Wall Invertase and Sugar Transporters Are Differentially Activated in Tomato Styles and Ovaries During Pollination and Fertilization. Front Plant Sci 2019;10:506.**

**[33] Lombard V, Golaconda Ramulu H, Drula E, Coutinho PM, Henrissat B. The carbohydrate-active enzymes database (CAZy) in 2013. Nucleic Acids Res 2014;42:D490-5.**

**[34] Zhang H, Yohe T, Huang L, Entwistle S, Wu P, Yang Z, et al. dbCAN2: a meta server for automated carbohydrate-active enzyme annotation. Nucleic Acids Res 2018;46:W95-w101.**

**[35] Langfelder P, Horvath S. WGCNA: an R package for weighted correlation network analysis. BMC Bioinformatics 2008;9:559.**

**[36] Kaczmarska A, Pieczywek PM, Cybulska J, Zdunek A. Structure and functionality of Rhamnogalacturonan I in the cell wall and in solution: A review. Carbohydr Polym 2022;278:118909.**

**[37] Wachananawat B, Kuroha T, Takenaka Y, Kajiura H, Naramoto S, Yokoyama R, et al. Diversity of Pectin Rhamnogalacturonan I Rhamnosyltransferases in Glycosyltransferase Family 106. Front Plant Sci 2020;11:997.**

**[38] Atmodjo MA, Sakuragi Y, Zhu X, Burrell AJ, Mohanty SS, Atwood JA, 3rd, et al. Galacturonosyltransferase (GAUT)1 and GAUT7 are the core of a plant cell wall pectin biosynthetic homogalacturonan:galacturonosyltransferase complex. Proc Natl Acad Sci U S A 2011;108:20225-30.**

**[39] Ridley BL, O'Neill MA, Mohnen D. Pectins: structure, biosynthesis, and oligogalacturonide-related signaling. Phytochemistry 2001;57:929-67.**

**[40] Philippe F, Pelloux J, Rayon C. Plant pectin acetylesterase structure and function: new insights from bioinformatic analysis. BMC Genomics 2017;18:456.**

**[41] Carpita NC, Gibeaut DM. Structural models of primary cell walls in flowering plants: consistency of molecular structure with the physical properties of the walls during growth. Plant J 1993;3:1-30.**

**[42] Mohnen D. Pectin structure and biosynthesis. Curr Opin Plant Biol 2008;11:266-77.**

**[43] Harholt J, Jensen JK, Sørensen SO, Orfila C, Pauly M, Scheller HV. ARABINAN DEFICIENT 1 is a putative arabinosyltransferase involved in biosynthesis of pectic arabinan in Arabidopsis. Plant Physiol 2006;140:49-58.**

**[44] Redgwell RJ, Curti D, Wang J, Dobruchowska JM, Gerwig GJ, Kamerling JP, et al. Cell wall polysaccharides of Chinese Wolfberry (Lycium barbarum): Part 2. Characterisation of arabinogalactan-proteins. Carbohydrate Polymers 2011;84:1075-83.**

**[45] Statello L, Guo CJ, Chen LL, Huarte M. Gene regulation by long non-coding RNAs and its biological functions. Nat Rev Mol Cell Biol 2021;22:96-118.**

**[46] Zhao L, Wang J, Li Y, Song T, Wu Y, Fang S, et al. NONCODEV6: an updated database dedicated to long non-coding RNA annotation in both animals and plants. Nucleic Acids Res 2021;49:D165-d71.**

**[47] Bailey TL, Johnson J, Grant CE, Noble WS. The MEME Suite. Nucleic Acids Res 2015;43:W39-49.**

**[48] Yin Y, Chen H, Hahn MG, Mohnen D, Xu Y. Evolution and function of the plant cell wall synthesis-related glycosyltransferase family 8. Plant Physiol 2010;153:1729-46.**

**[1] Gao Y, Wei Y, Wang Y, Gao F, Chen Z. Lycium Barbarum: A Traditional Chinese Herb and A Promising Anti-Aging Agent. Aging Dis 2017;8:778-91.**

**[2] Sun C, Chen X, Yang S, Jin C, Ding K, Chen C. LBP1C-2 from Lycium barbarum alleviated age-related bone loss by targeting BMPRIA/BMPRII/Noggin. Carbohydr Polym 2023;310:120725.**

**[3] Potterat O. Goji (Lycium barbarum and L. chinense): Phytochemistry, pharmacology and safety in the perspective of traditional uses and recent popularity. Planta Med 2010;76:7-19.**

**[4] Zhang XJ, Yu HY, Cai YJ, Ke M. Lycium barbarum polysaccharides inhibit proliferation and migration of bladder cancer cell lines BIU87 by suppressing Pi3K/AKT pathway. Oncotarget 2017;8:5936-42.**

**[5] Tang L, Bao S, Du Y, Jiang Z, Wuliji AO, Ren X, et al. Antioxidant effects of Lycium barbarum polysaccharides on photoreceptor degeneration in the light-exposed mouse retina. Biomed Pharmacother 2018;103:829-37.**

**[6] Zhang W, Zhang J, Ding D, Zhang L, Muehlmann LA, Deng SE, et al. Synthesis and antioxidant properties of Lycium barbarum polysaccharides capped selenium nanoparticles using tea extract. Artif Cells Nanomed Biotechnol 2018;46:1463-70.**

**[7] Ma K, Wang X, Feng S, Xia X, Zhang H, Rahaman A, et al. From the perspective of Traditional Chinese Medicine: Treatment of mental disorders in COVID-19 survivors. Biomed Pharmacother 2020;132:110810.**

**[8] Po KK, Leung JW, Chan JN, Fung TK, Sánchez-Vidaña DI, Sin EL, et al. Protective effect of Lycium Barbarum polysaccharides on dextromethorphan-induced mood impairment and neurogenesis suppression. Brain Res Bull 2017;134:10-7.**

**[9] Fang S, Dong L, Liu L, Guo J, Zhao L, Zhang J, et al. HERB: a high-throughput experiment- and reference-guided database of traditional Chinese medicine. Nucleic Acids Res 2021;49:D1197-d206.**

**[10] Zhang S, He F, Chen X, Ding K. Isolation and structural characterization of a pectin from Lycium ruthenicum Murr and its anti-pancreatic ductal adenocarcinoma cell activity. Carbohydr Polym 2019;223:115104.**

**[11] Cao YL, Li YL, Fan YF, Li Z, Yoshida K, Wang JY, et al. Wolfberry genomes and the evolution of Lycium (Solanaceae). Commun Biol 2021;4:671.**

**[12] Chen J, Liu X, Zhu L, Wang Y. Nuclear genome size estimation and karyotype analysis of Lycium species (Solanaceae). Scientia Horticulturae 2013;151:46-50.**

**[13] Giri MK, Swain S, Gautam JK, Singh S, Singh N, Bhattacharjee L, et al. The Arabidopsis thaliana At4g13040 gene, a unique member of the AP2/EREBP family, is a positive regulator for salicylic acid accumulation and basal defense against bacterial pathogens. J Plant Physiol 2014;171:860-7.**

**[15] Schauser L, Roussis A, Stiller J, Stougaard J. A plant regulator controlling development of symbiotic root nodules. Nature 1999;402:191-5.**

**[16] Hou X, Zhou J, Liu C, Liu L, Shen L, Yu H. Nuclear factor Y-mediated H3K27me3 demethylation of the SOC1 locus orchestrates flowering responses of Arabidopsis. Nat Commun 2014;5:4601.**

**[17] Ru L, He Y, Zhu Z, Patrick JW, Ruan YL. Integrating Sugar Metabolism With Transport: Elevation of Endogenous Cell Wall Invertase Activity Up-Regulates SlHT2 and SlSWEET12c Expression for Early Fruit Development in Tomato. Front Genet 2020;11:592596.**

**[18] Breia R, Conde A, Badim H, Fortes AM, Gerós H, Granell A. Plant SWEETs: from sugar transport to plant-pathogen interaction and more unexpected physiological roles. Plant Physiol 2021;186:836-52.**

**[19] Chen LQ, Hou BH, Lalonde S, Takanaga H, Hartung ML, Qu XQ, et al. Sugar transporters for intercellular exchange and nutrition of pathogens. Nature 2010;468:527-32.**

**[20] Eom JS, Chen LQ, Sosso D, Julius BT, Lin IW, Qu XQ, et al. SWEETs, transporters for intracellular and intercellular sugar translocation. Curr Opin Plant Biol 2015;25:53-62.**

**[21] Li Y, Feng S, Ma S, Sui X, Zhang Z. Spatiotemporal Expression and Substrate Specificity Analysis of the Cucumber SWEET Gene Family. Front Plant Sci 2017;8:1855.**

**[22] Patil G, Valliyodan B, Deshmukh R, Prince S, Nicander B, Zhao M, et al. Soybean (Glycine max) SWEET gene family: insights through comparative genomics, transcriptome profiling and whole genome re-sequence analysis. BMC Genomics 2015;16:520.**

**[23] Zhang W, Wang S, Yu F, Tang J, Shan X, Bao K, et al. Genome-wide characterization and expression profiling of SWEET genes in cabbage (Brassica oleracea var. capitata L.) reveal their roles in chilling and clubroot disease responses. BMC Genomics 2019;20:93.**

**[24] Feng CY, Han JX, Han XX, Jiang J. Genome-wide identification, phylogeny, and expression analysis of the SWEET gene family in tomato. Gene 2015;573:261-72.**

**[25] Manck-Götzenberger J, Requena N. Arbuscular mycorrhiza Symbiosis Induces a Major Transcriptional Reprogramming of the Potato SWEET Sugar Transporter Family. Front Plant Sci 2016;7:487.**

**[26] Ko HY, Ho LH, Neuhaus HE, Guo WJ. Transporter SlSWEET15 unloads sucrose from phloem and seed coat for fruit and seed development in tomato. Plant Physiol 2021;187:2230-45.**

**[27] Zhang X, Feng C, Wang M, Li T, Liu X, Jiang J. Plasma membrane-localized SlSWEET7a and SlSWEET14 regulate sugar transport and storage in tomato fruits. Hortic Res 2021;8:186.**

**[28] Pan L, Guo Q, Chai S, Cheng Y, Ruan M, Ye Q, et al. Evolutionary Conservation and Expression Patterns of Neutral/Alkaline Invertases in Solanum. Biomolecules 2019;9.**

**[29] Veillet F, Gaillard C, Coutos-Thévenot P, La Camera S. Targeting the AtCWIN1 Gene to Explore the Role of Invertases in Sucrose Transport in Roots and during Botrytis cinerea Infection. Front Plant Sci 2016;7:1899.**

**[30] Wan H, Wu L, Yang Y, Zhou G, Ruan YL. Evolution of Sucrose Metabolism: The Dichotomy of Invertases and Beyond. Trends Plant Sci 2018;23:163-77.**

**[31] Jin Y, Ni DA, Ruan YL. Posttranslational elevation of cell wall invertase activity by silencing its inhibitor in tomato delays leaf senescence and increases seed weight and fruit hexose level. Plant Cell 2009;21:2072-89.**

**[32] Shen S, Ma S, Liu Y, Liao S, Li J, Wu L, et al. Cell Wall Invertase and Sugar Transporters Are Differentially Activated in Tomato Styles and Ovaries During Pollination and Fertilization. Front Plant Sci 2019;10:506.**

**[33] Lombard V, Golaconda Ramulu H, Drula E, Coutinho PM, Henrissat B. The carbohydrate-active enzymes database (CAZy) in 2013. Nucleic Acids Res 2014;42:D490-5.**

**[34] Zhang H, Yohe T, Huang L, Entwistle S, Wu P, Yang Z, et al. dbCAN2: a meta server for automated carbohydrate-active enzyme annotation. Nucleic Acids Res 2018;46:W95-w101.**

**[35] Langfelder P, Horvath S. WGCNA: an R package for weighted correlation network analysis. BMC Bioinformatics 2008;9:559.**

**[36] Kaczmarska A, Pieczywek PM, Cybulska J, Zdunek A. Structure and functionality of Rhamnogalacturonan I in the cell wall and in solution: A review. Carbohydr Polym 2022;278:118909.**

**[37] Wachananawat B, Kuroha T, Takenaka Y, Kajiura H, Naramoto S, Yokoyama R, et al. Diversity of Pectin Rhamnogalacturonan I Rhamnosyltransferases in Glycosyltransferase Family 106. Front Plant Sci 2020;11:997.**

**[38] Atmodjo MA, Sakuragi Y, Zhu X, Burrell AJ, Mohanty SS, Atwood JA, 3rd, et al. Galacturonosyltransferase (GAUT)1 and GAUT7 are the core of a plant cell wall pectin biosynthetic homogalacturonan:galacturonosyltransferase complex. Proc Natl Acad Sci U S A 2011;108:20225-30.**

**[39] Ridley BL, O'Neill MA, Mohnen D. Pectins: structure, biosynthesis, and oligogalacturonide-related signaling. Phytochemistry 2001;57:929-67.**

**[40] Philippe F, Pelloux J, Rayon C. Plant pectin acetylesterase structure and function: new insights from bioinformatic analysis. BMC Genomics 2017;18:456.**

**[41] Carpita NC, Gibeaut DM. Structural models of primary cell walls in flowering plants: consistency of molecular structure with the physical properties of the walls during growth. Plant J 1993;3:1-30.**

**[42] Mohnen D. Pectin structure and biosynthesis. Curr Opin Plant Biol 2008;11:266-77.**

**[43] Harholt J, Jensen JK, Sørensen SO, Orfila C, Pauly M, Scheller HV. ARABINAN DEFICIENT 1 is a putative arabinosyltransferase involved in biosynthesis of pectic arabinan in Arabidopsis. Plant Physiol 2006;140:49-58.**

**[44] Redgwell RJ, Curti D, Wang J, Dobruchowska JM, Gerwig GJ, Kamerling JP, et al. Cell wall polysaccharides of Chinese Wolfberry (Lycium barbarum): Part 2. Characterisation of arabinogalactan-proteins. Carbohydrate Polymers 2011;84:1075-83.**

**[45] Statello L, Guo CJ, Chen LL, Huarte M. Gene regulation by long non-coding RNAs and its biological functions. Nat Rev Mol Cell Biol 2021;22:96-118.**

**[46] Zhao L, Wang J, Li Y, Song T, Wu Y, Fang S, et al. NONCODEV6: an updated database dedicated to long non-coding RNA annotation in both animals and plants. Nucleic Acids Res 2021;49:D165-d71.**

**[47] Bailey TL, Johnson J, Grant CE, Noble WS. The MEME Suite. Nucleic Acids Res 2015;43:W39-49.**

**[48] Yin Y, Chen H, Hahn MG, Mohnen D, Xu Y. Evolution and function of the plant cell wall synthesis-related glycosyltransferase family 8. Plant Physiol 2010;153:1729-46.**

**[49] Caffall KH, Pattathil S, Phillips SE, Hahn MG, Mohnen D. Arabidopsis thaliana T-DNA mutants implicate GAUT genes in the biosynthesis of pectin and xylan in cell walls and seed testa. Mol Plant 2009;2:1000-14.**

**[1] Gao Y, Wei Y, Wang Y, Gao F, Chen Z. Lycium Barbarum: A Traditional Chinese Herb and A Promising Anti-Aging Agent. Aging Dis 2017;8:778-91.**

**[2] Sun C, Chen X, Yang S, Jin C, Ding K, Chen C. LBP1C-2 from Lycium barbarum alleviated age-related bone loss by targeting BMPRIA/BMPRII/Noggin. Carbohydr Polym 2023;310:120725.**

**[3] Potterat O. Goji (Lycium barbarum and L. chinense): Phytochemistry, pharmacology and safety in the perspective of traditional uses and recent popularity. Planta Med 2010;76:7-19.**

**[4] Zhang XJ, Yu HY, Cai YJ, Ke M. Lycium barbarum polysaccharides inhibit proliferation and migration of bladder cancer cell lines BIU87 by suppressing Pi3K/AKT pathway. Oncotarget 2017;8:5936-42.**

**[5] Tang L, Bao S, Du Y, Jiang Z, Wuliji AO, Ren X, et al. Antioxidant effects of Lycium barbarum polysaccharides on photoreceptor degeneration in the light-exposed mouse retina. Biomed Pharmacother 2018;103:829-37.**

**[6] Zhang W, Zhang J, Ding D, Zhang L, Muehlmann LA, Deng SE, et al. Synthesis and antioxidant properties of Lycium barbarum polysaccharides capped selenium nanoparticles using tea extract. Artif Cells Nanomed Biotechnol 2018;46:1463-70.**

**[7] Ma K, Wang X, Feng S, Xia X, Zhang H, Rahaman A, et al. From the perspective of Traditional Chinese Medicine: Treatment of mental disorders in COVID-19 survivors. Biomed Pharmacother 2020;132:110810.**

**[8] Po KK, Leung JW, Chan JN, Fung TK, Sánchez-Vidaña DI, Sin EL, et al. Protective effect of Lycium Barbarum polysaccharides on dextromethorphan-induced mood impairment and neurogenesis suppression. Brain Res Bull 2017;134:10-7.**

**[9] Fang S, Dong L, Liu L, Guo J, Zhao L, Zhang J, et al. HERB: a high-throughput experiment- and reference-guided database of traditional Chinese medicine. Nucleic Acids Res 2021;49:D1197-d206.**

**[10] Zhang S, He F, Chen X, Ding K. Isolation and structural characterization of a pectin from Lycium ruthenicum Murr and its anti-pancreatic ductal adenocarcinoma cell activity. Carbohydr Polym 2019;223:115104.**

**[11] Cao YL, Li YL, Fan YF, Li Z, Yoshida K, Wang JY, et al. Wolfberry genomes and the evolution of Lycium (Solanaceae). Commun Biol 2021;4:671.**

**[12] Chen J, Liu X, Zhu L, Wang Y. Nuclear genome size estimation and karyotype analysis of Lycium species (Solanaceae). Scientia Horticulturae 2013;151:46-50.**

**[13] Giri MK, Swain S, Gautam JK, Singh S, Singh N, Bhattacharjee L, et al. The Arabidopsis thaliana At4g13040 gene, a unique member of the AP2/EREBP family, is a positive regulator for salicylic acid accumulation and basal defense against bacterial pathogens. J Plant Physiol 2014;171:860-7.**

**[14] Michaels SD, Ditta G, Gustafson-Brown C, Pelaz S, Yanofsky M, Amasino RM. AGL24 acts as a promoter of flowering in Arabidopsis and is positively regulated by vernalization. Plant J 2003;33:867-74.**

**[15] Schauser L, Roussis A, Stiller J, Stougaard J. A plant regulator controlling development of symbiotic root nodules. Nature 1999;402:191-5.**

**[16] Hou X, Zhou J, Liu C, Liu L, Shen L, Yu H. Nuclear factor Y-mediated H3K27me3 demethylation of the SOC1 locus orchestrates flowering responses of Arabidopsis. Nat Commun 2014;5:4601.**

**[17] Ru L, He Y, Zhu Z, Patrick JW, Ruan YL. Integrating Sugar Metabolism With Transport: Elevation of Endogenous Cell Wall Invertase Activity Up-Regulates SlHT2 and SlSWEET12c Expression for Early Fruit Development in Tomato. Front Genet 2020;11:592596.**

**[18] Breia R, Conde A, Badim H, Fortes AM, Gerós H, Granell A. Plant SWEETs: from sugar transport to plant-pathogen interaction and more unexpected physiological roles. Plant Physiol 2021;186:836-52.**

**[19] Chen LQ, Hou BH, Lalonde S, Takanaga H, Hartung ML, Qu XQ, et al. Sugar transporters for intercellular exchange and nutrition of pathogens. Nature 2010;468:527-32.**

**[20] Eom JS, Chen LQ, Sosso D, Julius BT, Lin IW, Qu XQ, et al. SWEETs, transporters for intracellular and intercellular sugar translocation. Curr Opin Plant Biol 2015;25:53-62.**

**[21] Li Y, Feng S, Ma S, Sui X, Zhang Z. Spatiotemporal Expression and Substrate Specificity Analysis of the Cucumber SWEET Gene Family. Front Plant Sci 2017;8:1855.**

**[22] Patil G, Valliyodan B, Deshmukh R, Prince S, Nicander B, Zhao M, et al. Soybean (Glycine max) SWEET gene family: insights through comparative genomics, transcriptome profiling and whole genome re-sequence analysis. BMC Genomics 2015;16:520.**

**[23] Zhang W, Wang S, Yu F, Tang J, Shan X, Bao K, et al. Genome-wide characterization and expression profiling of SWEET genes in cabbage (Brassica oleracea var. capitata L.) reveal their roles in chilling and clubroot disease responses. BMC Genomics 2019;20:93.**

**[24] Feng CY, Han JX, Han XX, Jiang J. Genome-wide identification, phylogeny, and expression analysis of the SWEET gene family in tomato. Gene 2015;573:261-72.**

**[25] Manck-Götzenberger J, Requena N. Arbuscular mycorrhiza Symbiosis Induces a Major Transcriptional Reprogramming of the Potato SWEET Sugar Transporter Family. Front Plant Sci 2016;7:487.**

**[26] Ko HY, Ho LH, Neuhaus HE, Guo WJ. Transporter SlSWEET15 unloads sucrose from phloem and seed coat for fruit and seed development in tomato. Plant Physiol 2021;187:2230-45.**

**[27] Zhang X, Feng C, Wang M, Li T, Liu X, Jiang J. Plasma membrane-localized SlSWEET7a and SlSWEET14 regulate sugar transport and storage in tomato fruits. Hortic Res 2021;8:186.**

**[28] Pan L, Guo Q, Chai S, Cheng Y, Ruan M, Ye Q, et al. Evolutionary Conservation and Expression Patterns of Neutral/Alkaline Invertases in Solanum. Biomolecules 2019;9.**

**[29] Veillet F, Gaillard C, Coutos-Thévenot P, La Camera S. Targeting the AtCWIN1 Gene to Explore the Role of Invertases in Sucrose Transport in Roots and during Botrytis cinerea Infection. Front Plant Sci 2016;7:1899.**

**[30] Wan H, Wu L, Yang Y, Zhou G, Ruan YL. Evolution of Sucrose Metabolism: The Dichotomy of Invertases and Beyond. Trends Plant Sci 2018;23:163-77.**

**[31] Jin Y, Ni DA, Ruan YL. Posttranslational elevation of cell wall invertase activity by silencing its inhibitor in tomato delays leaf senescence and increases seed weight and fruit hexose level. Plant Cell 2009;21:2072-89.**

**[32] Shen S, Ma S, Liu Y, Liao S, Li J, Wu L, et al. Cell Wall Invertase and Sugar Transporters Are Differentially Activated in Tomato Styles and Ovaries During Pollination and Fertilization. Front Plant Sci 2019;10:506.**

**[33] Lombard V, Golaconda Ramulu H, Drula E, Coutinho PM, Henrissat B. The carbohydrate-active enzymes database (CAZy) in 2013. Nucleic Acids Res 2014;42:D490-5.**

**[34] Zhang H, Yohe T, Huang L, Entwistle S, Wu P, Yang Z, et al. dbCAN2: a meta server for automated carbohydrate-active enzyme annotation. Nucleic Acids Res 2018;46:W95-w101.**

**[35] Langfelder P, Horvath S. WGCNA: an R package for weighted correlation network analysis. BMC Bioinformatics 2008;9:559.**

**[36] Kaczmarska A, Pieczywek PM, Cybulska J, Zdunek A. Structure and functionality of Rhamnogalacturonan I in the cell wall and in solution: A review. Carbohydr Polym 2022;278:118909.**

**[37] Wachananawat B, Kuroha T, Takenaka Y, Kajiura H, Naramoto S, Yokoyama R, et al. Diversity of Pectin Rhamnogalacturonan I Rhamnosyltransferases in Glycosyltransferase Family 106. Front Plant Sci 2020;11:997.**

**[38] Atmodjo MA, Sakuragi Y, Zhu X, Burrell AJ, Mohanty SS, Atwood JA, 3rd, et al. Galacturonosyltransferase (GAUT)1 and GAUT7 are the core of a plant cell wall pectin biosynthetic homogalacturonan:galacturonosyltransferase complex. Proc Natl Acad Sci U S A 2011;108:20225-30.**

**[39] Ridley BL, O'Neill MA, Mohnen D. Pectins: structure, biosynthesis, and oligogalacturonide-related signaling. Phytochemistry 2001;57:929-67.**

**[40] Philippe F, Pelloux J, Rayon C. Plant pectin acetylesterase structure and function: new insights from bioinformatic analysis. BMC Genomics 2017;18:456.**

**[41] Carpita NC, Gibeaut DM. Structural models of primary cell walls in flowering plants: consistency of molecular structure with the physical properties of the walls during growth. Plant J 1993;3:1-30.**

**[42] Mohnen D. Pectin structure and biosynthesis. Curr Opin Plant Biol 2008;11:266-77.**

**[43] Harholt J, Jensen JK, Sørensen SO, Orfila C, Pauly M, Scheller HV. ARABINAN DEFICIENT 1 is a putative arabinosyltransferase involved in biosynthesis of pectic arabinan in Arabidopsis. Plant Physiol 2006;140:49-58.**

**[44] Redgwell RJ, Curti D, Wang J, Dobruchowska JM, Gerwig GJ, Kamerling JP, et al. Cell wall polysaccharides of Chinese Wolfberry (Lycium barbarum): Part 2. Characterisation of arabinogalactan-proteins. Carbohydrate Polymers 2011;84:1075-83.**

**[45] Statello L, Guo CJ, Chen LL, Huarte M. Gene regulation by long non-coding RNAs and its biological functions. Nat Rev Mol Cell Biol 2021;22:96-118.**

**[46] Zhao L, Wang J, Li Y, Song T, Wu Y, Fang S, et al. NONCODEV6: an updated database dedicated to long non-coding RNA annotation in both animals and plants. Nucleic Acids Res 2021;49:D165-d71.**

**[47] Bailey TL, Johnson J, Grant CE, Noble WS. The MEME Suite. Nucleic Acids Res 2015;43:W39-49.**

**[48] Yin Y, Chen H, Hahn MG, Mohnen D, Xu Y. Evolution and function of the plant cell wall synthesis-related glycosyltransferase family 8. Plant Physiol 2010;153:1729-46.**

**[49] Caffall KH, Pattathil S, Phillips SE, Hahn MG, Mohnen D. Arabidopsis thaliana T-DNA mutants implicate GAUT genes in the biosynthesis of pectin and xylan in cell walls and seed testa. Mol Plant 2009;2:1000-14.**

**[50] Wang L, Wang W, Wang YQ, Liu YY, Wang JX, Zhang XQ, et al. Arabidopsis galacturonosyltransferase (GAUT) 13 and GAUT14 have redundant functions in pollen tube growth. Mol Plant 2013;6:1131-48.**

**[1] Gao Y, Wei Y, Wang Y, Gao F, Chen Z. Lycium Barbarum: A Traditional Chinese Herb and A Promising Anti-Aging Agent. Aging Dis 2017;8:778-91.**

**[2] Sun C, Chen X, Yang S, Jin C, Ding K, Chen C. LBP1C-2 from Lycium barbarum alleviated age-related bone loss by targeting BMPRIA/BMPRII/Noggin. Carbohydr Polym 2023;310:120725.**

**[3] Potterat O. Goji (Lycium barbarum and L. chinense): Phytochemistry, pharmacology and safety in the perspective of traditional uses and recent popularity. Planta Med 2010;76:7-19.**

**[4] Zhang XJ, Yu HY, Cai YJ, Ke M. Lycium barbarum polysaccharides inhibit proliferation and migration of bladder cancer cell lines BIU87 by suppressing Pi3K/AKT pathway. Oncotarget 2017;8:5936-42.**

**[5] Tang L, Bao S, Du Y, Jiang Z, Wuliji AO, Ren X, et al. Antioxidant effects of Lycium barbarum polysaccharides on photoreceptor degeneration in the light-exposed mouse retina. Biomed Pharmacother 2018;103:829-37.**

**[6] Zhang W, Zhang J, Ding D, Zhang L, Muehlmann LA, Deng SE, et al. Synthesis and antioxidant properties of Lycium barbarum polysaccharides capped selenium nanoparticles using tea extract. Artif Cells Nanomed Biotechnol 2018;46:1463-70.**

**[7] Ma K, Wang X, Feng S, Xia X, Zhang H, Rahaman A, et al. From the perspective of Traditional Chinese Medicine: Treatment of mental disorders in COVID-19 survivors. Biomed Pharmacother 2020;132:110810.**

**[8] Po KK, Leung JW, Chan JN, Fung TK, Sánchez-Vidaña DI, Sin EL, et al. Protective effect of Lycium Barbarum polysaccharides on dextromethorphan-induced mood impairment and neurogenesis suppression. Brain Res Bull 2017;134:10-7.**

**[9] Fang S, Dong L, Liu L, Guo J, Zhao L, Zhang J, et al. HERB: a high-throughput experiment- and reference-guided database of traditional Chinese medicine. Nucleic Acids Res 2021;49:D1197-d206.**

**[10] Zhang S, He F, Chen X, Ding K. Isolation and structural characterization of a pectin from Lycium ruthenicum Murr and its anti-pancreatic ductal adenocarcinoma cell activity. Carbohydr Polym 2019;223:115104.**

**[11] Cao YL, Li YL, Fan YF, Li Z, Yoshida K, Wang JY, et al. Wolfberry genomes and the evolution of Lycium (Solanaceae). Commun Biol 2021;4:671.**

**[12] Chen J, Liu X, Zhu L, Wang Y. Nuclear genome size estimation and karyotype analysis of Lycium species (Solanaceae). Scientia Horticulturae 2013;151:46-50.**

**[14] Michaels SD, Ditta G, Gustafson-Brown C, Pelaz S, Yanofsky M, Amasino RM. AGL24 acts as a promoter of flowering in Arabidopsis and is positively regulated by vernalization. Plant J 2003;33:867-74.**

**[15] Schauser L, Roussis A, Stiller J, Stougaard J. A plant regulator controlling development of symbiotic root nodules. Nature 1999;402:191-5.**

**[16] Hou X, Zhou J, Liu C, Liu L, Shen L, Yu H. Nuclear factor Y-mediated H3K27me3 demethylation of the SOC1 locus orchestrates flowering responses of Arabidopsis. Nat Commun 2014;5:4601.**

**[17] Ru L, He Y, Zhu Z, Patrick JW, Ruan YL. Integrating Sugar Metabolism With Transport: Elevation of Endogenous Cell Wall Invertase Activity Up-Regulates SlHT2 and SlSWEET12c Expression for Early Fruit Development in Tomato. Front Genet 2020;11:592596.**

**[18] Breia R, Conde A, Badim H, Fortes AM, Gerós H, Granell A. Plant SWEETs: from sugar transport to plant-pathogen interaction and more unexpected physiological roles. Plant Physiol 2021;186:836-52.**

**[19] Chen LQ, Hou BH, Lalonde S, Takanaga H, Hartung ML, Qu XQ, et al. Sugar transporters for intercellular exchange and nutrition of pathogens. Nature 2010;468:527-32.**

**[20] Eom JS, Chen LQ, Sosso D, Julius BT, Lin IW, Qu XQ, et al. SWEETs, transporters for intracellular and intercellular sugar translocation. Curr Opin Plant Biol 2015;25:53-62.**

**[21] Li Y, Feng S, Ma S, Sui X, Zhang Z. Spatiotemporal Expression and Substrate Specificity Analysis of the Cucumber SWEET Gene Family. Front Plant Sci 2017;8:1855.**

**[22] Patil G, Valliyodan B, Deshmukh R, Prince S, Nicander B, Zhao M, et al. Soybean (Glycine max) SWEET gene family: insights through comparative genomics, transcriptome profiling and whole genome re-sequence analysis. BMC Genomics 2015;16:520.**

**[23] Zhang W, Wang S, Yu F, Tang J, Shan X, Bao K, et al. Genome-wide characterization and expression profiling of SWEET genes in cabbage (Brassica oleracea var. capitata L.) reveal their roles in chilling and clubroot disease responses. BMC Genomics 2019;20:93.**

**[24] Feng CY, Han JX, Han XX, Jiang J. Genome-wide identification, phylogeny, and expression analysis of the SWEET gene family in tomato. Gene 2015;573:261-72.**

**[25] Manck-Götzenberger J, Requena N. Arbuscular mycorrhiza Symbiosis Induces a Major Transcriptional Reprogramming of the Potato SWEET Sugar Transporter Family. Front Plant Sci 2016;7:487.**

**[26] Ko HY, Ho LH, Neuhaus HE, Guo WJ. Transporter SlSWEET15 unloads sucrose from phloem and seed coat for fruit and seed development in tomato. Plant Physiol 2021;187:2230-45.**

**[27] Zhang X, Feng C, Wang M, Li T, Liu X, Jiang J. Plasma membrane-localized SlSWEET7a and SlSWEET14 regulate sugar transport and storage in tomato fruits. Hortic Res 2021;8:186.**

**[28] Pan L, Guo Q, Chai S, Cheng Y, Ruan M, Ye Q, et al. Evolutionary Conservation and Expression Patterns of Neutral/Alkaline Invertases in Solanum. Biomolecules 2019;9.**

**[29] Veillet F, Gaillard C, Coutos-Thévenot P, La Camera S. Targeting the AtCWIN1 Gene to Explore the Role of Invertases in Sucrose Transport in Roots and during Botrytis cinerea Infection. Front Plant Sci 2016;7:1899.**

**[30] Wan H, Wu L, Yang Y, Zhou G, Ruan YL. Evolution of Sucrose Metabolism: The Dichotomy of Invertases and Beyond. Trends Plant Sci 2018;23:163-77.**

**[31] Jin Y, Ni DA, Ruan YL. Posttranslational elevation of cell wall invertase activity by silencing its inhibitor in tomato delays leaf senescence and increases seed weight and fruit hexose level. Plant Cell 2009;21:2072-89.**

**[32] Shen S, Ma S, Liu Y, Liao S, Li J, Wu L, et al. Cell Wall Invertase and Sugar Transporters Are Differentially Activated in Tomato Styles and Ovaries During Pollination and Fertilization. Front Plant Sci 2019;10:506.**

**[33] Lombard V, Golaconda Ramulu H, Drula E, Coutinho PM, Henrissat B. The carbohydrate-active enzymes database (CAZy) in 2013. Nucleic Acids Res 2014;42:D490-5.**

**[34] Zhang H, Yohe T, Huang L, Entwistle S, Wu P, Yang Z, et al. dbCAN2: a meta server for automated carbohydrate-active enzyme annotation. Nucleic Acids Res 2018;46:W95-w101.**

**[35] Langfelder P, Horvath S. WGCNA: an R package for weighted correlation network analysis. BMC Bioinformatics 2008;9:559.**

**[36] Kaczmarska A, Pieczywek PM, Cybulska J, Zdunek A. Structure and functionality of Rhamnogalacturonan I in the cell wall and in solution: A review. Carbohydr Polym 2022;278:118909.**

**[37] Wachananawat B, Kuroha T, Takenaka Y, Kajiura H, Naramoto S, Yokoyama R, et al. Diversity of Pectin Rhamnogalacturonan I Rhamnosyltransferases in Glycosyltransferase Family 106. Front Plant Sci 2020;11:997.**

**[38] Atmodjo MA, Sakuragi Y, Zhu X, Burrell AJ, Mohanty SS, Atwood JA, 3rd, et al. Galacturonosyltransferase (GAUT)1 and GAUT7 are the core of a plant cell wall pectin biosynthetic homogalacturonan:galacturonosyltransferase complex. Proc Natl Acad Sci U S A 2011;108:20225-30.**

**[39] Ridley BL, O'Neill MA, Mohnen D. Pectins: structure, biosynthesis, and oligogalacturonide-related signaling. Phytochemistry 2001;57:929-67.**

**[40] Philippe F, Pelloux J, Rayon C. Plant pectin acetylesterase structure and function: new insights from bioinformatic analysis. BMC Genomics 2017;18:456.**

**[41] Carpita NC, Gibeaut DM. Structural models of primary cell walls in flowering plants: consistency of molecular structure with the physical properties of the walls during growth. Plant J 1993;3:1-30.**

**[42] Mohnen D. Pectin structure and biosynthesis. Curr Opin Plant Biol 2008;11:266-77.**

**[43] Harholt J, Jensen JK, Sørensen SO, Orfila C, Pauly M, Scheller HV. ARABINAN DEFICIENT 1 is a putative arabinosyltransferase involved in biosynthesis of pectic arabinan in Arabidopsis. Plant Physiol 2006;140:49-58.**

**[44] Redgwell RJ, Curti D, Wang J, Dobruchowska JM, Gerwig GJ, Kamerling JP, et al. Cell wall polysaccharides of Chinese Wolfberry (Lycium barbarum): Part 2. Characterisation of arabinogalactan-proteins. Carbohydrate Polymers 2011;84:1075-83.**

**[45] Statello L, Guo CJ, Chen LL, Huarte M. Gene regulation by long non-coding RNAs and its biological functions. Nat Rev Mol Cell Biol 2021;22:96-118.**

**[46] Zhao L, Wang J, Li Y, Song T, Wu Y, Fang S, et al. NONCODEV6: an updated database dedicated to long non-coding RNA annotation in both animals and plants. Nucleic Acids Res 2021;49:D165-d71.**

**[47] Bailey TL, Johnson J, Grant CE, Noble WS. The MEME Suite. Nucleic Acids Res 2015;43:W39-49.**

**[48] Yin Y, Chen H, Hahn MG, Mohnen D, Xu Y. Evolution and function of the plant cell wall synthesis-related glycosyltransferase family 8. Plant Physiol 2010;153:1729-46.**

**[50] Wang L, Wang W, Wang YQ, Liu YY, Wang JX, Zhang XQ, et al. Arabidopsis galacturonosyltransferase (GAUT) 13 and GAUT14 have redundant functions in pollen tube growth. Mol Plant 2013;6:1131-48.**

**[51] Biswal AK, Hao Z, Pattathil S, Yang X, Winkeler K, Collins C, et al. Downregulation of GAUT12 in Populus deltoides by RNA silencing results in reduced recalcitrance, increased growth and reduced xylan and pectin in a woody biofuel feedstock. Biotechnol Biofuels 2015;8:41.**

**[1] Gao Y, Wei Y, Wang Y, Gao F, Chen Z. Lycium Barbarum: A Traditional Chinese Herb and A Promising Anti-Aging Agent. Aging Dis 2017;8:778-91.**

**[2] Sun C, Chen X, Yang S, Jin C, Ding K, Chen C. LBP1C-2 from Lycium barbarum alleviated age-related bone loss by targeting BMPRIA/BMPRII/Noggin. Carbohydr Polym 2023;310:120725.**

**[3] Potterat O. Goji (Lycium barbarum and L. chinense): Phytochemistry, pharmacology and safety in the perspective of traditional uses and recent popularity. Planta Med 2010;76:7-19.**

**[4] Zhang XJ, Yu HY, Cai YJ, Ke M. Lycium barbarum polysaccharides inhibit proliferation and migration of bladder cancer cell lines BIU87 by suppressing Pi3K/AKT pathway. Oncotarget 2017;8:5936-42.**

**[5] Tang L, Bao S, Du Y, Jiang Z, Wuliji AO, Ren X, et al. Antioxidant effects of Lycium barbarum polysaccharides on photoreceptor degeneration in the light-exposed mouse retina. Biomed Pharmacother 2018;103:829-37.**

**[6] Zhang W, Zhang J, Ding D, Zhang L, Muehlmann LA, Deng SE, et al. Synthesis and antioxidant properties of Lycium barbarum polysaccharides capped selenium nanoparticles using tea extract. Artif Cells Nanomed Biotechnol 2018;46:1463-70.**

**[7] Ma K, Wang X, Feng S, Xia X, Zhang H, Rahaman A, et al. From the perspective of Traditional Chinese Medicine: Treatment of mental disorders in COVID-19 survivors. Biomed Pharmacother 2020;132:110810.**

**[8] Po KK, Leung JW, Chan JN, Fung TK, Sánchez-Vidaña DI, Sin EL, et al. Protective effect of Lycium Barbarum polysaccharides on dextromethorphan-induced mood impairment and neurogenesis suppression. Brain Res Bull 2017;134:10-7.**

**[9] Fang S, Dong L, Liu L, Guo J, Zhao L, Zhang J, et al. HERB: a high-throughput experiment- and reference-guided database of traditional Chinese medicine. Nucleic Acids Res 2021;49:D1197-d206.**

**[10] Zhang S, He F, Chen X, Ding K. Isolation and structural characterization of a pectin from Lycium ruthenicum Murr and its anti-pancreatic ductal adenocarcinoma cell activity. Carbohydr Polym 2019;223:115104.**

**[11] Cao YL, Li YL, Fan YF, Li Z, Yoshida K, Wang JY, et al. Wolfberry genomes and the evolution of Lycium (Solanaceae). Commun Biol 2021;4:671.**

**[12] Chen J, Liu X, Zhu L, Wang Y. Nuclear genome size estimation and karyotype analysis of Lycium species (Solanaceae). Scientia Horticulturae 2013;151:46-50.**

**[13] Giri MK, Swain S, Gautam JK, Singh S, Singh N, Bhattacharjee L, et al. The Arabidopsis thaliana At4g13040 gene, a unique member of the AP2/EREBP family, is a positive regulator for salicylic acid accumulation and basal defense against bacterial pathogens. J Plant Physiol 2014;171:860-7.**

**[14] Michaels SD, Ditta G, Gustafson-Brown C, Pelaz S, Yanofsky M, Amasino RM. AGL24 acts as a promoter of flowering in Arabidopsis and is positively regulated by vernalization. Plant J 2003;33:867-74.**

**[15] Schauser L, Roussis A, Stiller J, Stougaard J. A plant regulator controlling development of symbiotic root nodules. Nature 1999;402:191-5.**

**[16] Hou X, Zhou J, Liu C, Liu L, Shen L, Yu H. Nuclear factor Y-mediated H3K27me3 demethylation of the SOC1 locus orchestrates flowering responses of Arabidopsis. Nat Commun 2014;5:4601.**

**[17] Ru L, He Y, Zhu Z, Patrick JW, Ruan YL. Integrating Sugar Metabolism With Transport: Elevation of Endogenous Cell Wall Invertase Activity Up-Regulates SlHT2 and SlSWEET12c Expression for Early Fruit Development in Tomato. Front Genet 2020;11:592596.**

**[18] Breia R, Conde A, Badim H, Fortes AM, Gerós H, Granell A. Plant SWEETs: from sugar transport to plant-pathogen interaction and more unexpected physiological roles. Plant Physiol 2021;186:836-52.**

**[19] Chen LQ, Hou BH, Lalonde S, Takanaga H, Hartung ML, Qu XQ, et al. Sugar transporters for intercellular exchange and nutrition of pathogens. Nature 2010;468:527-32.**

**[20] Eom JS, Chen LQ, Sosso D, Julius BT, Lin IW, Qu XQ, et al. SWEETs, transporters for intracellular and intercellular sugar translocation. Curr Opin Plant Biol 2015;25:53-62.**

**[21] Li Y, Feng S, Ma S, Sui X, Zhang Z. Spatiotemporal Expression and Substrate Specificity Analysis of the Cucumber SWEET Gene Family. Front Plant Sci 2017;8:1855.**

**[22] Patil G, Valliyodan B, Deshmukh R, Prince S, Nicander B, Zhao M, et al. Soybean (Glycine max) SWEET gene family: insights through comparative genomics, transcriptome profiling and whole genome re-sequence analysis. BMC Genomics 2015;16:520.**

**[23] Zhang W, Wang S, Yu F, Tang J, Shan X, Bao K, et al. Genome-wide characterization and expression profiling of SWEET genes in cabbage (Brassica oleracea var. capitata L.) reveal their roles in chilling and clubroot disease responses. BMC Genomics 2019;20:93.**

**[24] Feng CY, Han JX, Han XX, Jiang J. Genome-wide identification, phylogeny, and expression analysis of the SWEET gene family in tomato. Gene 2015;573:261-72.**

**[25] Manck-Götzenberger J, Requena N. Arbuscular mycorrhiza Symbiosis Induces a Major Transcriptional Reprogramming of the Potato SWEET Sugar Transporter Family. Front Plant Sci 2016;7:487.**

**[26] Ko HY, Ho LH, Neuhaus HE, Guo WJ. Transporter SlSWEET15 unloads sucrose from phloem and seed coat for fruit and seed development in tomato. Plant Physiol 2021;187:2230-45.**

**[27] Zhang X, Feng C, Wang M, Li T, Liu X, Jiang J. Plasma membrane-localized SlSWEET7a and SlSWEET14 regulate sugar transport and storage in tomato fruits. Hortic Res 2021;8:186.**

**[28] Pan L, Guo Q, Chai S, Cheng Y, Ruan M, Ye Q, et al. Evolutionary Conservation and Expression Patterns of Neutral/Alkaline Invertases in Solanum. Biomolecules 2019;9.**

**[29] Veillet F, Gaillard C, Coutos-Thévenot P, La Camera S. Targeting the AtCWIN1 Gene to Explore the Role of Invertases in Sucrose Transport in Roots and during Botrytis cinerea Infection. Front Plant Sci 2016;7:1899.**

**[30] Wan H, Wu L, Yang Y, Zhou G, Ruan YL. Evolution of Sucrose Metabolism: The Dichotomy of Invertases and Beyond. Trends Plant Sci 2018;23:163-77.**

**[31] Jin Y, Ni DA, Ruan YL. Posttranslational elevation of cell wall invertase activity by silencing its inhibitor in tomato delays leaf senescence and increases seed weight and fruit hexose level. Plant Cell 2009;21:2072-89.**

**[32] Shen S, Ma S, Liu Y, Liao S, Li J, Wu L, et al. Cell Wall Invertase and Sugar Transporters Are Differentially Activated in Tomato Styles and Ovaries During Pollination and Fertilization. Front Plant Sci 2019;10:506.**

**[33] Lombard V, Golaconda Ramulu H, Drula E, Coutinho PM, Henrissat B. The carbohydrate-active enzymes database (CAZy) in 2013. Nucleic Acids Res 2014;42:D490-5.**

**[35] Langfelder P, Horvath S. WGCNA: an R package for weighted correlation network analysis. BMC Bioinformatics 2008;9:559.**

**[36] Kaczmarska A, Pieczywek PM, Cybulska J, Zdunek A. Structure and functionality of Rhamnogalacturonan I in the cell wall and in solution: A review. Carbohydr Polym 2022;278:118909.**

**[37] Wachananawat B, Kuroha T, Takenaka Y, Kajiura H, Naramoto S, Yokoyama R, et al. Diversity of Pectin Rhamnogalacturonan I Rhamnosyltransferases in Glycosyltransferase Family 106. Front Plant Sci 2020;11:997.**

**[38] Atmodjo MA, Sakuragi Y, Zhu X, Burrell AJ, Mohanty SS, Atwood JA, 3rd, et al. Galacturonosyltransferase (GAUT)1 and GAUT7 are the core of a plant cell wall pectin biosynthetic homogalacturonan:galacturonosyltransferase complex. Proc Natl Acad Sci U S A 2011;108:20225-30.**

**[39] Ridley BL, O'Neill MA, Mohnen D. Pectins: structure, biosynthesis, and oligogalacturonide-related signaling. Phytochemistry 2001;57:929-67.**

**[40] Philippe F, Pelloux J, Rayon C. Plant pectin acetylesterase structure and function: new insights from bioinformatic analysis. BMC Genomics 2017;18:456.**

**[41] Carpita NC, Gibeaut DM. Structural models of primary cell walls in flowering plants: consistency of molecular structure with the physical properties of the walls during growth. Plant J 1993;3:1-30.**

**[42] Mohnen D. Pectin structure and biosynthesis. Curr Opin Plant Biol 2008;11:266-77.**

**[43] Harholt J, Jensen JK, Sørensen SO, Orfila C, Pauly M, Scheller HV. ARABINAN DEFICIENT 1 is a putative arabinosyltransferase involved in biosynthesis of pectic arabinan in Arabidopsis. Plant Physiol 2006;140:49-58.**

**[44] Redgwell RJ, Curti D, Wang J, Dobruchowska JM, Gerwig GJ, Kamerling JP, et al. Cell wall polysaccharides of Chinese Wolfberry (Lycium barbarum): Part 2. Characterisation of arabinogalactan-proteins. Carbohydrate Polymers 2011;84:1075-83.**

**[45] Statello L, Guo CJ, Chen LL, Huarte M. Gene regulation by long non-coding RNAs and its biological functions. Nat Rev Mol Cell Biol 2021;22:96-118.**

**[46] Zhao L, Wang J, Li Y, Song T, Wu Y, Fang S, et al. NONCODEV6: an updated database dedicated to long non-coding RNA annotation in both animals and plants. Nucleic Acids Res 2021;49:D165-d71.**

**[47] Bailey TL, Johnson J, Grant CE, Noble WS. The MEME Suite. Nucleic Acids Res 2015;43:W39-49.**

**[48] Yin Y, Chen H, Hahn MG, Mohnen D, Xu Y. Evolution and function of the plant cell wall synthesis-related glycosyltransferase family 8. Plant Physiol 2010;153:1729-46.**

**[49] Caffall KH, Pattathil S, Phillips SE, Hahn MG, Mohnen D. Arabidopsis thaliana T-DNA mutants implicate GAUT genes in the biosynthesis of pectin and xylan in cell walls and seed testa. Mol Plant 2009;2:1000-14.**

**[50] Wang L, Wang W, Wang YQ, Liu YY, Wang JX, Zhang XQ, et al. Arabidopsis galacturonosyltransferase (GAUT) 13 and GAUT14 have redundant functions in pollen tube growth. Mol Plant 2013;6:1131-48.**

**[1] Gao Y, Wei Y, Wang Y, Gao F, Chen Z. Lycium Barbarum: A Traditional Chinese Herb and A Promising Anti-Aging Agent. Aging Dis 2017;8:778-91.**

**[2] Sun C, Chen X, Yang S, Jin C, Ding K, Chen C. LBP1C-2 from Lycium barbarum alleviated age-related bone loss by targeting BMPRIA/BMPRII/Noggin. Carbohydr Polym 2023;310:120725.**

**[3] Potterat O. Goji (Lycium barbarum and L. chinense): Phytochemistry, pharmacology and safety in the perspective of traditional uses and recent popularity. Planta Med 2010;76:7-19.**

**[4] Zhang XJ, Yu HY, Cai YJ, Ke M. Lycium barbarum polysaccharides inhibit proliferation and migration of bladder cancer cell lines BIU87 by suppressing Pi3K/AKT pathway. Oncotarget 2017;8:5936-42.**

**[5] Tang L, Bao S, Du Y, Jiang Z, Wuliji AO, Ren X, et al. Antioxidant effects of Lycium barbarum polysaccharides on photoreceptor degeneration in the light-exposed mouse retina. Biomed Pharmacother 2018;103:829-37.**

**[6] Zhang W, Zhang J, Ding D, Zhang L, Muehlmann LA, Deng SE, et al. Synthesis and antioxidant properties of Lycium barbarum polysaccharides capped selenium nanoparticles using tea extract. Artif Cells Nanomed Biotechnol 2018;46:1463-70.**

**[7] Ma K, Wang X, Feng S, Xia X, Zhang H, Rahaman A, et al. From the perspective of Traditional Chinese Medicine: Treatment of mental disorders in COVID-19 survivors. Biomed Pharmacother 2020;132:110810.**

**[8] Po KK, Leung JW, Chan JN, Fung TK, Sánchez-Vidaña DI, Sin EL, et al. Protective effect of Lycium Barbarum polysaccharides on dextromethorphan-induced mood impairment and neurogenesis suppression. Brain Res Bull 2017;134:10-7.**

**[9] Fang S, Dong L, Liu L, Guo J, Zhao L, Zhang J, et al. HERB: a high-throughput experiment- and reference-guided database of traditional Chinese medicine. Nucleic Acids Res 2021;49:D1197-d206.**

**[10] Zhang S, He F, Chen X, Ding K. Isolation and structural characterization of a pectin from Lycium ruthenicum Murr and its anti-pancreatic ductal adenocarcinoma cell activity. Carbohydr Polym 2019;223:115104.**

**[11] Cao YL, Li YL, Fan YF, Li Z, Yoshida K, Wang JY, et al. Wolfberry genomes and the evolution of Lycium (Solanaceae). Commun Biol 2021;4:671.**

**[12] Chen J, Liu X, Zhu L, Wang Y. Nuclear genome size estimation and karyotype analysis of Lycium species (Solanaceae). Scientia Horticulturae 2013;151:46-50.**

**[13] Giri MK, Swain S, Gautam JK, Singh S, Singh N, Bhattacharjee L, et al. The Arabidopsis thaliana At4g13040 gene, a unique member of the AP2/EREBP family, is a positive regulator for salicylic acid accumulation and basal defense against bacterial pathogens. J Plant Physiol 2014;171:860-7.**

**[14] Michaels SD, Ditta G, Gustafson-Brown C, Pelaz S, Yanofsky M, Amasino RM. AGL24 acts as a promoter of flowering in Arabidopsis and is positively regulated by vernalization. Plant J 2003;33:867-74.**

**[15] Schauser L, Roussis A, Stiller J, Stougaard J. A plant regulator controlling development of symbiotic root nodules. Nature 1999;402:191-5.**

**[16] Hou X, Zhou J, Liu C, Liu L, Shen L, Yu H. Nuclear factor Y-mediated H3K27me3 demethylation of the SOC1 locus orchestrates flowering responses of Arabidopsis. Nat Commun 2014;5:4601.**

**[17] Ru L, He Y, Zhu Z, Patrick JW, Ruan YL. Integrating Sugar Metabolism With Transport: Elevation of Endogenous Cell Wall Invertase Activity Up-Regulates SlHT2 and SlSWEET12c Expression for Early Fruit Development in Tomato. Front Genet 2020;11:592596.**

**[18] Breia R, Conde A, Badim H, Fortes AM, Gerós H, Granell A. Plant SWEETs: from sugar transport to plant-pathogen interaction and more unexpected physiological roles. Plant Physiol 2021;186:836-52.**

**[19] Chen LQ, Hou BH, Lalonde S, Takanaga H, Hartung ML, Qu XQ, et al. Sugar transporters for intercellular exchange and nutrition of pathogens. Nature 2010;468:527-32.**

**[20] Eom JS, Chen LQ, Sosso D, Julius BT, Lin IW, Qu XQ, et al. SWEETs, transporters for intracellular and intercellular sugar translocation. Curr Opin Plant Biol 2015;25:53-62.**

**[21] Li Y, Feng S, Ma S, Sui X, Zhang Z. Spatiotemporal Expression and Substrate Specificity Analysis of the Cucumber SWEET Gene Family. Front Plant Sci 2017;8:1855.**

**[22] Patil G, Valliyodan B, Deshmukh R, Prince S, Nicander B, Zhao M, et al. Soybean (Glycine max) SWEET gene family: insights through comparative genomics, transcriptome profiling and whole genome re-sequence analysis. BMC Genomics 2015;16:520.**

**[23] Zhang W, Wang S, Yu F, Tang J, Shan X, Bao K, et al. Genome-wide characterization and expression profiling of SWEET genes in cabbage (Brassica oleracea var. capitata L.) reveal their roles in chilling and clubroot disease responses. BMC Genomics 2019;20:93.**

**[24] Feng CY, Han JX, Han XX, Jiang J. Genome-wide identification, phylogeny, and expression analysis of the SWEET gene family in tomato. Gene 2015;573:261-72.**

**[25] Manck-Götzenberger J, Requena N. Arbuscular mycorrhiza Symbiosis Induces a Major Transcriptional Reprogramming of the Potato SWEET Sugar Transporter Family. Front Plant Sci 2016;7:487.**

**[26] Ko HY, Ho LH, Neuhaus HE, Guo WJ. Transporter SlSWEET15 unloads sucrose from phloem and seed coat for fruit and seed development in tomato. Plant Physiol 2021;187:2230-45.**

**[27] Zhang X, Feng C, Wang M, Li T, Liu X, Jiang J. Plasma membrane-localized SlSWEET7a and SlSWEET14 regulate sugar transport and storage in tomato fruits. Hortic Res 2021;8:186.**

**[28] Pan L, Guo Q, Chai S, Cheng Y, Ruan M, Ye Q, et al. Evolutionary Conservation and Expression Patterns of Neutral/Alkaline Invertases in Solanum. Biomolecules 2019;9.**

**[29] Veillet F, Gaillard C, Coutos-Thévenot P, La Camera S. Targeting the AtCWIN1 Gene to Explore the Role of Invertases in Sucrose Transport in Roots and during Botrytis cinerea Infection. Front Plant Sci 2016;7:1899.**

**[30] Wan H, Wu L, Yang Y, Zhou G, Ruan YL. Evolution of Sucrose Metabolism: The Dichotomy of Invertases and Beyond. Trends Plant Sci 2018;23:163-77.**

**[31] Jin Y, Ni DA, Ruan YL. Posttranslational elevation of cell wall invertase activity by silencing its inhibitor in tomato delays leaf senescence and increases seed weight and fruit hexose level. Plant Cell 2009;21:2072-89.**

**[32] Shen S, Ma S, Liu Y, Liao S, Li J, Wu L, et al. Cell Wall Invertase and Sugar Transporters Are Differentially Activated in Tomato Styles and Ovaries During Pollination and Fertilization. Front Plant Sci 2019;10:506.**

**[33] Lombard V, Golaconda Ramulu H, Drula E, Coutinho PM, Henrissat B. The carbohydrate-active enzymes database (CAZy) in 2013. Nucleic Acids Res 2014;42:D490-5.**

**[34] Zhang H, Yohe T, Huang L, Entwistle S, Wu P, Yang Z, et al. dbCAN2: a meta server for automated carbohydrate-active enzyme annotation. Nucleic Acids Res 2018;46:W95-w101.**

**[35] Langfelder P, Horvath S. WGCNA: an R package for weighted correlation network analysis. BMC Bioinformatics 2008;9:559.**

**[36] Kaczmarska A, Pieczywek PM, Cybulska J, Zdunek A. Structure and functionality of Rhamnogalacturonan I in the cell wall and in solution: A review. Carbohydr Polym 2022;278:118909.**

**[37] Wachananawat B, Kuroha T, Takenaka Y, Kajiura H, Naramoto S, Yokoyama R, et al. Diversity of Pectin Rhamnogalacturonan I Rhamnosyltransferases in Glycosyltransferase Family 106. Front Plant Sci 2020;11:997.**

**[39] Ridley BL, O'Neill MA, Mohnen D. Pectins: structure, biosynthesis, and oligogalacturonide-related signaling. Phytochemistry 2001;57:929-67.**

**[40] Philippe F, Pelloux J, Rayon C. Plant pectin acetylesterase structure and function: new insights from bioinformatic analysis. BMC Genomics 2017;18:456.**

**[41] Carpita NC, Gibeaut DM. Structural models of primary cell walls in flowering plants: consistency of molecular structure with the physical properties of the walls during growth. Plant J 1993;3:1-30.**

**[42] Mohnen D. Pectin structure and biosynthesis. Curr Opin Plant Biol 2008;11:266-77.**

**[43] Harholt J, Jensen JK, Sørensen SO, Orfila C, Pauly M, Scheller HV. ARABINAN DEFICIENT 1 is a putative arabinosyltransferase involved in biosynthesis of pectic arabinan in Arabidopsis. Plant Physiol 2006;140:49-58.**

**[44] Redgwell RJ, Curti D, Wang J, Dobruchowska JM, Gerwig GJ, Kamerling JP, et al. Cell wall polysaccharides of Chinese Wolfberry (Lycium barbarum): Part 2. Characterisation of arabinogalactan-proteins. Carbohydrate Polymers 2011;84:1075-83.**

**[45] Statello L, Guo CJ, Chen LL, Huarte M. Gene regulation by long non-coding RNAs and its biological functions. Nat Rev Mol Cell Biol 2021;22:96-118.**

**[46] Zhao L, Wang J, Li Y, Song T, Wu Y, Fang S, et al. NONCODEV6: an updated database dedicated to long non-coding RNA annotation in both animals and plants. Nucleic Acids Res 2021;49:D165-d71.**

**[47] Bailey TL, Johnson J, Grant CE, Noble WS. The MEME Suite. Nucleic Acids Res 2015;43:W39-49.**

**[48] Yin Y, Chen H, Hahn MG, Mohnen D, Xu Y. Evolution and function of the plant cell wall synthesis-related glycosyltransferase family 8. Plant Physiol 2010;153:1729-46.**

**[49] Caffall KH, Pattathil S, Phillips SE, Hahn MG, Mohnen D. Arabidopsis thaliana T-DNA mutants implicate GAUT genes in the biosynthesis of pectin and xylan in cell walls and seed testa. Mol Plant 2009;2:1000-14.**

**[50] Wang L, Wang W, Wang YQ, Liu YY, Wang JX, Zhang XQ, et al. Arabidopsis galacturonosyltransferase (GAUT) 13 and GAUT14 have redundant functions in pollen tube growth. Mol Plant 2013;6:1131-48.**

**[51] Biswal AK, Hao Z, Pattathil S, Yang X, Winkeler K, Collins C, et al. Downregulation of GAUT12 in Populus deltoides by RNA silencing results in reduced recalcitrance, increased growth and reduced xylan and pectin in a woody biofuel feedstock. Biotechnol Biofuels 2015;8:41.**

**[1] Gao Y, Wei Y, Wang Y, Gao F, Chen Z. Lycium Barbarum: A Traditional Chinese Herb and A Promising Anti-Aging Agent. Aging Dis 2017;8:778-91.**

**[2] Sun C, Chen X, Yang S, Jin C, Ding K, Chen C. LBP1C-2 from Lycium barbarum alleviated age-related bone loss by targeting BMPRIA/BMPRII/Noggin. Carbohydr Polym 2023;310:120725.**

**[3] Potterat O. Goji (Lycium barbarum and L. chinense): Phytochemistry, pharmacology and safety in the perspective of traditional uses and recent popularity. Planta Med 2010;76:7-19.**

**[4] Zhang XJ, Yu HY, Cai YJ, Ke M. Lycium barbarum polysaccharides inhibit proliferation and migration of bladder cancer cell lines BIU87 by suppressing Pi3K/AKT pathway. Oncotarget 2017;8:5936-42.**

**[5] Tang L, Bao S, Du Y, Jiang Z, Wuliji AO, Ren X, et al. Antioxidant effects of Lycium barbarum polysaccharides on photoreceptor degeneration in the light-exposed mouse retina. Biomed Pharmacother 2018;103:829-37.**

**[7] Ma K, Wang X, Feng S, Xia X, Zhang H, Rahaman A, et al. From the perspective of Traditional Chinese Medicine: Treatment of mental disorders in COVID-19 survivors. Biomed Pharmacother 2020;132:110810.**

**[8] Po KK, Leung JW, Chan JN, Fung TK, Sánchez-Vidaña DI, Sin EL, et al. Protective effect of Lycium Barbarum polysaccharides on dextromethorphan-induced mood impairment and neurogenesis suppression. Brain Res Bull 2017;134:10-7.**

**[9] Fang S, Dong L, Liu L, Guo J, Zhao L, Zhang J, et al. HERB: a high-throughput experiment- and reference-guided database of traditional Chinese medicine. Nucleic Acids Res 2021;49:D1197-d206.**

**[10] Zhang S, He F, Chen X, Ding K. Isolation and structural characterization of a pectin from Lycium ruthenicum Murr and its anti-pancreatic ductal adenocarcinoma cell activity. Carbohydr Polym 2019;223:115104.**

**[11] Cao YL, Li YL, Fan YF, Li Z, Yoshida K, Wang JY, et al. Wolfberry genomes and the evolution of Lycium (Solanaceae). Commun Biol 2021;4:671.**

**[12] Chen J, Liu X, Zhu L, Wang Y. Nuclear genome size estimation and karyotype analysis of Lycium species (Solanaceae). Scientia Horticulturae 2013;151:46-50.**

**[13] Giri MK, Swain S, Gautam JK, Singh S, Singh N, Bhattacharjee L, et al. The Arabidopsis thaliana At4g13040 gene, a unique member of the AP2/EREBP family, is a positive regulator for salicylic acid accumulation and basal defense against bacterial pathogens. J Plant Physiol 2014;171:860-7.**

**[14] Michaels SD, Ditta G, Gustafson-Brown C, Pelaz S, Yanofsky M, Amasino RM. AGL24 acts as a promoter of flowering in Arabidopsis and is positively regulated by vernalization. Plant J 2003;33:867-74.**

**[15] Schauser L, Roussis A, Stiller J, Stougaard J. A plant regulator controlling development of symbiotic root nodules. Nature 1999;402:191-5.**

**[16] Hou X, Zhou J, Liu C, Liu L, Shen L, Yu H. Nuclear factor Y-mediated H3K27me3 demethylation of the SOC1 locus orchestrates flowering responses of Arabidopsis. Nat Commun 2014;5:4601.**

**[17] Ru L, He Y, Zhu Z, Patrick JW, Ruan YL. Integrating Sugar Metabolism With Transport: Elevation of Endogenous Cell Wall Invertase Activity Up-Regulates SlHT2 and SlSWEET12c Expression for Early Fruit Development in Tomato. Front Genet 2020;11:592596.**

**[18] Breia R, Conde A, Badim H, Fortes AM, Gerós H, Granell A. Plant SWEETs: from sugar transport to plant-pathogen interaction and more unexpected physiological roles. Plant Physiol 2021;186:836-52.**

**[19] Chen LQ, Hou BH, Lalonde S, Takanaga H, Hartung ML, Qu XQ, et al. Sugar transporters for intercellular exchange and nutrition of pathogens. Nature 2010;468:527-32.**

**[20] Eom JS, Chen LQ, Sosso D, Julius BT, Lin IW, Qu XQ, et al. SWEETs, transporters for intracellular and intercellular sugar translocation. Curr Opin Plant Biol 2015;25:53-62.**

**[21] Li Y, Feng S, Ma S, Sui X, Zhang Z. Spatiotemporal Expression and Substrate Specificity Analysis of the Cucumber SWEET Gene Family. Front Plant Sci 2017;8:1855.**

**[22] Patil G, Valliyodan B, Deshmukh R, Prince S, Nicander B, Zhao M, et al. Soybean (Glycine max) SWEET gene family: insights through comparative genomics, transcriptome profiling and whole genome re-sequence analysis. BMC Genomics 2015;16:520.**

**[23] Zhang W, Wang S, Yu F, Tang J, Shan X, Bao K, et al. Genome-wide characterization and expression profiling of SWEET genes in cabbage (Brassica oleracea var. capitata L.) reveal their roles in chilling and clubroot disease responses. BMC Genomics 2019;20:93.**

**[24] Feng CY, Han JX, Han XX, Jiang J. Genome-wide identification, phylogeny, and expression analysis of the SWEET gene family in tomato. Gene 2015;573:261-72.**

**[25] Manck-Götzenberger J, Requena N. Arbuscular mycorrhiza Symbiosis Induces a Major Transcriptional Reprogramming of the Potato SWEET Sugar Transporter Family. Front Plant Sci 2016;7:487.**

**[26] Ko HY, Ho LH, Neuhaus HE, Guo WJ. Transporter SlSWEET15 unloads sucrose from phloem and seed coat for fruit and seed development in tomato. Plant Physiol 2021;187:2230-45.**

**[27] Zhang X, Feng C, Wang M, Li T, Liu X, Jiang J. Plasma membrane-localized SlSWEET7a and SlSWEET14 regulate sugar transport and storage in tomato fruits. Hortic Res 2021;8:186.**

**[28] Pan L, Guo Q, Chai S, Cheng Y, Ruan M, Ye Q, et al. Evolutionary Conservation and Expression Patterns of Neutral/Alkaline Invertases in Solanum. Biomolecules 2019;9.**

**[29] Veillet F, Gaillard C, Coutos-Thévenot P, La Camera S. Targeting the AtCWIN1 Gene to Explore the Role of Invertases in Sucrose Transport in Roots and during Botrytis cinerea Infection. Front Plant Sci 2016;7:1899.**

**[30] Wan H, Wu L, Yang Y, Zhou G, Ruan YL. Evolution of Sucrose Metabolism: The Dichotomy of Invertases and Beyond. Trends Plant Sci 2018;23:163-77.**

**[32] Shen S, Ma S, Liu Y, Liao S, Li J, Wu L, et al. Cell Wall Invertase and Sugar Transporters Are Differentially Activated in Tomato Styles and Ovaries During Pollination and Fertilization. Front Plant Sci 2019;10:506.**

**[33] Lombard V, Golaconda Ramulu H, Drula E, Coutinho PM, Henrissat B. The carbohydrate-active enzymes database (CAZy) in 2013. Nucleic Acids Res 2014;42:D490-5.**

**[34] Zhang H, Yohe T, Huang L, Entwistle S, Wu P, Yang Z, et al. dbCAN2: a meta server for automated carbohydrate-active enzyme annotation. Nucleic Acids Res 2018;46:W95-w101.**

**[35] Langfelder P, Horvath S. WGCNA: an R package for weighted correlation network analysis. BMC Bioinformatics 2008;9:559.**

**[36] Kaczmarska A, Pieczywek PM, Cybulska J, Zdunek A. Structure and functionality of Rhamnogalacturonan I in the cell wall and in solution: A review. Carbohydr Polym 2022;278:118909.**

**[37] Wachananawat B, Kuroha T, Takenaka Y, Kajiura H, Naramoto S, Yokoyama R, et al. Diversity of Pectin Rhamnogalacturonan I Rhamnosyltransferases in Glycosyltransferase Family 106. Front Plant Sci 2020;11:997.**

**[38] Atmodjo MA, Sakuragi Y, Zhu X, Burrell AJ, Mohanty SS, Atwood JA, 3rd, et al. Galacturonosyltransferase (GAUT)1 and GAUT7 are the core of a plant cell wall pectin biosynthetic homogalacturonan:galacturonosyltransferase complex. Proc Natl Acad Sci U S A 2011;108:20225-30.**

**[39] Ridley BL, O'Neill MA, Mohnen D. Pectins: structure, biosynthesis, and oligogalacturonide-related signaling. Phytochemistry 2001;57:929-67.**

**[40] Philippe F, Pelloux J, Rayon C. Plant pectin acetylesterase structure and function: new insights from bioinformatic analysis. BMC Genomics 2017;18:456.**

**[41] Carpita NC, Gibeaut DM. Structural models of primary cell walls in flowering plants: consistency of molecular structure with the physical properties of the walls during growth. Plant J 1993;3:1-30.**

**[42] Mohnen D. Pectin structure and biosynthesis. Curr Opin Plant Biol 2008;11:266-77.**

**[43] Harholt J, Jensen JK, Sørensen SO, Orfila C, Pauly M, Scheller HV. ARABINAN DEFICIENT 1 is a putative arabinosyltransferase involved in biosynthesis of pectic arabinan in Arabidopsis. Plant Physiol 2006;140:49-58.**

**[44] Redgwell RJ, Curti D, Wang J, Dobruchowska JM, Gerwig GJ, Kamerling JP, et al. Cell wall polysaccharides of Chinese Wolfberry (Lycium barbarum): Part 2. Characterisation of arabinogalactan-proteins. Carbohydrate Polymers 2011;84:1075-83.**

**[45] Statello L, Guo CJ, Chen LL, Huarte M. Gene regulation by long non-coding RNAs and its biological functions. Nat Rev Mol Cell Biol 2021;22:96-118.**

**[46] Zhao L, Wang J, Li Y, Song T, Wu Y, Fang S, et al. NONCODEV6: an updated database dedicated to long non-coding RNA annotation in both animals and plants. Nucleic Acids Res 2021;49:D165-d71.**

**[47] Bailey TL, Johnson J, Grant CE, Noble WS. The MEME Suite. Nucleic Acids Res 2015;43:W39-49.**

**[48] Yin Y, Chen H, Hahn MG, Mohnen D, Xu Y. Evolution and function of the plant cell wall synthesis-related glycosyltransferase family 8. Plant Physiol 2010;153:1729-46.**

**[49] Caffall KH, Pattathil S, Phillips SE, Hahn MG, Mohnen D. Arabidopsis thaliana T-DNA mutants implicate GAUT genes in the biosynthesis of pectin and xylan in cell walls and seed testa. Mol Plant 2009;2:1000-14.**

**[50] Wang L, Wang W, Wang YQ, Liu YY, Wang JX, Zhang XQ, et al. Arabidopsis galacturonosyltransferase (GAUT) 13 and GAUT14 have redundant functions in pollen tube growth. Mol Plant 2013;6:1131-48.**

**[51] Biswal AK, Hao Z, Pattathil S, Yang X, Winkeler K, Collins C, et al. Downregulation of GAUT12 in Populus deltoides by RNA silencing results in reduced recalcitrance, increased growth and reduced xylan and pectin in a woody biofuel feedstock. Biotechnol Biofuels 2015;8:41.**

**[1] Gao Y, Wei Y, Wang Y, Gao F, Chen Z. Lycium Barbarum: A Traditional Chinese Herb and A Promising Anti-Aging Agent. Aging Dis 2017;8:778-91.**

**[2] Sun C, Chen X, Yang S, Jin C, Ding K, Chen C. LBP1C-2 from Lycium barbarum alleviated age-related bone loss by targeting BMPRIA/BMPRII/Noggin. Carbohydr Polym 2023;310:120725.**

**[3] Potterat O. Goji (Lycium barbarum and L. chinense): Phytochemistry, pharmacology and safety in the perspective of traditional uses and recent popularity. Planta Med 2010;76:7-19.**

**[4] Zhang XJ, Yu HY, Cai YJ, Ke M. Lycium barbarum polysaccharides inhibit proliferation and migration of bladder cancer cell lines BIU87 by suppressing Pi3K/AKT pathway. Oncotarget 2017;8:5936-42.**

**[5] Tang L, Bao S, Du Y, Jiang Z, Wuliji AO, Ren X, et al. Antioxidant effects of Lycium barbarum polysaccharides on photoreceptor degeneration in the light-exposed mouse retina. Biomed Pharmacother 2018;103:829-37.**

**[6] Zhang W, Zhang J, Ding D, Zhang L, Muehlmann LA, Deng SE, et al. Synthesis and antioxidant properties of Lycium barbarum polysaccharides capped selenium nanoparticles using tea extract. Artif Cells Nanomed Biotechnol 2018;46:1463-70.**

**[7] Ma K, Wang X, Feng S, Xia X, Zhang H, Rahaman A, et al. From the perspective of Traditional Chinese Medicine: Treatment of mental disorders in COVID-19 survivors. Biomed Pharmacother 2020;132:110810.**

**[8] Po KK, Leung JW, Chan JN, Fung TK, Sánchez-Vidaña DI, Sin EL, et al. Protective effect of Lycium Barbarum polysaccharides on dextromethorphan-induced mood impairment and neurogenesis suppression. Brain Res Bull 2017;134:10-7.**

**[9] Fang S, Dong L, Liu L, Guo J, Zhao L, Zhang J, et al. HERB: a high-throughput experiment- and reference-guided database of traditional Chinese medicine. Nucleic Acids Res 2021;49:D1197-d206.**

**[10] Zhang S, He F, Chen X, Ding K. Isolation and structural characterization of a pectin from Lycium ruthenicum Murr and its anti-pancreatic ductal adenocarcinoma cell activity. Carbohydr Polym 2019;223:115104.**

**[11] Cao YL, Li YL, Fan YF, Li Z, Yoshida K, Wang JY, et al. Wolfberry genomes and the evolution of Lycium (Solanaceae). Commun Biol 2021;4:671.**

**[12] Chen J, Liu X, Zhu L, Wang Y. Nuclear genome size estimation and karyotype analysis of Lycium species (Solanaceae). Scientia Horticulturae 2013;151:46-50.**

**[13] Giri MK, Swain S, Gautam JK, Singh S, Singh N, Bhattacharjee L, et al. The Arabidopsis thaliana At4g13040 gene, a unique member of the AP2/EREBP family, is a positive regulator for salicylic acid accumulation and basal defense against bacterial pathogens. J Plant Physiol 2014;171:860-7.**

**[14] Michaels SD, Ditta G, Gustafson-Brown C, Pelaz S, Yanofsky M, Amasino RM. AGL24 acts as a promoter of flowering in Arabidopsis and is positively regulated by vernalization. Plant J 2003;33:867-74.**

**[15] Schauser L, Roussis A, Stiller J, Stougaard J. A plant regulator controlling development of symbiotic root nodules. Nature 1999;402:191-5.**

**[16] Hou X, Zhou J, Liu C, Liu L, Shen L, Yu H. Nuclear factor Y-mediated H3K27me3 demethylation of the SOC1 locus orchestrates flowering responses of Arabidopsis. Nat Commun 2014;5:4601.**

**[17] Ru L, He Y, Zhu Z, Patrick JW, Ruan YL. Integrating Sugar Metabolism With Transport: Elevation of Endogenous Cell Wall Invertase Activity Up-Regulates SlHT2 and SlSWEET12c Expression for Early Fruit Development in Tomato. Front Genet 2020;11:592596.**

**[18] Breia R, Conde A, Badim H, Fortes AM, Gerós H, Granell A. Plant SWEETs: from sugar transport to plant-pathogen interaction and more unexpected physiological roles. Plant Physiol 2021;186:836-52.**

**[19] Chen LQ, Hou BH, Lalonde S, Takanaga H, Hartung ML, Qu XQ, et al. Sugar transporters for intercellular exchange and nutrition of pathogens. Nature 2010;468:527-32.**

**[20] Eom JS, Chen LQ, Sosso D, Julius BT, Lin IW, Qu XQ, et al. SWEETs, transporters for intracellular and intercellular sugar translocation. Curr Opin Plant Biol 2015;25:53-62.**

**[21] Li Y, Feng S, Ma S, Sui X, Zhang Z. Spatiotemporal Expression and Substrate Specificity Analysis of the Cucumber SWEET Gene Family. Front Plant Sci 2017;8:1855.**

**[22] Patil G, Valliyodan B, Deshmukh R, Prince S, Nicander B, Zhao M, et al. Soybean (Glycine max) SWEET gene family: insights through comparative genomics, transcriptome profiling and whole genome re-sequence analysis. BMC Genomics 2015;16:520.**

**[23] Zhang W, Wang S, Yu F, Tang J, Shan X, Bao K, et al. Genome-wide characterization and expression profiling of SWEET genes in cabbage (Brassica oleracea var. capitata L.) reveal their roles in chilling and clubroot disease responses. BMC Genomics 2019;20:93.**

**[24] Feng CY, Han JX, Han XX, Jiang J. Genome-wide identification, phylogeny, and expression analysis of the SWEET gene family in tomato. Gene 2015;573:261-72.**

**[25] Manck-Götzenberger J, Requena N. Arbuscular mycorrhiza Symbiosis Induces a Major Transcriptional Reprogramming of the Potato SWEET Sugar Transporter Family. Front Plant Sci 2016;7:487.**

**[26] Ko HY, Ho LH, Neuhaus HE, Guo WJ. Transporter SlSWEET15 unloads sucrose from phloem and seed coat for fruit and seed development in tomato. Plant Physiol 2021;187:2230-45.**

**[27] Zhang X, Feng C, Wang M, Li T, Liu X, Jiang J. Plasma membrane-localized SlSWEET7a and SlSWEET14 regulate sugar transport and storage in tomato fruits. Hortic Res 2021;8:186.**

**[28] Pan L, Guo Q, Chai S, Cheng Y, Ruan M, Ye Q, et al. Evolutionary Conservation and Expression Patterns of Neutral/Alkaline Invertases in Solanum. Biomolecules 2019;9.**

**[29] Veillet F, Gaillard C, Coutos-Thévenot P, La Camera S. Targeting the AtCWIN1 Gene to Explore the Role of Invertases in Sucrose Transport in Roots and during Botrytis cinerea Infection. Front Plant Sci 2016;7:1899.**

**[30] Wan H, Wu L, Yang Y, Zhou G, Ruan YL. Evolution of Sucrose Metabolism: The Dichotomy of Invertases and Beyond. Trends Plant Sci 2018;23:163-77.**

**[31] Jin Y, Ni DA, Ruan YL. Posttranslational elevation of cell wall invertase activity by silencing its inhibitor in tomato delays leaf senescence and increases seed weight and fruit hexose level. Plant Cell 2009;21:2072-89.**

**[32] Shen S, Ma S, Liu Y, Liao S, Li J, Wu L, et al. Cell Wall Invertase and Sugar Transporters Are Differentially Activated in Tomato Styles and Ovaries During Pollination and Fertilization. Front Plant Sci 2019;10:506.**

**[33] Lombard V, Golaconda Ramulu H, Drula E, Coutinho PM, Henrissat B. The carbohydrate-active enzymes database (CAZy) in 2013. Nucleic Acids Res 2014;42:D490-5.**

**[34] Zhang H, Yohe T, Huang L, Entwistle S, Wu P, Yang Z, et al. dbCAN2: a meta server for automated carbohydrate-active enzyme annotation. Nucleic Acids Res 2018;46:W95-w101.**

**[35] Langfelder P, Horvath S. WGCNA: an R package for weighted correlation network analysis. BMC Bioinformatics 2008;9:559.**

**[36] Kaczmarska A, Pieczywek PM, Cybulska J, Zdunek A. Structure and functionality of Rhamnogalacturonan I in the cell wall and in solution: A review. Carbohydr Polym 2022;278:118909.**

**[37] Wachananawat B, Kuroha T, Takenaka Y, Kajiura H, Naramoto S, Yokoyama R, et al. Diversity of Pectin Rhamnogalacturonan I Rhamnosyltransferases in Glycosyltransferase Family 106. Front Plant Sci 2020;11:997.**

**[38] Atmodjo MA, Sakuragi Y, Zhu X, Burrell AJ, Mohanty SS, Atwood JA, 3rd, et al. Galacturonosyltransferase (GAUT)1 and GAUT7 are the core of a plant cell wall pectin biosynthetic homogalacturonan:galacturonosyltransferase complex. Proc Natl Acad Sci U S A 2011;108:20225-30.**

**[39] Ridley BL, O'Neill MA, Mohnen D. Pectins: structure, biosynthesis, and oligogalacturonide-related signaling. Phytochemistry 2001;57:929-67.**

**[40] Philippe F, Pelloux J, Rayon C. Plant pectin acetylesterase structure and function: new insights from bioinformatic analysis. BMC Genomics 2017;18:456.**

**[41] Carpita NC, Gibeaut DM. Structural models of primary cell walls in flowering plants: consistency of molecular structure with the physical properties of the walls during growth. Plant J 1993;3:1-30.**

**[42] Mohnen D. Pectin structure and biosynthesis. Curr Opin Plant Biol 2008;11:266-77.**

**[43] Harholt J, Jensen JK, Sørensen SO, Orfila C, Pauly M, Scheller HV. ARABINAN DEFICIENT 1 is a putative arabinosyltransferase involved in biosynthesis of pectic arabinan in Arabidopsis. Plant Physiol 2006;140:49-58.**

**[44] Redgwell RJ, Curti D, Wang J, Dobruchowska JM, Gerwig GJ, Kamerling JP, et al. Cell wall polysaccharides of Chinese Wolfberry (Lycium barbarum): Part 2. Characterisation of arabinogalactan-proteins. Carbohydrate Polymers 2011;84:1075-83.**

**[45] Statello L, Guo CJ, Chen LL, Huarte M. Gene regulation by long non-coding RNAs and its biological functions. Nat Rev Mol Cell Biol 2021;22:96-118.**

**[46] Zhao L, Wang J, Li Y, Song T, Wu Y, Fang S, et al. NONCODEV6: an updated database dedicated to long non-coding RNA annotation in both animals and plants. Nucleic Acids Res 2021;49:D165-d71.**

**[47] Bailey TL, Johnson J, Grant CE, Noble WS. The MEME Suite. Nucleic Acids Res 2015;43:W39-49.**

**[48] Yin Y, Chen H, Hahn MG, Mohnen D, Xu Y. Evolution and function of the plant cell wall synthesis-related glycosyltransferase family 8. Plant Physiol 2010;153:1729-46.**

**[49] Caffall KH, Pattathil S, Phillips SE, Hahn MG, Mohnen D. Arabidopsis thaliana T-DNA mutants implicate GAUT genes in the biosynthesis of pectin and xylan in cell walls and seed testa. Mol Plant 2009;2:1000-14.**

**[50] Wang L, Wang W, Wang YQ, Liu YY, Wang JX, Zhang XQ, et al. Arabidopsis galacturonosyltransferase (GAUT) 13 and GAUT14 have redundant functions in pollen tube growth. Mol Plant 2013;6:1131-48.**

**[51] Biswal AK, Hao Z, Pattathil S, Yang X, Winkeler K, Collins C, et al. Downregulation of GAUT12 in Populus deltoides by RNA silencing results in reduced recalcitrance, increased growth and reduced xylan and pectin in a woody biofuel feedstock. Biotechnol Biofuels 2015;8:41.**

**[52] de Souza A, Hull PA, Gille S, Pauly M. Identification and functional characterization of the distinct plant pectin esterases PAE8 and PAE9 and their deletion mutants. Planta 2014;240:1123-38.**

**[1] Gao Y, Wei Y, Wang Y, Gao F, Chen Z. Lycium Barbarum: A Traditional Chinese Herb and A Promising Anti-Aging Agent. Aging Dis 2017;8:778-91.**

**[2] Sun C, Chen X, Yang S, Jin C, Ding K, Chen C. LBP1C-2 from Lycium barbarum alleviated age-related bone loss by targeting BMPRIA/BMPRII/Noggin. Carbohydr Polym 2023;310:120725.**

**[3] Potterat O. Goji (Lycium barbarum and L. chinense): Phytochemistry, pharmacology and safety in the perspective of traditional uses and recent popularity. Planta Med 2010;76:7-19.**

**[4] Zhang XJ, Yu HY, Cai YJ, Ke M. Lycium barbarum polysaccharides inhibit proliferation and migration of bladder cancer cell lines BIU87 by suppressing Pi3K/AKT pathway. Oncotarget 2017;8:5936-42.**

**[5] Tang L, Bao S, Du Y, Jiang Z, Wuliji AO, Ren X, et al. Antioxidant effects of Lycium barbarum polysaccharides on photoreceptor degeneration in the light-exposed mouse retina. Biomed Pharmacother 2018;103:829-37.**

**[6] Zhang W, Zhang J, Ding D, Zhang L, Muehlmann LA, Deng SE, et al. Synthesis and antioxidant properties of Lycium barbarum polysaccharides capped selenium nanoparticles using tea extract. Artif Cells Nanomed Biotechnol 2018;46:1463-70.**

**[7] Ma K, Wang X, Feng S, Xia X, Zhang H, Rahaman A, et al. From the perspective of Traditional Chinese Medicine: Treatment of mental disorders in COVID-19 survivors. Biomed Pharmacother 2020;132:110810.**

**[8] Po KK, Leung JW, Chan JN, Fung TK, Sánchez-Vidaña DI, Sin EL, et al. Protective effect of Lycium Barbarum polysaccharides on dextromethorphan-induced mood impairment and neurogenesis suppression. Brain Res Bull 2017;134:10-7.**

**[9] Fang S, Dong L, Liu L, Guo J, Zhao L, Zhang J, et al. HERB: a high-throughput experiment- and reference-guided database of traditional Chinese medicine. Nucleic Acids Res 2021;49:D1197-d206.**

**[10] Zhang S, He F, Chen X, Ding K. Isolation and structural characterization of a pectin from Lycium ruthenicum Murr and its anti-pancreatic ductal adenocarcinoma cell activity. Carbohydr Polym 2019;223:115104.**

**[11] Cao YL, Li YL, Fan YF, Li Z, Yoshida K, Wang JY, et al. Wolfberry genomes and the evolution of Lycium (Solanaceae). Commun Biol 2021;4:671.**

**[12] Chen J, Liu X, Zhu L, Wang Y. Nuclear genome size estimation and karyotype analysis of Lycium species (Solanaceae). Scientia Horticulturae 2013;151:46-50.**

**[13] Giri MK, Swain S, Gautam JK, Singh S, Singh N, Bhattacharjee L, et al. The Arabidopsis thaliana At4g13040 gene, a unique member of the AP2/EREBP family, is a positive regulator for salicylic acid accumulation and basal defense against bacterial pathogens. J Plant Physiol 2014;171:860-7.**

**[14] Michaels SD, Ditta G, Gustafson-Brown C, Pelaz S, Yanofsky M, Amasino RM. AGL24 acts as a promoter of flowering in Arabidopsis and is positively regulated by vernalization. Plant J 2003;33:867-74.**

**[15] Schauser L, Roussis A, Stiller J, Stougaard J. A plant regulator controlling development of symbiotic root nodules. Nature 1999;402:191-5.**

**[16] Hou X, Zhou J, Liu C, Liu L, Shen L, Yu H. Nuclear factor Y-mediated H3K27me3 demethylation of the SOC1 locus orchestrates flowering responses of Arabidopsis. Nat Commun 2014;5:4601.**

**[17] Ru L, He Y, Zhu Z, Patrick JW, Ruan YL. Integrating Sugar Metabolism With Transport: Elevation of Endogenous Cell Wall Invertase Activity Up-Regulates SlHT2 and SlSWEET12c Expression for Early Fruit Development in Tomato. Front Genet 2020;11:592596.**

**[18] Breia R, Conde A, Badim H, Fortes AM, Gerós H, Granell A. Plant SWEETs: from sugar transport to plant-pathogen interaction and more unexpected physiological roles. Plant Physiol 2021;186:836-52.**

**[19] Chen LQ, Hou BH, Lalonde S, Takanaga H, Hartung ML, Qu XQ, et al. Sugar transporters for intercellular exchange and nutrition of pathogens. Nature 2010;468:527-32.**

**[20] Eom JS, Chen LQ, Sosso D, Julius BT, Lin IW, Qu XQ, et al. SWEETs, transporters for intracellular and intercellular sugar translocation. Curr Opin Plant Biol 2015;25:53-62.**

**[21] Li Y, Feng S, Ma S, Sui X, Zhang Z. Spatiotemporal Expression and Substrate Specificity Analysis of the Cucumber SWEET Gene Family. Front Plant Sci 2017;8:1855.**

**[22] Patil G, Valliyodan B, Deshmukh R, Prince S, Nicander B, Zhao M, et al. Soybean (Glycine max) SWEET gene family: insights through comparative genomics, transcriptome profiling and whole genome re-sequence analysis. BMC Genomics 2015;16:520.**

**[23] Zhang W, Wang S, Yu F, Tang J, Shan X, Bao K, et al. Genome-wide characterization and expression profiling of SWEET genes in cabbage (Brassica oleracea var. capitata L.) reveal their roles in chilling and clubroot disease responses. BMC Genomics 2019;20:93.**

**[25] Manck-Götzenberger J, Requena N. Arbuscular mycorrhiza Symbiosis Induces a Major Transcriptional Reprogramming of the Potato SWEET Sugar Transporter Family. Front Plant Sci 2016;7:487.**

**[26] Ko HY, Ho LH, Neuhaus HE, Guo WJ. Transporter SlSWEET15 unloads sucrose from phloem and seed coat for fruit and seed development in tomato. Plant Physiol 2021;187:2230-45.**

**[27] Zhang X, Feng C, Wang M, Li T, Liu X, Jiang J. Plasma membrane-localized SlSWEET7a and SlSWEET14 regulate sugar transport and storage in tomato fruits. Hortic Res 2021;8:186.**

**[28] Pan L, Guo Q, Chai S, Cheng Y, Ruan M, Ye Q, et al. Evolutionary Conservation and Expression Patterns of Neutral/Alkaline Invertases in Solanum. Biomolecules 2019;9.**

**[29] Veillet F, Gaillard C, Coutos-Thévenot P, La Camera S. Targeting the AtCWIN1 Gene to Explore the Role of Invertases in Sucrose Transport in Roots and during Botrytis cinerea Infection. Front Plant Sci 2016;7:1899.**

**[31] Jin Y, Ni DA, Ruan YL. Posttranslational elevation of cell wall invertase activity by silencing its inhibitor in tomato delays leaf senescence and increases seed weight and fruit hexose level. Plant Cell 2009;21:2072-89.**

**[33] Lombard V, Golaconda Ramulu H, Drula E, Coutinho PM, Henrissat B. The carbohydrate-active enzymes database (CAZy) in 2013. Nucleic Acids Res 2014;42:D490-5.**

**[34] Zhang H, Yohe T, Huang L, Entwistle S, Wu P, Yang Z, et al. dbCAN2: a meta server for automated carbohydrate-active enzyme annotation. Nucleic Acids Res 2018;46:W95-w101.**

**[35] Langfelder P, Horvath S. WGCNA: an R package for weighted correlation network analysis. BMC Bioinformatics 2008;9:559.**

**[36] Kaczmarska A, Pieczywek PM, Cybulska J, Zdunek A. Structure and functionality of Rhamnogalacturonan I in the cell wall and in solution: A review. Carbohydr Polym 2022;278:118909.**

**[37] Wachananawat B, Kuroha T, Takenaka Y, Kajiura H, Naramoto S, Yokoyama R, et al. Diversity of Pectin Rhamnogalacturonan I Rhamnosyltransferases in Glycosyltransferase Family 106. Front Plant Sci 2020;11:997.**

**[38] Atmodjo MA, Sakuragi Y, Zhu X, Burrell AJ, Mohanty SS, Atwood JA, 3rd, et al. Galacturonosyltransferase (GAUT)1 and GAUT7 are the core of a plant cell wall pectin biosynthetic homogalacturonan:galacturonosyltransferase complex. Proc Natl Acad Sci U S A 2011;108:20225-30.**

**[39] Ridley BL, O'Neill MA, Mohnen D. Pectins: structure, biosynthesis, and oligogalacturonide-related signaling. Phytochemistry 2001;57:929-67.**

**[40] Philippe F, Pelloux J, Rayon C. Plant pectin acetylesterase structure and function: new insights from bioinformatic analysis. BMC Genomics 2017;18:456.**

**[41] Carpita NC, Gibeaut DM. Structural models of primary cell walls in flowering plants: consistency of molecular structure with the physical properties of the walls during growth. Plant J 1993;3:1-30.**

**[42] Mohnen D. Pectin structure and biosynthesis. Curr Opin Plant Biol 2008;11:266-77.**

**[43] Harholt J, Jensen JK, Sørensen SO, Orfila C, Pauly M, Scheller HV. ARABINAN DEFICIENT 1 is a putative arabinosyltransferase involved in biosynthesis of pectic arabinan in Arabidopsis. Plant Physiol 2006;140:49-58.**

**[44] Redgwell RJ, Curti D, Wang J, Dobruchowska JM, Gerwig GJ, Kamerling JP, et al. Cell wall polysaccharides of Chinese Wolfberry (Lycium barbarum): Part 2. Characterisation of arabinogalactan-proteins. Carbohydrate Polymers 2011;84:1075-83.**

**[45] Statello L, Guo CJ, Chen LL, Huarte M. Gene regulation by long non-coding RNAs and its biological functions. Nat Rev Mol Cell Biol 2021;22:96-118.**

**[46] Zhao L, Wang J, Li Y, Song T, Wu Y, Fang S, et al. NONCODEV6: an updated database dedicated to long non-coding RNA annotation in both animals and plants. Nucleic Acids Res 2021;49:D165-d71.**

**[47] Bailey TL, Johnson J, Grant CE, Noble WS. The MEME Suite. Nucleic Acids Res 2015;43:W39-49.**

**[48] Yin Y, Chen H, Hahn MG, Mohnen D, Xu Y. Evolution and function of the plant cell wall synthesis-related glycosyltransferase family 8. Plant Physiol 2010;153:1729-46.**

**[49] Caffall KH, Pattathil S, Phillips SE, Hahn MG, Mohnen D. Arabidopsis thaliana T-DNA mutants implicate GAUT genes in the biosynthesis of pectin and xylan in cell walls and seed testa. Mol Plant 2009;2:1000-14.**

**[50] Wang L, Wang W, Wang YQ, Liu YY, Wang JX, Zhang XQ, et al. Arabidopsis galacturonosyltransferase (GAUT) 13 and GAUT14 have redundant functions in pollen tube growth. Mol Plant 2013;6:1131-48.**

**[51] Biswal AK, Hao Z, Pattathil S, Yang X, Winkeler K, Collins C, et al. Downregulation of GAUT12 in Populus deltoides by RNA silencing results in reduced recalcitrance, increased growth and reduced xylan and pectin in a woody biofuel feedstock. Biotechnol Biofuels 2015;8:41.**

**[52] de Souza A, Hull PA, Gille S, Pauly M. Identification and functional characterization of the distinct plant pectin esterases PAE8 and PAE9 and their deletion mutants. Planta 2014;240:1123-38.**

**[1] Gao Y, Wei Y, Wang Y, Gao F, Chen Z. Lycium Barbarum: A Traditional Chinese Herb and A Promising Anti-Aging Agent. Aging Dis 2017;8:778-91.**

**[2] Sun C, Chen X, Yang S, Jin C, Ding K, Chen C. LBP1C-2 from Lycium barbarum alleviated age-related bone loss by targeting BMPRIA/BMPRII/Noggin. Carbohydr Polym 2023;310:120725.**

**[3] Potterat O. Goji (Lycium barbarum and L. chinense): Phytochemistry, pharmacology and safety in the perspective of traditional uses and recent popularity. Planta Med 2010;76:7-19.**

**[4] Zhang XJ, Yu HY, Cai YJ, Ke M. Lycium barbarum polysaccharides inhibit proliferation and migration of bladder cancer cell lines BIU87 by suppressing Pi3K/AKT pathway. Oncotarget 2017;8:5936-42.**

**[5] Tang L, Bao S, Du Y, Jiang Z, Wuliji AO, Ren X, et al. Antioxidant effects of Lycium barbarum polysaccharides on photoreceptor degeneration in the light-exposed mouse retina. Biomed Pharmacother 2018;103:829-37.**

**[6] Zhang W, Zhang J, Ding D, Zhang L, Muehlmann LA, Deng SE, et al. Synthesis and antioxidant properties of Lycium barbarum polysaccharides capped selenium nanoparticles using tea extract. Artif Cells Nanomed Biotechnol 2018;46:1463-70.**

**[7] Ma K, Wang X, Feng S, Xia X, Zhang H, Rahaman A, et al. From the perspective of Traditional Chinese Medicine: Treatment of mental disorders in COVID-19 survivors. Biomed Pharmacother 2020;132:110810.**

**[8] Po KK, Leung JW, Chan JN, Fung TK, Sánchez-Vidaña DI, Sin EL, et al. Protective effect of Lycium Barbarum polysaccharides on dextromethorphan-induced mood impairment and neurogenesis suppression. Brain Res Bull 2017;134:10-7.**

**[9] Fang S, Dong L, Liu L, Guo J, Zhao L, Zhang J, et al. HERB: a high-throughput experiment- and reference-guided database of traditional Chinese medicine. Nucleic Acids Res 2021;49:D1197-d206.**

**[10] Zhang S, He F, Chen X, Ding K. Isolation and structural characterization of a pectin from Lycium ruthenicum Murr and its anti-pancreatic ductal adenocarcinoma cell activity. Carbohydr Polym 2019;223:115104.**

**[11] Cao YL, Li YL, Fan YF, Li Z, Yoshida K, Wang JY, et al. Wolfberry genomes and the evolution of Lycium (Solanaceae). Commun Biol 2021;4:671.**

**[12] Chen J, Liu X, Zhu L, Wang Y. Nuclear genome size estimation and karyotype analysis of Lycium species (Solanaceae). Scientia Horticulturae 2013;151:46-50.**

**[14] Michaels SD, Ditta G, Gustafson-Brown C, Pelaz S, Yanofsky M, Amasino RM. AGL24 acts as a promoter of flowering in Arabidopsis and is positively regulated by vernalization. Plant J 2003;33:867-74.**

**[15] Schauser L, Roussis A, Stiller J, Stougaard J. A plant regulator controlling development of symbiotic root nodules. Nature 1999;402:191-5.**

**[16] Hou X, Zhou J, Liu C, Liu L, Shen L, Yu H. Nuclear factor Y-mediated H3K27me3 demethylation of the SOC1 locus orchestrates flowering responses of Arabidopsis. Nat Commun 2014;5:4601.**

**[17] Ru L, He Y, Zhu Z, Patrick JW, Ruan YL. Integrating Sugar Metabolism With Transport: Elevation of Endogenous Cell Wall Invertase Activity Up-Regulates SlHT2 and SlSWEET12c Expression for Early Fruit Development in Tomato. Front Genet 2020;11:592596.**

**[18] Breia R, Conde A, Badim H, Fortes AM, Gerós H, Granell A. Plant SWEETs: from sugar transport to plant-pathogen interaction and more unexpected physiological roles. Plant Physiol 2021;186:836-52.**

**[19] Chen LQ, Hou BH, Lalonde S, Takanaga H, Hartung ML, Qu XQ, et al. Sugar transporters for intercellular exchange and nutrition of pathogens. Nature 2010;468:527-32.**

**[20] Eom JS, Chen LQ, Sosso D, Julius BT, Lin IW, Qu XQ, et al. SWEETs, transporters for intracellular and intercellular sugar translocation. Curr Opin Plant Biol 2015;25:53-62.**

**[21] Li Y, Feng S, Ma S, Sui X, Zhang Z. Spatiotemporal Expression and Substrate Specificity Analysis of the Cucumber SWEET Gene Family. Front Plant Sci 2017;8:1855.**

**[22] Patil G, Valliyodan B, Deshmukh R, Prince S, Nicander B, Zhao M, et al. Soybean (Glycine max) SWEET gene family: insights through comparative genomics, transcriptome profiling and whole genome re-sequence analysis. BMC Genomics 2015;16:520.**

**[23] Zhang W, Wang S, Yu F, Tang J, Shan X, Bao K, et al. Genome-wide characterization and expression profiling of SWEET genes in cabbage (Brassica oleracea var. capitata L.) reveal their roles in chilling and clubroot disease responses. BMC Genomics 2019;20:93.**

**[24] Feng CY, Han JX, Han XX, Jiang J. Genome-wide identification, phylogeny, and expression analysis of the SWEET gene family in tomato. Gene 2015;573:261-72.**

**[25] Manck-Götzenberger J, Requena N. Arbuscular mycorrhiza Symbiosis Induces a Major Transcriptional Reprogramming of the Potato SWEET Sugar Transporter Family. Front Plant Sci 2016;7:487.**

**[26] Ko HY, Ho LH, Neuhaus HE, Guo WJ. Transporter SlSWEET15 unloads sucrose from phloem and seed coat for fruit and seed development in tomato. Plant Physiol 2021;187:2230-45.**

**[27] Zhang X, Feng C, Wang M, Li T, Liu X, Jiang J. Plasma membrane-localized SlSWEET7a and SlSWEET14 regulate sugar transport and storage in tomato fruits. Hortic Res 2021;8:186.**

**[28] Pan L, Guo Q, Chai S, Cheng Y, Ruan M, Ye Q, et al. Evolutionary Conservation and Expression Patterns of Neutral/Alkaline Invertases in Solanum. Biomolecules 2019;9.**

**[29] Veillet F, Gaillard C, Coutos-Thévenot P, La Camera S. Targeting the AtCWIN1 Gene to Explore the Role of Invertases in Sucrose Transport in Roots and during Botrytis cinerea Infection. Front Plant Sci 2016;7:1899.**

**[30] Wan H, Wu L, Yang Y, Zhou G, Ruan YL. Evolution of Sucrose Metabolism: The Dichotomy of Invertases and Beyond. Trends Plant Sci 2018;23:163-77.**

**[31] Jin Y, Ni DA, Ruan YL. Posttranslational elevation of cell wall invertase activity by silencing its inhibitor in tomato delays leaf senescence and increases seed weight and fruit hexose level. Plant Cell 2009;21:2072-89.**

**[32] Shen S, Ma S, Liu Y, Liao S, Li J, Wu L, et al. Cell Wall Invertase and Sugar Transporters Are Differentially Activated in Tomato Styles and Ovaries During Pollination and Fertilization. Front Plant Sci 2019;10:506.**

**[33] Lombard V, Golaconda Ramulu H, Drula E, Coutinho PM, Henrissat B. The carbohydrate-active enzymes database (CAZy) in 2013. Nucleic Acids Res 2014;42:D490-5.**

**[34] Zhang H, Yohe T, Huang L, Entwistle S, Wu P, Yang Z, et al. dbCAN2: a meta server for automated carbohydrate-active enzyme annotation. Nucleic Acids Res 2018;46:W95-w101.**

**[35] Langfelder P, Horvath S. WGCNA: an R package for weighted correlation network analysis. BMC Bioinformatics 2008;9:559.**

**[36] Kaczmarska A, Pieczywek PM, Cybulska J, Zdunek A. Structure and functionality of Rhamnogalacturonan I in the cell wall and in solution: A review. Carbohydr Polym 2022;278:118909.**

**[37] Wachananawat B, Kuroha T, Takenaka Y, Kajiura H, Naramoto S, Yokoyama R, et al. Diversity of Pectin Rhamnogalacturonan I Rhamnosyltransferases in Glycosyltransferase Family 106. Front Plant Sci 2020;11:997.**

**[38] Atmodjo MA, Sakuragi Y, Zhu X, Burrell AJ, Mohanty SS, Atwood JA, 3rd, et al. Galacturonosyltransferase (GAUT)1 and GAUT7 are the core of a plant cell wall pectin biosynthetic homogalacturonan:galacturonosyltransferase complex. Proc Natl Acad Sci U S A 2011;108:20225-30.**

**[39] Ridley BL, O'Neill MA, Mohnen D. Pectins: structure, biosynthesis, and oligogalacturonide-related signaling. Phytochemistry 2001;57:929-67.**

**[40] Philippe F, Pelloux J, Rayon C. Plant pectin acetylesterase structure and function: new insights from bioinformatic analysis. BMC Genomics 2017;18:456.**

**[41] Carpita NC, Gibeaut DM. Structural models of primary cell walls in flowering plants: consistency of molecular structure with the physical properties of the walls during growth. Plant J 1993;3:1-30.**

**[42] Mohnen D. Pectin structure and biosynthesis. Curr Opin Plant Biol 2008;11:266-77.**

**[43] Harholt J, Jensen JK, Sørensen SO, Orfila C, Pauly M, Scheller HV. ARABINAN DEFICIENT 1 is a putative arabinosyltransferase involved in biosynthesis of pectic arabinan in Arabidopsis. Plant Physiol 2006;140:49-58.**

**[44] Redgwell RJ, Curti D, Wang J, Dobruchowska JM, Gerwig GJ, Kamerling JP, et al. Cell wall polysaccharides of Chinese Wolfberry (Lycium barbarum): Part 2. Characterisation of arabinogalactan-proteins. Carbohydrate Polymers 2011;84:1075-83.**

**[45] Statello L, Guo CJ, Chen LL, Huarte M. Gene regulation by long non-coding RNAs and its biological functions. Nat Rev Mol Cell Biol 2021;22:96-118.**

**[46] Zhao L, Wang J, Li Y, Song T, Wu Y, Fang S, et al. NONCODEV6: an updated database dedicated to long non-coding RNA annotation in both animals and plants. Nucleic Acids Res 2021;49:D165-d71.**

**[47] Bailey TL, Johnson J, Grant CE, Noble WS. The MEME Suite. Nucleic Acids Res 2015;43:W39-49.**

**[48] Yin Y, Chen H, Hahn MG, Mohnen D, Xu Y. Evolution and function of the plant cell wall synthesis-related glycosyltransferase family 8. Plant Physiol 2010;153:1729-46.**

**[49] Caffall KH, Pattathil S, Phillips SE, Hahn MG, Mohnen D. Arabidopsis thaliana T-DNA mutants implicate GAUT genes in the biosynthesis of pectin and xylan in cell walls and seed testa. Mol Plant 2009;2:1000-14.**

**[50] Wang L, Wang W, Wang YQ, Liu YY, Wang JX, Zhang XQ, et al. Arabidopsis galacturonosyltransferase (GAUT) 13 and GAUT14 have redundant functions in pollen tube growth. Mol Plant 2013;6:1131-48.**

**[51] Biswal AK, Hao Z, Pattathil S, Yang X, Winkeler K, Collins C, et al. Downregulation of GAUT12 in Populus deltoides by RNA silencing results in reduced recalcitrance, increased growth and reduced xylan and pectin in a woody biofuel feedstock. Biotechnol Biofuels 2015;8:41.**

**[52] de Souza A, Hull PA, Gille S, Pauly M. Identification and functional characterization of the distinct plant pectin esterases PAE8 and PAE9 and their deletion mutants. Planta 2014;240:1123-38.**

**[1] Gao Y, Wei Y, Wang Y, Gao F, Chen Z. Lycium Barbarum: A Traditional Chinese Herb and A Promising Anti-Aging Agent. Aging Dis 2017;8:778-91.**

**[2] Sun C, Chen X, Yang S, Jin C, Ding K, Chen C. LBP1C-2 from Lycium barbarum alleviated age-related bone loss by targeting BMPRIA/BMPRII/Noggin. Carbohydr Polym 2023;310:120725.**

**[3] Potterat O. Goji (Lycium barbarum and L. chinense): Phytochemistry, pharmacology and safety in the perspective of traditional uses and recent popularity. Planta Med 2010;76:7-19.**

**[4] Zhang XJ, Yu HY, Cai YJ, Ke M. Lycium barbarum polysaccharides inhibit proliferation and migration of bladder cancer cell lines BIU87 by suppressing Pi3K/AKT pathway. Oncotarget 2017;8:5936-42.**

**[5] Tang L, Bao S, Du Y, Jiang Z, Wuliji AO, Ren X, et al. Antioxidant effects of Lycium barbarum polysaccharides on photoreceptor degeneration in the light-exposed mouse retina. Biomed Pharmacother 2018;103:829-37.**

**[6] Zhang W, Zhang J, Ding D, Zhang L, Muehlmann LA, Deng SE, et al. Synthesis and antioxidant properties of Lycium barbarum polysaccharides capped selenium nanoparticles using tea extract. Artif Cells Nanomed Biotechnol 2018;46:1463-70.**

**[7] Ma K, Wang X, Feng S, Xia X, Zhang H, Rahaman A, et al. From the perspective of Traditional Chinese Medicine: Treatment of mental disorders in COVID-19 survivors. Biomed Pharmacother 2020;132:110810.**

**[8] Po KK, Leung JW, Chan JN, Fung TK, Sánchez-Vidaña DI, Sin EL, et al. Protective effect of Lycium Barbarum polysaccharides on dextromethorphan-induced mood impairment and neurogenesis suppression. Brain Res Bull 2017;134:10-7.**

**[9] Fang S, Dong L, Liu L, Guo J, Zhao L, Zhang J, et al. HERB: a high-throughput experiment- and reference-guided database of traditional Chinese medicine. Nucleic Acids Res 2021;49:D1197-d206.**

**[10] Zhang S, He F, Chen X, Ding K. Isolation and structural characterization of a pectin from Lycium ruthenicum Murr and its anti-pancreatic ductal adenocarcinoma cell activity. Carbohydr Polym 2019;223:115104.**

**[11] Cao YL, Li YL, Fan YF, Li Z, Yoshida K, Wang JY, et al. Wolfberry genomes and the evolution of Lycium (Solanaceae). Commun Biol 2021;4:671.**

**[12] Chen J, Liu X, Zhu L, Wang Y. Nuclear genome size estimation and karyotype analysis of Lycium species (Solanaceae). Scientia Horticulturae 2013;151:46-50.**

**[13] Giri MK, Swain S, Gautam JK, Singh S, Singh N, Bhattacharjee L, et al. The Arabidopsis thaliana At4g13040 gene, a unique member of the AP2/EREBP family, is a positive regulator for salicylic acid accumulation and basal defense against bacterial pathogens. J Plant Physiol 2014;171:860-7.**

**[14] Michaels SD, Ditta G, Gustafson-Brown C, Pelaz S, Yanofsky M, Amasino RM. AGL24 acts as a promoter of flowering in Arabidopsis and is positively regulated by vernalization. Plant J 2003;33:867-74.**

**[15] Schauser L, Roussis A, Stiller J, Stougaard J. A plant regulator controlling development of symbiotic root nodules. Nature 1999;402:191-5.**

**[16] Hou X, Zhou J, Liu C, Liu L, Shen L, Yu H. Nuclear factor Y-mediated H3K27me3 demethylation of the SOC1 locus orchestrates flowering responses of Arabidopsis. Nat Commun 2014;5:4601.**

**[17] Ru L, He Y, Zhu Z, Patrick JW, Ruan YL. Integrating Sugar Metabolism With Transport: Elevation of Endogenous Cell Wall Invertase Activity Up-Regulates SlHT2 and SlSWEET12c Expression for Early Fruit Development in Tomato. Front Genet 2020;11:592596.**

**[18] Breia R, Conde A, Badim H, Fortes AM, Gerós H, Granell A. Plant SWEETs: from sugar transport to plant-pathogen interaction and more unexpected physiological roles. Plant Physiol 2021;186:836-52.**

**[19] Chen LQ, Hou BH, Lalonde S, Takanaga H, Hartung ML, Qu XQ, et al. Sugar transporters for intercellular exchange and nutrition of pathogens. Nature 2010;468:527-32.**

**[20] Eom JS, Chen LQ, Sosso D, Julius BT, Lin IW, Qu XQ, et al. SWEETs, transporters for intracellular and intercellular sugar translocation. Curr Opin Plant Biol 2015;25:53-62.**

**[21] Li Y, Feng S, Ma S, Sui X, Zhang Z. Spatiotemporal Expression and Substrate Specificity Analysis of the Cucumber SWEET Gene Family. Front Plant Sci 2017;8:1855.**

**[22] Patil G, Valliyodan B, Deshmukh R, Prince S, Nicander B, Zhao M, et al. Soybean (Glycine max) SWEET gene family: insights through comparative genomics, transcriptome profiling and whole genome re-sequence analysis. BMC Genomics 2015;16:520.**

**[23] Zhang W, Wang S, Yu F, Tang J, Shan X, Bao K, et al. Genome-wide characterization and expression profiling of SWEET genes in cabbage (Brassica oleracea var. capitata L.) reveal their roles in chilling and clubroot disease responses. BMC Genomics 2019;20:93.**

**[24] Feng CY, Han JX, Han XX, Jiang J. Genome-wide identification, phylogeny, and expression analysis of the SWEET gene family in tomato. Gene 2015;573:261-72.**

**[25] Manck-Götzenberger J, Requena N. Arbuscular mycorrhiza Symbiosis Induces a Major Transcriptional Reprogramming of the Potato SWEET Sugar Transporter Family. Front Plant Sci 2016;7:487.**

**[26] Ko HY, Ho LH, Neuhaus HE, Guo WJ. Transporter SlSWEET15 unloads sucrose from phloem and seed coat for fruit and seed development in tomato. Plant Physiol 2021;187:2230-45.**

**[27] Zhang X, Feng C, Wang M, Li T, Liu X, Jiang J. Plasma membrane-localized SlSWEET7a and SlSWEET14 regulate sugar transport and storage in tomato fruits. Hortic Res 2021;8:186.**

**[28] Pan L, Guo Q, Chai S, Cheng Y, Ruan M, Ye Q, et al. Evolutionary Conservation and Expression Patterns of Neutral/Alkaline Invertases in Solanum. Biomolecules 2019;9.**

**[29] Veillet F, Gaillard C, Coutos-Thévenot P, La Camera S. Targeting the AtCWIN1 Gene to Explore the Role of Invertases in Sucrose Transport in Roots and during Botrytis cinerea Infection. Front Plant Sci 2016;7:1899.**

**[30] Wan H, Wu L, Yang Y, Zhou G, Ruan YL. Evolution of Sucrose Metabolism: The Dichotomy of Invertases and Beyond. Trends Plant Sci 2018;23:163-77.**

**[31] Jin Y, Ni DA, Ruan YL. Posttranslational elevation of cell wall invertase activity by silencing its inhibitor in tomato delays leaf senescence and increases seed weight and fruit hexose level. Plant Cell 2009;21:2072-89.**

**[32] Shen S, Ma S, Liu Y, Liao S, Li J, Wu L, et al. Cell Wall Invertase and Sugar Transporters Are Differentially Activated in Tomato Styles and Ovaries During Pollination and Fertilization. Front Plant Sci 2019;10:506.**

**[33] Lombard V, Golaconda Ramulu H, Drula E, Coutinho PM, Henrissat B. The carbohydrate-active enzymes database (CAZy) in 2013. Nucleic Acids Res 2014;42:D490-5.**

**[34] Zhang H, Yohe T, Huang L, Entwistle S, Wu P, Yang Z, et al. dbCAN2: a meta server for automated carbohydrate-active enzyme annotation. Nucleic Acids Res 2018;46:W95-w101.**

**[35] Langfelder P, Horvath S. WGCNA: an R package for weighted correlation network analysis. BMC Bioinformatics 2008;9:559.**

**[36] Kaczmarska A, Pieczywek PM, Cybulska J, Zdunek A. Structure and functionality of Rhamnogalacturonan I in the cell wall and in solution: A review. Carbohydr Polym 2022;278:118909.**

**[37] Wachananawat B, Kuroha T, Takenaka Y, Kajiura H, Naramoto S, Yokoyama R, et al. Diversity of Pectin Rhamnogalacturonan I Rhamnosyltransferases in Glycosyltransferase Family 106. Front Plant Sci 2020;11:997.**

**[38] Atmodjo MA, Sakuragi Y, Zhu X, Burrell AJ, Mohanty SS, Atwood JA, 3rd, et al. Galacturonosyltransferase (GAUT)1 and GAUT7 are the core of a plant cell wall pectin biosynthetic homogalacturonan:galacturonosyltransferase complex. Proc Natl Acad Sci U S A 2011;108:20225-30.**

**[39] Ridley BL, O'Neill MA, Mohnen D. Pectins: structure, biosynthesis, and oligogalacturonide-related signaling. Phytochemistry 2001;57:929-67.**

**[40] Philippe F, Pelloux J, Rayon C. Plant pectin acetylesterase structure and function: new insights from bioinformatic analysis. BMC Genomics 2017;18:456.**

**[41] Carpita NC, Gibeaut DM. Structural models of primary cell walls in flowering plants: consistency of molecular structure with the physical properties of the walls during growth. Plant J 1993;3:1-30.**

**[42] Mohnen D. Pectin structure and biosynthesis. Curr Opin Plant Biol 2008;11:266-77.**

**[43] Harholt J, Jensen JK, Sørensen SO, Orfila C, Pauly M, Scheller HV. ARABINAN DEFICIENT 1 is a putative arabinosyltransferase involved in biosynthesis of pectic arabinan in Arabidopsis. Plant Physiol 2006;140:49-58.**

**[44] Redgwell RJ, Curti D, Wang J, Dobruchowska JM, Gerwig GJ, Kamerling JP, et al. Cell wall polysaccharides of Chinese Wolfberry (Lycium barbarum): Part 2. Characterisation of arabinogalactan-proteins. Carbohydrate Polymers 2011;84:1075-83.**

**[45] Statello L, Guo CJ, Chen LL, Huarte M. Gene regulation by long non-coding RNAs and its biological functions. Nat Rev Mol Cell Biol 2021;22:96-118.**

**[46] Zhao L, Wang J, Li Y, Song T, Wu Y, Fang S, et al. NONCODEV6: an updated database dedicated to long non-coding RNA annotation in both animals and plants. Nucleic Acids Res 2021;49:D165-d71.**

**[47] Bailey TL, Johnson J, Grant CE, Noble WS. The MEME Suite. Nucleic Acids Res 2015;43:W39-49.**

**[48] Yin Y, Chen H, Hahn MG, Mohnen D, Xu Y. Evolution and function of the plant cell wall synthesis-related glycosyltransferase family 8. Plant Physiol 2010;153:1729-46.**

**[49] Caffall KH, Pattathil S, Phillips SE, Hahn MG, Mohnen D. Arabidopsis thaliana T-DNA mutants implicate GAUT genes in the biosynthesis of pectin and xylan in cell walls and seed testa. Mol Plant 2009;2:1000-14.**

**[51] Biswal AK, Hao Z, Pattathil S, Yang X, Winkeler K, Collins C, et al. Downregulation of GAUT12 in Populus deltoides by RNA silencing results in reduced recalcitrance, increased growth and reduced xylan and pectin in a woody biofuel feedstock. Biotechnol Biofuels 2015;8:41.**

**[52] de Souza A, Hull PA, Gille S, Pauly M. Identification and functional characterization of the distinct plant pectin esterases PAE8 and PAE9 and their deletion mutants. Planta 2014;240:1123-38.**

**[1] Gao Y, Wei Y, Wang Y, Gao F, Chen Z. Lycium Barbarum: A Traditional Chinese Herb and A Promising Anti-Aging Agent. Aging Dis 2017;8:778-91.**

**[2] Sun C, Chen X, Yang S, Jin C, Ding K, Chen C. LBP1C-2 from Lycium barbarum alleviated age-related bone loss by targeting BMPRIA/BMPRII/Noggin. Carbohydr Polym 2023;310:120725.**

**[3] Potterat O. Goji (Lycium barbarum and L. chinense): Phytochemistry, pharmacology and safety in the perspective of traditional uses and recent popularity. Planta Med 2010;76:7-19.**

**[4] Zhang XJ, Yu HY, Cai YJ, Ke M. Lycium barbarum polysaccharides inhibit proliferation and migration of bladder cancer cell lines BIU87 by suppressing Pi3K/AKT pathway. Oncotarget 2017;8:5936-42.**

**[5] Tang L, Bao S, Du Y, Jiang Z, Wuliji AO, Ren X, et al. Antioxidant effects of Lycium barbarum polysaccharides on photoreceptor degeneration in the light-exposed mouse retina. Biomed Pharmacother 2018;103:829-37.**

**[6] Zhang W, Zhang J, Ding D, Zhang L, Muehlmann LA, Deng SE, et al. Synthesis and antioxidant properties of Lycium barbarum polysaccharides capped selenium nanoparticles using tea extract. Artif Cells Nanomed Biotechnol 2018;46:1463-70.**

**[7] Ma K, Wang X, Feng S, Xia X, Zhang H, Rahaman A, et al. From the perspective of Traditional Chinese Medicine: Treatment of mental disorders in COVID-19 survivors. Biomed Pharmacother 2020;132:110810.**

**[8] Po KK, Leung JW, Chan JN, Fung TK, Sánchez-Vidaña DI, Sin EL, et al. Protective effect of Lycium Barbarum polysaccharides on dextromethorphan-induced mood impairment and neurogenesis suppression. Brain Res Bull 2017;134:10-7.**

**[9] Fang S, Dong L, Liu L, Guo J, Zhao L, Zhang J, et al. HERB: a high-throughput experiment- and reference-guided database of traditional Chinese medicine. Nucleic Acids Res 2021;49:D1197-d206.**

**[10] Zhang S, He F, Chen X, Ding K. Isolation and structural characterization of a pectin from Lycium ruthenicum Murr and its anti-pancreatic ductal adenocarcinoma cell activity. Carbohydr Polym 2019;223:115104.**

**[11] Cao YL, Li YL, Fan YF, Li Z, Yoshida K, Wang JY, et al. Wolfberry genomes and the evolution of Lycium (Solanaceae). Commun Biol 2021;4:671.**

**[12] Chen J, Liu X, Zhu L, Wang Y. Nuclear genome size estimation and karyotype analysis of Lycium species (Solanaceae). Scientia Horticulturae 2013;151:46-50.**

**[13] Giri MK, Swain S, Gautam JK, Singh S, Singh N, Bhattacharjee L, et al. The Arabidopsis thaliana At4g13040 gene, a unique member of the AP2/EREBP family, is a positive regulator for salicylic acid accumulation and basal defense against bacterial pathogens. J Plant Physiol 2014;171:860-7.**

**[14] Michaels SD, Ditta G, Gustafson-Brown C, Pelaz S, Yanofsky M, Amasino RM. AGL24 acts as a promoter of flowering in Arabidopsis and is positively regulated by vernalization. Plant J 2003;33:867-74.**

**[15] Schauser L, Roussis A, Stiller J, Stougaard J. A plant regulator controlling development of symbiotic root nodules. Nature 1999;402:191-5.**

**[16] Hou X, Zhou J, Liu C, Liu L, Shen L, Yu H. Nuclear factor Y-mediated H3K27me3 demethylation of the SOC1 locus orchestrates flowering responses of Arabidopsis. Nat Commun 2014;5:4601.**

**[17] Ru L, He Y, Zhu Z, Patrick JW, Ruan YL. Integrating Sugar Metabolism With Transport: Elevation of Endogenous Cell Wall Invertase Activity Up-Regulates SlHT2 and SlSWEET12c Expression for Early Fruit Development in Tomato. Front Genet 2020;11:592596.**

**[18] Breia R, Conde A, Badim H, Fortes AM, Gerós H, Granell A. Plant SWEETs: from sugar transport to plant-pathogen interaction and more unexpected physiological roles. Plant Physiol 2021;186:836-52.**

**[19] Chen LQ, Hou BH, Lalonde S, Takanaga H, Hartung ML, Qu XQ, et al. Sugar transporters for intercellular exchange and nutrition of pathogens. Nature 2010;468:527-32.**

**[20] Eom JS, Chen LQ, Sosso D, Julius BT, Lin IW, Qu XQ, et al. SWEETs, transporters for intracellular and intercellular sugar translocation. Curr Opin Plant Biol 2015;25:53-62.**

**[21] Li Y, Feng S, Ma S, Sui X, Zhang Z. Spatiotemporal Expression and Substrate Specificity Analysis of the Cucumber SWEET Gene Family. Front Plant Sci 2017;8:1855.**

**[22] Patil G, Valliyodan B, Deshmukh R, Prince S, Nicander B, Zhao M, et al. Soybean (Glycine max) SWEET gene family: insights through comparative genomics, transcriptome profiling and whole genome re-sequence analysis. BMC Genomics 2015;16:520.**

**[24] Feng CY, Han JX, Han XX, Jiang J. Genome-wide identification, phylogeny, and expression analysis of the SWEET gene family in tomato. Gene 2015;573:261-72.**

**[25] Manck-Götzenberger J, Requena N. Arbuscular mycorrhiza Symbiosis Induces a Major Transcriptional Reprogramming of the Potato SWEET Sugar Transporter Family. Front Plant Sci 2016;7:487.**

**[26] Ko HY, Ho LH, Neuhaus HE, Guo WJ. Transporter SlSWEET15 unloads sucrose from phloem and seed coat for fruit and seed development in tomato. Plant Physiol 2021;187:2230-45.**

**[27] Zhang X, Feng C, Wang M, Li T, Liu X, Jiang J. Plasma membrane-localized SlSWEET7a and SlSWEET14 regulate sugar transport and storage in tomato fruits. Hortic Res 2021;8:186.**

**[28] Pan L, Guo Q, Chai S, Cheng Y, Ruan M, Ye Q, et al. Evolutionary Conservation and Expression Patterns of Neutral/Alkaline Invertases in Solanum. Biomolecules 2019;9.**

**[29] Veillet F, Gaillard C, Coutos-Thévenot P, La Camera S. Targeting the AtCWIN1 Gene to Explore the Role of Invertases in Sucrose Transport in Roots and during Botrytis cinerea Infection. Front Plant Sci 2016;7:1899.**

**[30] Wan H, Wu L, Yang Y, Zhou G, Ruan YL. Evolution of Sucrose Metabolism: The Dichotomy of Invertases and Beyond. Trends Plant Sci 2018;23:163-77.**

**[31] Jin Y, Ni DA, Ruan YL. Posttranslational elevation of cell wall invertase activity by silencing its inhibitor in tomato delays leaf senescence and increases seed weight and fruit hexose level. Plant Cell 2009;21:2072-89.**

**[32] Shen S, Ma S, Liu Y, Liao S, Li J, Wu L, et al. Cell Wall Invertase and Sugar Transporters Are Differentially Activated in Tomato Styles and Ovaries During Pollination and Fertilization. Front Plant Sci 2019;10:506.**

**[33] Lombard V, Golaconda Ramulu H, Drula E, Coutinho PM, Henrissat B. The carbohydrate-active enzymes database (CAZy) in 2013. Nucleic Acids Res 2014;42:D490-5.**

**[34] Zhang H, Yohe T, Huang L, Entwistle S, Wu P, Yang Z, et al. dbCAN2: a meta server for automated carbohydrate-active enzyme annotation. Nucleic Acids Res 2018;46:W95-w101.**

**[35] Langfelder P, Horvath S. WGCNA: an R package for weighted correlation network analysis. BMC Bioinformatics 2008;9:559.**

**[36] Kaczmarska A, Pieczywek PM, Cybulska J, Zdunek A. Structure and functionality of Rhamnogalacturonan I in the cell wall and in solution: A review. Carbohydr Polym 2022;278:118909.**

**[37] Wachananawat B, Kuroha T, Takenaka Y, Kajiura H, Naramoto S, Yokoyama R, et al. Diversity of Pectin Rhamnogalacturonan I Rhamnosyltransferases in Glycosyltransferase Family 106. Front Plant Sci 2020;11:997.**

**[38] Atmodjo MA, Sakuragi Y, Zhu X, Burrell AJ, Mohanty SS, Atwood JA, 3rd, et al. Galacturonosyltransferase (GAUT)1 and GAUT7 are the core of a plant cell wall pectin biosynthetic homogalacturonan:galacturonosyltransferase complex. Proc Natl Acad Sci U S A 2011;108:20225-30.**

**[39] Ridley BL, O'Neill MA, Mohnen D. Pectins: structure, biosynthesis, and oligogalacturonide-related signaling. Phytochemistry 2001;57:929-67.**

**[41] Carpita NC, Gibeaut DM. Structural models of primary cell walls in flowering plants: consistency of molecular structure with the physical properties of the walls during growth. Plant J 1993;3:1-30.**

**[42] Mohnen D. Pectin structure and biosynthesis. Curr Opin Plant Biol 2008;11:266-77.**

**[43] Harholt J, Jensen JK, Sørensen SO, Orfila C, Pauly M, Scheller HV. ARABINAN DEFICIENT 1 is a putative arabinosyltransferase involved in biosynthesis of pectic arabinan in Arabidopsis. Plant Physiol 2006;140:49-58.**

**[44] Redgwell RJ, Curti D, Wang J, Dobruchowska JM, Gerwig GJ, Kamerling JP, et al. Cell wall polysaccharides of Chinese Wolfberry (Lycium barbarum): Part 2. Characterisation of arabinogalactan-proteins. Carbohydrate Polymers 2011;84:1075-83.**

**[45] Statello L, Guo CJ, Chen LL, Huarte M. Gene regulation by long non-coding RNAs and its biological functions. Nat Rev Mol Cell Biol 2021;22:96-118.**

**[46] Zhao L, Wang J, Li Y, Song T, Wu Y, Fang S, et al. NONCODEV6: an updated database dedicated to long non-coding RNA annotation in both animals and plants. Nucleic Acids Res 2021;49:D165-d71.**

**[47] Bailey TL, Johnson J, Grant CE, Noble WS. The MEME Suite. Nucleic Acids Res 2015;43:W39-49.**

**[48] Yin Y, Chen H, Hahn MG, Mohnen D, Xu Y. Evolution and function of the plant cell wall synthesis-related glycosyltransferase family 8. Plant Physiol 2010;153:1729-46.**

**[49] Caffall KH, Pattathil S, Phillips SE, Hahn MG, Mohnen D. Arabidopsis thaliana T-DNA mutants implicate GAUT genes in the biosynthesis of pectin and xylan in cell walls and seed testa. Mol Plant 2009;2:1000-14.**

**[50] Wang L, Wang W, Wang YQ, Liu YY, Wang JX, Zhang XQ, et al. Arabidopsis galacturonosyltransferase (GAUT) 13 and GAUT14 have redundant functions in pollen tube growth. Mol Plant 2013;6:1131-48.**

**[51] Biswal AK, Hao Z, Pattathil S, Yang X, Winkeler K, Collins C, et al. Downregulation of GAUT12 in Populus deltoides by RNA silencing results in reduced recalcitrance, increased growth and reduced xylan and pectin in a woody biofuel feedstock. Biotechnol Biofuels 2015;8:41.**

**[40] Philippe F, Pelloux J, Rayon C. Plant pectin acetylesterase structure and function: new insights from bioinformatic analysis. BMC Genomics 2017;18:456.**

**[52] de Souza A, Hull PA, Gille S, Pauly M. Identification and functional characterization of the distinct plant pectin esterases PAE8 and PAE9 and their deletion mutants. Planta 2014;240:1123-38.**

**[53] Maxwell EG, Colquhoun IJ, Chau HK, Hotchkiss AT, Waldron KW, Morris VJ, et al. Modified sugar beet pectin induces apoptosis of colon cancer cells via an interaction with the neutral sugar side-chains. Carbohydr Polym 2016;136:923-9.**

**[1] Gao Y, Wei Y, Wang Y, Gao F, Chen Z. Lycium Barbarum: A Traditional Chinese Herb and A Promising Anti-Aging Agent. Aging Dis 2017;8:778-91.**

**[2] Sun C, Chen X, Yang S, Jin C, Ding K, Chen C. LBP1C-2 from Lycium barbarum alleviated age-related bone loss by targeting BMPRIA/BMPRII/Noggin. Carbohydr Polym 2023;310:120725.**

**[3] Potterat O. Goji (Lycium barbarum and L. chinense): Phytochemistry, pharmacology and safety in the perspective of traditional uses and recent popularity. Planta Med 2010;76:7-19.**

**[4] Zhang XJ, Yu HY, Cai YJ, Ke M. Lycium barbarum polysaccharides inhibit proliferation and migration of bladder cancer cell lines BIU87 by suppressing Pi3K/AKT pathway. Oncotarget 2017;8:5936-42.**

**[5] Tang L, Bao S, Du Y, Jiang Z, Wuliji AO, Ren X, et al. Antioxidant effects of Lycium barbarum polysaccharides on photoreceptor degeneration in the light-exposed mouse retina. Biomed Pharmacother 2018;103:829-37.**

**[6] Zhang W, Zhang J, Ding D, Zhang L, Muehlmann LA, Deng SE, et al. Synthesis and antioxidant properties of Lycium barbarum polysaccharides capped selenium nanoparticles using tea extract. Artif Cells Nanomed Biotechnol 2018;46:1463-70.**

**[7] Ma K, Wang X, Feng S, Xia X, Zhang H, Rahaman A, et al. From the perspective of Traditional Chinese Medicine: Treatment of mental disorders in COVID-19 survivors. Biomed Pharmacother 2020;132:110810.**

**[8] Po KK, Leung JW, Chan JN, Fung TK, Sánchez-Vidaña DI, Sin EL, et al. Protective effect of Lycium Barbarum polysaccharides on dextromethorphan-induced mood impairment and neurogenesis suppression. Brain Res Bull 2017;134:10-7.**

**[9] Fang S, Dong L, Liu L, Guo J, Zhao L, Zhang J, et al. HERB: a high-throughput experiment- and reference-guided database of traditional Chinese medicine. Nucleic Acids Res 2021;49:D1197-d206.**

**[10] Zhang S, He F, Chen X, Ding K. Isolation and structural characterization of a pectin from Lycium ruthenicum Murr and its anti-pancreatic ductal adenocarcinoma cell activity. Carbohydr Polym 2019;223:115104.**

**[12] Chen J, Liu X, Zhu L, Wang Y. Nuclear genome size estimation and karyotype analysis of Lycium species (Solanaceae). Scientia Horticulturae 2013;151:46-50.**

**[13] Giri MK, Swain S, Gautam JK, Singh S, Singh N, Bhattacharjee L, et al. The Arabidopsis thaliana At4g13040 gene, a unique member of the AP2/EREBP family, is a positive regulator for salicylic acid accumulation and basal defense against bacterial pathogens. J Plant Physiol 2014;171:860-7.**

**[14] Michaels SD, Ditta G, Gustafson-Brown C, Pelaz S, Yanofsky M, Amasino RM. AGL24 acts as a promoter of flowering in Arabidopsis and is positively regulated by vernalization. Plant J 2003;33:867-74.**

**[15] Schauser L, Roussis A, Stiller J, Stougaard J. A plant regulator controlling development of symbiotic root nodules. Nature 1999;402:191-5.**

**[16] Hou X, Zhou J, Liu C, Liu L, Shen L, Yu H. Nuclear factor Y-mediated H3K27me3 demethylation of the SOC1 locus orchestrates flowering responses of Arabidopsis. Nat Commun 2014;5:4601.**

**[17] Ru L, He Y, Zhu Z, Patrick JW, Ruan YL. Integrating Sugar Metabolism With Transport: Elevation of Endogenous Cell Wall Invertase Activity Up-Regulates SlHT2 and SlSWEET12c Expression for Early Fruit Development in Tomato. Front Genet 2020;11:592596.**

**[18] Breia R, Conde A, Badim H, Fortes AM, Gerós H, Granell A. Plant SWEETs: from sugar transport to plant-pathogen interaction and more unexpected physiological roles. Plant Physiol 2021;186:836-52.**

**[19] Chen LQ, Hou BH, Lalonde S, Takanaga H, Hartung ML, Qu XQ, et al. Sugar transporters for intercellular exchange and nutrition of pathogens. Nature 2010;468:527-32.**

**[20] Eom JS, Chen LQ, Sosso D, Julius BT, Lin IW, Qu XQ, et al. SWEETs, transporters for intracellular and intercellular sugar translocation. Curr Opin Plant Biol 2015;25:53-62.**

**[22] Patil G, Valliyodan B, Deshmukh R, Prince S, Nicander B, Zhao M, et al. Soybean (Glycine max) SWEET gene family: insights through comparative genomics, transcriptome profiling and whole genome re-sequence analysis. BMC Genomics 2015;16:520.**

**[23] Zhang W, Wang S, Yu F, Tang J, Shan X, Bao K, et al. Genome-wide characterization and expression profiling of SWEET genes in cabbage (Brassica oleracea var. capitata L.) reveal their roles in chilling and clubroot disease responses. BMC Genomics 2019;20:93.**

**[24] Feng CY, Han JX, Han XX, Jiang J. Genome-wide identification, phylogeny, and expression analysis of the SWEET gene family in tomato. Gene 2015;573:261-72.**

**[26] Ko HY, Ho LH, Neuhaus HE, Guo WJ. Transporter SlSWEET15 unloads sucrose from phloem and seed coat for fruit and seed development in tomato. Plant Physiol 2021;187:2230-45.**

**[27] Zhang X, Feng C, Wang M, Li T, Liu X, Jiang J. Plasma membrane-localized SlSWEET7a and SlSWEET14 regulate sugar transport and storage in tomato fruits. Hortic Res 2021;8:186.**

**[28] Pan L, Guo Q, Chai S, Cheng Y, Ruan M, Ye Q, et al. Evolutionary Conservation and Expression Patterns of Neutral/Alkaline Invertases in Solanum. Biomolecules 2019;9.**

**[29] Veillet F, Gaillard C, Coutos-Thévenot P, La Camera S. Targeting the AtCWIN1 Gene to Explore the Role of Invertases in Sucrose Transport in Roots and during Botrytis cinerea Infection. Front Plant Sci 2016;7:1899.**

**[30] Wan H, Wu L, Yang Y, Zhou G, Ruan YL. Evolution of Sucrose Metabolism: The Dichotomy of Invertases and Beyond. Trends Plant Sci 2018;23:163-77.**

**[31] Jin Y, Ni DA, Ruan YL. Posttranslational elevation of cell wall invertase activity by silencing its inhibitor in tomato delays leaf senescence and increases seed weight and fruit hexose level. Plant Cell 2009;21:2072-89.**

**[32] Shen S, Ma S, Liu Y, Liao S, Li J, Wu L, et al. Cell Wall Invertase and Sugar Transporters Are Differentially Activated in Tomato Styles and Ovaries During Pollination and Fertilization. Front Plant Sci 2019;10:506.**

**[33] Lombard V, Golaconda Ramulu H, Drula E, Coutinho PM, Henrissat B. The carbohydrate-active enzymes database (CAZy) in 2013. Nucleic Acids Res 2014;42:D490-5.**

**[34] Zhang H, Yohe T, Huang L, Entwistle S, Wu P, Yang Z, et al. dbCAN2: a meta server for automated carbohydrate-active enzyme annotation. Nucleic Acids Res 2018;46:W95-w101.**

**[35] Langfelder P, Horvath S. WGCNA: an R package for weighted correlation network analysis. BMC Bioinformatics 2008;9:559.**

**[11] Cao YL, Li YL, Fan YF, Li Z, Yoshida K, Wang JY, et al. Wolfberry genomes and the evolution of Lycium (Solanaceae). Commun Biol 2021;4:671.**

**[36] Kaczmarska A, Pieczywek PM, Cybulska J, Zdunek A. Structure and functionality of Rhamnogalacturonan I in the cell wall and in solution: A review. Carbohydr Polym 2022;278:118909.**

**[37] Wachananawat B, Kuroha T, Takenaka Y, Kajiura H, Naramoto S, Yokoyama R, et al. Diversity of Pectin Rhamnogalacturonan I Rhamnosyltransferases in Glycosyltransferase Family 106. Front Plant Sci 2020;11:997.**

**[38] Atmodjo MA, Sakuragi Y, Zhu X, Burrell AJ, Mohanty SS, Atwood JA, 3rd, et al. Galacturonosyltransferase (GAUT)1 and GAUT7 are the core of a plant cell wall pectin biosynthetic homogalacturonan:galacturonosyltransferase complex. Proc Natl Acad Sci U S A 2011;108:20225-30.**

**[39] Ridley BL, O'Neill MA, Mohnen D. Pectins: structure, biosynthesis, and oligogalacturonide-related signaling. Phytochemistry 2001;57:929-67.**

**[40] Philippe F, Pelloux J, Rayon C. Plant pectin acetylesterase structure and function: new insights from bioinformatic analysis. BMC Genomics 2017;18:456.**

**[41] Carpita NC, Gibeaut DM. Structural models of primary cell walls in flowering plants: consistency of molecular structure with the physical properties of the walls during growth. Plant J 1993;3:1-30.**

**[42] Mohnen D. Pectin structure and biosynthesis. Curr Opin Plant Biol 2008;11:266-77.**

**[43] Harholt J, Jensen JK, Sørensen SO, Orfila C, Pauly M, Scheller HV. ARABINAN DEFICIENT 1 is a putative arabinosyltransferase involved in biosynthesis of pectic arabinan in Arabidopsis. Plant Physiol 2006;140:49-58.**

**[44] Redgwell RJ, Curti D, Wang J, Dobruchowska JM, Gerwig GJ, Kamerling JP, et al. Cell wall polysaccharides of Chinese Wolfberry (Lycium barbarum): Part 2. Characterisation of arabinogalactan-proteins. Carbohydrate Polymers 2011;84:1075-83.**

**[45] Statello L, Guo CJ, Chen LL, Huarte M. Gene regulation by long non-coding RNAs and its biological functions. Nat Rev Mol Cell Biol 2021;22:96-118.**

**[46] Zhao L, Wang J, Li Y, Song T, Wu Y, Fang S, et al. NONCODEV6: an updated database dedicated to long non-coding RNA annotation in both animals and plants. Nucleic Acids Res 2021;49:D165-d71.**

**[47] Bailey TL, Johnson J, Grant CE, Noble WS. The MEME Suite. Nucleic Acids Res 2015;43:W39-49.**

**[48] Yin Y, Chen H, Hahn MG, Mohnen D, Xu Y. Evolution and function of the plant cell wall synthesis-related glycosyltransferase family 8. Plant Physiol 2010;153:1729-46.**

**[49] Caffall KH, Pattathil S, Phillips SE, Hahn MG, Mohnen D. Arabidopsis thaliana T-DNA mutants implicate GAUT genes in the biosynthesis of pectin and xylan in cell walls and seed testa. Mol Plant 2009;2:1000-14.**

**[50] Wang L, Wang W, Wang YQ, Liu YY, Wang JX, Zhang XQ, et al. Arabidopsis galacturonosyltransferase (GAUT) 13 and GAUT14 have redundant functions in pollen tube growth. Mol Plant 2013;6:1131-48.**

**[51] Biswal AK, Hao Z, Pattathil S, Yang X, Winkeler K, Collins C, et al. Downregulation of GAUT12 in Populus deltoides by RNA silencing results in reduced recalcitrance, increased growth and reduced xylan and pectin in a woody biofuel feedstock. Biotechnol Biofuels 2015;8:41.**

**[52] de Souza A, Hull PA, Gille S, Pauly M. Identification and functional characterization of the distinct plant pectin esterases PAE8 and PAE9 and their deletion mutants. Planta 2014;240:1123-38.**

**[53] Maxwell EG, Colquhoun IJ, Chau HK, Hotchkiss AT, Waldron KW, Morris VJ, et al. Modified sugar beet pectin induces apoptosis of colon cancer cells via an interaction with the neutral sugar side-chains. Carbohydr Polym 2016;136:923-9.**

**[54] Zhang W, Xu P, Zhang H. Pectin in cancer therapy: A review. Trends in Food Science & Technology 2015;44:258-71.**

**[1] Gao Y, Wei Y, Wang Y, Gao F, Chen Z. Lycium Barbarum: A Traditional Chinese Herb and A Promising Anti-Aging Agent. Aging Dis 2017;8:778-91.**

**[2] Sun C, Chen X, Yang S, Jin C, Ding K, Chen C. LBP1C-2 from Lycium barbarum alleviated age-related bone loss by targeting BMPRIA/BMPRII/Noggin. Carbohydr Polym 2023;310:120725.**

**[3] Potterat O. Goji (Lycium barbarum and L. chinense): Phytochemistry, pharmacology and safety in the perspective of traditional uses and recent popularity. Planta Med 2010;76:7-19.**

**[4] Zhang XJ, Yu HY, Cai YJ, Ke M. Lycium barbarum polysaccharides inhibit proliferation and migration of bladder cancer cell lines BIU87 by suppressing Pi3K/AKT pathway. Oncotarget 2017;8:5936-42.**

**[5] Tang L, Bao S, Du Y, Jiang Z, Wuliji AO, Ren X, et al. Antioxidant effects of Lycium barbarum polysaccharides on photoreceptor degeneration in the light-exposed mouse retina. Biomed Pharmacother 2018;103:829-37.**

**[6] Zhang W, Zhang J, Ding D, Zhang L, Muehlmann LA, Deng SE, et al. Synthesis and antioxidant properties of Lycium barbarum polysaccharides capped selenium nanoparticles using tea extract. Artif Cells Nanomed Biotechnol 2018;46:1463-70.**

**[7] Ma K, Wang X, Feng S, Xia X, Zhang H, Rahaman A, et al. From the perspective of Traditional Chinese Medicine: Treatment of mental disorders in COVID-19 survivors. Biomed Pharmacother 2020;132:110810.**

**[8] Po KK, Leung JW, Chan JN, Fung TK, Sánchez-Vidaña DI, Sin EL, et al. Protective effect of Lycium Barbarum polysaccharides on dextromethorphan-induced mood impairment and neurogenesis suppression. Brain Res Bull 2017;134:10-7.**

**[9] Fang S, Dong L, Liu L, Guo J, Zhao L, Zhang J, et al. HERB: a high-throughput experiment- and reference-guided database of traditional Chinese medicine. Nucleic Acids Res 2021;49:D1197-d206.**

**[10] Zhang S, He F, Chen X, Ding K. Isolation and structural characterization of a pectin from Lycium ruthenicum Murr and its anti-pancreatic ductal adenocarcinoma cell activity. Carbohydr Polym 2019;223:115104.**

**[12] Chen J, Liu X, Zhu L, Wang Y. Nuclear genome size estimation and karyotype analysis of Lycium species (Solanaceae). Scientia Horticulturae 2013;151:46-50.**

**[13] Giri MK, Swain S, Gautam JK, Singh S, Singh N, Bhattacharjee L, et al. The Arabidopsis thaliana At4g13040 gene, a unique member of the AP2/EREBP family, is a positive regulator for salicylic acid accumulation and basal defense against bacterial pathogens. J Plant Physiol 2014;171:860-7.**

**[14] Michaels SD, Ditta G, Gustafson-Brown C, Pelaz S, Yanofsky M, Amasino RM. AGL24 acts as a promoter of flowering in Arabidopsis and is positively regulated by vernalization. Plant J 2003;33:867-74.**

**[15] Schauser L, Roussis A, Stiller J, Stougaard J. A plant regulator controlling development of symbiotic root nodules. Nature 1999;402:191-5.**

**[16] Hou X, Zhou J, Liu C, Liu L, Shen L, Yu H. Nuclear factor Y-mediated H3K27me3 demethylation of the SOC1 locus orchestrates flowering responses of Arabidopsis. Nat Commun 2014;5:4601.**

**[17] Ru L, He Y, Zhu Z, Patrick JW, Ruan YL. Integrating Sugar Metabolism With Transport: Elevation of Endogenous Cell Wall Invertase Activity Up-Regulates SlHT2 and SlSWEET12c Expression for Early Fruit Development in Tomato. Front Genet 2020;11:592596.**

**[18] Breia R, Conde A, Badim H, Fortes AM, Gerós H, Granell A. Plant SWEETs: from sugar transport to plant-pathogen interaction and more unexpected physiological roles. Plant Physiol 2021;186:836-52.**

**[19] Chen LQ, Hou BH, Lalonde S, Takanaga H, Hartung ML, Qu XQ, et al. Sugar transporters for intercellular exchange and nutrition of pathogens. Nature 2010;468:527-32.**

**[20] Eom JS, Chen LQ, Sosso D, Julius BT, Lin IW, Qu XQ, et al. SWEETs, transporters for intracellular and intercellular sugar translocation. Curr Opin Plant Biol 2015;25:53-62.**

**[21] Li Y, Feng S, Ma S, Sui X, Zhang Z. Spatiotemporal Expression and Substrate Specificity Analysis of the Cucumber SWEET Gene Family. Front Plant Sci 2017;8:1855.**

**[22] Patil G, Valliyodan B, Deshmukh R, Prince S, Nicander B, Zhao M, et al. Soybean (Glycine max) SWEET gene family: insights through comparative genomics, transcriptome profiling and whole genome re-sequence analysis. BMC Genomics 2015;16:520.**

**[23] Zhang W, Wang S, Yu F, Tang J, Shan X, Bao K, et al. Genome-wide characterization and expression profiling of SWEET genes in cabbage (Brassica oleracea var. capitata L.) reveal their roles in chilling and clubroot disease responses. BMC Genomics 2019;20:93.**

**[24] Feng CY, Han JX, Han XX, Jiang J. Genome-wide identification, phylogeny, and expression analysis of the SWEET gene family in tomato. Gene 2015;573:261-72.**

**[26] Ko HY, Ho LH, Neuhaus HE, Guo WJ. Transporter SlSWEET15 unloads sucrose from phloem and seed coat for fruit and seed development in tomato. Plant Physiol 2021;187:2230-45.**

**[27] Zhang X, Feng C, Wang M, Li T, Liu X, Jiang J. Plasma membrane-localized SlSWEET7a and SlSWEET14 regulate sugar transport and storage in tomato fruits. Hortic Res 2021;8:186.**

**[28] Pan L, Guo Q, Chai S, Cheng Y, Ruan M, Ye Q, et al. Evolutionary Conservation and Expression Patterns of Neutral/Alkaline Invertases in Solanum. Biomolecules 2019;9.**

**[29] Veillet F, Gaillard C, Coutos-Thévenot P, La Camera S. Targeting the AtCWIN1 Gene to Explore the Role of Invertases in Sucrose Transport in Roots and during Botrytis cinerea Infection. Front Plant Sci 2016;7:1899.**

**[30] Wan H, Wu L, Yang Y, Zhou G, Ruan YL. Evolution of Sucrose Metabolism: The Dichotomy of Invertases and Beyond. Trends Plant Sci 2018;23:163-77.**

**[31] Jin Y, Ni DA, Ruan YL. Posttranslational elevation of cell wall invertase activity by silencing its inhibitor in tomato delays leaf senescence and increases seed weight and fruit hexose level. Plant Cell 2009;21:2072-89.**

**[32] Shen S, Ma S, Liu Y, Liao S, Li J, Wu L, et al. Cell Wall Invertase and Sugar Transporters Are Differentially Activated in Tomato Styles and Ovaries During Pollination and Fertilization. Front Plant Sci 2019;10:506.**

**[34] Zhang H, Yohe T, Huang L, Entwistle S, Wu P, Yang Z, et al. dbCAN2: a meta server for automated carbohydrate-active enzyme annotation. Nucleic Acids Res 2018;46:W95-w101.**

**[35] Langfelder P, Horvath S. WGCNA: an R package for weighted correlation network analysis. BMC Bioinformatics 2008;9:559.**

**[11] Cao YL, Li YL, Fan YF, Li Z, Yoshida K, Wang JY, et al. Wolfberry genomes and the evolution of Lycium (Solanaceae). Commun Biol 2021;4:671.**

**[36] Kaczmarska A, Pieczywek PM, Cybulska J, Zdunek A. Structure and functionality of Rhamnogalacturonan I in the cell wall and in solution: A review. Carbohydr Polym 2022;278:118909.**

**[37] Wachananawat B, Kuroha T, Takenaka Y, Kajiura H, Naramoto S, Yokoyama R, et al. Diversity of Pectin Rhamnogalacturonan I Rhamnosyltransferases in Glycosyltransferase Family 106. Front Plant Sci 2020;11:997.**

**[38] Atmodjo MA, Sakuragi Y, Zhu X, Burrell AJ, Mohanty SS, Atwood JA, 3rd, et al. Galacturonosyltransferase (GAUT)1 and GAUT7 are the core of a plant cell wall pectin biosynthetic homogalacturonan:galacturonosyltransferase complex. Proc Natl Acad Sci U S A 2011;108:20225-30.**

**[39] Ridley BL, O'Neill MA, Mohnen D. Pectins: structure, biosynthesis, and oligogalacturonide-related signaling. Phytochemistry 2001;57:929-67.**

**[40] Philippe F, Pelloux J, Rayon C. Plant pectin acetylesterase structure and function: new insights from bioinformatic analysis. BMC Genomics 2017;18:456.**

**[41] Carpita NC, Gibeaut DM. Structural models of primary cell walls in flowering plants: consistency of molecular structure with the physical properties of the walls during growth. Plant J 1993;3:1-30.**

**[42] Mohnen D. Pectin structure and biosynthesis. Curr Opin Plant Biol 2008;11:266-77.**

**[43] Harholt J, Jensen JK, Sørensen SO, Orfila C, Pauly M, Scheller HV. ARABINAN DEFICIENT 1 is a putative arabinosyltransferase involved in biosynthesis of pectic arabinan in Arabidopsis. Plant Physiol 2006;140:49-58.**

**[44] Redgwell RJ, Curti D, Wang J, Dobruchowska JM, Gerwig GJ, Kamerling JP, et al. Cell wall polysaccharides of Chinese Wolfberry (Lycium barbarum): Part 2. Characterisation of arabinogalactan-proteins. Carbohydrate Polymers 2011;84:1075-83.**

**[45] Statello L, Guo CJ, Chen LL, Huarte M. Gene regulation by long non-coding RNAs and its biological functions. Nat Rev Mol Cell Biol 2021;22:96-118.**

**[46] Zhao L, Wang J, Li Y, Song T, Wu Y, Fang S, et al. NONCODEV6: an updated database dedicated to long non-coding RNA annotation in both animals and plants. Nucleic Acids Res 2021;49:D165-d71.**

**[47] Bailey TL, Johnson J, Grant CE, Noble WS. The MEME Suite. Nucleic Acids Res 2015;43:W39-49.**

**[48] Yin Y, Chen H, Hahn MG, Mohnen D, Xu Y. Evolution and function of the plant cell wall synthesis-related glycosyltransferase family 8. Plant Physiol 2010;153:1729-46.**

**[49] Caffall KH, Pattathil S, Phillips SE, Hahn MG, Mohnen D. Arabidopsis thaliana T-DNA mutants implicate GAUT genes in the biosynthesis of pectin and xylan in cell walls and seed testa. Mol Plant 2009;2:1000-14.**

**[50] Wang L, Wang W, Wang YQ, Liu YY, Wang JX, Zhang XQ, et al. Arabidopsis galacturonosyltransferase (GAUT) 13 and GAUT14 have redundant functions in pollen tube growth. Mol Plant 2013;6:1131-48.**

**[51] Biswal AK, Hao Z, Pattathil S, Yang X, Winkeler K, Collins C, et al. Downregulation of GAUT12 in Populus deltoides by RNA silencing results in reduced recalcitrance, increased growth and reduced xylan and pectin in a woody biofuel feedstock. Biotechnol Biofuels 2015;8:41.**

**[52] de Souza A, Hull PA, Gille S, Pauly M. Identification and functional characterization of the distinct plant pectin esterases PAE8 and PAE9 and their deletion mutants. Planta 2014;240:1123-38.**

**[53] Maxwell EG, Colquhoun IJ, Chau HK, Hotchkiss AT, Waldron KW, Morris VJ, et al. Modified sugar beet pectin induces apoptosis of colon cancer cells via an interaction with the neutral sugar side-chains. Carbohydr Polym 2016;136:923-9.**

**[55] Ouyang J, Yang M, Gong T, Ou J, Tan Y, Zhang Z, et al. Doxorubicin-loading core-shell pectin nanocell: A novel nanovehicle for anticancer agent delivery with multidrug resistance reversal. PLoS One 2020;15:e0235090.**

**[1] Gao Y, Wei Y, Wang Y, Gao F, Chen Z. Lycium Barbarum: A Traditional Chinese Herb and A Promising Anti-Aging Agent. Aging Dis 2017;8:778-91.**

**[2] Sun C, Chen X, Yang S, Jin C, Ding K, Chen C. LBP1C-2 from Lycium barbarum alleviated age-related bone loss by targeting BMPRIA/BMPRII/Noggin. Carbohydr Polym 2023;310:120725.**

**[3] Potterat O. Goji (Lycium barbarum and L. chinense): Phytochemistry, pharmacology and safety in the perspective of traditional uses and recent popularity. Planta Med 2010;76:7-19.**

**[4] Zhang XJ, Yu HY, Cai YJ, Ke M. Lycium barbarum polysaccharides inhibit proliferation and migration of bladder cancer cell lines BIU87 by suppressing Pi3K/AKT pathway. Oncotarget 2017;8:5936-42.**

**[6] Zhang W, Zhang J, Ding D, Zhang L, Muehlmann LA, Deng SE, et al. Synthesis and antioxidant properties of Lycium barbarum polysaccharides capped selenium nanoparticles using tea extract. Artif Cells Nanomed Biotechnol 2018;46:1463-70.**

**[7] Ma K, Wang X, Feng S, Xia X, Zhang H, Rahaman A, et al. From the perspective of Traditional Chinese Medicine: Treatment of mental disorders in COVID-19 survivors. Biomed Pharmacother 2020;132:110810.**

**[8] Po KK, Leung JW, Chan JN, Fung TK, Sánchez-Vidaña DI, Sin EL, et al. Protective effect of Lycium Barbarum polysaccharides on dextromethorphan-induced mood impairment and neurogenesis suppression. Brain Res Bull 2017;134:10-7.**

**[10] Zhang S, He F, Chen X, Ding K. Isolation and structural characterization of a pectin from Lycium ruthenicum Murr and its anti-pancreatic ductal adenocarcinoma cell activity. Carbohydr Polym 2019;223:115104.**

**[11] Cao YL, Li YL, Fan YF, Li Z, Yoshida K, Wang JY, et al. Wolfberry genomes and the evolution of Lycium (Solanaceae). Commun Biol 2021;4:671.**

**[12] Chen J, Liu X, Zhu L, Wang Y. Nuclear genome size estimation and karyotype analysis of Lycium species (Solanaceae). Scientia Horticulturae 2013;151:46-50.**

**[13] Giri MK, Swain S, Gautam JK, Singh S, Singh N, Bhattacharjee L, et al. The Arabidopsis thaliana At4g13040 gene, a unique member of the AP2/EREBP family, is a positive regulator for salicylic acid accumulation and basal defense against bacterial pathogens. J Plant Physiol 2014;171:860-7.**

**[14] Michaels SD, Ditta G, Gustafson-Brown C, Pelaz S, Yanofsky M, Amasino RM. AGL24 acts as a promoter of flowering in Arabidopsis and is positively regulated by vernalization. Plant J 2003;33:867-74.**

**[15] Schauser L, Roussis A, Stiller J, Stougaard J. A plant regulator controlling development of symbiotic root nodules. Nature 1999;402:191-5.**

**[16] Hou X, Zhou J, Liu C, Liu L, Shen L, Yu H. Nuclear factor Y-mediated H3K27me3 demethylation of the SOC1 locus orchestrates flowering responses of Arabidopsis. Nat Commun 2014;5:4601.**

**[17] Ru L, He Y, Zhu Z, Patrick JW, Ruan YL. Integrating Sugar Metabolism With Transport: Elevation of Endogenous Cell Wall Invertase Activity Up-Regulates SlHT2 and SlSWEET12c Expression for Early Fruit Development in Tomato. Front Genet 2020;11:592596.**

**[18] Breia R, Conde A, Badim H, Fortes AM, Gerós H, Granell A. Plant SWEETs: from sugar transport to plant-pathogen interaction and more unexpected physiological roles. Plant Physiol 2021;186:836-52.**

**[19] Chen LQ, Hou BH, Lalonde S, Takanaga H, Hartung ML, Qu XQ, et al. Sugar transporters for intercellular exchange and nutrition of pathogens. Nature 2010;468:527-32.**

**[20] Eom JS, Chen LQ, Sosso D, Julius BT, Lin IW, Qu XQ, et al. SWEETs, transporters for intracellular and intercellular sugar translocation. Curr Opin Plant Biol 2015;25:53-62.**

**[21] Li Y, Feng S, Ma S, Sui X, Zhang Z. Spatiotemporal Expression and Substrate Specificity Analysis of the Cucumber SWEET Gene Family. Front Plant Sci 2017;8:1855.**

**[22] Patil G, Valliyodan B, Deshmukh R, Prince S, Nicander B, Zhao M, et al. Soybean (Glycine max) SWEET gene family: insights through comparative genomics, transcriptome profiling and whole genome re-sequence analysis. BMC Genomics 2015;16:520.**

**[23] Zhang W, Wang S, Yu F, Tang J, Shan X, Bao K, et al. Genome-wide characterization and expression profiling of SWEET genes in cabbage (Brassica oleracea var. capitata L.) reveal their roles in chilling and clubroot disease responses. BMC Genomics 2019;20:93.**

**[24] Feng CY, Han JX, Han XX, Jiang J. Genome-wide identification, phylogeny, and expression analysis of the SWEET gene family in tomato. Gene 2015;573:261-72.**

**[25] Manck-Götzenberger J, Requena N. Arbuscular mycorrhiza Symbiosis Induces a Major Transcriptional Reprogramming of the Potato SWEET Sugar Transporter Family. Front Plant Sci 2016;7:487.**

**[26] Ko HY, Ho LH, Neuhaus HE, Guo WJ. Transporter SlSWEET15 unloads sucrose from phloem and seed coat for fruit and seed development in tomato. Plant Physiol 2021;187:2230-45.**

**[27] Zhang X, Feng C, Wang M, Li T, Liu X, Jiang J. Plasma membrane-localized SlSWEET7a and SlSWEET14 regulate sugar transport and storage in tomato fruits. Hortic Res 2021;8:186.**

**[28] Pan L, Guo Q, Chai S, Cheng Y, Ruan M, Ye Q, et al. Evolutionary Conservation and Expression Patterns of Neutral/Alkaline Invertases in Solanum. Biomolecules 2019;9.**

**[30] Wan H, Wu L, Yang Y, Zhou G, Ruan YL. Evolution of Sucrose Metabolism: The Dichotomy of Invertases and Beyond. Trends Plant Sci 2018;23:163-77.**

**[31] Jin Y, Ni DA, Ruan YL. Posttranslational elevation of cell wall invertase activity by silencing its inhibitor in tomato delays leaf senescence and increases seed weight and fruit hexose level. Plant Cell 2009;21:2072-89.**

**[32] Shen S, Ma S, Liu Y, Liao S, Li J, Wu L, et al. Cell Wall Invertase and Sugar Transporters Are Differentially Activated in Tomato Styles and Ovaries During Pollination and Fertilization. Front Plant Sci 2019;10:506.**

**[33] Lombard V, Golaconda Ramulu H, Drula E, Coutinho PM, Henrissat B. The carbohydrate-active enzymes database (CAZy) in 2013. Nucleic Acids Res 2014;42:D490-5.**

**[34] Zhang H, Yohe T, Huang L, Entwistle S, Wu P, Yang Z, et al. dbCAN2: a meta server for automated carbohydrate-active enzyme annotation. Nucleic Acids Res 2018;46:W95-w101.**

**[35] Langfelder P, Horvath S. WGCNA: an R package for weighted correlation network analysis. BMC Bioinformatics 2008;9:559.**

**[36] Kaczmarska A, Pieczywek PM, Cybulska J, Zdunek A. Structure and functionality of Rhamnogalacturonan I in the cell wall and in solution: A review. Carbohydr Polym 2022;278:118909.**

**[37] Wachananawat B, Kuroha T, Takenaka Y, Kajiura H, Naramoto S, Yokoyama R, et al. Diversity of Pectin Rhamnogalacturonan I Rhamnosyltransferases in Glycosyltransferase Family 106. Front Plant Sci 2020;11:997.**

**[38] Atmodjo MA, Sakuragi Y, Zhu X, Burrell AJ, Mohanty SS, Atwood JA, 3rd, et al. Galacturonosyltransferase (GAUT)1 and GAUT7 are the core of a plant cell wall pectin biosynthetic homogalacturonan:galacturonosyltransferase complex. Proc Natl Acad Sci U S A 2011;108:20225-30.**

**[39] Ridley BL, O'Neill MA, Mohnen D. Pectins: structure, biosynthesis, and oligogalacturonide-related signaling. Phytochemistry 2001;57:929-67.**

**[40] Philippe F, Pelloux J, Rayon C. Plant pectin acetylesterase structure and function: new insights from bioinformatic analysis. BMC Genomics 2017;18:456.**

**[41] Carpita NC, Gibeaut DM. Structural models of primary cell walls in flowering plants: consistency of molecular structure with the physical properties of the walls during growth. Plant J 1993;3:1-30.**

**[42] Mohnen D. Pectin structure and biosynthesis. Curr Opin Plant Biol 2008;11:266-77.**

**[44] Redgwell RJ, Curti D, Wang J, Dobruchowska JM, Gerwig GJ, Kamerling JP, et al. Cell wall polysaccharides of Chinese Wolfberry (Lycium barbarum): Part 2. Characterisation of arabinogalactan-proteins. Carbohydrate Polymers 2011;84:1075-83.**

**[45] Statello L, Guo CJ, Chen LL, Huarte M. Gene regulation by long non-coding RNAs and its biological functions. Nat Rev Mol Cell Biol 2021;22:96-118.**

**[46] Zhao L, Wang J, Li Y, Song T, Wu Y, Fang S, et al. NONCODEV6: an updated database dedicated to long non-coding RNA annotation in both animals and plants. Nucleic Acids Res 2021;49:D165-d71.**

**[47] Bailey TL, Johnson J, Grant CE, Noble WS. The MEME Suite. Nucleic Acids Res 2015;43:W39-49.**

**[48] Yin Y, Chen H, Hahn MG, Mohnen D, Xu Y. Evolution and function of the plant cell wall synthesis-related glycosyltransferase family 8. Plant Physiol 2010;153:1729-46.**

**[49] Caffall KH, Pattathil S, Phillips SE, Hahn MG, Mohnen D. Arabidopsis thaliana T-DNA mutants implicate GAUT genes in the biosynthesis of pectin and xylan in cell walls and seed testa. Mol Plant 2009;2:1000-14.**

**[50] Wang L, Wang W, Wang YQ, Liu YY, Wang JX, Zhang XQ, et al. Arabidopsis galacturonosyltransferase (GAUT) 13 and GAUT14 have redundant functions in pollen tube growth. Mol Plant 2013;6:1131-48.**

**[51] Biswal AK, Hao Z, Pattathil S, Yang X, Winkeler K, Collins C, et al. Downregulation of GAUT12 in Populus deltoides by RNA silencing results in reduced recalcitrance, increased growth and reduced xylan and pectin in a woody biofuel feedstock. Biotechnol Biofuels 2015;8:41.**

**[52] de Souza A, Hull PA, Gille S, Pauly M. Identification and functional characterization of the distinct plant pectin esterases PAE8 and PAE9 and their deletion mutants. Planta 2014;240:1123-38.**

**[53] Maxwell EG, Colquhoun IJ, Chau HK, Hotchkiss AT, Waldron KW, Morris VJ, et al. Modified sugar beet pectin induces apoptosis of colon cancer cells via an interaction with the neutral sugar side-chains. Carbohydr Polym 2016;136:923-9.**

**[54] Zhang W, Xu P, Zhang H. Pectin in cancer therapy: A review. Trends in Food Science & Technology 2015;44:258-71.**

**[55] Ouyang J, Yang M, Gong T, Ou J, Tan Y, Zhang Z, et al. Doxorubicin-loading core-shell pectin nanocell: A novel nanovehicle for anticancer agent delivery with multidrug resistance reversal. PLoS One 2020;15:e0235090.**

**[56] Sriamornsak P. Application of pectin in oral drug delivery. Expert Opin Drug Deliv 2011;8:1009-23.**

**[1] Gao Y, Wei Y, Wang Y, Gao F, Chen Z. Lycium Barbarum: A Traditional Chinese Herb and A Promising Anti-Aging Agent. Aging Dis 2017;8:778-91.**

**[2] Sun C, Chen X, Yang S, Jin C, Ding K, Chen C. LBP1C-2 from Lycium barbarum alleviated age-related bone loss by targeting BMPRIA/BMPRII/Noggin. Carbohydr Polym 2023;310:120725.**

**[3] Potterat O. Goji (Lycium barbarum and L. chinense): Phytochemistry, pharmacology and safety in the perspective of traditional uses and recent popularity. Planta Med 2010;76:7-19.**

**[4] Zhang XJ, Yu HY, Cai YJ, Ke M. Lycium barbarum polysaccharides inhibit proliferation and migration of bladder cancer cell lines BIU87 by suppressing Pi3K/AKT pathway. Oncotarget 2017;8:5936-42.**

**[5] Tang L, Bao S, Du Y, Jiang Z, Wuliji AO, Ren X, et al. Antioxidant effects of Lycium barbarum polysaccharides on photoreceptor degeneration in the light-exposed mouse retina. Biomed Pharmacother 2018;103:829-37.**

**[6] Zhang W, Zhang J, Ding D, Zhang L, Muehlmann LA, Deng SE, et al. Synthesis and antioxidant properties of Lycium barbarum polysaccharides capped selenium nanoparticles using tea extract. Artif Cells Nanomed Biotechnol 2018;46:1463-70.**
[truncated: 217,506 more chars]
