# Supplementary figures and images for: Enzyme Repertoires and Genomic Insights into Lycium barbarum Pectin Polysaccharide Biosynthesis
Source: Genomics Proteomics Bioinformatics. 2024 Nov 4;22(6):qzae079. doi: 10.1093/gpbjnl/qzae079 (PMC12011363; doi:10.1093/gpbjnl/qzae079)

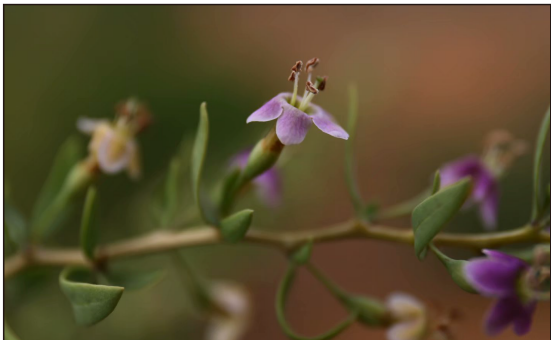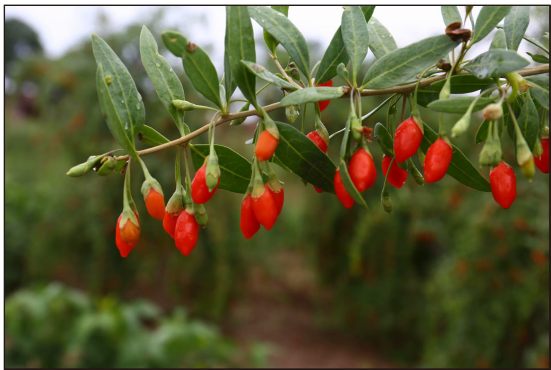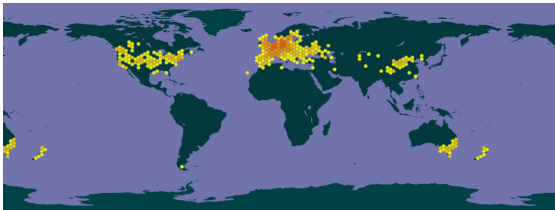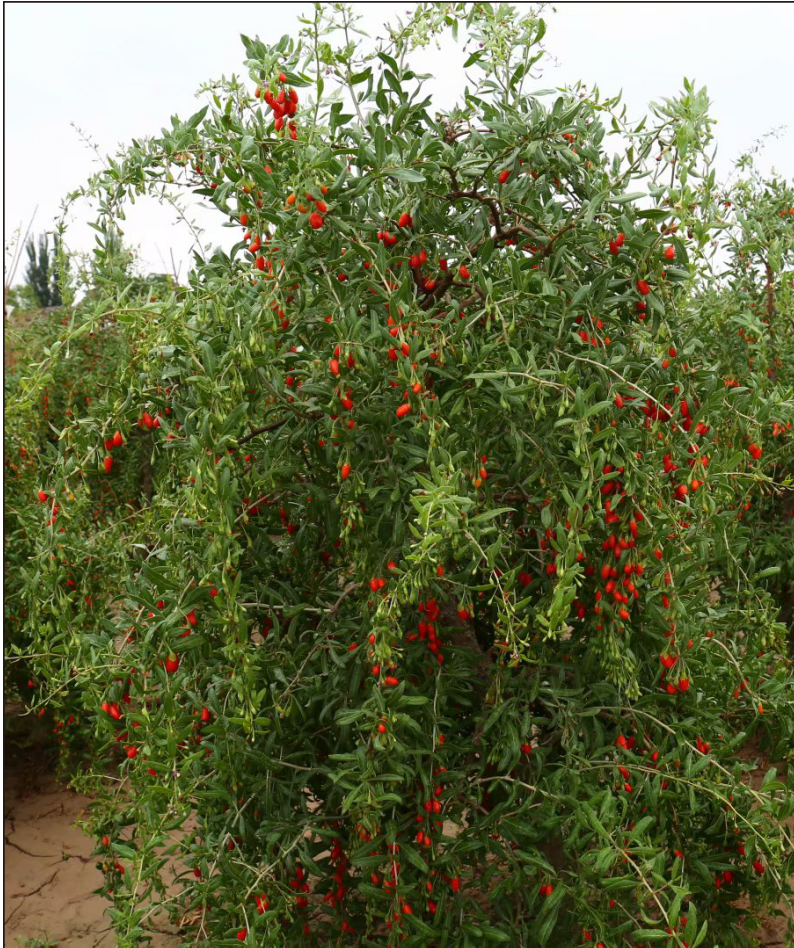

Supplement: qzae079_Supplementary_Data [file qzae079_supplementary_data.zip › Figure S1.pdf]

A

NF-YB

30

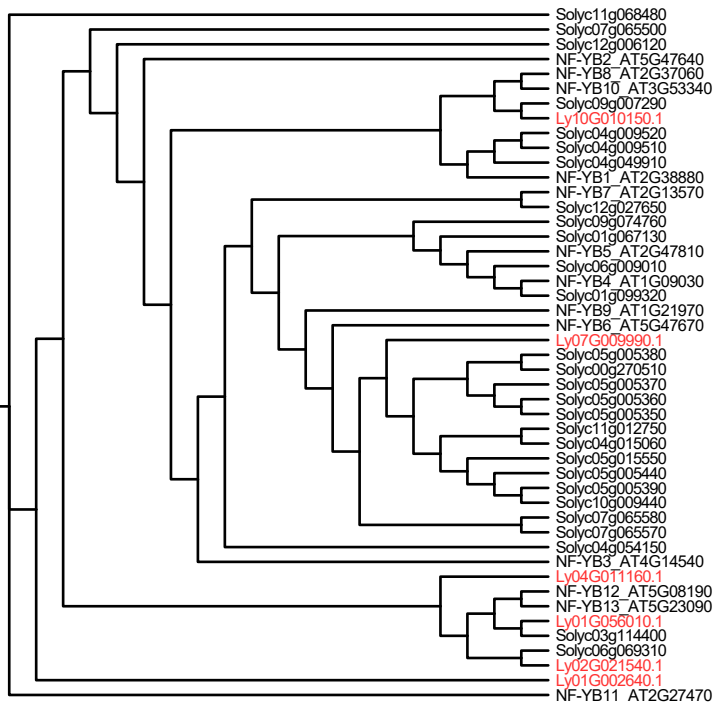

B

NF-YC

20

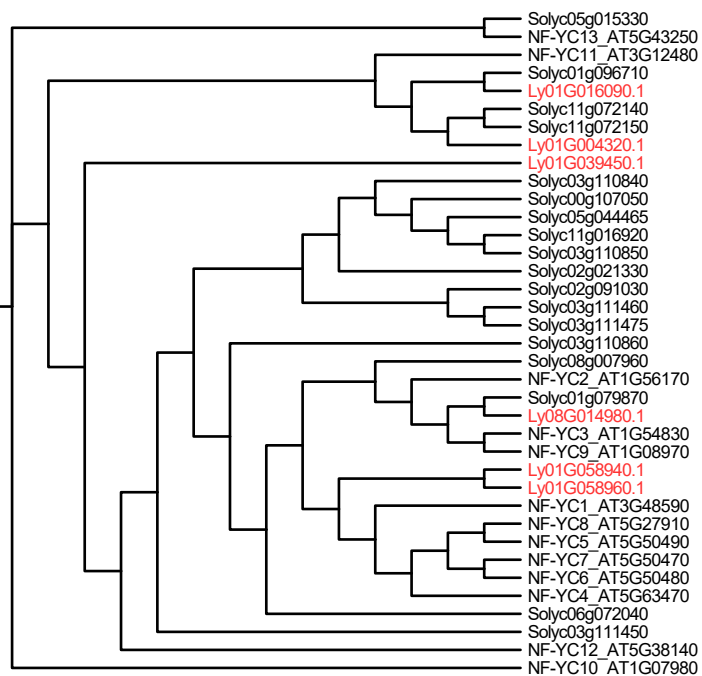

Supplement: qzae079_Supplementary_Data [file qzae079_supplementary_data.zip › Figure S10.pdf]

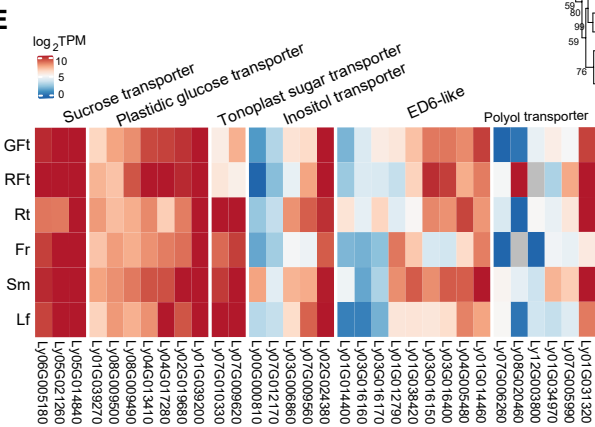

Supplement: qzae079_Supplementary_Data [file qzae079_supplementary_data.zip › Figure S11.pdf]

**A** Tree scale: 1

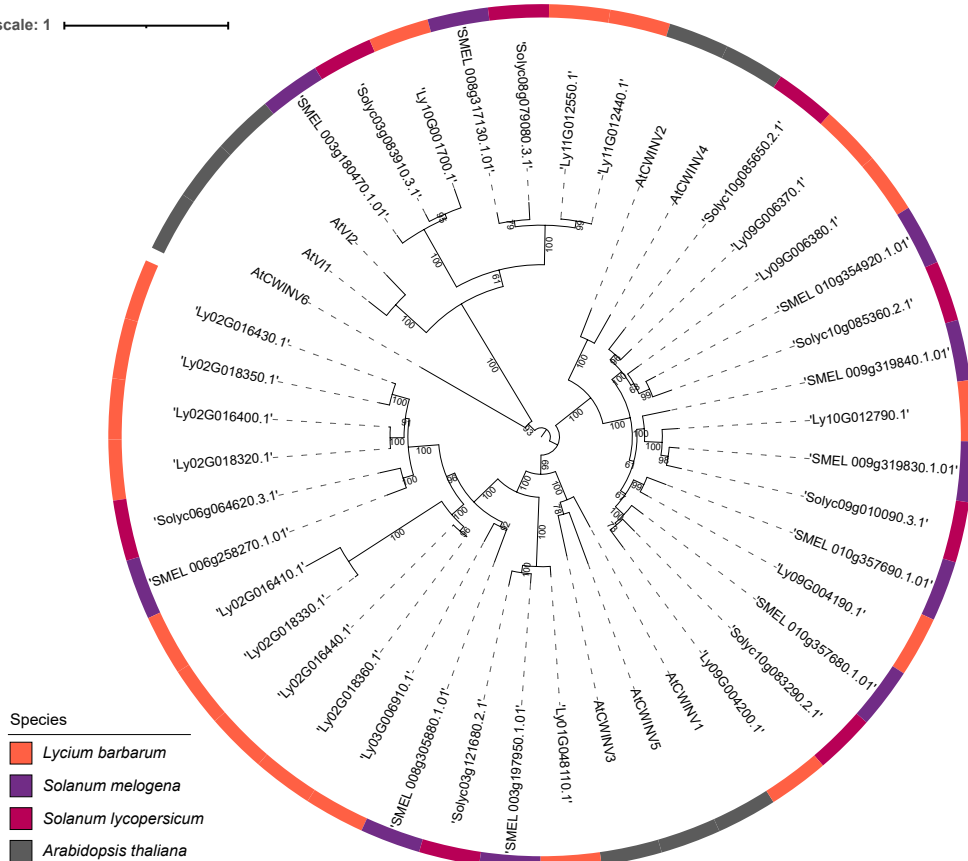

**B**

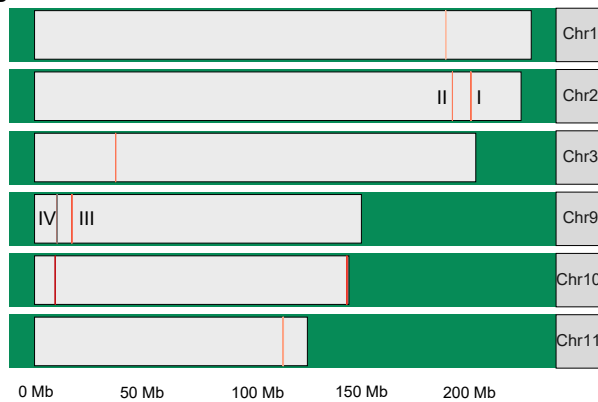

**C**

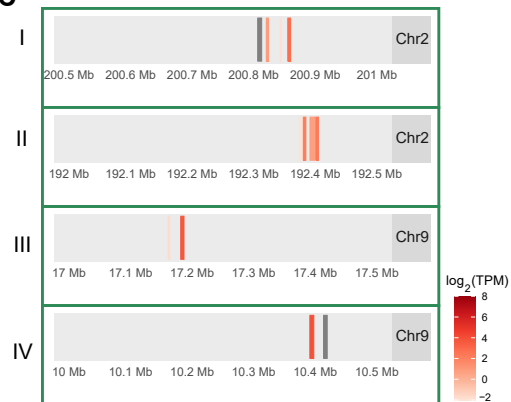

Supplement: qzae079_Supplementary_Data [file qzae079_supplementary_data.zip › Figure S12.pdf]

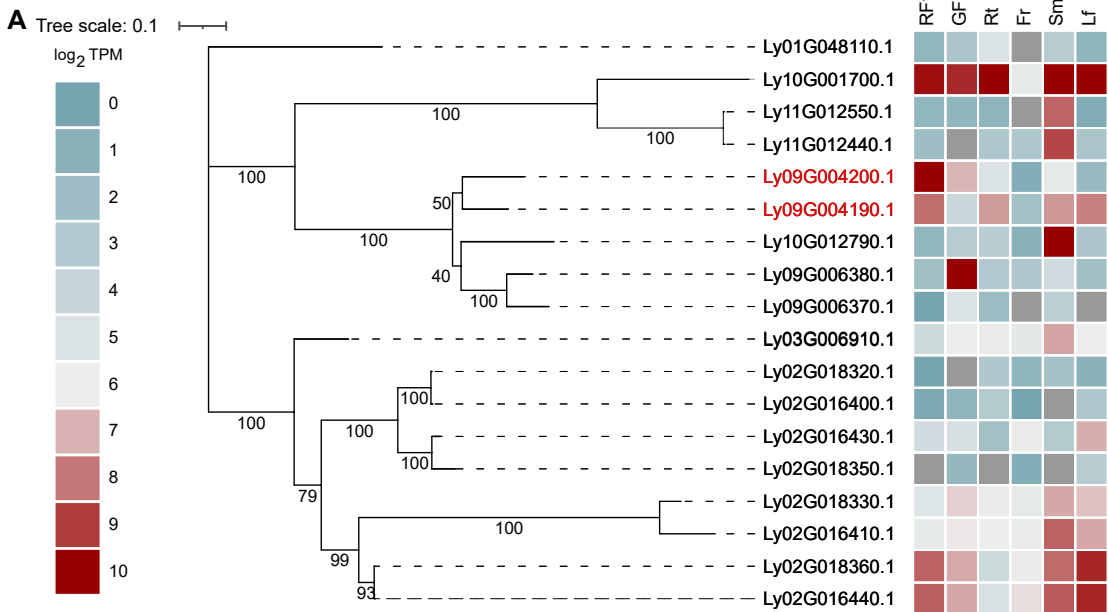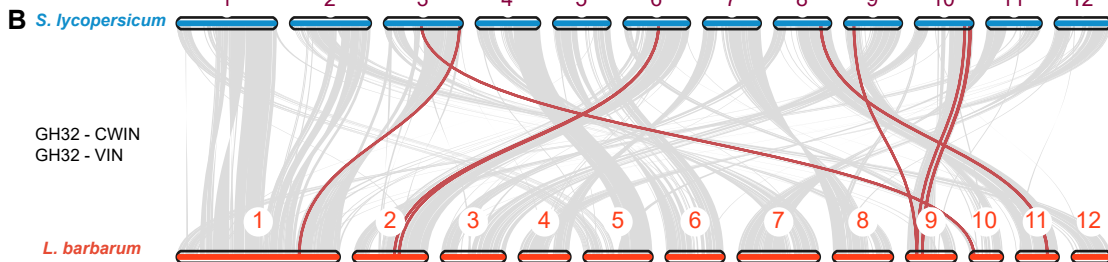

Supplement: qzae079_Supplementary_Data [file qzae079_supplementary_data.zip › Figure S13.pdf]

**A** Tree scale: 1

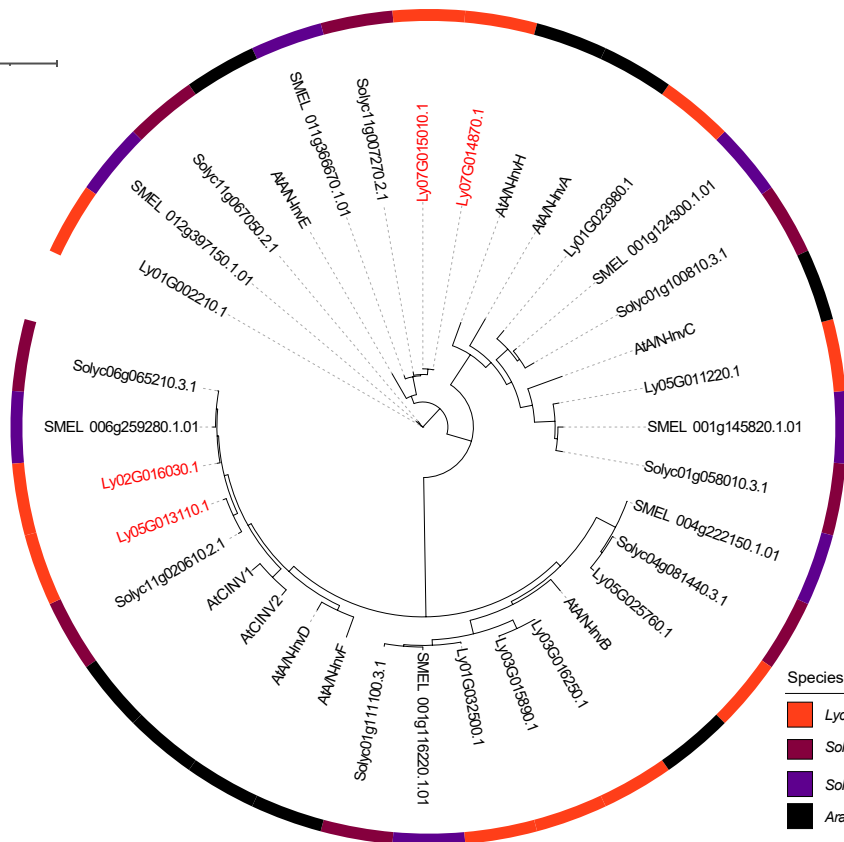

**B** Tree scale: 1

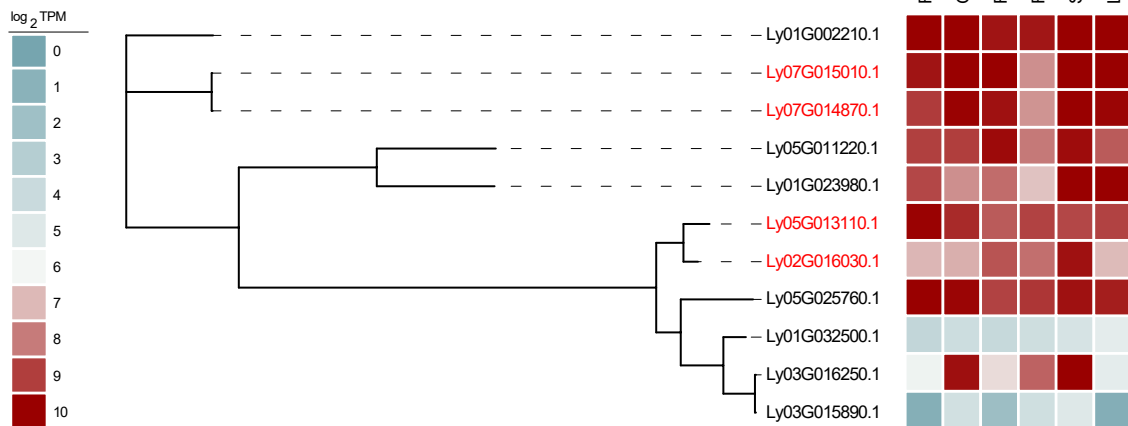

Supplement: qzae079_Supplementary_Data [file qzae079_supplementary_data.zip › Figure S14.pdf]

A

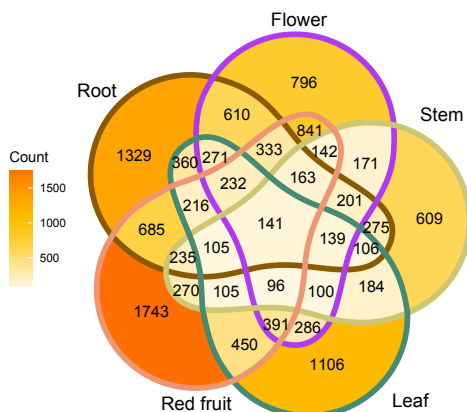

B

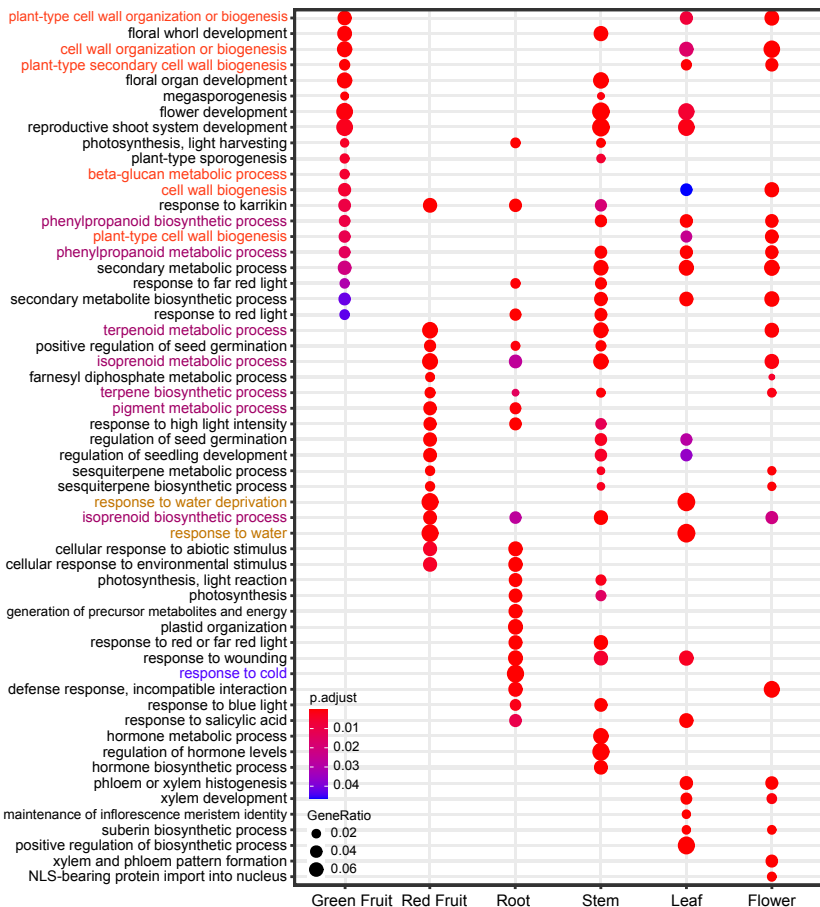

Supplement: qzae079_Supplementary_Data [file qzae079_supplementary_data.zip › Figure S15.pdf]

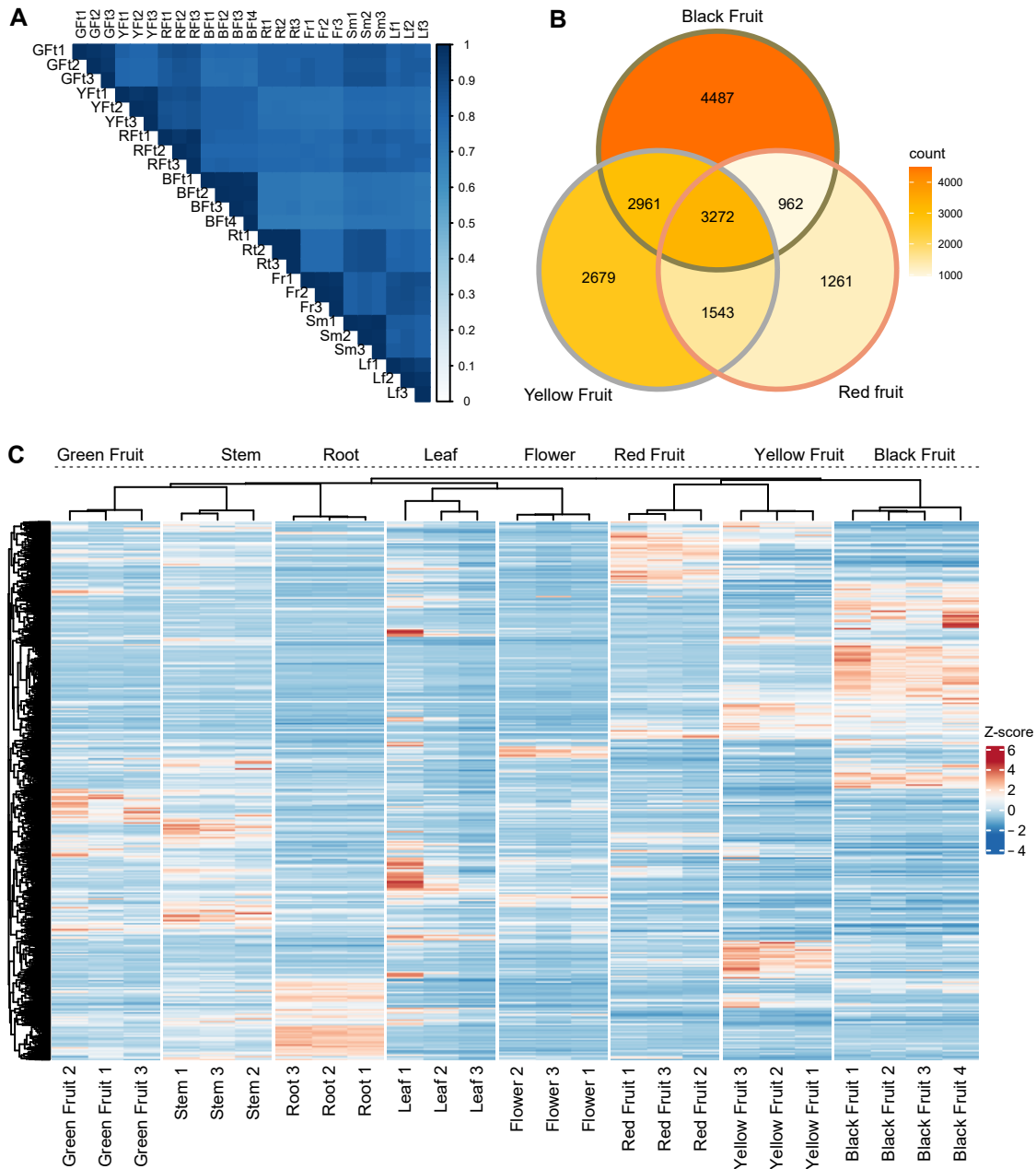

Supplement: qzae079_Supplementary_Data [file qzae079_supplementary_data.zip › Figure S16.pdf]

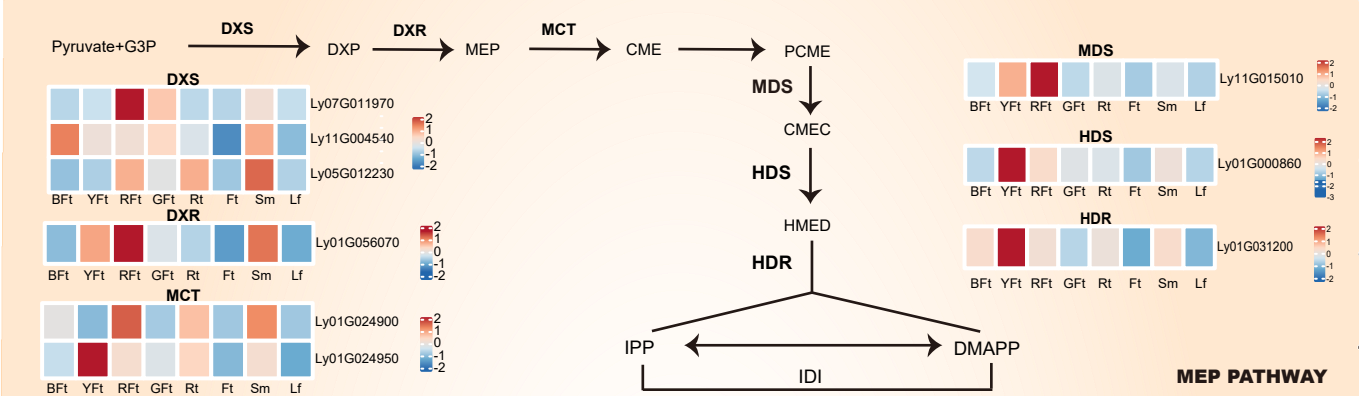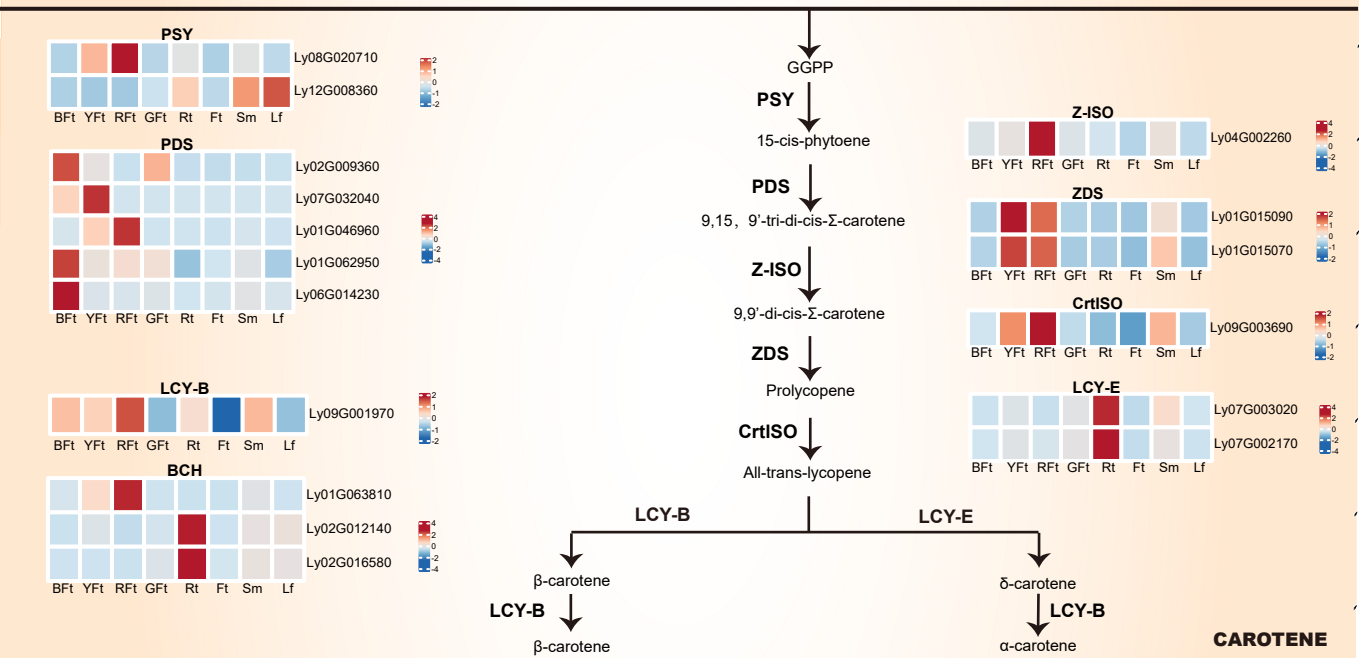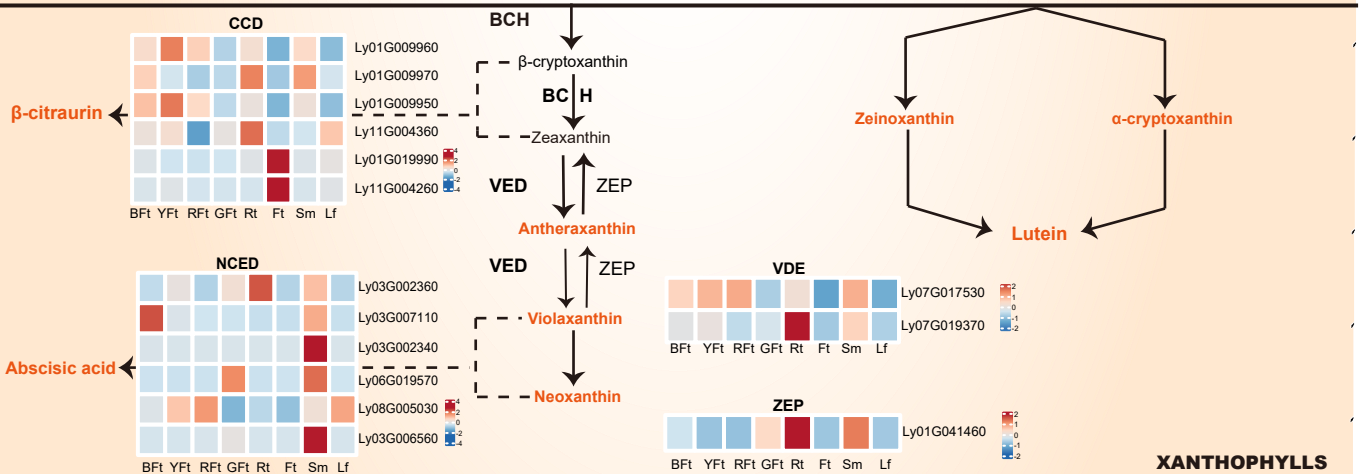

Supplement: qzae079_Supplementary_Data [file qzae079_supplementary_data.zip › Figure S17.pdf]

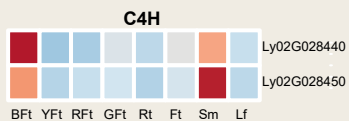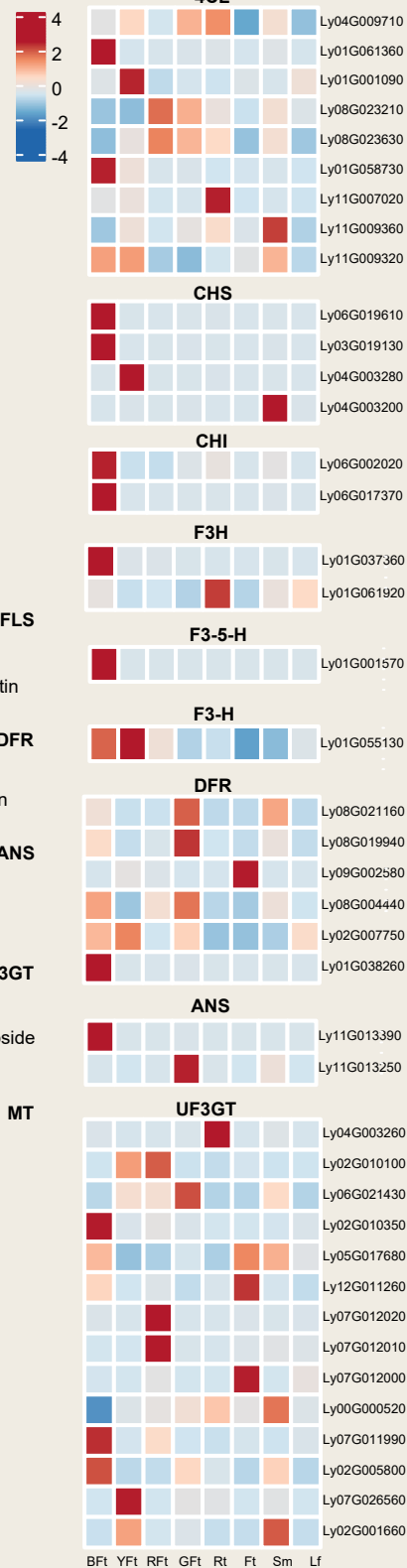

Supplement: qzae079_Supplementary_Data [file qzae079_supplementary_data.zip › Figure S18.pdf]

**A**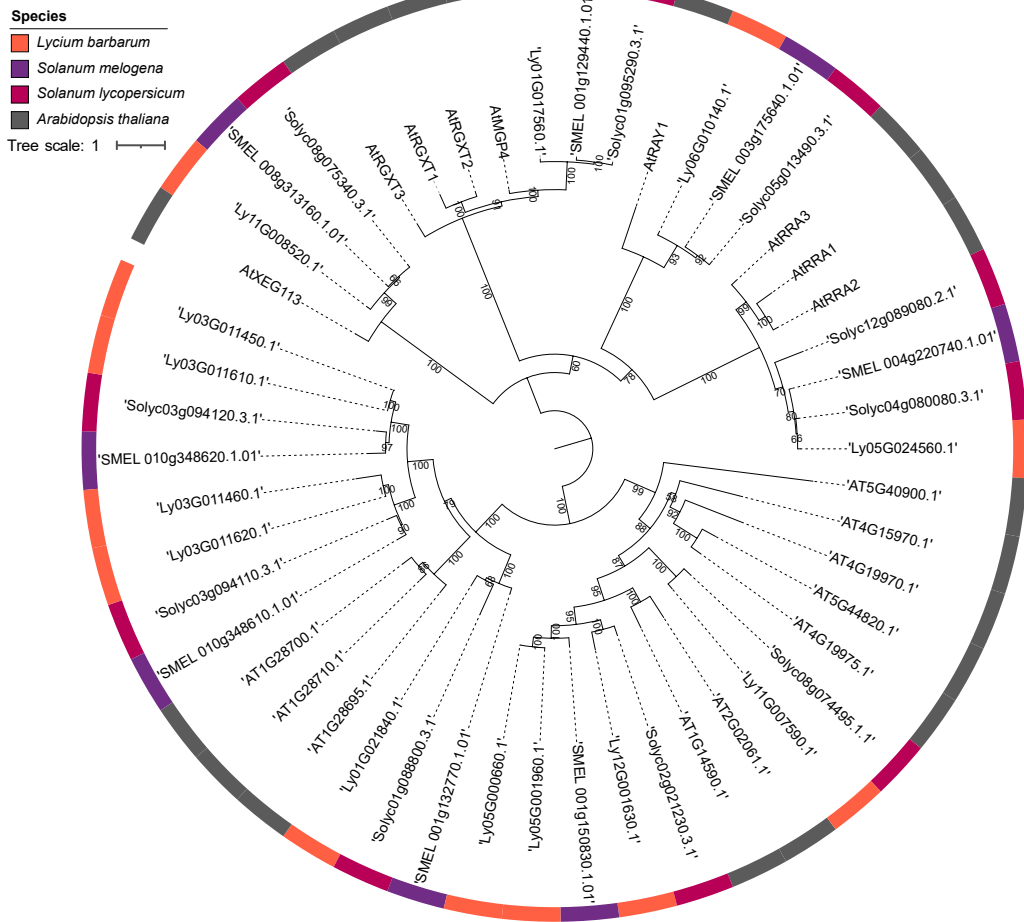**B**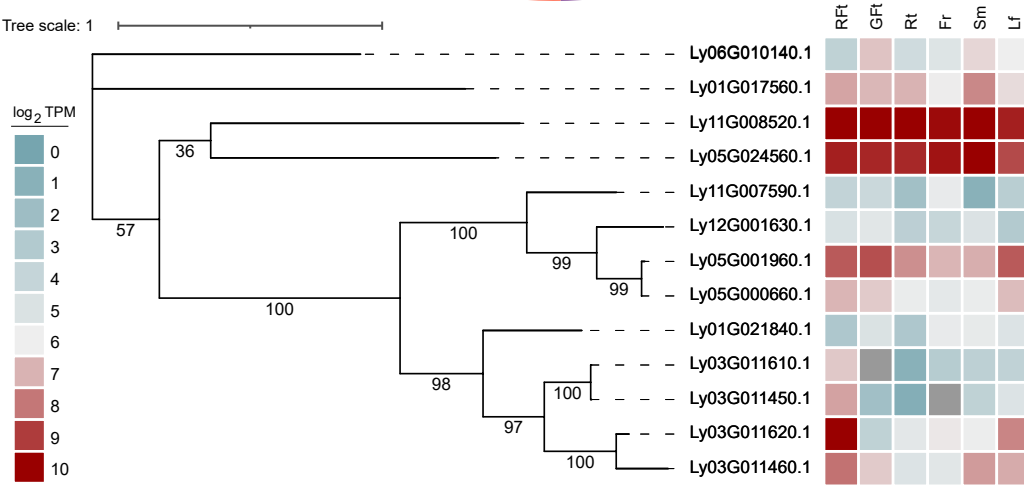

Supplement: qzae079_Supplementary_Data [file qzae079_supplementary_data.zip › Figure S19.pdf]

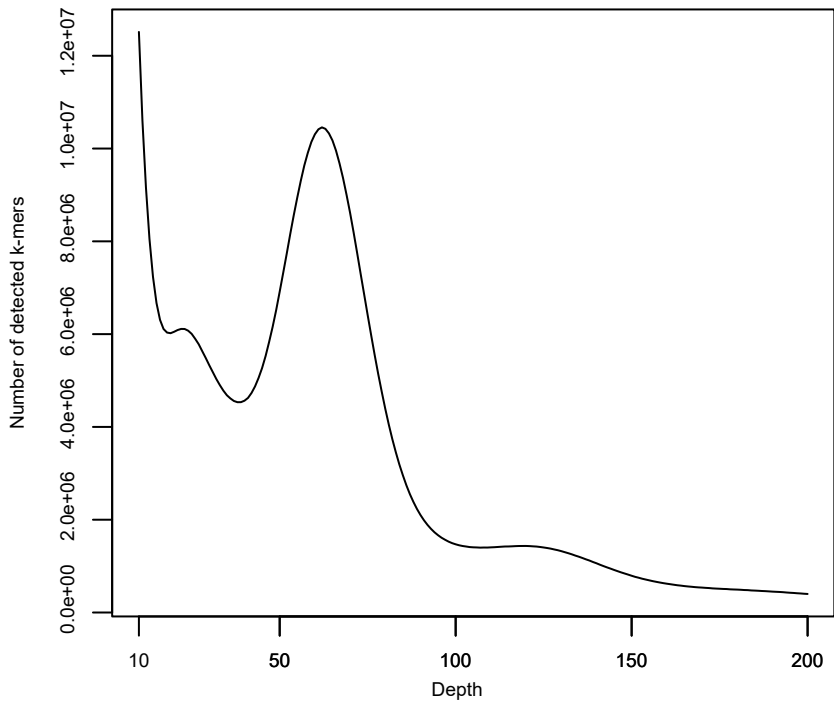

Supplement: qzae079_Supplementary_Data [file qzae079_supplementary_data.zip › Figure S2.pdf]

## A Species

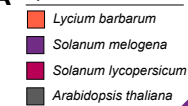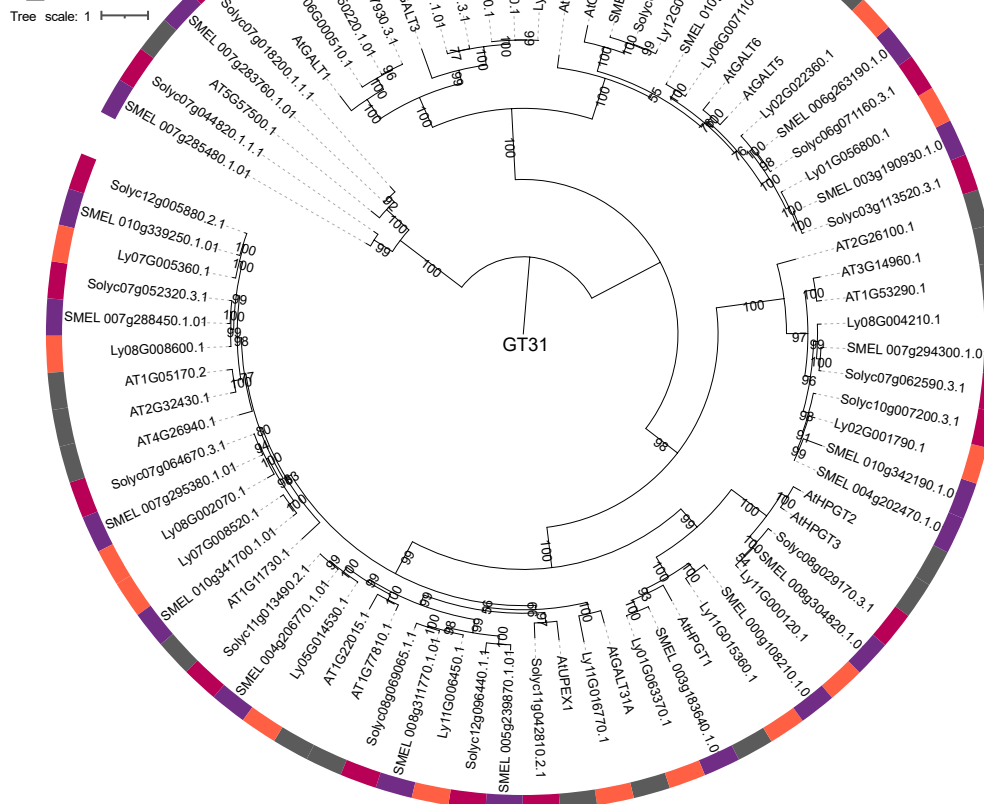

## B

Tree scale: 1

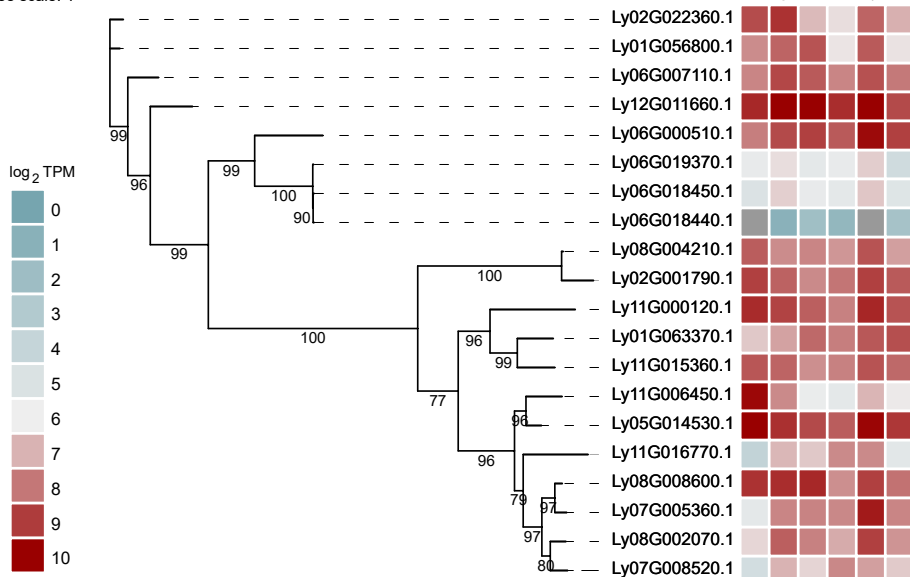

Supplement: qzae079_Supplementary_Data [file qzae079_supplementary_data.zip › Figure S20.pdf]

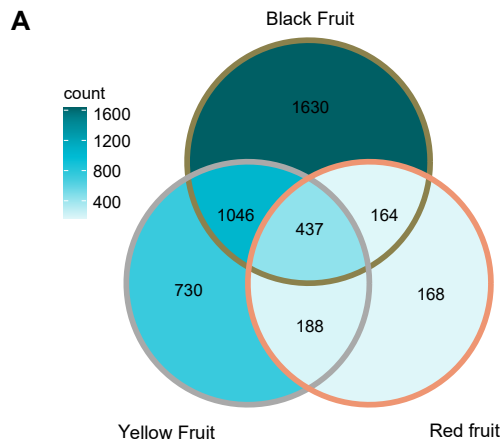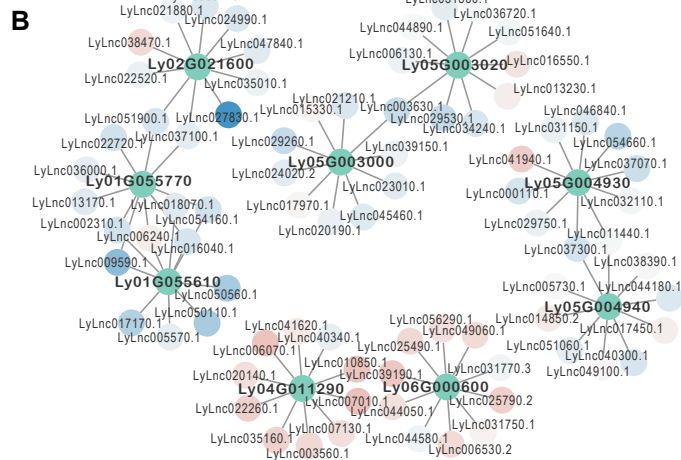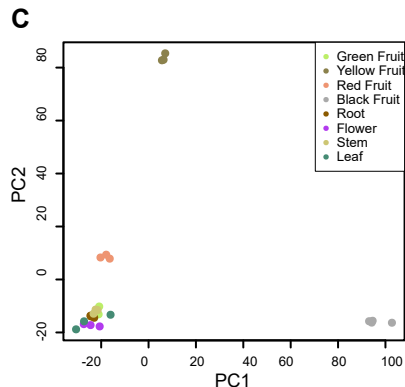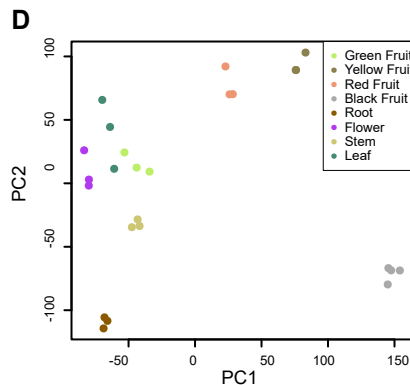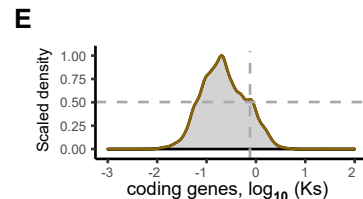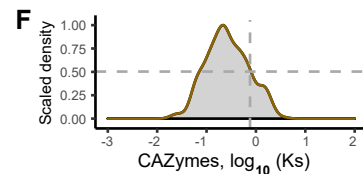

Supplement: qzae079_Supplementary_Data [file qzae079_supplementary_data.zip › Figure S21.pdf]

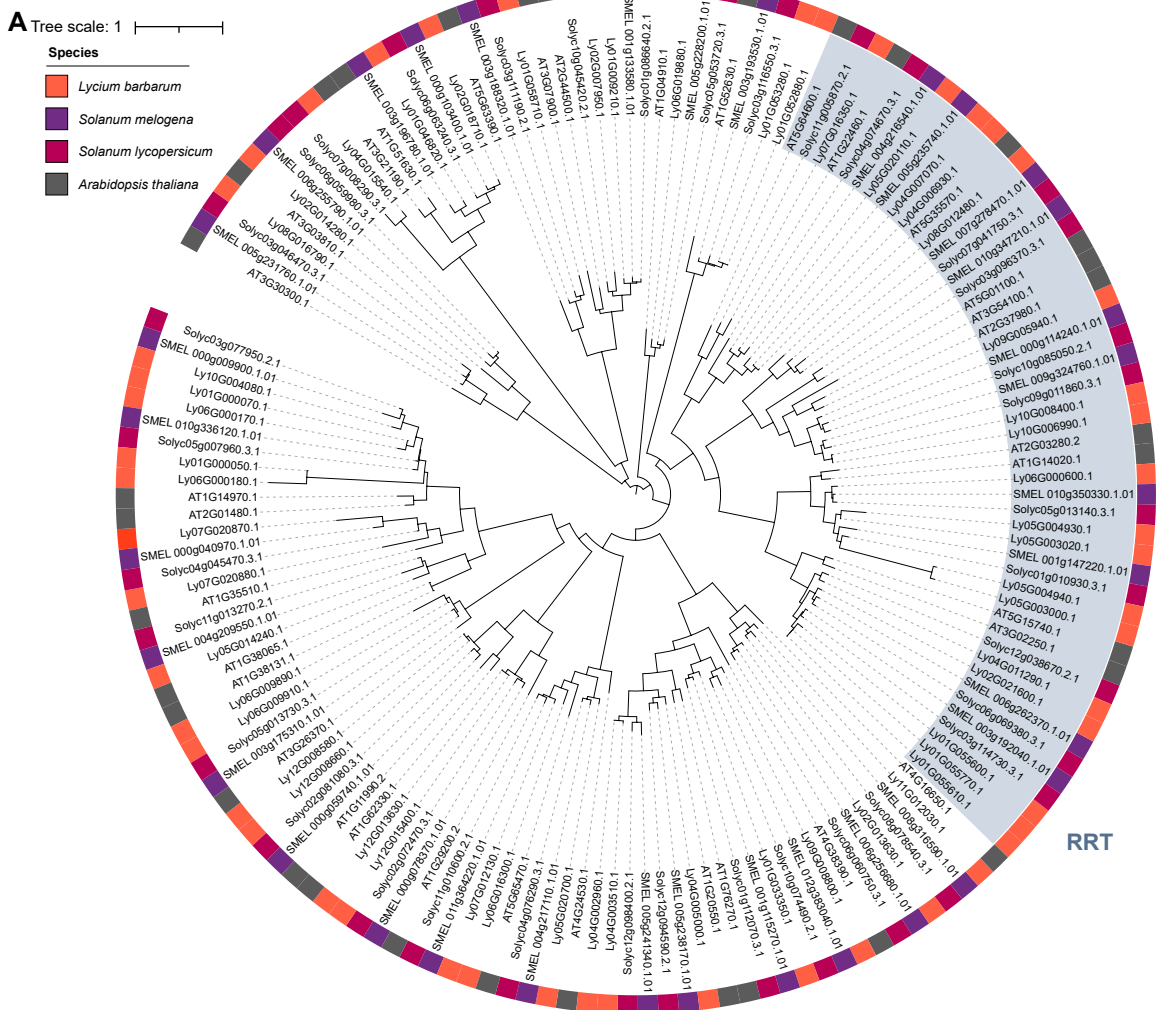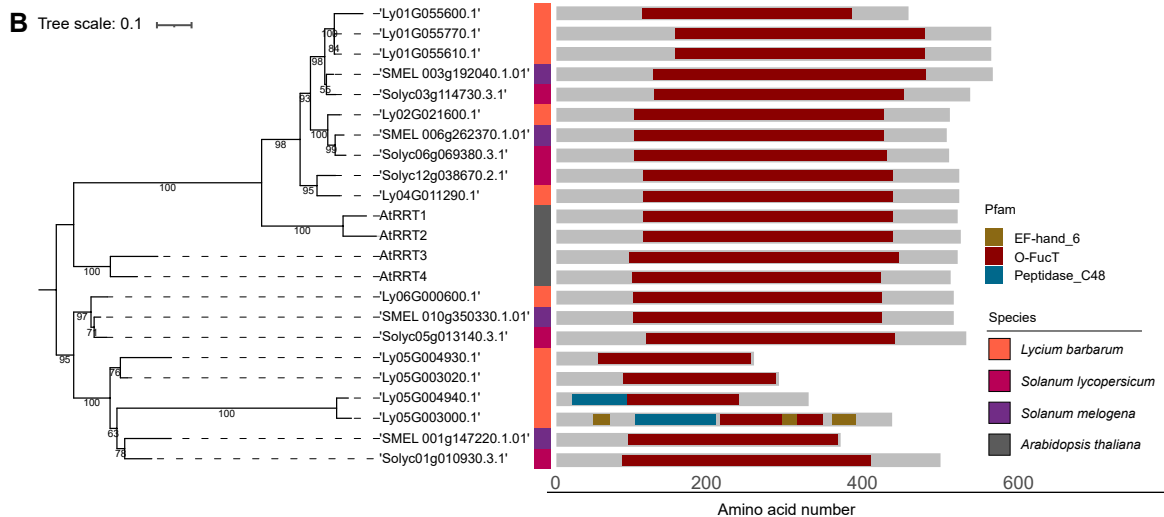

Supplement: qzae079_Supplementary_Data [file qzae079_supplementary_data.zip › Figure S22.pdf]

**A**

Tree scale: 1

 $\log_2$  TPM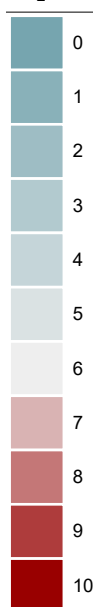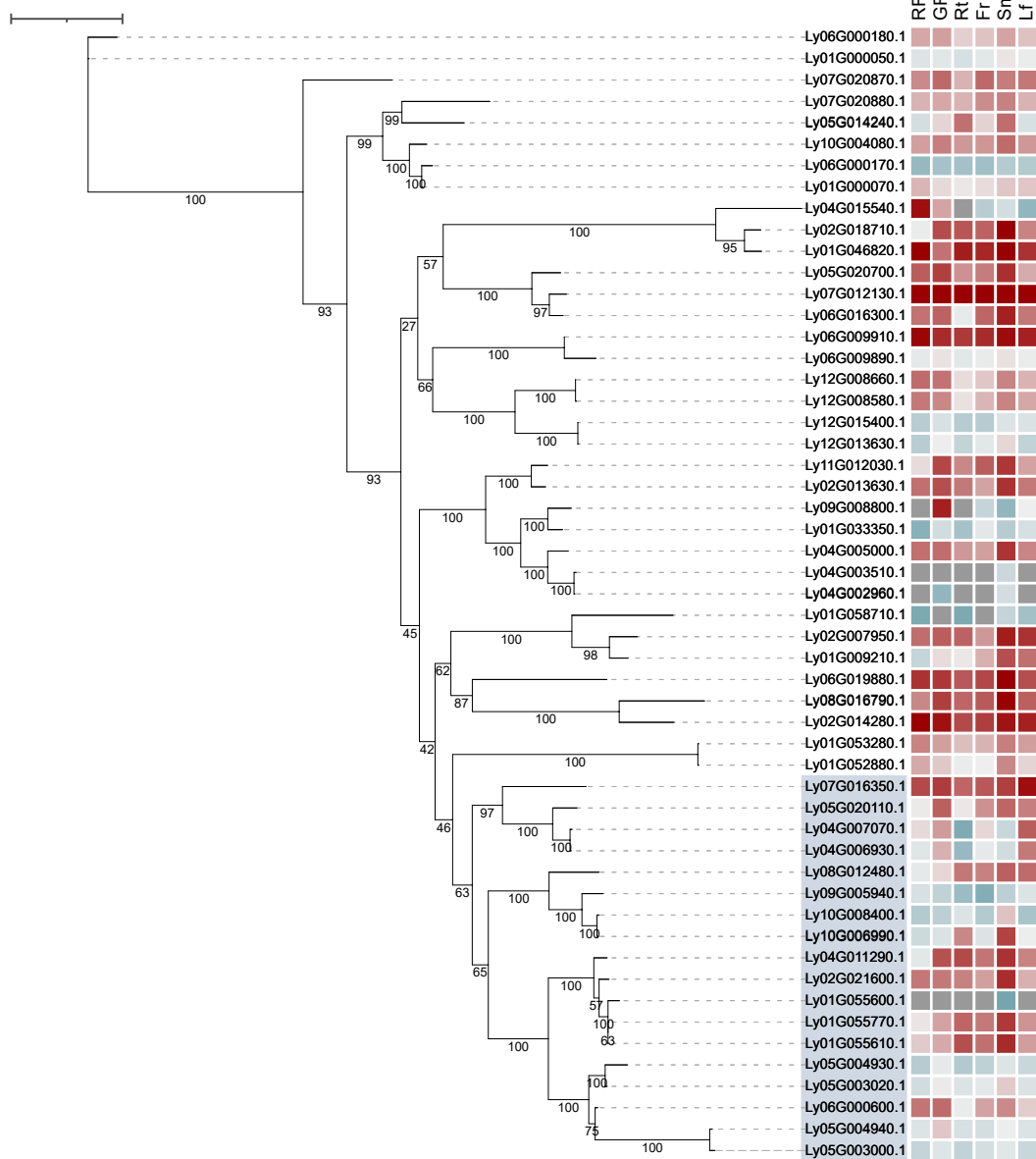**B***S. lycopersicum*

Soly01g010930.3.1

GT106 - RRT

*L. barbarum*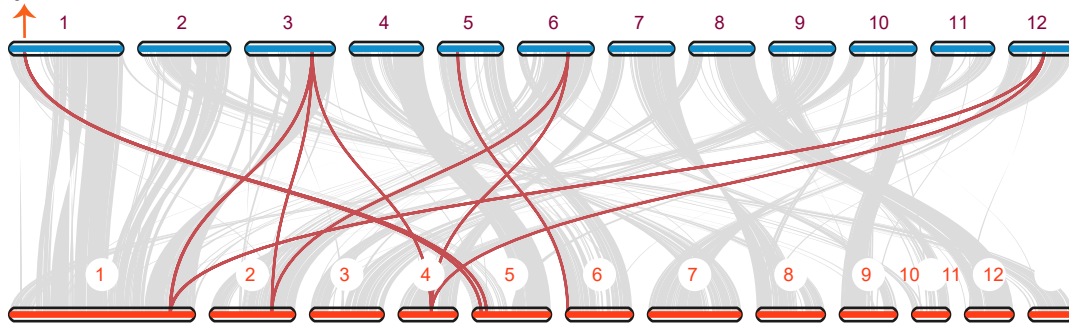

Ly05G003020 / Ly05G003000 / Ly05G004930 / Ly05G004940

Supplement: qzae079_Supplementary_Data [file qzae079_supplementary_data.zip › Figure S23.pdf]

**A**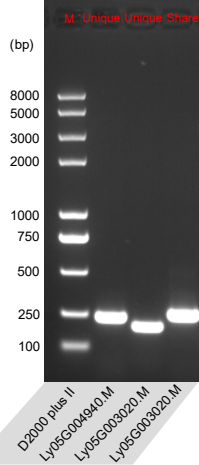**B**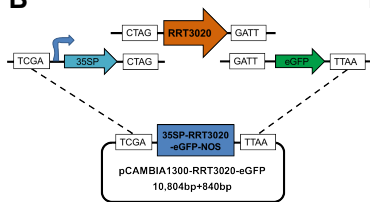**C**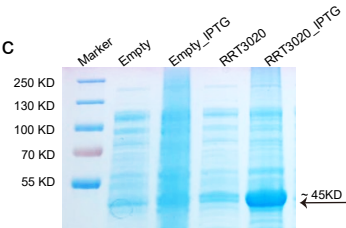**E**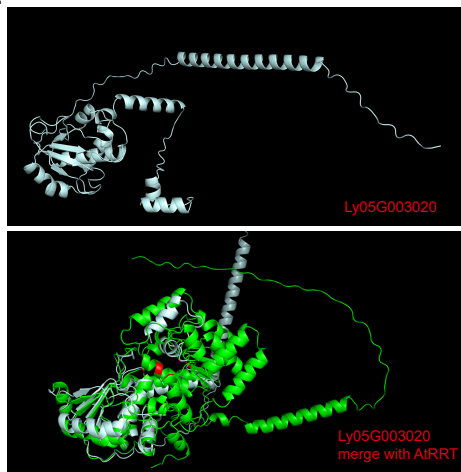**D**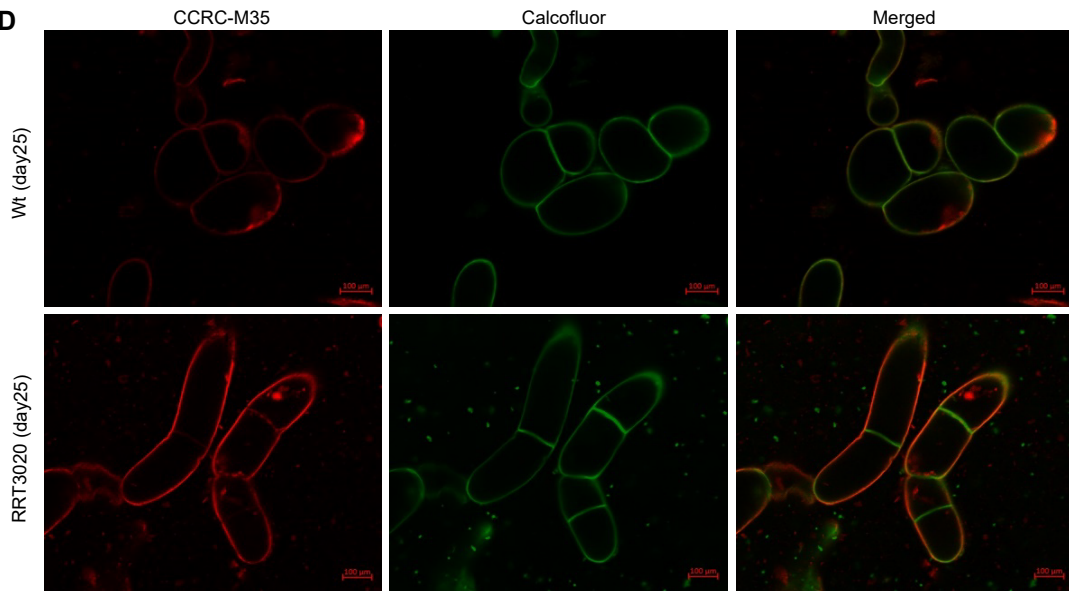

Supplement: qzae079_Supplementary_Data [file qzae079_supplementary_data.zip › Figure S24.pdf]

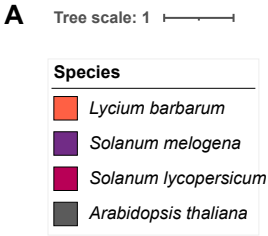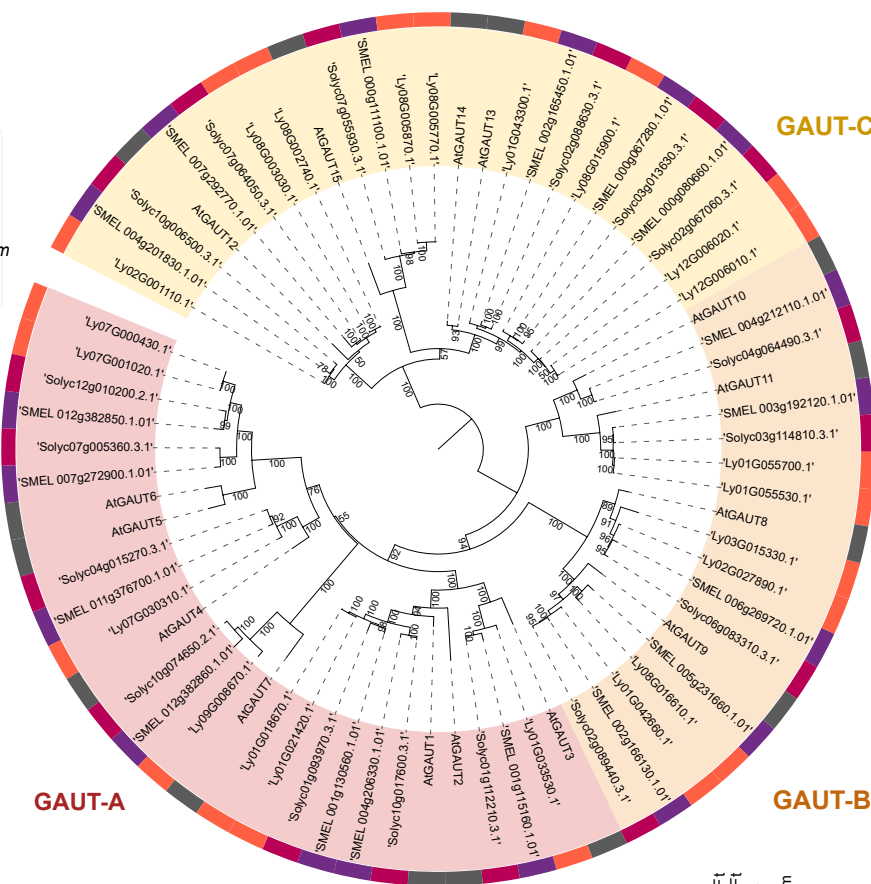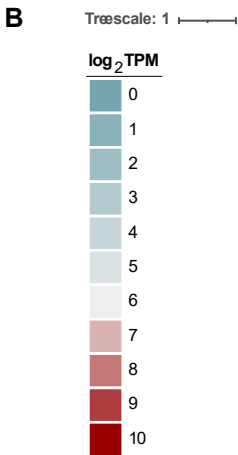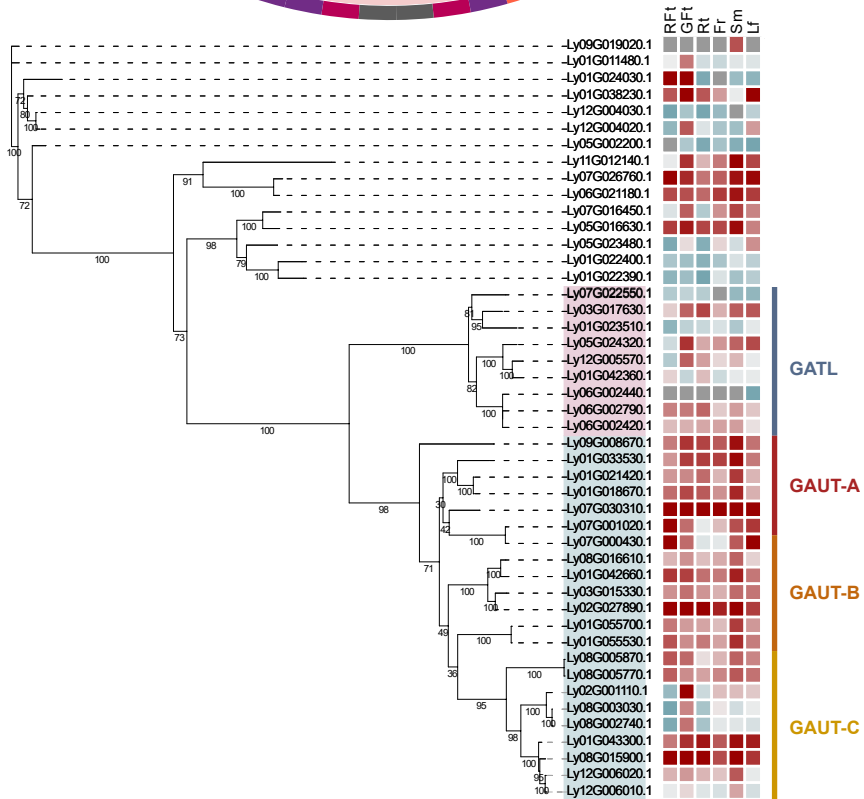

Supplement: qzae079_Supplementary_Data [file qzae079_supplementary_data.zip › Figure S25.pdf]

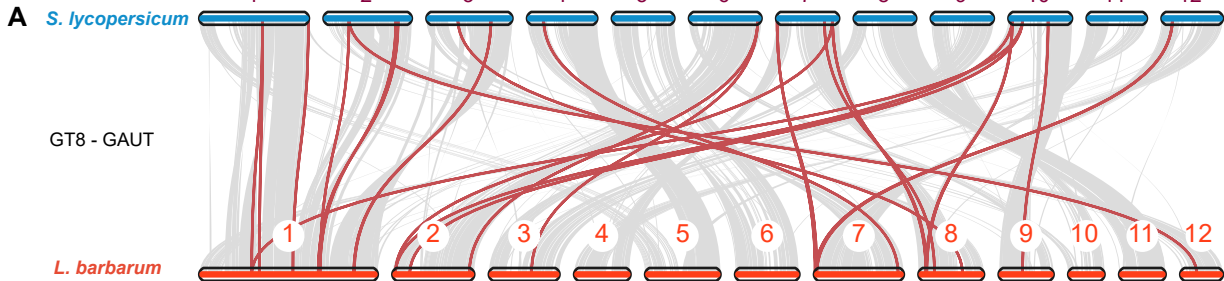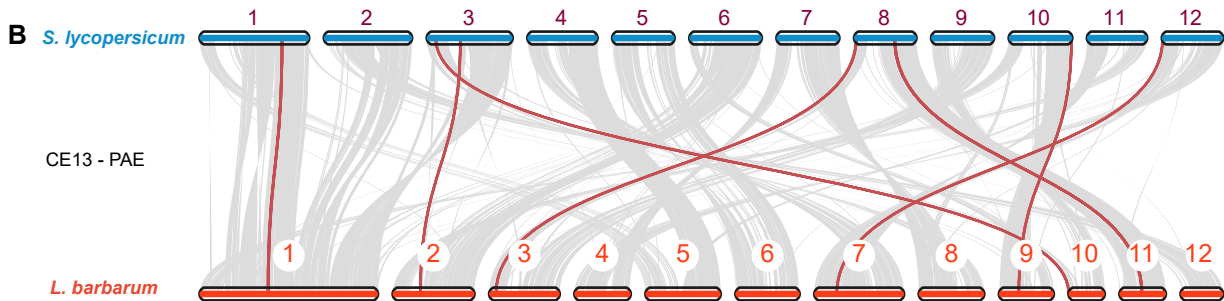

Supplement: qzae079_Supplementary_Data [file qzae079_supplementary_data.zip › Figure S26.pdf]

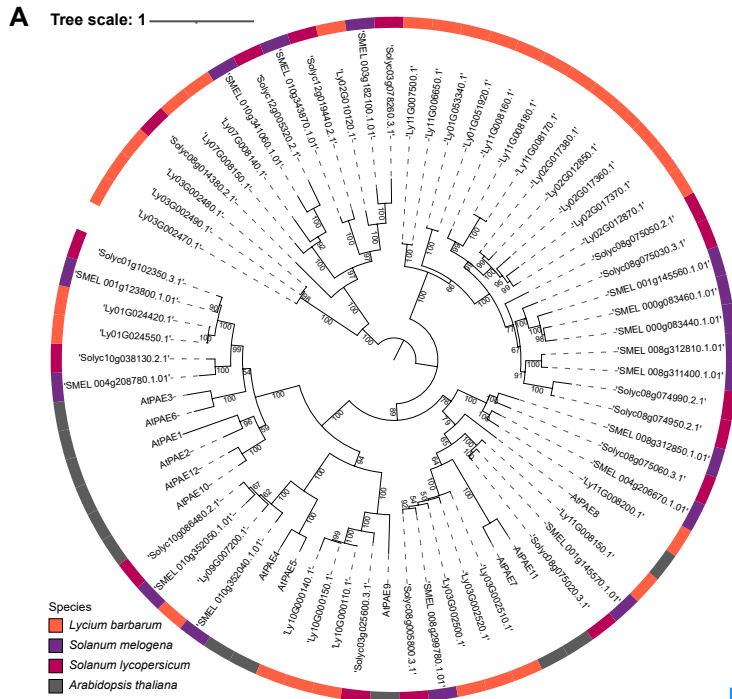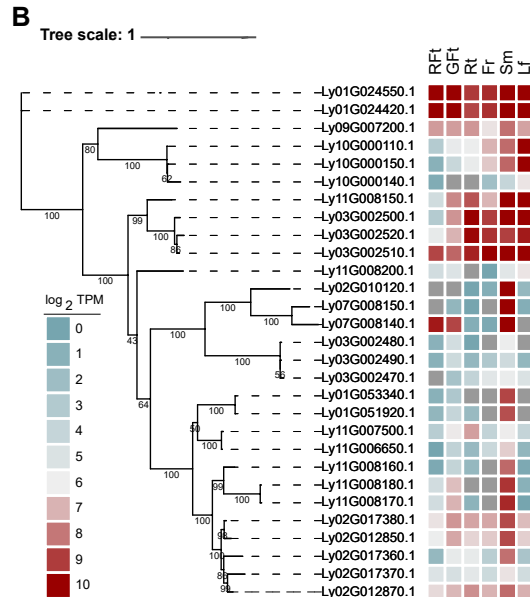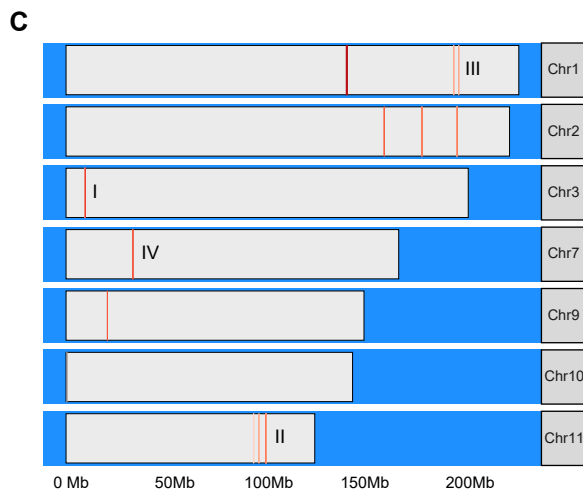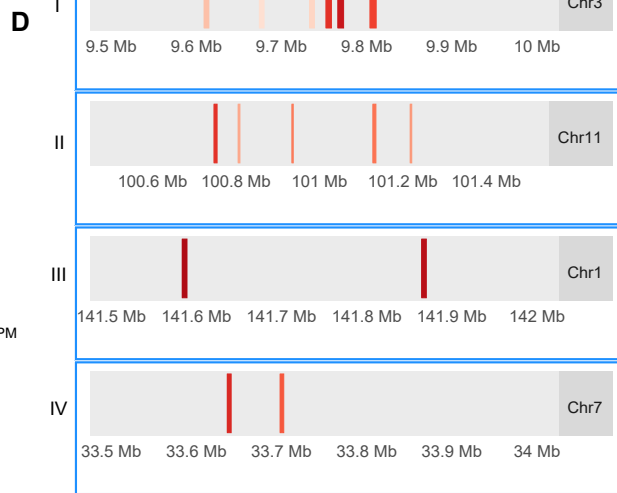

Supplement: qzae079_Supplementary_Data [file qzae079_supplementary_data.zip › Figure S27.pdf]

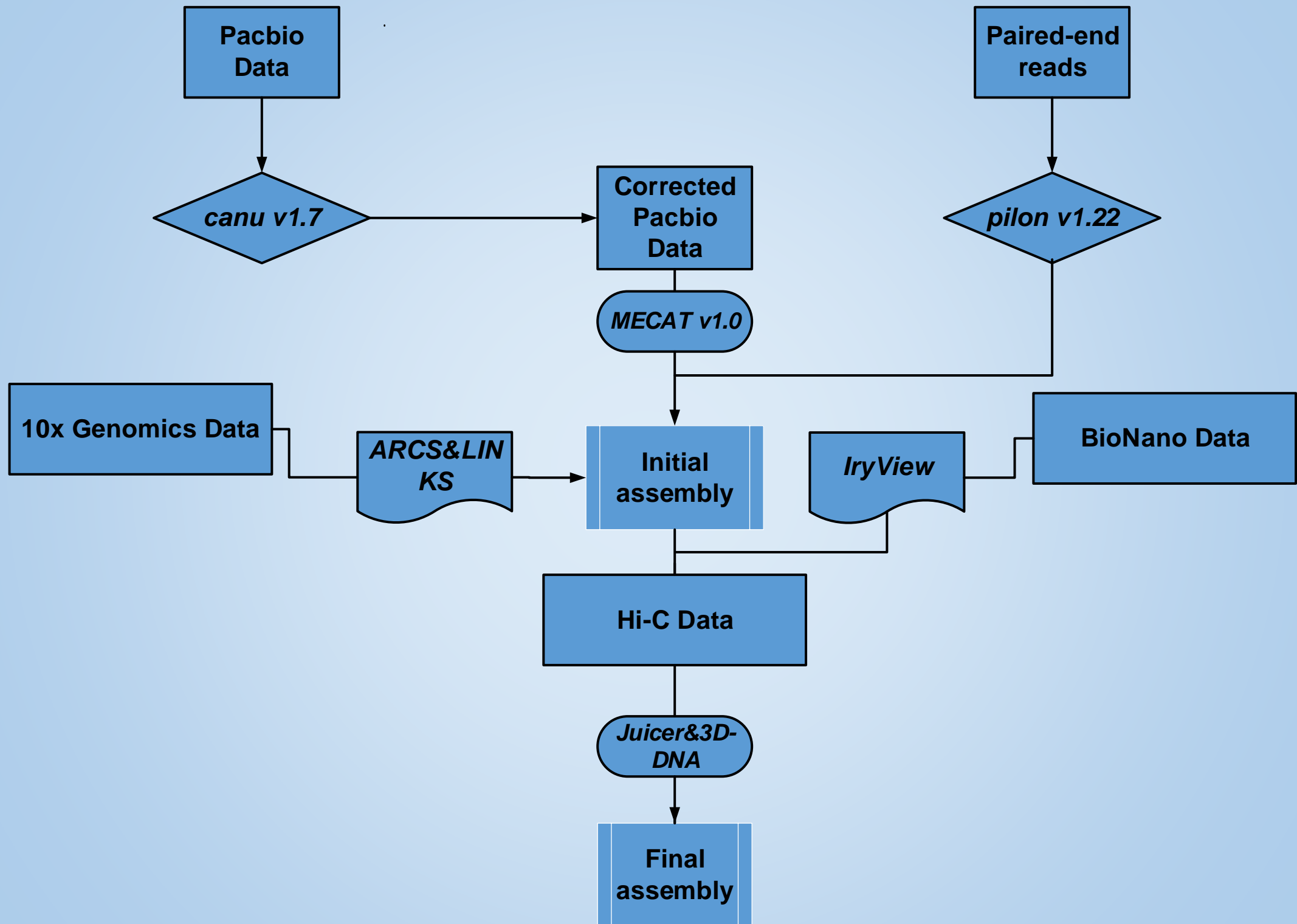

Supplement: qzae079_Supplementary_Data [file qzae079_supplementary_data.zip › Figure S3.pdf]

**A**

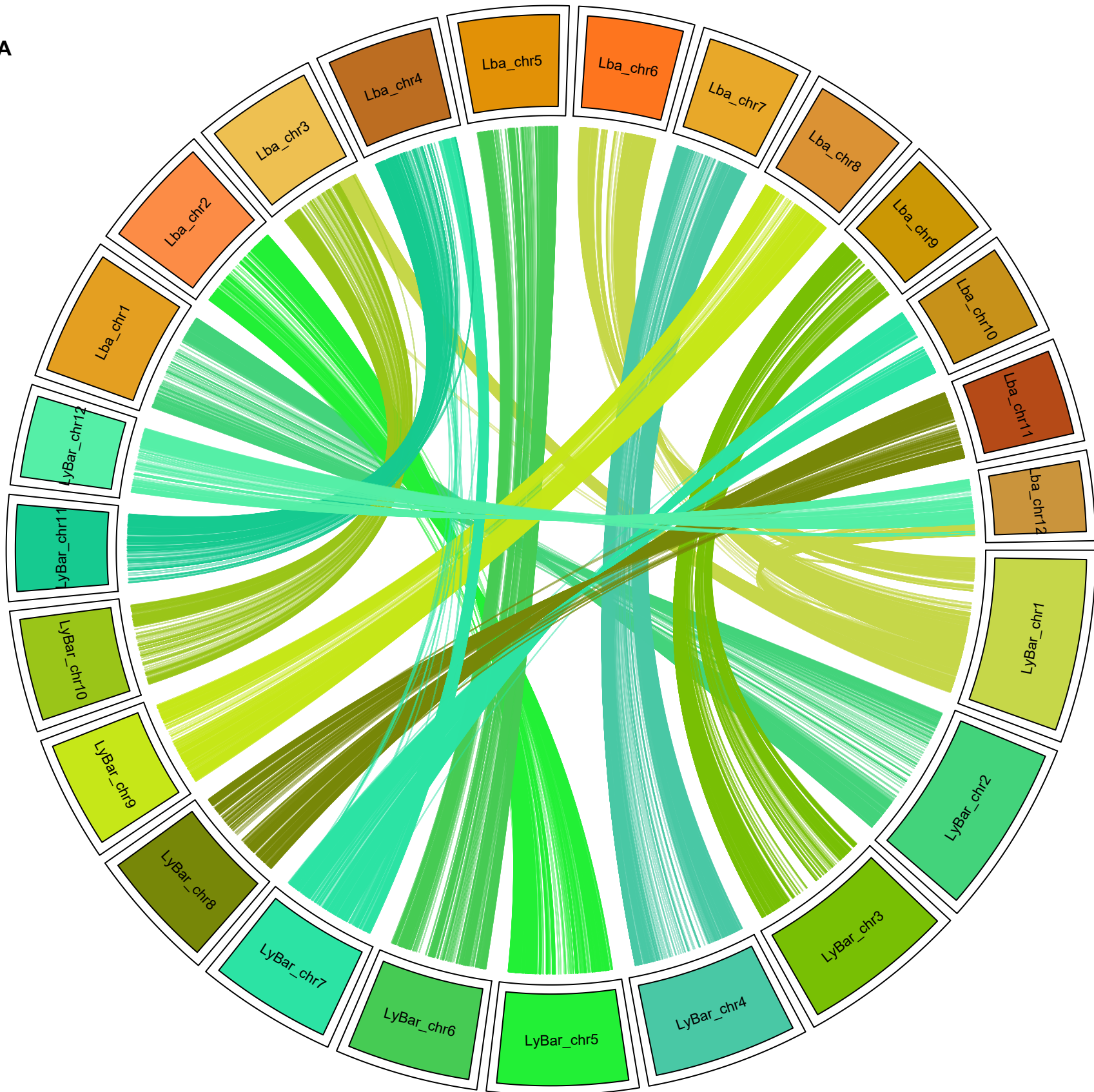

**B**

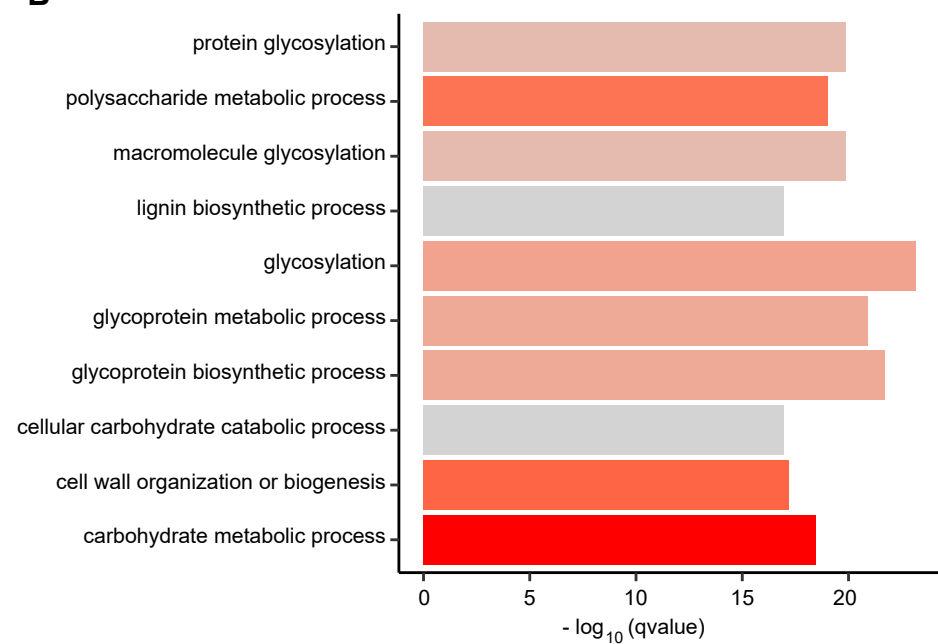

**C**

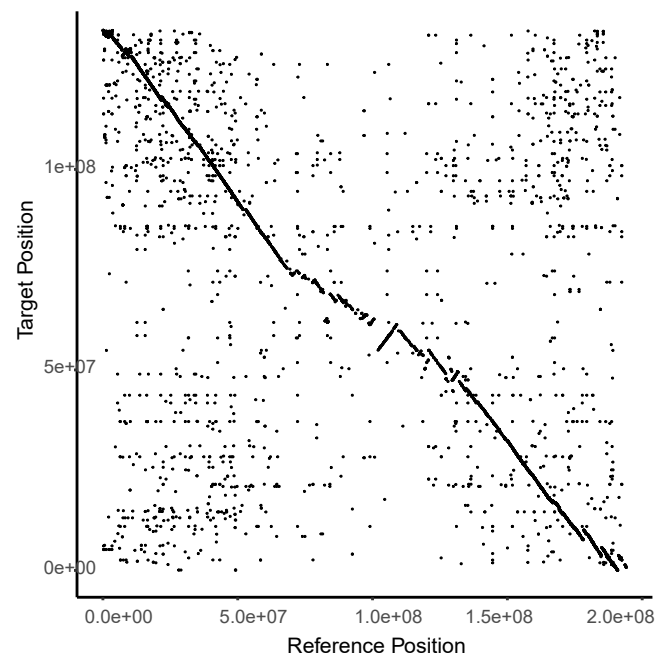

Supplement: qzae079_Supplementary_Data [file qzae079_supplementary_data.zip › Figure S5.pdf]

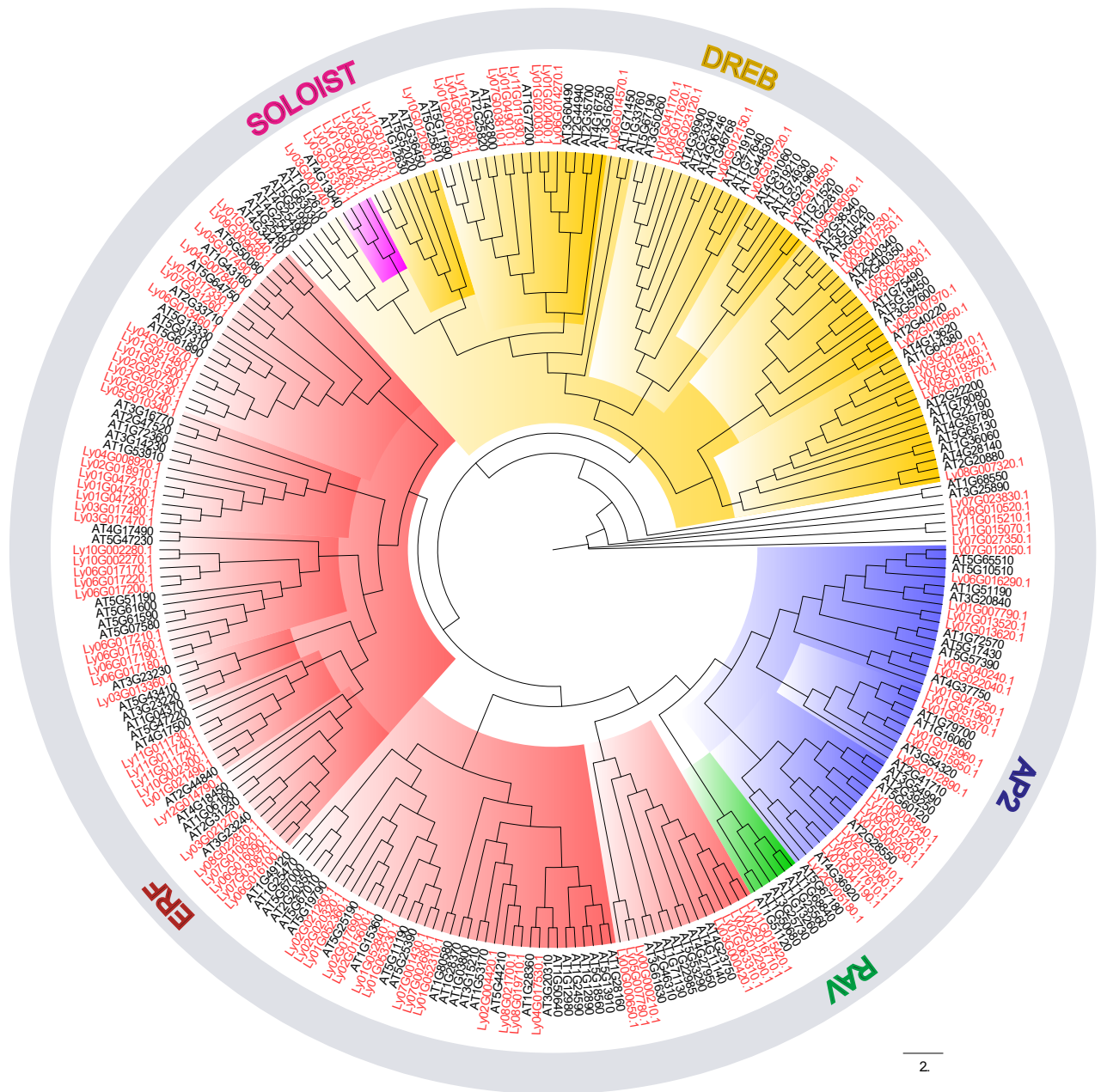

Supplement: qzae079_Supplementary_Data [file qzae079_supplementary_data.zip › Figure S6.pdf]

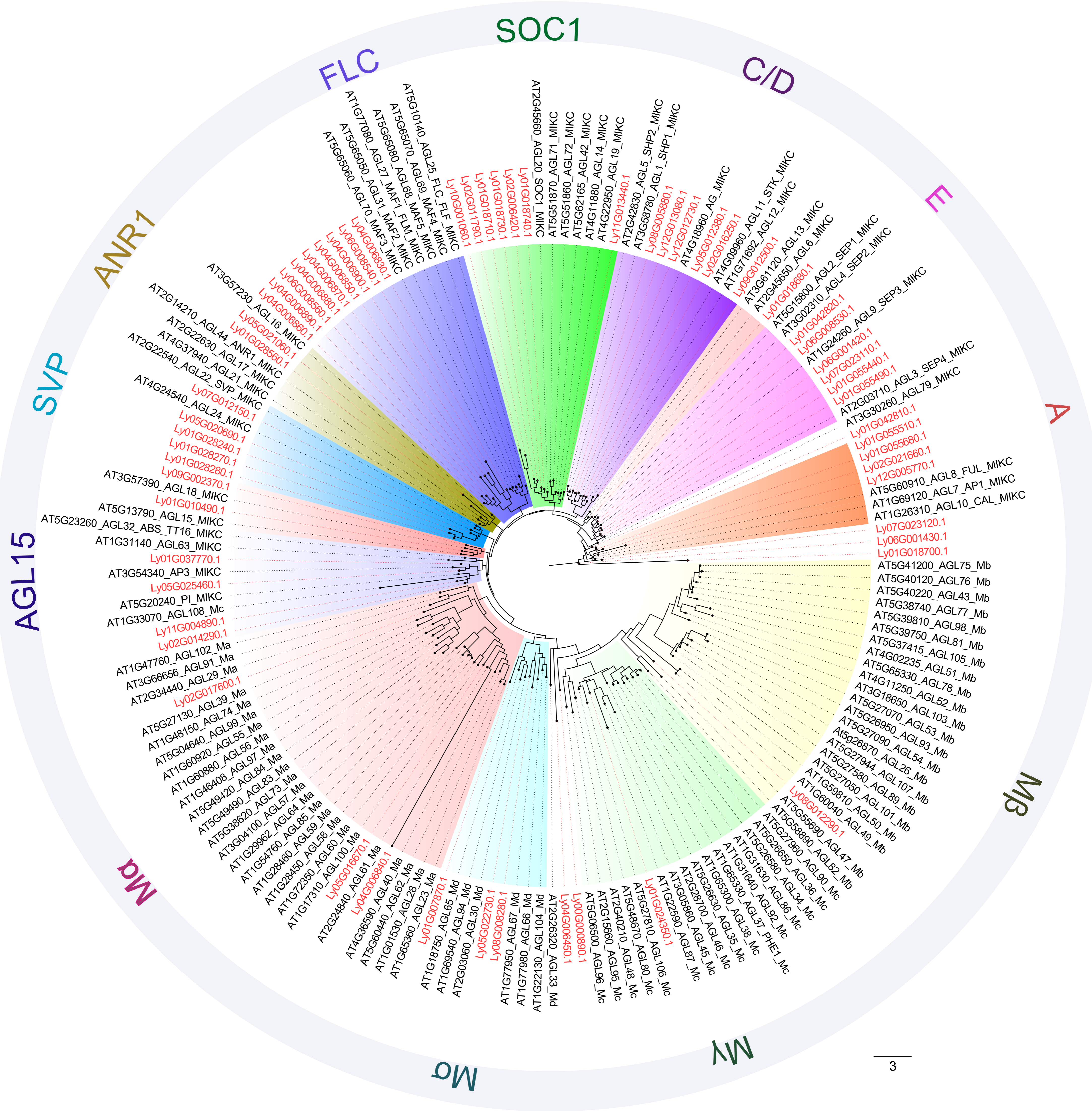

Supplement: qzae079_Supplementary_Data [file qzae079_supplementary_data.zip › Figure S7.pdf]

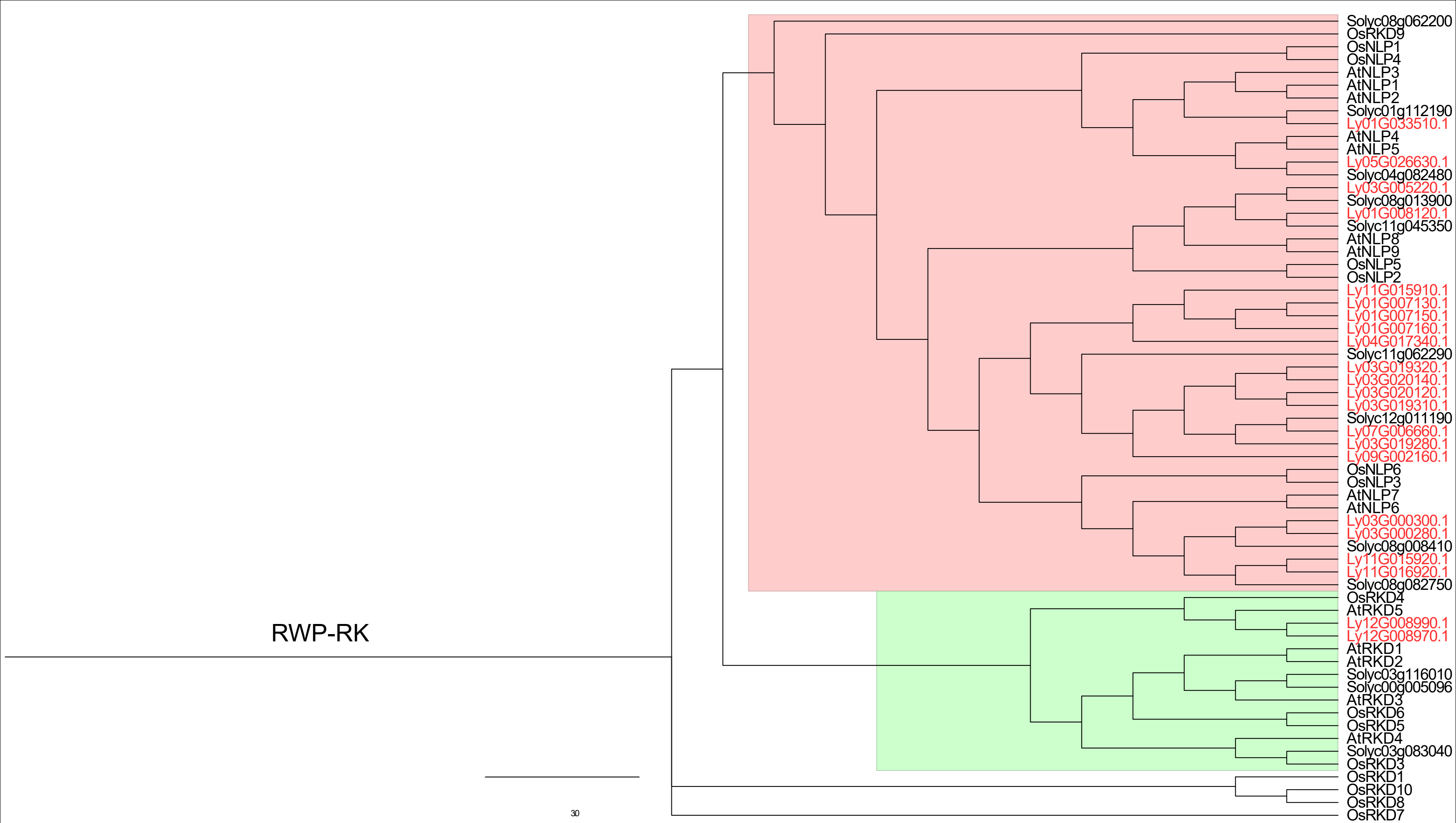

Supplement: qzae079_Supplementary_Data [file qzae079_supplementary_data.zip › Figure S8.pdf]

A

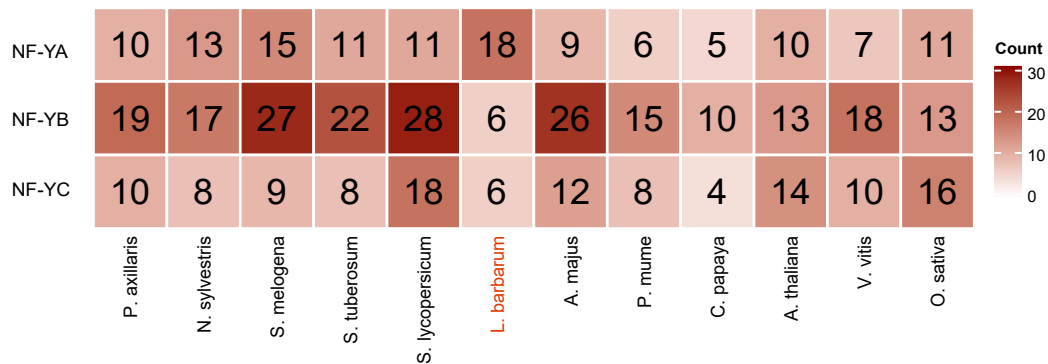

B

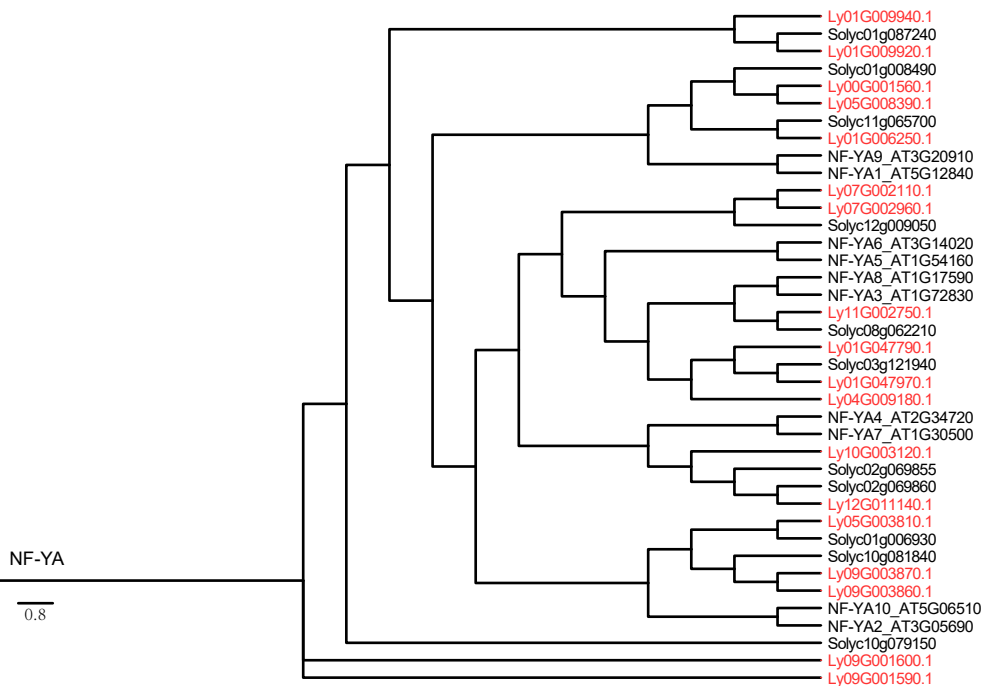

Supplement: qzae079_Supplementary_Data [file qzae079_supplementary_data.zip › Figure S9.pdf]
