# Supplementary material for: Enzyme Repertoires and Genomic Insights into Lycium barbarum Pectin Polysaccharide Biosynthesis
Source: Genomics Proteomics Bioinformatics. 2024 Nov 4;22(6):qzae079. doi: 10.1093/gpbjnl/qzae079 (PMC12011363; doi:10.1093/gpbjnl/qzae079)
Supplement: qzae079_Supplementary_Data [file Figure S4.pdf › Figure S4.pdf]

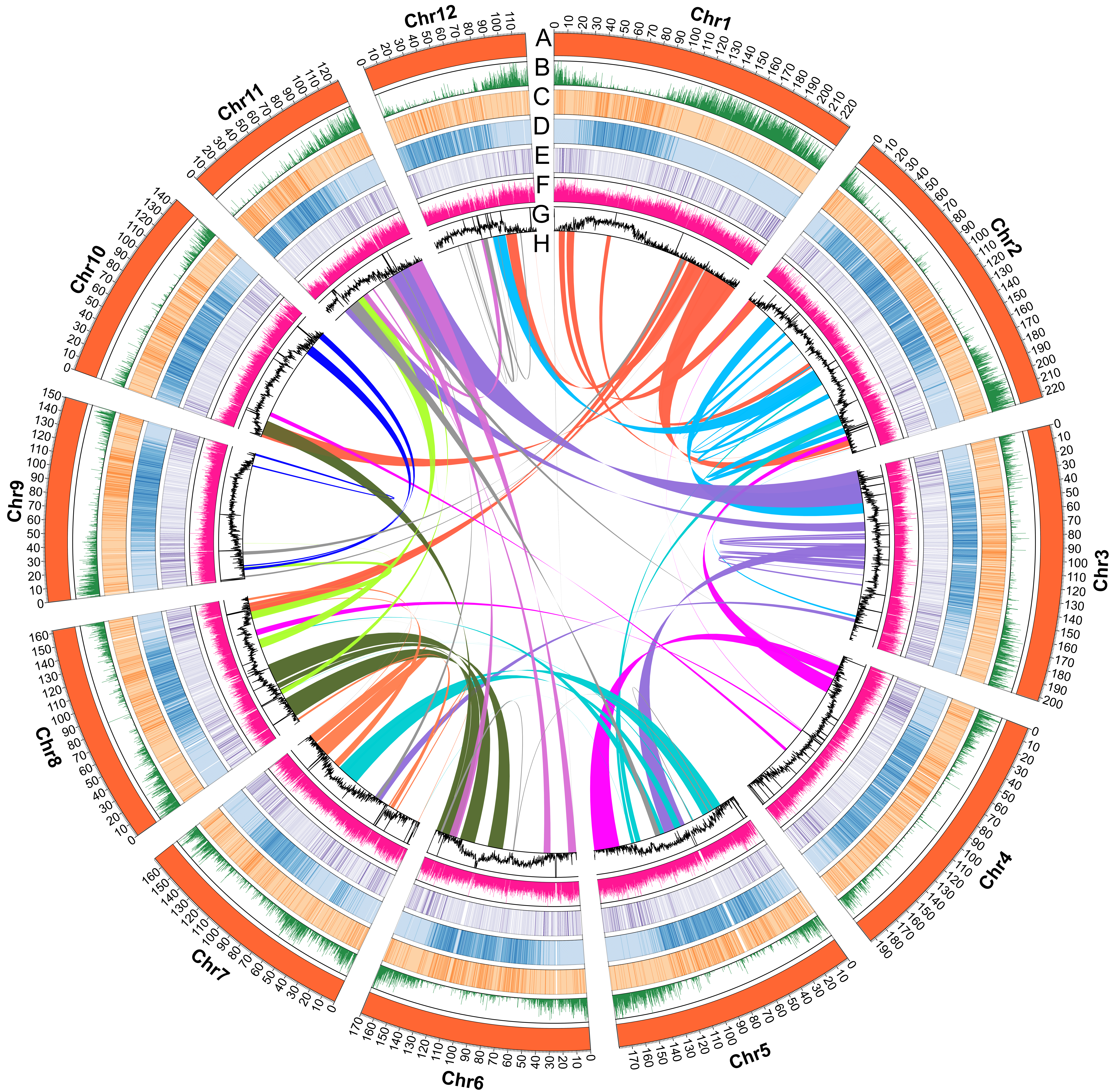

Chr1
Chr2
Chr3
Chr4
Chr5
Chr6
Chr7
Chr8
Chr9
Chr10
Chr11
Chr12
A
B
C
D
E
F
G
H
